# Photocatalytic deaminative benzylation and alkylation of tetrahydroisoquinolines with *N*-alkylpyrydinium salts

**DOI:** 10.3762/bjoc.16.74

**Published:** 2020-04-21

**Authors:** David Schönbauer, Carlo Sambiagio, Timothy Noël, Michael Schnürch

**Affiliations:** 1 Institute of Applied Synthetic Chemistry, TU Wien, Getreidemarkt 9/163, 1060 Vienna, Austria; 2 Department of Chemical Engineering and Chemistry, Micro Flow Chemistry and Synthetic Methodology, Eindhoven University of Technology, Den Dolech 2, 5612 AZ Eindhoven, The Netherlands

**Keywords:** C–H functionalization, C(sp^3^)–C(sp^3^) coupling, deaminative coupling, Katritzky salt, photoredox catalysis

## Abstract

A ruthenium-catalyzed photoredox coupling of substituted *N*-aryltetrahydroisoquinolines (THIQs) and different bench-stable pyridinium salts was successfully developed to give fast access to 1-benzyl-THIQs. Furthermore, secondary alkyl and allyl groups were also successfully introduced via the same method. Additionally, the typically applied *N*-phenyl group in the THIQ substrate could be replaced by the cleavable *p*-methoxyphenyl (PMP) group and successful N-deprotection was demonstrated.

## Introduction

The selective formation of new carbon–carbon bonds via direct C–H functionalization bears the potential of being a process of high efficiency [1–3]. Since C–H bonds are omnipresent in organic compounds it is very appealing to exploit them as “functional groups”, avoiding tedious prefunctionalization. However, the ubiquity of C–H bonds gives rise to another problem, namely selectivity. Since the bond strength of C–H bonds in a typical organic compound varies only by a few kcal/mol, it is difficult to address a single C–H bond selectively without compromising the others. To circumvent this problem, directing groups can be installed, which guide a metal catalyst to a specific C–H bond [4–5]. In case the directing group is not needed in the final product, this strategy accounts for additional reaction steps for the installation and removal of the directing group. However, there is also a good number of substrates containing C–H bonds that are inherently more reactive than others in the same molecule, and in such cases a selective C–H functionalization can be achieved in the absence of any directing group [2,6–7]. For example, in tetrahydroisoquinolines (THIQs) the benzylic C1-position is significantly more reactive compared to the others and its selective functionalization has been reported [8]. The THIQ moiety is of special interest due to its presence in several different natural products [9] and pharmaceuticals [10–11]. Consequently, the direct functionalization of this scaffold has attracted significant interest in recent years. Even though many transformations have been realized via a cross-dehydrogenative coupling approach [12–13] (e.g., arylations [14–15], cyanomethylation [16], alkynation [17–18], or allylation [19]), this method has certain drawbacks, most importantly the frequent requirement of superstoichiometric amounts of an oxidant. Hence, alternative methods were investigated and photoredox catalysis proved to be a viable option [20–22].

**Scheme 1:**
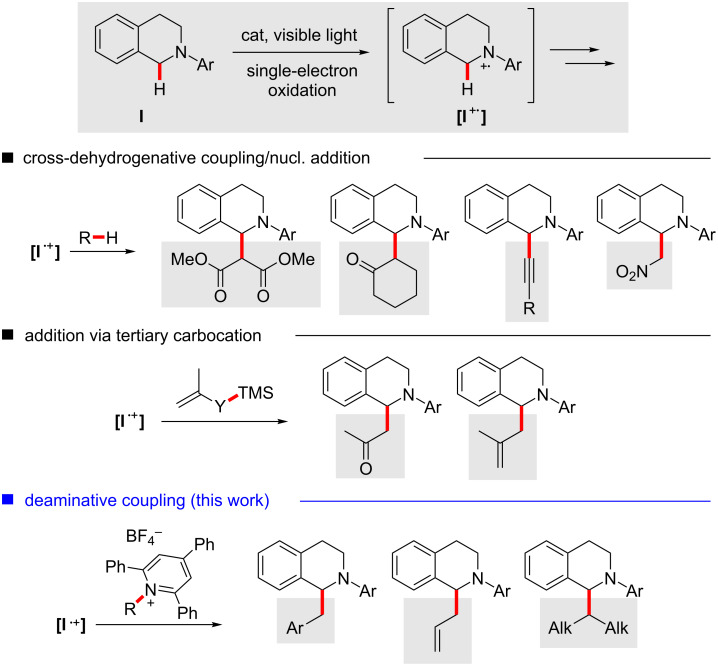
Examples of photocatalytic C–C bond formation by nucleophilic trapping of a reactive THIQ intermediate.

By now, several different methods for C–C bond formation at the α position of these amines have been studied under photoredox catalysis (Scheme 1) [23–26].

Among the functionalized THIQs, 1-benzyl-substituted analogues were shown to be able to modulate Ca/K channels, and the synthesis of these compounds is therefore of interest [27]. In our continuing quest to develop methods for the introduction of pure hydrocarbon residues avoiding volatile reagents [28–29], our attention was drawn to stable *N*-alkyl-(2,4,6-triphenyl)pyridinium salts (Katritzky salts) as alkylation reagents in the context of non-directed C–H functionalization. These salts are known since the late 1970s [30], but only in the last few years they have found application in a wide variety of radical processes, initiated by a single-electron reduction of the pyridinium salts, and subsequent generation of alkyl radicals [31–34]. Among these, electrophilic alkyl radicals were used in several transformations, such as electrophilic cross couplings under nickel catalysis, either with boronic acids [35] or different (aryl)halides [36–38]. Furthermore, visible light-promoted uncatalyzed electron transfer via the formation of electron donor–acceptor (EDA) complexes [39] was established between Hantzsch esters and pyridinium salts generating radicals, which were coupled with several substrates [40]. Later, methods without sacrificial reductant were also established, forming an EDA complex between the used substrates. Examples of this strategy are borylations [41–42], heteroarylations [43], and thioesterifications [44]. Additionally, several methods using photoredox chemistry were also published. For instance, the alkylation of isoquinolines under iridium catalysis and alkynylation with eosin Y as the catalyst [45–46].

In this contribution, we demonstrate the deaminative coupling of *N*-benzylpyridinium Katritzky salts with THIQs under ruthenium photoredox catalysis. During the preparation of this article, a similar transformation was disclosed using an iridium photoredox catalyst [47].

## Results and Discussion

**Scheme 2:**
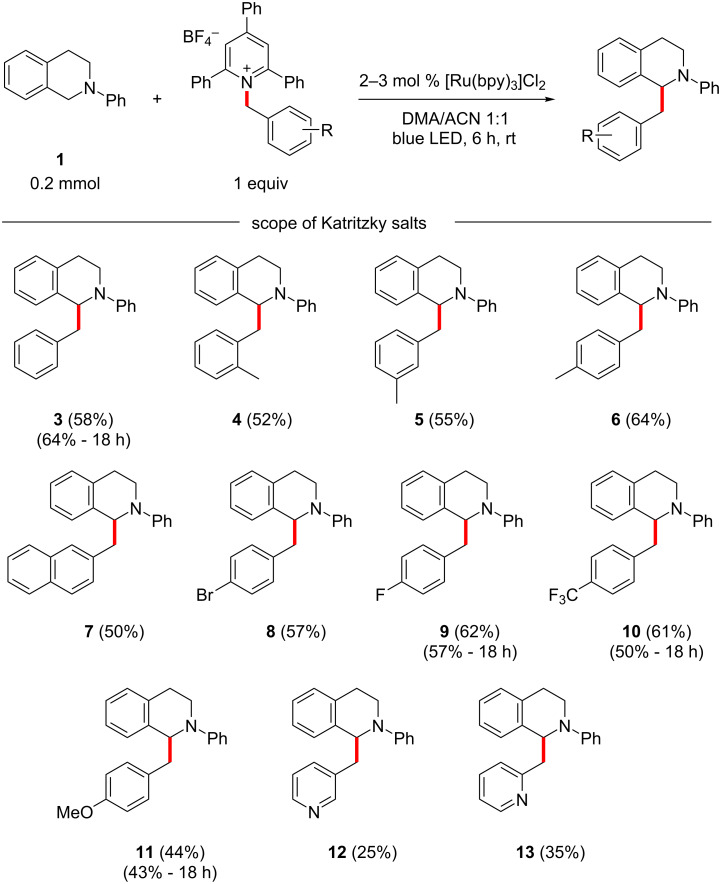
Benzylation of *N*-phenyl-THIQ.

We started our investigations by screening the coupling between *N*-phenyl-THIQ (**1**) with *N*-benzyl-(2,4,6-triphenyl)pyridinium tetrafluoroborate (**2**, Table 1; the complete optimization table can be found in Supporting Information File 1). First, a screening of different photocatalysts was performed (Table 1, entries 1–4). The best results were obtained with [Ru(bpy)_3_]Cl_2_, albeit the reaction was still low-yielding (29%, Table 1, entry 1) and was accompanied with substantial decomposition of the starting material. Eosin Y, fluorescein, and [Ir(dtbbpy)(ppy)_2_]PF_6_ gave lower yields in comparison (Table 1, entries 2, 3 and 4). Applying strictly inert conditions did not improve the yield, but decomposition of **1** was significantly reduced (Table 1, entry 5). Next, a solvent screening (Table 1, entries 6–12) was performed, showing that reactions in DMSO, DMA, DMF, or DMA/ACN 1:1 improved the yield to >50%, with mass balances of 78–91%. DCE and DCM had no beneficial effects on the yield. Different ratios of substrate and salt had also no impact on the yield (Table 1, entries 13 and 14). However, the isolated yield was increased to 67% when using a 1:1 mixture of DMA/ACN and a concentration of 0.05 M instead of 0.10 M (Table 1, entry 15). Additionally, the catalyst loading could be decreased to 2 mol % without negative effects (cf. entries 15 and 16 in Table 1). Further dilution gave no further improvement in the yield (see Supporting Information File 1). Next, increasing the power of the LEDs from 6 to 18 W gave 73% GC yield and a 64% isolated yield of **3** (Table 1, entry 17; see also Scheme 2). Different catalysts were then reinvestigated using the DMA/ACN solvent mixture at 0.05 M concentration. [Ru(bpy)_3_](PF_6_)_2_ led to a decreased yield of 57% (Table 1, entry 18), whereas [Ir(dtbbpy)(ppy)_2_]PF_6_ showed a comparable efficiency (69%, Table 1, entry 19). Eosin Y was inefficient under these conditions (Table 1, entry 20). Since [Ru(bpy)_3_]Cl_2_ is significantly cheaper than the iridium catalyst, the former was used in further reactions. Regarding the addition of different bases, the yield remained unchanged, when lutidine was added. Potassium carbonate and DIPEA reduced the yield substantially (Table 1, entries 21–23). Separate control experiments without catalyst (Table 1, entry 24) or in the dark (Table 1, entry 25) gave no or little conversion to the product, indicating the catalytic role of the ruthenium complex.

**Table 1:** Selected optimization of the reaction conditions.^a^

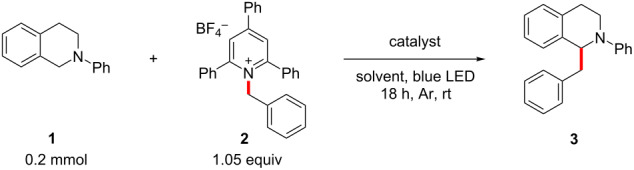				

entry	catalyst [5 mol %]	solvent	**1**	**3**

1^b^	[Ru(bpy)_3_]Cl_2_	ACN (0.1 M)	14%	29%
2^b^	eosin Y	ACN (0.1 M)	36%	21%
3^b^	fluorescein	ACN (0.1 M)	74%	13%
4	[Ir(dtbbpy)(ppy)_2_]PF_6_	ACN (0.1 M)	9%	21%
5^c^	[Ru(bpy)_3_]Cl_2_	ACN (0.1 M)	54%	30%
6^d^	[Ru(bpy)_3_]Cl_2_	ACN (0.1 M)	6%	33%
7	[Ru(bpy)_3_]Cl_2_	DCE (0.1 M)	63%	22%
8	[Ru(bpy)_3_]Cl_2_	DCM (0.1 M)	55%	25%
9	[Ru(bpy)_3_]Cl_2_	DMSO (0.1 M)	27%	55%
10	[Ru(bpy)_3_]Cl_2_	DMA (0.1 M)	27%	51%
11	[Ru(bpy)_3_]Cl_2_	DMF (0.1 M)	37%	54%
12	[Ru(bpy)_3_]Cl_2_	DMA/ACN (0.1 M)	24%	58%
13^e^	[Ru(bpy)_3_]Cl_2_	DMF (0.1 M)	34%	56%
14^f^	[Ru(bpy)_3_]Cl_2_	DMF (0.1 M)	41%	56%
15	[Ru(bpy)_3_]Cl_2_	DMA/ACN (0.05 M)	14%	67%
16^g^	[Ru(bpy)_3_]Cl_2_	DMA/ACN (0.05 M)	21%	64%
17^b,g^	[Ru(bpy)_3_]Cl_2_	DMA/ACN (0.05 M)	12%	73% (64%)
18^g^	[Ru(bpy)_3_](PF_6_)_2_	DMA/ACN (0.05 M)	33%	57%
19^g^	[Ir(dtbbpy)(ppy)_2_]PF_6_	DMA/ACN (0.05 M)	18%	69%
20^g^	eosin Y	DMA/ACN (0.05 M)	77%	12%
21^g,h^	[Ru(bpy)_3_]Cl_2_	DMA/ACN (0.05 M)	19%	33%
22^g,i^	[Ru(bpy)_3_]Cl_2_	DMA/ACN (0.05 M)	15%	67%
23^g,j^	[Ru(bpy)_3_]Cl_2_	DMA/ACN (0.05 M)	27%	32%
24^b^	–	DMA/ACN (0.05 M)	73%	18%
25^b,g,k^	[Ru(bpy)_3_]Cl_2_	DMA/ACN (0.05 M)	91%	9%

^a^Yields refer to calibrated GC yields. Numbers in parentheses are isolated yields. If not otherwise noted, 5 mol % catalyst and a 6 W LED were used; ^b^18 W LED; ^c^degassed solvent; ^d^ambient atmosphere; ^e^**1a**/**2a** 1.0:1.3; ^f^**1a**/**2a** 1.3:1.0; ^g^2 mol % catalyst; ^h^K_2_CO_3_ (1.5 equiv) was added; ^i^lutidine (1.5 equiv) was added; ^j^DIPEA (1.5 equiv) was added; ^k^no light.

**Figure 1:**
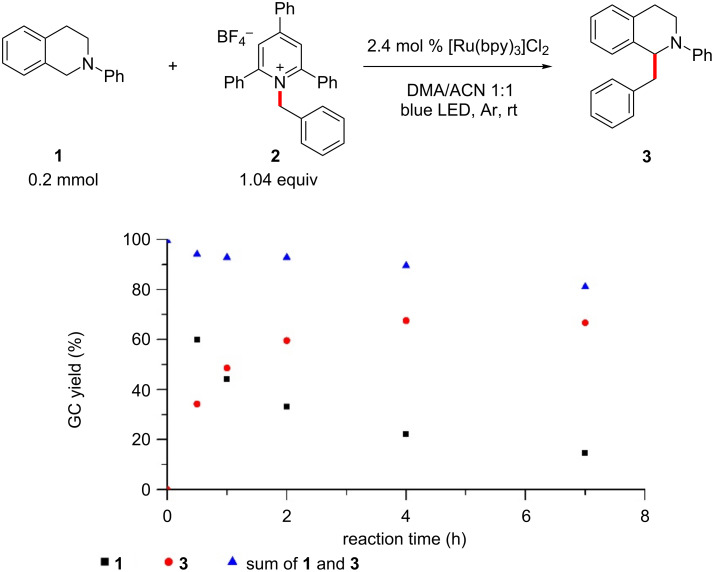
Kinetic profile for the benzylation of **1** to **3**.

With the optimized reaction conditions in hand, we looked at the kinetic profile of the transformation in order to determine the ideal reaction time. The kinetic profile clearly showed that the product formation stopped after approximately 5–6 hours, with further reaction time only resulting in lower mass balance, suggesting possible decomposition of the reaction components (Figure 1).

Then, the substrate scope of the transformation was investigated, reacting different benzylic pyridinium salts with *N*-phenyl-THIQ (**1**, Scheme 2). Initially, steric effects were investigated using *ortho*, *meta*, and *para*-methylated benzylpyridinium salts. The *para*-substituted product **6** was isolated in 64% yield, and the two sterically more congested products **4** and **5** gave about 10% lower yields (Scheme 2). This went in line with the similarly sterically demanding 2-methylnaphthyl product **7**, which was obtained in 50% yield. Halides, such as bromo and fluoro substituents were also well tolerated. Especially the *para*-bromo compound **8** was interesting, since the bromine provides a handle for further functionalization. The electron-withdrawing CF_3_ group gave a good yield of **10** (61%), whereas the electron-donating methoxy group led to a decrease in yield (**11**, 44%). The α- and β-picolinylpyridinium salts gave significantly lower yields for **12** and **13** in comparison. In four examples, **3**, **9**, **10**, and **11**, the effect of longer reaction times was also investigated, and experiments with 18 h reaction time were carried out. It was found that the yields were within the experimental error for three of the examples, while for compound **10**, a longer reaction time gave a significantly lower yield (50% vs 61%).

**Scheme 3:**
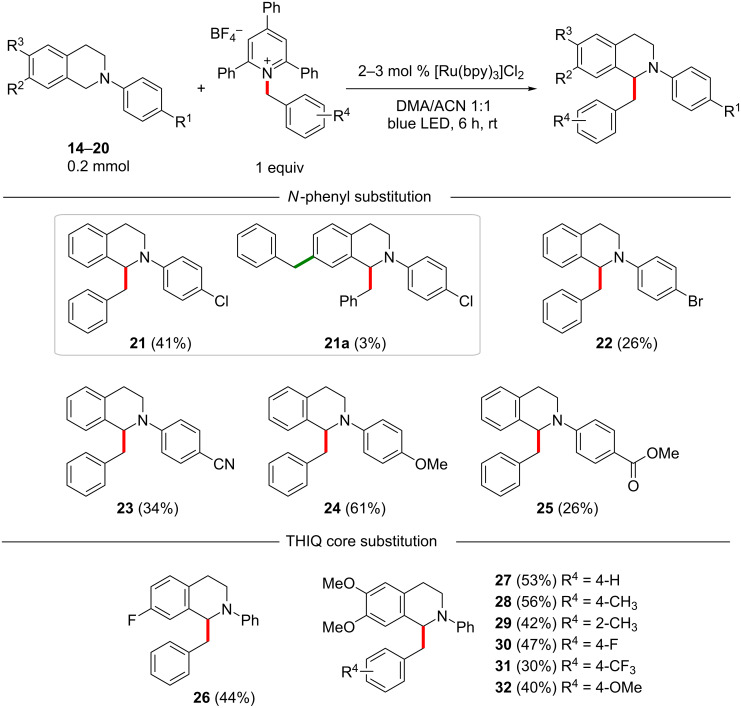
Benzylation of substituted *N*-arylTHIQs.

Next, differently substituted THIQs were tested with (substituted) benzylpyridinium salts as reaction partners (Scheme 3). Initially, different substituents on the *N*-phenyl group were investigated. Instead of the *N*-phenyl group, other protecting groups such as acetyl, benzoyl, or carbamates were tested, but the corresponding starting materials proved to be unreactive in the desired transformation. The use of substituted *N*-phenyl groups revealed that almost all of the substituents had a negative effect on the efficiency of the transformation, independently of their electronic effects (Scheme 3, **21**–**25**). Still, halides, nitriles, and ester substituents were tolerated, giving a possible handle for further manipulation. However, the presence of a nitro group impeded the reaction completely (not shown). Notably, PMP-protected THIQ was found to give a good yield of 61% (compound **24**), which was specifically important since this group is relatively easily removed, contrarily to the unsubstituted phenyl group. The benzylation of THIQ **14** was accompanied by small amounts (3%) of the bisbenzylated side product **21a**. According to NMR analysis (COSY, HSQC and HMBC, see Supporting Information File 1), this second benzyl group was attached to position 7 of the THIQ scaffold.

Additionally, the effect of different substituents on the THIQ core was investigated. A fluoro substituent in position 7 (in **19**) gave **26** in 44% yield after 6 h (Scheme 3). A more relevant substrate was the 6,7-dimethoxy-THIQ (**20**), since this structural motif occurs in many natural products. The yields were slightly lower as compared to the transformations on substrate **1**. The unsubstituted benzyl residue was incorporated to give **27** in 53% yield (vs 58% in 6 h for **3**). Again, a *p-*methylbenzyl substituent led to higher yields as compared to an *o-*methylbenzyl group (cf. Scheme 3, compounds **28** and **29**). Also, the *p-*F and *p-*CF_3_-substituted products **30** and **31** were obtained in 47% and 30% yields, respectively. Interestingly, the *p-*OMe-substituted product **32** was obtained in only slightly lower yield as compared to the corresponding unsubstituted THIQ product **11**.

**Scheme 4:**
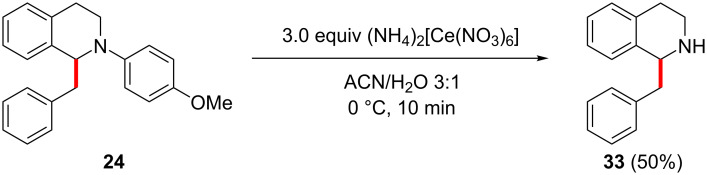
Removal of the PMP protecting group.

For better application in the synthesis of bioactive compounds or natural products, the removal of the aryl moiety on the nitrogen is necessary as most of these compounds are either free amines or N-alkylated [48–49]. Hence, we tested the oxidative cleavage of the PMP group of compound **24** (Scheme 4), with ceric ammonium nitrate (CAN) [50–51]. After column chromatography, 50% of the desired free amine were isolated, which made this method a viable route towards desired bioactive compounds.

**Scheme 5:**
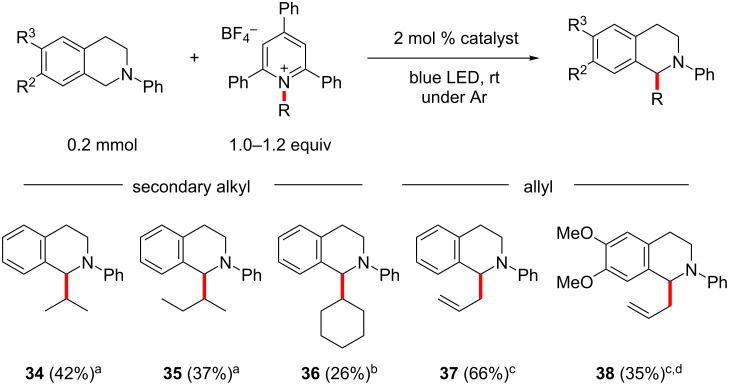
Alkylation of *N*-phenyl-THIQ derivatives. Conditions: ^a^2 mol % [Ir(dtbbpy)(ppy)_2_]PF_6_, DMA, 60 h; ^b^2 mol % [Ir(dtbbpy)(ppy)_2_]PF_6_, DMA, 48 h; ^c^2 mol % [Ru(bpy)_3_]Cl_2_, DMA/ACN 1:1, 6 h; ^d^2 mol % [Ru(bpy)_3_]Cl_2_, DMA/ACN 1:1, 3 h.

At last, we turned our interest towards non-benzylic Katritzky salts, showcasing that also the reaction with unactivated secondary alkyl and allyl radicals takes place (Scheme 5). For the less reactive secondary alkyls the more expensive catalyst [Ir(dtbbpy)(ppy)_2_]PF_6_ had to be employed instead of [Ru(bpy)_3_]Cl_2_ [45,47]. Considerably lower conversion rates were observed in comparison to the benzylic radicals, which translated to longer reaction times for all the substrates tested. For the isoproplyation and *sec*-butylation, moderate yields of about 40% were observed after 60 h reaction time (**34** and **35**). For the cyclohexylation the yield was even lower and conversion stopped after 48 h (26%, **36**). When [Ru(bpy)_3_]Cl_2_ was used as the catalyst, GC–MS analysis of the crude material showed considerably less conversion in comparison. We found that Katritzky salts deriving from amino acids gave a complex reaction mixture, and the desired products could not be isolated. Additionally, alkyl salts derived from primary amines were unreactive.

For the more stable allyl radicals, our initially optimized conditions with [Ru(bpy)_3_]Cl_2_ in DMA/ACN were again applicable and the reaction time was kept at 6 h, or was even shortened to 3 h for 6,7-dimethoxy-*N*-phenyl-THIQ (products **37** and **38**). Allylation of the unsubstituted phenyl-THIQ was again more efficient compared to the dimethoxylated substrate.

**Scheme 6:**
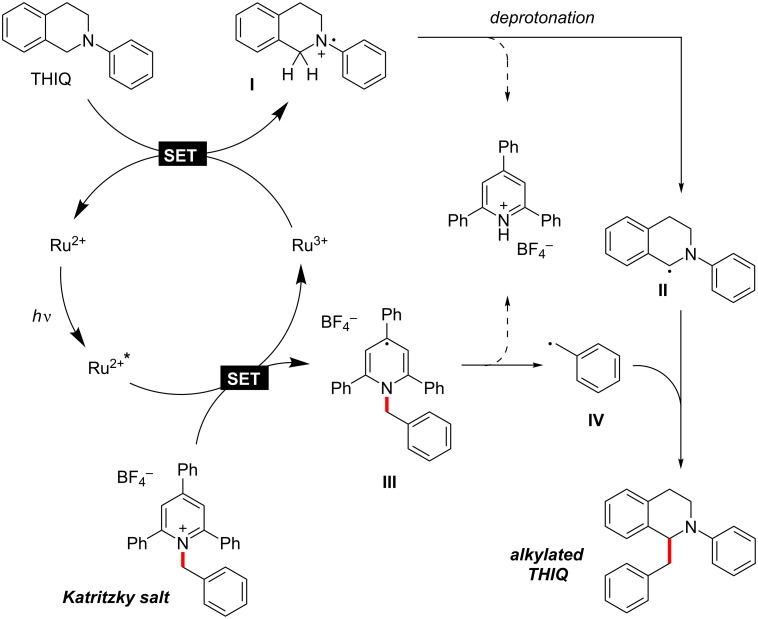
Proposed mechanism.

Based on previous reports [45,47,52], we propose the following mechanism for the benzylation of *N*-aryl-THIQs [47]: After excitation of the catalyst, the Katritzky salt is reduced via SET, giving a Ru(III) species and intermediate **III**, followed by C–N cleavage to give benzylic radical **IV** and triphenylpyridine [45]. The Ru(II) catalyst is regenerated by another SET from the THIQ derivative. The now positively charged THIQ radical **I** is deprotonated to give neutral radical **II** [52]. Combination of **II** and **IV** finally furnishes the benzylated (alkylated) THIQ derivative (Scheme 6). The product formation by radical–radical coupling might explain why high yields were difficult to achieve.

## Conclusion

In summary, we developed a photoredox-catalyzed C(sp³)–H alkylation of *N*-aryltetrahydroisoquinolines under mild conditions. Easily accessible and bench-stable pyridinium salts were used as precursors for the required alkyl radicals. The transformation gives good to moderate yields using benzyl and allyl pyridinium salts. The introduction of secondary alkyl chains was also proved possible. Additionally, the crucial removal of a PMP protecting group to furnish unprotected THIQ was demonstrated, which enables this method to be used for the synthesis of natural products and bioactive substances. Further investigations in this direction are ongoing in our labaratories.

## Supporting Information

File 1 Experimental procedures, characterization data and copies of spectra.

## References

[1] Yi H, Zhang G, Wang H, Huang Z, Wang J, Singh A K, Lei A (2017). Chem Rev.

[2] Liu C, Yuan J, Gao M, Tang S, Li W, Shi R, Lei A (2015). Chem Rev.

[3] Hashiguchi B G, Bischof S M, Konnick M M, Periana R A (2012). Acc Chem Res.

[4] Chen Z, Wang B, Zhang J, Yu W, Liu Z, Zhang Y (2015). Org Chem Front.

[5] Sambiagio C, Schönbauer D, Blieck R, Dao-Huy T, Pototschnig G, Schaaf P, Wiesinger T, Zia M F, Wencel-Delord J, Besset T (2018). Chem Soc Rev.

[6] Hartwig J F, Larsen M A (2016). ACS Cent Sci.

[7] Davies H M L, Morton D (2016). J Org Chem.

[8] Li Z, Li C-J (2005). J Am Chem Soc.

[9] Bentley K W (2005). Nat Prod Rep.

[10] Scott J D, Williams R M (2002). Chem Rev.

[11] Le V H, Inai M, Williams R M, Kan T (2015). Nat Prod Rep.

[12] Li Z, Bohle D S, Li C-J (2006). Proc Natl Acad Sci U S A.

[13] Boess E, Schmitz C, Klussmann M (2012). J Am Chem Soc.

[14] Baslé O, Li C-J (2008). Org Lett.

[15] Li Z, Li C-J (2005). J Am Chem Soc.

[16] Zhang W, Yang S, Shen Z (2016). Adv Synth Catal.

[17] Li Z, Li C-J (2004). Org Lett.

[18] Huang T, Liu X, Lang J, Xu J, Lin L, Feng X (2017). ACS Catal.

[19] Yan C, Liu Y, Wang Q (2015). Org Lett.

[20] Sambiagio C, Noël T (2020). Trends Chem.

[21] McAtee R C, McClain E J, Stephenson C R J (2019). Trends Chem.

[22] Marzo L, Pagire S K, Reiser O, König B (2018). Angew Chem, Int Ed.

[23] Condie A G, González-Gómez J C, Stephenson C R J (2010). J Am Chem Soc.

[24] Freeman D B, Furst L, Condie A G, Stephenson C R J (2012). Org Lett.

[25] Nakajima K, Miyake Y, Nishibayashi Y (2016). Acc Chem Res.

[26] Hou H, Zhu S, Atodiresei I, Rueping M (2018). Eur J Org Chem.

[27] Graulich A, Scuvée-Moreau J, Alleva L, Lamy C, Waroux O, Seutin V, Liégeois J-F (2006). J Med Chem.

[28] Schönbauer D, Spettel M, Pollice R, Pittenauer E, Schnürch M (2019). Org Biomol Chem.

[29] Spettel M, Pollice R, Schnürch M (2017). Org Lett.

[30] Bapat J B, Blade R J, Boulton A J, Epsztajn J, Katritzky A R, Lewis J, Molina-Buendia P, Nie P-L, Ramsden C A (1976). Tetrahedron Lett.

[31] Sowmiah S, Esperança J M S S, Rebelo L P N, Afonso C A M (2018). Org Chem Front.

[32] He F-S, Ye S, Wu J (2019). ACS Catal.

[33] Correia J T M, Fernandes V A, Matsuo B T, Delgado J A C, de Souza W C, Paixão M W (2020). Chem Commun.

[34] Pang Y, Moser D, Cornella J (2020). Synthesis.

[35] Liao J, Guan W, Boscoe B P, Tucker J W, Tomlin J W, Garnsey M R, Watson M P (2018). Org Lett.

[36] Martin-Montero R, Yatham V R, Yin H, Davies J, Martin R (2019). Org Lett.

[37] Yue H, Zhu C, Shen L, Geng Q, Hock K J, Yuan T, Cavallo L, Rueping M (2019). Chem Sci.

[38] Ni S, Li C-X, Mao Y, Han J, Wang Y, Yan H, Pan Y (2019). Sci Adv.

[39] Lima C G S, de M. Lima T, Duarte M, Jurberg I D, Paixão M W (2016). ACS Catal.

[40] Wu J, He L, Noble A, Aggarwal V K (2018). J Am Chem Soc.

[41] Wu J, Grant P S, Li X, Noble A, Aggarwal V K (2019). Angew Chem, Int Ed.

[42] Sandfort F, Strieth-Kalthoff F, Klauck F J R, James M J, Glorius F (2018). Chem – Eur J.

[43] James M J, Strieth‐Kalthoff F, Sandfort F, Klauck F J R, Wagener F, Glorius F (2019). Chem – Eur J.

[44] Yang M, Cao T, Xu T, Liao S (2019). Org Lett.

[45] Klauck F J R, James M J, Glorius F (2017). Angew Chem, Int Ed.

[46] Ociepa M, Turkowska J, Gryko D (2018). ACS Catal.

[47] Xu Y, Xu Z-J, Liu Z-P, Lou H (2019). Org Chem Front.

[48] Menachery M D, Lavanier G L, Wetherly M L, Guinaudeau H, Shamma M (1986). J Nat Prod.

[49] Jangir R, Argade N P (2017). Synthesis.

[50] Wuts P G M, Greene T W (2006). Protection for the Amino Group. Greene's Protective Groups in Organic Synthesis.

[51] Wang T, Schrempp M, Berndhäuser A, Schiemann O, Menche D (2015). Org Lett.

[52] Bartling H, Eisenhofer A, König B, Gschwind R M (2016). J Am Chem Soc.

