# Supplementary material for: Photocatalytic deaminative benzylation and alkylation of tetrahydroisoquinolines with N-alkylpyrydinium salts
Source: Beilstein J Org Chem. 2020 Apr 21;16:809–17. doi: 10.3762/bjoc.16.74 (PMC7189001; doi:10.3762/bjoc.16.74)
Supplement: File 1 — Experimental procedures, characterization data and copies of spectra. [file Beilstein_J_Org_Chem-16-809-s001.pdf]

BEILSTEIN JOURNAL OF ORGANIC CHEMISTRY

Supporting Information

for

Photocatalytic deaminative benzylation and alkylation of tetrahydroisoquinolines with *N*-alkylpyrydinium salts

David Schönbauer, Carlo Sambiagio, Timothy Noël and Michael Schnürch

Beilstein J. Org. Chem. **2020,** *16,* 809–817. doi:10.3762/bjoc.16.74

Experimental procedures, characterization data and copies of spectra

License and Terms: This is a supporting information file under the terms of the Creative Commons Attribution License (http://creativecommons.org/licenses/by/4.0). Please note that the reuse, redistribution and reproduction in particular requires that the authors and source are credited.
The license is subject to the *Beilstein Journal of Organic Chemistry* terms and conditions: (https://www.beilstein-journals.org/bjoc)

Table of contents

1. Abbreviations S1
2. Materials and methods S2
3. Reaction optimization S4
   3.1. Calibration S4
   3.2. Sample preparation S4
   3.3. Screening results S4
   3.4. Unreactive substrates S5
4. Photochemical benzylation/alkylation of THIQs S5
   4.1. General procedure for photoredox reactions (GP1) S5
   4.2. Analytical data of products S7
5. *N*-Dearylation procedure S21
6. Synthesis of THIQs S22
   6.1. General procedures for arylation of THIQ (GP2 and GP3) S22
   6.2. Analytical data of substrates S22
   6.3. Synthesis and analytical data of THIQ derivatives S3 and S4 .... S26
7. Synthesis of *N*-pyridinium salts (GP4) S28
   7.1. Analytical data of pyridinium salts S28
8. 2,4,6-Triphenylpyrylium tetrafluoroborate (S16) S34
9. References S34
10. Spectra S35

1. Abbreviations

$[Ru(bpy)_3]Cl_2$	Tris(2,2′-bipyridyl)dichlororuthenium(II) hexahydrate
EA	ethyl acetate
ACN	acetonitrile
DMSO	dimethylsulfoxide
DMA	dimethylacetamide
DCE	dichloroethane
DCM	dichloromethane
DMF	dimethylformamide
KOtBu	potassium *tert* butoxide
LP	light pertrol
MTBE	*tert*-butyl methyl ether
THIQ	1,2,3,4-tetrahydroisoquinoline
TEA	triethylamine
CAN	cerium(IV)-ammonium nitrate

2. Materials and Methods

The following general procedures were used in all reactions, unless otherwise noted. All glassware was flame-dried under argon and reactions were either set up using Schlenk technique with a slight overpressure of Argon, or the vials were transferred into a glove box, if not otherwise noted. All reactions were magnetically stirred and heated either in an oil bath, a metallic reaction block, or were performed at room temperature.

Unless otherwise specified, reagents were purchased from commercial suppliers and used without further purification. Dry solvents used were obtained as below:

- DCM, toluene, and THF were retrieved from an Innovative Technologies PureSolv system and used directly.
- Methanol was retrieved from an Innovative Technologies PureSolv system and stored over molecular sieves.
- ACN, DMA, DMF, and DMSO were purchased as water-free over molecular sieves and degassed prior to use.

All other solvents used were p.a. or HPLC grade.

TLC analysis was done with precoated aluminium-backed plates (silica gel 60 F_{254}, Merck or aluminium Oxide 60 F_{254}, neutral, Merck). Compounds were marked under UV light or visualized by submerging in staining solutions as stated.

Flash column chromatography was mostly performed on a Büchi Sepacore Flash System (2 x Büchi Pump Module C-605, Büchi Pump Manager C-615, Büchi UV Photometer C-635, Büchi Fraction Collector C-660) using a 9 g column (15 mL/min) or 45 g columns (flow 40 mL/min, Büchi). For all other separations silica gel from Merck (40–63 µm) was used.

HPLC preparative chromatography was carried out with an autopurification system of Waters using an ACQUITY QDA MS-detector in combination with a 2998 photodiode array detector. Analytical separation was made using XSELECT CSH fluorophenyl 5 µm, 4.6 × 150 mm and XSELECT CSH C18, 5 µm, 4.6 × 150 mm columns. Preparative separation was made using XSELECT CSH Prep fluorophenyl 5 µm, 30 × 150 mm and XSELECT CSH Prep C18, 5 µm OBD, 30 × 150 mm columns. HPLC grade methanol and HPLC grade water containing 0.1% formic acid were used as solvents.

LC–MS analysis was carried out on a Shimadzu Nexera X2 UHPLC system comprised of LC-30AD pumps, a SIL-30AC autosampler, a CTO-20AC column oven, and a DGU-$20A_{5/3}$ degasser module. Detection was accomplished by concerted efforts of SPD-M20A photodiode array, a RF-20Axs fluorescence detector, an ELS-2041 evaporative light scattering detector (JASCO®) and finally via a LCMS-2020 mass spectrometer. If not stated otherwise, all separations were performed using a Waters® XSelect® CSH™ C18, 2.5 µm (3.0 × 50 mm) column XP at 40 °C, and a flow rate of 1.7 mL/min. Mobile phases used were UHPLC grade water and acetonitrile containing 0.1% of formic acid.

Melting points of crystalline compounds were determined with a Kofler-type Leica Galen III micro hot stage microscope and are uncorrected (Aigner-Unilab Laborfachhandel GmbH).

NMR spectra were recorded in deuterated solvents on a Bruker AC 200 (200 MHz), a Bruker Avance UltraShield 400 (400 MHz), or an Avance III HD 600 (600 MHz) spectrometer, and chemical shifts (δ) are reported in ppm and are referenced to the solvent peak. For $CDCl_3$, proton NMR spectra were referenced to 7.26 ppm and carbon NMR spectra to 77.16 ppm; spectra measured in DMSO-d_6 were referenced to 2.50

ppm for proton, and 39.52 ppm for carbon spectra. ^{19}F NMR spectra were recorded without internal standard and were locked to the deuterated solvent used for the measurement. Coupling constants (*J*) are given in hertz (Hz). Multiplicities of the signals are abbreviated as follows: s = singlet, d = doublet, t = triplet, q = quartet, m = multiplet, dd = doublet of doublet, dt = doublet of triplet, td = triplet of doublet, ddd = doublet of doublet of doublet and bs = broad singlet. Carbon NMR were recorded either as APT, DEPTQC or standard decoupled C13 spectra.

GC–MS runs were performed on a Thermo Finnigan Focus GC/DSQ II with a standard capillary RXi-5Sil MS column (30 m, 0.25 mmID, 0.25 µm df, Restek) using standardized temperature programs: "STD10min" (2 min at 100 °C, 35 °C/min until 300 °C, 2 min at 300 °C), "STD12min" (2 min at 100 °C, 35 °C/min until 300 °C, 4 min at 300 °C), "STD14min" (2 min at 100 °C, 35 °C/min until 300 °C, 6 min at 300 °C) or “STD100” (2 min at 100 °C, 18 °C/min until 280 °C, 3 min at 280 °C) or with a GC–MS hyphenation from Thermo Finnigan: focus GC with a BGB5 column (l = 30 m, diameter = 0.25 mm, 0.25 mm film); DSQ II Quadrupole (EI+ mode) running the following temperature program: “STD17” (2 min at 100 °C, 20 °C/min until 300 °C, 5 min at 300 °C)

GC–FID analysis was performed on a Trace Dual GC, equipped with a TR-5MS column (length 15 m, inner diameter 0.25 mm, film thickness 1.0 µm) and an FID detector running the following temperature program: 1.0 min at 100 °C, 60 °C/min until 300°C, 6.70 min at 300 °C). Other analyses were performed on a Shimadzu GC-2010 Plus with AOC-20i Autosampler running the following temperature program: 1 min at 50° C, 13 °C/min until 125 °C for 1 min, 25 °C/min until 250 °C for 6.23 min and 50 °C/min to 270 °C for 3.10 min.

For the removal of high-boiling solvents, a vacuum concentrator RVC 2-25 CDPLUS with an Alpha 2-4 LDplus freeze-dryer was used.

HR–MS analysis was carried out from methanol solutions by using an HTC PAL system autosampler, an Agilent 1100/1200 HPLC with binary pumps, degasser, and column thermostat and Agilent 6230 AJS ESI–TOF mass spectrometer.

3. Reaction optimization

3.1. Calibration

All conversion data were calculated from peak areas of calibrated GC–FID measurements after automatic uniform integration. For GC calibrations, EA was used as solvent. Volumes were measured with Kolbenhub pipettes. Each analyte was calibrated with concentrations of 0.40 mM to 5.00 mM using dodecane as the internal standard. All compounds were found to have a linear response over the whole concentration range.

3.2. Sample preparation

For analysis, 10 µL dodecane were added to the reaction mixture after the reaction and the solution was filtered through a plug of silica using EA as eluent (in total 2.5 mL solvent were used, including the reaction mixture). Then, a 50 µL sample was taken and filled up with EA to 1.0 mL total volume, to set the concentrations to 1 mM for dodecane and 5 mM for the analytes. All samples were filtered through PALL Acrodisc CR 13mm syringe filters with 0.2 µm PTFE membrane prior to GC analysis.

3.3. Screening results

Table S1: Optimization of the reaction conditions.

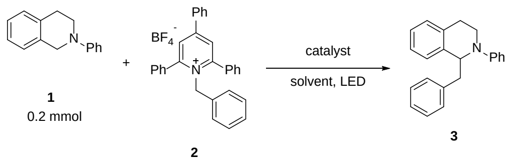

entry	cat. [5 mol %]	solvent	light	time [h]	**1a**	**3a**
1	$[Ru(bpy)_3]Cl_2$	ACN (0.1 M)	blue, 18 W	18	14%	29%
2	Eosin Y	ACN (0.1 M)	blue, 18 W	18	36%	21%
3	Fluorecein	ACN (0.1 M)	blue, 18 W	18	74%	13%
4	Ir(ppy)2(dtbbpy)PF6	ACN (0.1 M)	blue, 6 W	18	9%	21%
5[a]	$[Ru(bpy)_3]Cl_2$	ACN (0.1 M)	blue, 6 W	18	54%	30%
6[b]	$[Ru(bpy)_3]Cl_2$	ACN (0.1 M)	blue, 6 W	18	6%	33%
7	$[Ru(bpy)_3]Cl_2$	DMSO (0.1 M)	blue, 6 W	18	27%	55%
8	$[Ru(bpy)_3]Cl_2$	DMA (0.1 M)	blue, 6 W	18	27%	51%
9	$[Ru(bpy)_3]Cl_2$	DCE (0.1 M)	blue, 6 W	18	63%	22%
10	$[Ru(bpy)_3]Cl_2$	DCM (0.1 M)	blue, 6 W	18	55%	25%
11	$[Ru(bpy)_3]Cl_2$	DMF (0.1 M)	blue, 6 W	18	37%	54%
12	$[Ru(bpy)_3]Cl_2$	DMF (0.1 M)	blue, 6 W	40	17%	55%
13	$[Ru(bpy)_3]Cl_2$	DMF (0.1 M)	blue, 4.5 W	46	3%	55%
14[c]	$[Ru(bpy)_3]Cl_2$	DMF (0.1 M)	blue, 6 W	18	34%	56%
15[d]	$[Ru(bpy)_3]Cl_2$	DMF (0.1 M)	blue, 6 W	18	41%	56%
16	$[Ru(bpy)_3]Cl_2$	DMF (0.2 M)	blue, 6 W	18	20%	51%

17	$[Ru(bpy)_3]Cl_2$	DMF (0.05 M)	blue, 6 W	18	22%	59%
18	$[Ru(bpy)_3]Cl_2$	DMF/ACN (0.1 M)	blue, 6 W	18	34%	45%
19	$[Ru(bpy)_3]Cl_2$	DMF/DMSO (0.1 M)	blue, 6 W	18	26%	57%
20	$[Ru(bpy)_3]Cl_2$	DMA/ACN (0.1 M)	blue, 6 W	18	24%	58%
21	$[Ru(bpy)_3]Cl_2$	DMSO/ACN (0.1 M)	blue, 6 W	18	15%	44%
22	$[Ru(bpy)_3]Cl_2$	DMA/ACN (0.05 M)	blue, 6 W	18	14%	67%
23	$[Ru(bpy)_3]Cl_2$	DMA/ACN (0.025 M)	blue, 6 W	18	9%	65%
24	$[Ru(bpy)_3]Cl_2$	DMA/ACN 2/1 (0.05 M)	blue, 6 W	18	21%	66%
25	$[Ru(bpy)_3]Cl_2$	DMA/ACN 1/2 (0.05 M)	blue, 6 W	18	26%	61%
26	$[Ru(bpy)_3]Cl_2$ (2 mol %)	DMA/ACN (0.05 M)	blue, 6 W	18	21%	64%
27	$Ru(bpy)_3(PF_6)_2$ (2 mol %)	DMA/ACN (0.05 M)	blue, 6 W	18	33%	57%
28	Eosin Y (2 mol %)	DMA/ACN (0.05 M)	blue, 6 W	18	77%	12%
29	$Ir(ppy)_2(dtbbpy)PF_6$ (2 mol %)	DMA/ACN (0.05 M)	blue, 6 W	18	18%	69%
30[e]	$[Ru(bpy)_3]Cl_2$ (2 mol %)	DMA/ACN (0.05 M)	blue, 6 W	18	19%	33%
31[f]	$[Ru(bpy)_3]Cl_2$ (2 mol %)	DMA/ACN (0.05 M)	blue, 6 W	18	15%	67%
32[g]	$[Ru(bpy)_3]Cl_2$ (2 mol %)	DMA/ACN (0.05 M)	blue, 6 W	18	27%	32%
**33**	**$[Ru(bpy)_3]Cl_2$ (2 mol %)**	**DMA/ACN (0.05 M)**	**blue, 18 W**	**24**	**12%**	**73% (64%)**
34	–	DMA/ACN (0.05 M)	blue, 18 W	18	73%	18%
35	$[Ru(bpy)_3]Cl_2$ (2 mol %)	DMA/ACN (0.05 M)	–	18	96%	9%
36	$[Ru(bpy)_3]Cl_2$ (2 mol %)	DMA/ACN/MeOH 4:4:1 (0.05 M)	blue, 18 W	6	n.d.	(40%)
37	$[Ru(bpy)_3]Cl_2$ (2 mol %)	MeOH (0.05 M)	blue, 18 W	6	n.d.	n.d.

Yields refer to calibrated GC yields. Numbers in parentheses are isolated yields.
[a]solvent degassed, [b]ambient atmosphere, [c]**1a**/**2a** = 1.0:1.3, [d]**1a**/**2a** = 1.3:1.0, [e]K_2CO_3 (1.5 equiv), [f]lutidine (1.5 equiv), [g]DIPEA (1.5 equiv).

3.4. Unreactive substrates

The following substrates were tested in the transformation, but showed no reactivity.

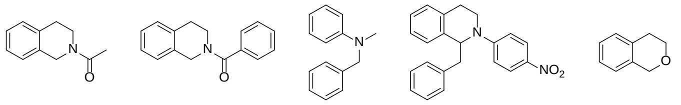

4. Photochemical benzylation/alkylation of THIQs

4.1. General procedure for photoredox reactions (GP1)

Photoreactions were performed in a custom-made photoreactor: 2.5 m (18 W) of a flexible LED strip (36 W, 300 LEDS, 24 V and an intensity maximum of 462 nm; see Figure S1) was wrapped inside a 3D-printed case, and the reaction vials were placed inside as shown in Figure S2 (distance ≈ 1 cm from the light source). A fan or a flow of compressed air was used for cooling (see Figure S2).

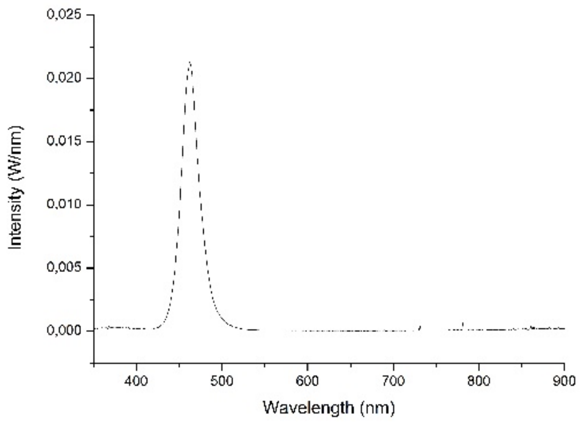

Figure S1 Emission spectrum for blue LED

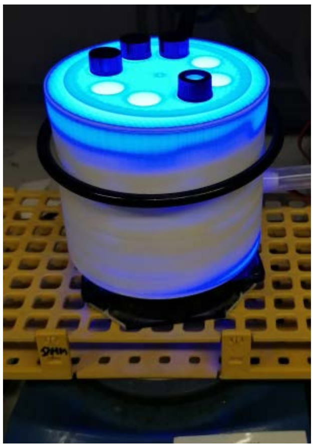

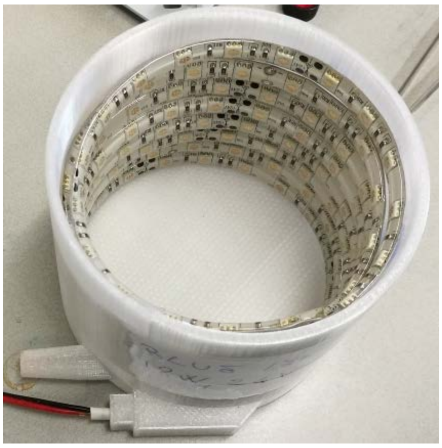

Figure S2 Left: Setup for photoredox reactions; right: top view of the 3D-printed case and light arrangement.

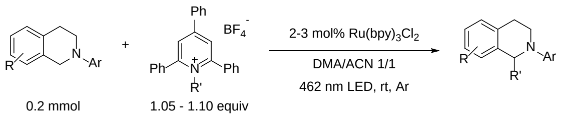

A flame-dried 8 mL vial equipped with a stirring bar was charged with the tetrahydroisoquinoline derivative (0.2 mmol, 1.0 equiv), the 2,4,6-triphenylpyridinium tetrafluoroborate derivative (1.05–1.1 equiv), $[Ru(bpy)_3]Cl_2$ (2–3 mol %), and closed with a septum. Then, the vial was set under argon and charged with DMA/ACN 1:1 (0.05 M solution of amine)*. The solution was then stirred and irradiated for the indicated time with 18 W blue LED. The reaction was stopped, and the reaction mixture was filtered through a small plug of silica, and rinsed with either DCM or EA (depending on the R_f values). Then, the solvents were removed under reduced pressure at 40 °C, and the crude material purified by column chromatography using either hex/EA or hex/DCM as eluents.

* The solvent was either degassed prior by gas bubbling and sonic bath for at least 20 min or three freeze-pump-thaw cycles. If the solvent was not degassed prior, the solution was quickly degassed by three consecutive Schlenk cycles under vigorous stirring. The different degassing methods do not change the result of the reactions.

4.2. Analytical data of products

1-Benzyl-2-phenyl-1,2,3,4-tetrahydroisoquinoline (3)

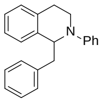

3

According to GP1. A solution of 2-phenyl-1,2,3,4-tetrahydroisoquinoline (80.1 mg, 382.7 µmol, 1.0 equiv), 1-benzyl-2,4,6 triphenylpyridinium tetrafluoroborate (184.6, 380.4 µmol, 1.0 equiv) and 6.3 mg $[Ru(bpy)_3]Cl_2$.(2.2 mol %) in 4 mL DMA/ACN 1:1 was irradiated for 18 h. After workup, the product was isolated as colorless oil in 64% yield (73.0 mg, 243.8 µmol). If the reaction was stopped after 6 h, a yield of 58% was obtained.

Spectroscopic data were in accordance with literature [1].

^{1}H NMR (400 MHz, $CDCl_3$) δ = 7.28 – 7.10 (m, 7H), 7.09 – 6.99 (m, 3H), 6.84 (d, *J*=8.1, 2H), 6.80 – 6.69 (m, 2H), 4.90 (t, *J*=6.6, 1H), 3.65 (ddd, *J*=12.5, 7.7, 5.0, 1H), 3.55 (dt, *J*=12.1, 6.0, 1H), 3.26 (dd, *J*=13.4, 5.8, 1H), 3.00 (dt, *J*=14.6, 7.3, 2H), 2.75 (dt, *J*=15.9, 5.8, 1H).

^{13}C NMR (101 MHz, $CDCl_3$) δ = 149.3, 138.9, 137.6, 135.1, 129.8, 129.3, 128.3, 128.2, 127.7, 126.7, 126.3, 125.6, 117.3, 113.8, 61.6, 42.4, 42.3, 27.5.

Rf: 0.5 (LP/DCM = 1/1 – UV)

GC-MS: STD17min; rt = 11.8 min; 115 (10), 108 (100), 91 (22), 77 (16).

1-(2-Methylbenzyl)-2-phenyl-1,2,3,4-tetrahydroisoquinoline (4)

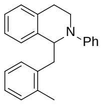

4

According to GP1. A solution of 2-phenyl-1,2,3,4-tetrahydroisoquinoline (40.2 mg, 192.1 µmol, 1.0 equiv), 1-(2-methylbenzyl)-2,4,6-triphenylpyridinium tetrafluoroborate (103.7 mg, 207.7 µmol, 1.1 equiv) and $[Ru(bpy)_3]Cl_2$ (3.3 mg, 2.3 mol %) was irradiated for 6 h. After workup and purification by column chromatography, 52% (31.4 mg, 100.2 µmol) product were isolated as colorless oil.

^{1}H NMR (600 MHz, $CDCl_3$) δ = 7.44 – 7.36 (m, 2H), 7.33 – 7.20 (m, 6H), 7.19 – 7.14 (m, 1H), 6.99 (d, *J*=8.2, 2H), 6.89 (t, *J*=7.2, 1H), 6.82 (d, *J*=7.6, 1H), 5.06 (t, *J*=6.9, 1H), 3.89 (ddd, *J*=12.5, 7.8, 5.0, 1H), 3.75 (dt, *J*=12.1, 5.9, 1H), 3.38 (dd, *J*=13.6, 6.3, 1H), 3.27 (dd, *J*=13.6, 7.5, 1H), 3.20 (ddd, *J*=15.7, 7.7, 5.5, 1H), 2.99 (dt, *J*=15.8, 5.7, 1H), 2.15 (s, 3H).

^{13}C NMR (151 MHz, $CDCl_3$) δ = 149.5, 137.8, 137.4, 137.0, 135.2, 130.7, 130.2, 129.3, 128.3, 127.6, 126.8, 126.5, 126.0, 125.7, 117.4, 113.8, 61.4, 42.2, 39.0, 27.5, 19.5.

Rf: 0.57 (LP/DCM = 1/1 – UV)

GC-MS: STD12min; rt = 8.8 min; 208 (100), 128 (3), 115 (13), 77 (24)

HRMS: calculated for $C_{23}H_{24}N$ $[M+H]^+$ 314.1903; found 314.1907; Δ = 1.20 ppm.

1-(3-Methylbenzyl)-2-phenyl-1,2,3,4-tetrahydroisoquinoline (5)

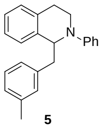

5

According to GP1. A solution of 2-phenyl-1,2,3,4-tetrahydroisoquinoline (40.0 mg, 191.1 µmol, 1.0 equiv), 1-(3-methylbenzyl)-2,4,6-triphenylpyridinium tetrafluoroborate (103.7 mg, 207.7 µmol, 1.1 equiv) and $[Ru(bpy)_3]Cl_2$ (3.2 mg, 2.2 mol %) was irradiated for 6 h. After workup and purification by column chromatography, 55% (32.9 mg, 105.0 µmol) product were isolated as colorless oil, which was purified by preparative HPLC for a pure analytical sample.

^{1}H NMR (600 MHz, $CDCl_3$) δ = 7.42 – 7.33 (m, 2H), 7.30 – 7.20 (m, 3H), 7.16 (t, *J*=6.7, 1H), 7.12 (d, *J*=7.4, 1H), 6.98 (d, *J*=8.2, 2H), 6.95 – 6.91 (m, 2H), 6.89 – 6.80 (m, 2H), 5.01 (t, *J*=6.6, 1H), 3.78 (ddd, *J*=12.4, 7.5, 5.1, 1H), 3.67 (dt, *J*=12.0, 6.0, 1H), 3.35 (dd, *J*=13.4, 5.7, 1H), 3.12 (dt, *J*=16.8, 7.1, 6.0, 1H), 3.07 (dd, *J*=13.4, 7.5, 1H), 2.90 (dt, *J*=15.7, 5.7, 1H), 2.39 (s, 3H).

^{13}C NMR (151 MHz, $CDCl_3$) δ = 149.5, 138.9, 137.8, 137.7, 135.2, 130.7, 129.3, 128.3, 128.1, 127.9, 127.1, 126.9, 126.7, 125.5, 61.6, 42.5, 42.3, 27.6, 21.5.

Rf: 0.47 (LP/DCM = 2/1 – UV)

GC-MS: STD12min; rt = 8.7 min; 208 (100), 128 (3), 115 (12), 103 (7), 77 (22)

HRMS: calculated for $C_{23}H_{24}N$ $[M+H]^+$ 314.1903; found 314.1905; Δ = 0.60 ppm.

1-(4-Methylbenzyl)-2-phenyl-1,2,3,4-tetrahydroisoquinoline (6)

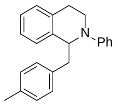

6

According to GP1. A solution of 2-phenyl-1,2,3,4-tetrahydroisoquinoline (40.6 mg, 194.0 µmol, 1.0 equiv), 1-(4-methylbenzyl)-2,4,6-triphenylpyridinium tetrafluoroborate (103.0 mg, 206.3 µmol, 1.1 equiv) and $[Ru(bpy)_3]Cl_2$ (3.6 mg, 2.5 mol %) was irradiated for 6 h. After workup and column chromatography, 64% (32.9 mg, 105.0 µmol) product were isolated as colorless oil, which was purified by preparative HPLC for a pure analytical sample.

Spectroscopic data were in accordance with literature [1].

^{1}H NMR (600 MHz, $CDCl_3$) δ = 7.24 (td, *J*=7.3, 2.0, 2H), 7.17 – 7.11 (m, 2H), 7.05 (dd, *J*=7.3, 1.3, 1H), 7.03 (d, *J*=7.8, 2H), 6.90 (d, *J*=7.9, 2H), 6.85 (d, *J*=8.1, 2H), 6.75 (d, *J*=7.6, 1H), 6.73 (t, *J*=7.2, 1H), 4.99 – 4.75 (m, 1H), 3.64 (ddd, *J*=12.4, 7.6, 5.0, 1H), 3.57 – 3.51 (m, 1H), 3.21 (dd, *J*=13.5, 5.7, 1H), 3.04 – 2.92 (m, 2H), 2.76 (dt, *J*=15.8, 5.8, 1H), 2.31 (s, 3H).

^{13}C NMR (151 MHz, $CDCl_3$) δ = 149.5, 137.9, 135.9, 135.8, 135.2, 129.8, 129.4, 129.0, 128.3, 127.8, 126.7, 125.6, 117.2, 113.7, 61.6, 42.3, 42.1, 27.6, 21.2.

Rf: 0.47 (LP/DCM = 2/1 – UV)

GC-MS: STD14min; rt = 8.8 min; 208 (100), 165 (4), 128 (4), 115 (13), 103 (6), 77 (24)

LC-MS: rt = 2.35; 314 (100, M+H), 208 (67).

1-(Naphthalen-2-ylmethyl)-2-phenyl-1,2,3,4-tetrahydroisoquinoline (7)

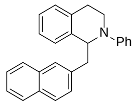

7

According to GP1. A solution of 2-phenyl-1,2,3,4-tetrahydroisoquinoline (40.2 mg, 192.0 µmol, 1.0 equiv), 1-(naphthalen-2-ylmethyl)-2,4,6-triphenylpyridinium tetrafluoroborate (104.0 mg, 194.3 µmol, 1.0 equiv) and $[Ru(bpy)_3]Cl_2$ (3.0 mg, 2.1 mol %) was irradiated for 6 h. After workup and column chromatography, 50% (33.7 mg, 96.4 µmol) product were isolated as colorless oil.

^{1}H NMR (600 MHz, $CDCl_3$) δ = 7.93 (d, *J*=8.4, 1H), 7.90 – 7.85 (m, 1H), 7.74 (d, *J*=8.2, 1H), 7.48 (ddd, *J*=8.1, 6.8, 1.3, 1H), 7.44 (ddd, *J*=8.2, 6.8, 1.5, 1H), 7.32 (dd, *J*=8.1, 7.0, 1H), 7.20 (td, *J*=7.2, 2.0, 2H), 7.16 – 7.10 (m, 3H), 7.01 – 6.95 (m, 1H), 6.81 (d, *J*=8.2, 2H), 6.74 (t, *J*=7.2, 1H), 6.65 (d, *J*=7.6, 1H), 5.13 (t, *J*=6.7, 1H), 3.78 (ddd, *J*=13.4, 8.8, 4.9, 1H), 3.69 – 3.62 (m, 2H), 3.59 (dd, *J*=13.8, 7.1, 1H), 3.06 (ddd, *J*=14.8, 8.7, 5.7, 1H), 2.79 (dt, *J*=15.9, 4.9, 1H).

^{13}C NMR (151 MHz, $CDCl_3$) δ = 149.6, 137.9, 135.0, 134.8, 133.8, 132.4, 129.2, 128.8, 128.5, 128.4, 127.6, 127.1, 126.7, 125.8, 125.5, 125.4, 123.7, 117.6, 114.5, 60.8, 41.7, 38.9, 27.2.

Rf: 0.54 (LP/DCM = 1/1 – UV)

GC-MS: STD14min; rt = 10.8 min; 208 (100), 141 (8), 115 (22), 91 (4), 77 (19)

HRMS: calculated for $C_{26}H_{24}N$ $[M+H]^+$ 350.1903; found 314.1907; Δ = 0.99 ppm.

1-(4-Bromobenzyl)-2-phenyl-1,2,3,4-tetrahydroisoquinoline (8)

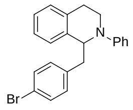

8

According to GP1. A solution of 2-phenyl-1,2,3,4-tetrahydroisoquinoline (40.4 mg, 193.0 µmol, 1.0 equiv), 1-(4-methoxybenzyl)-2,4,6-triphenylpyridinium tetrafluoroborate (115.0 mg, 203.8 µmol, 1.1 equiv) and $[Ru(bpy)_3]Cl_2$ (3.2 mg, 2.2 mol %) was irradiated for 6 h. After workup and purification by column chromatography, 57% (41.9 mg, 110.8 µmol) product were isolated as colorless oil, which was purified for an analytical sample by preparative HPLC.

^{1}H NMR (600 MHz, $CDCl_3$) δ = 7.27 (d, *J*=8.2, 2H), 7.22 – 7.15 (m, 2H), 7.12 – 7.08 (m, 1H), 7.07 (d, *J*=7.2, 1H), 7.01 (t, *J*=7.2, 1H), 6.81 (d, *J*=8.2, 2H), 6.78 (d, *J*=8.2, 2H), 6.72 (d, *J*=7.6, 1H), 6.69 (t, *J*=7.2, 1H), 4.82 (t, *J*=6.4, 1H), 3.64 – 3.50 (m, 1H), 3.47 (dt, *J*=12.0, 5.9, 1H), 3.12 (dd, *J*=13.5, 5.8, 1H), 2.96 – 2.83 (m, 2H), 2.64 (dt, *J*=15.8, 5.7, 1H).

^{13}C NMR (151 MHz, $CDCl_3$) δ = 149.4, 137.9, 137.4, 135.2, 131.6, 131.3, 129.4, 128.5, 127.7, 126.9, 125.8, 120.3, 117.6, 113.9, 61.3, 42.3, 41.9, 27.6.

Rf: 0.75 (LP/DCM = 1/1 – UV, after longer exposition to UV light, the product stains)

GC-MS: STD14min; rt = 9.4 min; 208 (100), 165 (4), 115 (12), 77 (19)

HRMS: calculated for $C_{22}H_{21}BrN$ $[M+H]^+$ 378.0852; found 378.0855; Δ = 0.82 ppm.

1-(4-Fluorobenzyl)-2-phenyl-1,2,3,4-tetrahydroisoquinoline (9)

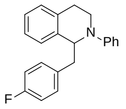

9

According to GP1. A solution of 2-phenyl-1,2,3,4-tetrahydroisoquinoline (40.0 mg, 191.1 µmol, 1.0 equiv), 1-(4-fluorobenzyl)-2,4,6 triphenylpyridinium tetrafluoroborate (105.2 mg, 209.0 µmol, 1.1 equiv) and 3.1 mg $[Ru(bpy)_3]Cl_2$.(2.2 mol %) in 2 mL DMA/ACN 1:1 was irradiated for 6 h. After workup, the product was isolated as colorless oil in 62% yield (37.9 mg, 119.4 µmol). For an analytical sample, the material was purified by HPLC preparative chromatography.

Spectroscopic data were in accordance with literature [1].

^{1}H NMR (600 MHz, $CDCl_3$) δ = 7.27 – 7.22 (m, 2H), 7.17 (td, *J*=7.4, 1.1, 1H), 7.14 (d, *J*=6.7, 1H), 7.08 (td, *J*=7.6, 1.5, 1H), 6.96 (ddd, *J*=8.2, 5.3, 2.4, 2H), 6.94 – 6.88 (m, 2H), 6.84 (d, *J*=8.3, 2H), 6.78 (d, *J*=7.6, 1H), 6.75 (t, *J*=7.2, 1H), 4.88 (t, *J*=6.5, 1H), 3.63 (ddd, *J*=12.4, 7.7, 5.0, 1H), 3.54 (ddd, *J*=12.1, 6.5, 5.5, 1H), 3.22 (dd, *J*=13.6, 5.9, 1H), 3.06 – 2.93 (m, 2H), 2.72 (dt, *J*=15.8, 5.7, 1H).

^{13}C NMR (151 MHz, $CDCl_3$) δ = 161.8 (d, *J*=244.1), 149.4, 137.5, 135.3, 134.6 (d, *J*=3.1), 131.3 (d, *J*=7.8), 129.4, 128.5, 127.7, 126.8, 125.8, 117.5, 115.0 (d, *J*=21.0), 113.8, 61.5, 42.3, 41.7, 27.6.

^{19}F NMR (565 MHz, $CDCl_3$) δ = -116.9.

Rf: 0.58 (LP/DCM = 1/1 – UV and $KMnO_4$)

GC-MS: STD12min; rt = 8.5 min; 208 (100), 115 (10), .77 (15).

LC-MS: rt = 2.3; 318 ($M+H^+$)

2-Phenyl-1-(4-(trifluoromethyl)benzyl)-1,2,3,4-tetrahydroisoquinoline (10)

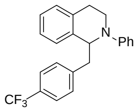

10

According to GP1. A solution of 2-phenyl-1,2,3,4-tetrahydroisoquinoline (40.1 mg, 191.6 µmol, 1.0 equiv), 2,4,6-triphenyl-1-(4-(trifluoromethyl)benzyl)pyridinium tetrafluoroborate (119.0 mg, 215.1 µmol, 1.1 equiv) and $[Ru(bpy)_3]Cl_2$ (4.4 mg, 3.1 mol %) was irradiated for 6 h. After workup and purification by column chromatography, 52.80 mg material (82% purity according to NMR, 43.3 mg, 117.8 µmol, 61% calculated yield) were collected as colorless oil, which was purified for an analytical sample by preparative HPLC.

Spectroscopic data were in accordance with literature [1].

^{1}H NMR (600 MHz, $CDCl_3$) δ = 7.46 (d, *J*=8.0, 2H), 7.28 – 7.21 (m, 2H), 7.20 – 7.10 (m, 4H), 7.08 (t, *J*=7.3, 1H), 6.83 (d, *J*=8.1, 2H), 6.79 (d, *J*=7.6, 1H), 6.76 (t, *J*=7.2, 1H), 4.92 (t, *J*=6.5, 1H), 3.63 (ddd, *J*=12.6, 7.9, 4.9, 1H), 3.55 (dt, *J*=12.1, 5.8, 1H), 3.29 (dd, *J*=13.4, 6.1, 1H), 3.09 (dd, *J*=13.4, 7.0, 1H), 2.99 (ddd, *J*=15.8, 7.8, 5.4, 1H), 2.71 (dt, *J*=15.9, 5.6, 1H).

^{13}C NMR (151 MHz, $CDCl_3$) δ = 149.4, 143.1, 137.3, 135.2, 130.2, 129.5, 128.6, 127.6, 127.0, 125.9, 125.1 (q, *J*=3.7), 124.5 (q, *J*=271.9), 117.8, 114.1, 61.3, 42.4, 42.3, 27.5.†

† carbon *ipso* to CF3 was not observed due to low sensibility of the quartet.

^{19}**F NMR** (565 MHz, $CDCl_3$) δ = -62.3.

Rf: 0.69 (LP/DCM = 1/1 – UV and $KMnO_4$)

GC-MS: STD17min; rt = 11.5 min; 208 (100), 159 (9), 115 (10), 77 (15)

LC-MS: rt = 2.3; 386 (M+H)

1-(4-Methoxybenzyl)-2-phenyl-1,2,3,4-tetrahydroisoquinoline (11)

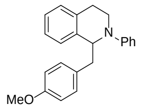

11

According to GP1. A solution of 2-phenyl-1,2,3,4-tetrahydroisoquinoline (41.0 mg, 195.9 µmol, 1.0 equiv), 1-(4-methoxybenzyl)-2,4,6-triphenylpyridinium tetrafluoroborate (107.6 mg, 208.8 µmol, 1.1 equiv) and $[Ru(bpy)_3]Cl_2$ (4.1 mg, 2.8 mol %) was irradiated for 6 h. After workup and purification by column chromatography, 85.5 mg material were collected as a mixture of 2,4,6 triphenylpyridine and the product. A yield of 44% (28.1 mg, 85.3 µmol) was calculated from NMR. The product was isolated as colorless oil by preparative HPLC.

Spectroscopic data were in accordance with literature [1].

^{1}**H NMR** (600 MHz, $CDCl_3$) δ = 7.27 – 7.22 (m, 2H), 7.14 (dt, *J*=12.9, 6.9, 2H), 7.09 – 7.03 (m, 1H), 6.92 (d, *J*=8.5, 2H), 6.86 (d, *J*=8.3, 2H), 6.79 – 6.75 (m, 3H), 6.73 (t, *J*=7.2, 1H), 4.86 (t, *J*=6.5, 1H), 3.78 (s, 3H), 3.64 (ddd, *J*=12.3, 7.5, 5.0, 1H), 3.57 – 3.49 (m, 1H), 3.19 (dd, *J*=13.6, 5.7, 1H), 3.02 – 2.93 (m, 2H), 2.74 (dt, *J*=15.8, 5.8, 1H).

^{13}**C NMR** (151 MHz, $CDCl_3$) δ = 158.3, 149.5, 137.8, 135.3, 131.1, 130.8, 129.4, 128.3, 127.8, 126.7, 125.6, 117.2, 113.7, 113.7, 61.7, 55.4, 42.3, 41.6, 27.7.

Rf: 0.26 (LP/DCM = 3/1 – UV)

GC-MS: STD14min; rt = 9.4 min; 208 (100), 165 (4), 115 (11), 77 (19)

2-Phenyl-1-(pyridin-3-ylmethyl)-1,2,3,4-tetrahydroisoquinoline (12)

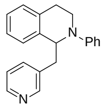

12

According to GP1. A solution of 2-phenyl-1,2,3,4-tetrahydroisoquinoline (42.2 mg, 201.6 µmol, 1.0 equiv), 2,4,6-triphenyl-1-(pyridin-3-ylmethyl)pyridinium tetrafluoroborate (105.9 mg, 217.8 µmol, 1.0 equiv) and $[Ru(bpy)_3]Cl_2$ (2.9 mg, 1.9 mol %) was irradiated for 6 h. After workup and purification by column chromatogrpahy, 25% (15.4 mg, 51.3 µmol) product were isolated as colorless oil.

^{1}**H NMR** (400 MHz, $CDCl_3$) δ = 8.44 (s, 1H), 8.29 (s, 1H), 7.32 (d, *J*=7.7, 1H), 7.27 – 7.19 (m, 2H), 7.19 – 7.08 (m, 4H), 6.90 – 6.80 (m, 3H), 6.75 (t, *J*=7.2, 1H), 4.91 (t, *J*=6.4, 1H), 3.66 – 3.61 (m, 1H), 3.60 – 3.51 (m, 1H), 3.23 (dd, *J*=13.7, 6.4, 1H), 3.06 (dd, *J*=13.7, 6.5, 1H), 3.02 – 2.90 (m, 1H), 2.66 (dt, *J*=15.9, 5.5, 1H).

^{13}**C NMR** (101 MHz, $CDCl_3$) δ = 150.4, 149.4, 147.3, 137.9, 137.0, 135.3, 134.7, 129.5, 128.7, 127.5, 127.1, 126.0, 118.0, 114.3, 61.0, 42.4, 39.7, 27.4.

Rf: 0.12 (LP/EA = 10/1 – UV)

GC-MS: STD12min; rt = 8.9 min; 208 (100), 165 (4), 128.6 (5), 115 (14), 103 (7), 77 (25)

2-Phenyl-1-(pyridin-2-ylmethyl)-1,2,3,4-tetrahydroisoquinoline (13)

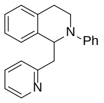

13

According to GP1. A solution of 2-phenyl-1,2,3,4-tetrahydroisoquinoline (41.9 mg, 200.2 µmol, 1.0 equiv), 2,4,6-triphenyl-1-(pyridin-2-ylmethyl)pyridinium tetrafluoroborate (97.2 mg, 199.9 µmol, 1.0 equiv) and $[Ru(bpy)_3]Cl_2$ (3.0 mg, 2.0 mol %) was irradiated for 6 h. After workup and column chromatography, 35% (20.9 mg, 69.6 µmol) product were isolated as colorless oil.

^{1}H NMR (600 MHz, $CDCl_3$) δ = 8.63 (ddd, *J*=4.9, 1.7, 0.8, 1H), 7.48 (td, *J*=7.6, 1.8, 1H), 7.19 – 7.16 (m, 2H), 7.14 (dd, *J*=6.3, 1.1, 2H), 7.13 – 7.09 (m, 1H), 7.05 – 7.01 (m, 1H), 6.91 (d, *J*=7.7, 1H), 6.86 (d, *J*=8.0, 2H), 6.81 (d, *J*=7.6, 1H), 6.70 – 6.65 (m, 1H), 5.28 (t, *J*=7.2, 1H), 3.75 (ddd, *J*=13.3, 8.7, 4.7, 1H), 3.70 – 3.63 (m, 1H), 3.47 (dd, *J*=13.4, 7.4, 1H), 3.14 (dd, *J*=13.4, 6.8, 1H), 3.12 – 3.07 (m, 1H), 2.91 (dt, *J*=15.9, 5.2, 1H).

^{13}C NMR (151 MHz, $CDCl_3$) δ = 159.1, 149.3, 149.2, 138.1, 136.2, 134.9, 129.2, 128.5, 127.3, 126.6, 125.7, 124.6, 121.4, 117.2, 113.8, 60.1, 44.8, 41.6, 27.2.

Rf: 0.12 (LP/EA = 10/1 – UV)

GC-MS: STD12min; rt = 8.7 min; 208 (100), 165 (5), 128.6 (22), 115 (17), 103 (7), 77 (31)

HRMS: calculated for $C_{21}H_{21}N_2$ $[M+H]^+$ 301.1699; found 301.1907; Δ = 3.09 ppm.

1-Benzyl-2-(4-chlorophenyl)-1,2,3,4-tetrahydroisoquinoline (21)

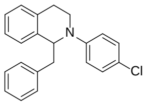

21

According to GP1. A solution of 2-(4-chlorophenyl)-1,2,3,4-tetrahydroisoquinoline (48.2 mg, 197.8 µmol, 1.0 equiv), 1-benzyl-2,4,6-triphenylpyridinium tetrafluoroborate (104.0 mg, 214.3 µmol, 1.1 equiv) and $[Ru(bpy)_3]Cl_2$ (4.3 mg, 2.9 mol %) was irradiated for 6 h. After workup and column chromatography, 41% (27.0 mg, 197.8 µmol) product were isolated as colorless oil.

^{1}H NMR (600 MHz, $CDCl_3$) δ = 7.37 – 7.30 (m, 3H), 7.30 – 7.23 (m, 4H), 7.20 (td, *J*=7.6, 1.5, 1H), 7.15 – 7.11 (m, 2H), 6.92 (d, *J*=7.6, 1H), 6.87 – 6.81 (m, 2H), 4.96 (t, *J*=6.6, 1H), 3.74 (ddd, *J*=12.6, 7.8, 5.0, 1H), 3.63 (dt, *J*=12.1, 5.7, 1H), 3.34 (dd, *J*=13.5, 6.2, 1H), 3.17 – 3.04 (m, 2H), 2.85 (dt, *J*=15.9, 5.7, 1H).

^{13}C NMR (151 MHz, $CDCl_3$) δ = 147.9, 138.6, 137.4, 134.9, 129.8, 129.0, 128.4, 128.2, 127.6, 126.8, 126.4, 125.7, 121.9, 114.8, 61.6, 42.4, 42.2, 27.3.

Rf: 0.58 (LP/DCM = 1/1 – UV)

GC-MS: STD12min; rt = 9.4 min; 242 (100), 207 (7), 138.0 (9), 115 (20), 91 (15).

HRMS: calculated for $C_{22}H_{21}ClN$ $[M+H]^+$ 334.1357; found 334.1360; Δ = 0.76 ppm.

Sideproduct of 1-benzyl-2-(4-chlorophenyl)-1,2,3,4-tetrahydroisoquinoline (21a)

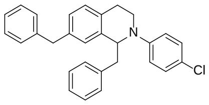

^{1}H NMR (600 MHz, $CDCl_3$) δ = 7.29 (td, *J*=7.7, 1.8, 2H), 7.25 – 7.16 (m, 6H), 7.13 (dt, *J*=8.7, 3.3, 2.1, 2H), 7.02 (dd, *J*=7.8, 1.5, 2H), 6.95 (s, 1H), 6.91 (dd, *J*=7.9, 1.6, 1H), 6.73 (d, *J*=7.8, 3H), 4.82 (t, *J*=6.6, 1H), 3.93 (s, 2H), 3.61 (ddd, *J*=12.6, 8.1, 5.0, 1H), 3.52 (dt, *J*=12.1, 5.7, 1H), 3.22 (s, 1H), 3.00 (dd, *J*=13.5, 6.8, 1H), 2.96 – 2.88 (m, 1H), 2.70 (d, *J*=15.7, 1H).

^{13}C NMR (151 MHz, $CDCl_3$) δ 147.1‡ 141.0, 139.7, 138.5, 134.7, 129.7, 129.0, 128.9, 128.8, 128.5, 128.2, 127.6, 126.5, 126.4, 126.1, 61.8, 42.2, 41.5, 27.0.

Rf: 0.58 (LP/DCM = 1/1 – UV)

LC-MS: rt. = 2.3; 424 ($M+H^+$)

HRMS: calculated for $C_{29}H_{26}ClN$ $[M+H]^+$ 424.1827; found 424.1833; Δ = 1.08 ppm.

1-Benzyl-2-(4-bromophenyl)-1,2,3,4-tetrahydroisoquinoline (22)

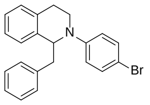

22

According to GP1. A solution of 2-(4-bromophenyl)-1,2,3,4-tetrahydroisoquinoline (61.2 mg, 212.4 µmol, 1.0 equiv), 1-benzyl-2,4,6-triphenylpyridinium tetrafluoroborate (110.0 mg, 226.6 µmol, 1.1 equiv) and $[Ru(bpy)_3]Cl_2$ (3.4 mg, 2.1 mol %) was irradiated for 6 h. After workup and column chromatography, 26% (20.9 mg, 55.3 µmol) product were isolated as colorless oil.

^{1}H NMR (600 MHz, $CDCl_3$) δ = 7.30 – 7.26 (m, 2H), 7.24 – 7.16 (m, 4H), 7.14 (d, *J*=6.9, 1H), 7.08 (td, *J*=7.6, 1.4, 1H), 7.01 (dd, *J*=7.7, 1.6, 2H), 6.80 (d, *J*=7.6, 1H), 6.71 – 6.63 (m, 2H), 4.84 (t, *J*=6.6, 1H), 3.62 (ddd, *J*=12.5, 7.8, 5.0, 1H), 3.55 – 3.46 (m, 1H), 3.22 (dd, *J*=13.5, 6.2, 1H), 3.02 (dd, *J*=13.5, 7.0, 1H), 3.00 – 2.95 (m, 1H), 2.74 (dt, *J*=15.8, 5.8, 1H).

^{13}C NMR (151 MHz, $CDCl_3$) δ = 148.4, 138.7, 137.4, 135.0, 132.0, 129.8, 128.5, 128.4, 127.7, 126.9, 126.5, 125.8, 115.2, 109.1, 61.6, 42.5, 42.3, 27.4.

Rf: 0.56 (LP/DCM = 1/1 – UV)

GC-MS: STD12min; rt = 9.4 min; 286 (100), 207 (16), 115 (26), 91 (23).

HRMS: calculated for $C_{22}H_{21}BrN$ $[M+H]^+$ 378.0852; found 378.0854; Δ = 0.76 ppm.

‡ from HMBC

4-(1-Benzyl-3,4-dihydroisoquinolin-2(1*H*)-yl)benzonitrile (23)

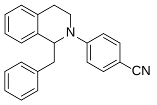

23

According to GP1. A solution of 4-(3,4-dihydroisoquinolin-2(1*H*)-yl)benzonitrile (45.4 mg, 193.8 µmol, 1.0 equiv), 1-benzyl-2,4,6-triphenylpyridinium tetrafluoroborate (105.9 mg, 218.2 µmol, 1.1 equiv) and $[Ru(bpy)_3]Cl_2$ (6.0 mg, 4.1 mol %) was irradiated for 6 h. After workup and column chromatography, 34% (20.9 mg, 55.3 µmol) product were isolated as colorless oil. For an analytical sample, the material was purified by preparative HPLC.

^{1}H NMR (400 MHz, $CDCl_3$) δ = 7.44 (d, *J*=9.1, 2H), 7.24 – 7.14 (m, 5H), 7.11 (td, *J*=7.3, 7.1, 1.6, 1H), 6.98 (dd, *J*=6.4, 3.0, 2H), 6.82 (d, *J*=7.6, 1H), 6.73 (d, *J*=9.1, 2H), 4.94 (t, *J*=6.6, 1H), 3.69 (dt, *J*=11.9, 5.4, 1H), 3.55 (ddd, *J*=12.4, 8.0, 5.1, 1H), 3.23 (dd, *J*=13.4, 6.1, 1H), 3.05 (dd, *J*=13.5, 7.2, 1H), 2.98 (dt, *J*=11.6, 5.8, 1H), 2.89 – 2.77 (m, 1H).

^{13}C NMR (101 MHz, $CDCl_3$) δ = 151.6, 137.9, 136.8, 134.7, 129.8, 128.5, 128.3, 127.7, 127.3, 126.9, 126.2, 120.6, 112.1, 98.2, 61.1, 42.6, 42.4, 27.6.

Rf: 0.31 (LP/EA = 10/1 – UV)

GC-MS: STD12min; rt = 10.8 min; 233 (100), 190 (4), 115 (19), 102 (14).

1-Benzyl-2-(4-methoxyphenyl)-1,2,3,4-tetrahydroisoquinoline (24)

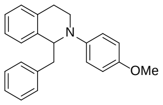

24

According to GP1. A solution of 2-(4-methoxyphenyl)-1,2,3,4-tetrahydroisoquinoline (42.0 mg, 175.5 µmol, 1.0 equiv), 1-benzyl-2,4,6-triphenylpyridinium tetrafluoroborate (162.3 mg, 334.4 µmol, 1.0 equiv) and $[Ru(bpy)_3]Cl_2$ (5.1 mg, 2.0 mol %) was irradiated for 6 h. After workup and column chromatography, 61% (60.0 mg, 182.1 µmol) product were isolated as colorless oil.

^{1}H NMR (400 MHz, $CDCl_3$) δ = 7.19 – 7.01 (m, 5H), 6.99 – 6.93 (m, 3H), 6.77 – 6.70 (m, 4H), 6.66 (d, *J*=7.6, 1H), 4.70 (t, *J*=6.5, 1H), 3.67 (s, 3H), 3.53 (ddd, *J*=12.6, 9.1, 4.8, 1H), 3.46 – 3.38 (m, 1H), 3.12 (dd, *J*=13.4, 6.1, 1H), 2.99 – 2.82 (m, 2H), 2.62 (dt, *J*=16.2, 4.8, 1H).

^{13}C NMR (101 MHz, $CDCl_3$) δ = 152.5, 144.4, 139.4, 138.0, 135.0, 129.9, 128.7, 128.2, 127.7, 126.6, 126.2, 125.5, 117.0, 114.8, 62.5, 55.8, 42.7, 42.2, 27.3.

Rf: 0.25 (LP/DCM = 1/1 – UV)

GC-MS: STD17min; rt = 12.7 min; 238 (100), 223 (6), 195 (6), 115 (10), 91 (16).

HRMS: calculated for $C_{23}H_{24}NO$ $[M+H]^+$ 330.1852; found 330.1856; Δ = 1.06 ppm.

Methyl 4-(1-benzyl-3,4-dihydroisoquinolin-2(1*H*)-yl)benzoate (25)

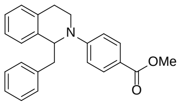

25

According to GP1. A solution of methyl 4-(3,4-dihydroisoquinolin-2(1*H*)-yl)benzoate (55.5 mg, 207.6 µmol, 1.0 equiv), 1-benzyl-2,4,6-triphenylpyridinium tetrafluoroborate (103.2 mg, 212.6 µmol, 1.0 equiv) and $[Ru(bpy)_3]Cl_2$ (3.2 mg, 2.1 mol %) was irradiated for 6 h. After workup and column chromatography, 26% (19.0 mg, 57.7 µmol) product were isolated as colorless oil. For an analytical sample, the material was purified by preparative HPLC.

^{1}H NMR (400 MHz, $CDCl_3$) δ = 7.89 (d, *J*=9.1, 2H), 7.25 – 7.13 (m, 5H), 7.11 – 7.04 (m, 1H), 6.99 (dd, *J*=7.0, 2.3, 2H), 6.80 (d, *J*=7.7, 1H), 6.76 (d, *J*=9.1, 2H), 4.99 (t, *J*=6.6, 1H), 3.86 (s, 3H), 3.75 – 3.54 (m, 3H), 3.26 (dd, *J*=13.4, 5.8, 1H), 3.12 – 2.92 (m, 2H), 2.87 – 2.74 (m, 1H).

^{13}C NMR (101 MHz, $CDCl_3$) δ = 167.3, 152.1, 138.1, 136.9, 134.9, 131.4, 129.7, 128.3, 128.1, 127.6, 127.0, 126.6, 125.9, 117.6, 111.3, 60.9, 51.5, 42.4 (d, *J*=13.2), 27.6.

Rf: 0.23 (LP/EA = 20/1 – UV)

GC-MS: STD14min; rt = 11.3 min; 266 (100), 207(10), 162 (4), 115 (12), 91 (7).

HRMS: calculated for $C_{24}H_{24}NO_2$ $[M+H]^+$ 358.1802; found 358.1809; Δ = 1.95 ppm.

1-Benzyl-7-fluoro-2-phenyl-1,2,3,4-tetrahydroisoquinoline (26)

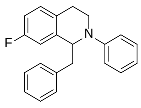

26

According to GP1. A solution of 7-fluoro-2-phenyl-1,2,3,4-tetrahydroisoquinoline (44.7 mg, 196.7 µmol, 1.0 equiv), 1-benzyl-2,4,6-triphenylpyridinium tetrafluoroborate (100.3 mg, 206.7 µmol, 1.1 equiv) and $[Ru(bpy)_3]Cl_2$ (4.6 mg, 2.0 mol %) was irradiated for 6 h. After workup and purification by column chromatography, 44% (27.3 mg, 86.0 µmol) product were isolated as colorless oil.

^{1}H NMR (400 MHz, $CDCl_3$) δ = 7.31 – 7.16 (m, 5H), 7.08 (dd, *J*=8.3, 5.7, 1H), 7.04 (dd, *J*=7.7, 1.6, 2H), 6.91 – 6.79 (m, 3H), 6.76 (t, *J*=7.3, 1H), 6.45 (dd, *J*=9.5, 2.6, 1H), 4.87 (t, *J*=6.6, 1H), 3.71 – 3.51 (m, 2H), 3.25 (dd, *J*=13.4, 5.9, 1H), 3.00 (dd, *J*=13.4, 7.3, 1H),3.00 – 2.91 (m, 1H), 2.70 (dt, *J*=15.8, 5.5, 1H).

^{13}C NMR (101 MHz, $CDCl_3$) δ = 160.6 (d, *J*=243.9), 149.2, 139.5 (d, *J*=6.6), 138.5, 130.6 (d, *J*=2.9), 129.7, 129.6, 129.3, 128.3, 126.5, 117.7, 114.1 (d, *J*=21.6), 114.0, 113.6 (d, *J*=21.3), 61.5, 42.2, 42.16, 42.10, 26.7.

^{19}F NMR (376 MHz, $CDCl_3$) δ = -117.04.

Rf: 0.43 (LP/DCM = 1/1 – UV)

GC-MS: STD12min; rt = 8.5 min; 226 (100), 183 (3), 133 (13), 104 (12), 77 (20).

HRMS: calculated for $C_{22}H_{21}FN$ $[M+H]^+$ 318.1653; found 318.1657; Δ = 1.24 ppm.

1-Benzyl-6,7-dimethoxy-2-phenyl-1,2,3,4-tetrahydroisoquinoline (27)

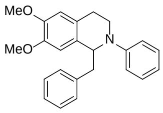

27

According to GP1. A solution of 6,7-dimethoxy-2-phenyl-1,2,3,4-tetrahydroisoquinoline (43.8 mg, 162.6 µmol, 1.0 equiv), 1-benzyl-2,4,6-triphenylpyridinium tetrafluoroborate (86.3 mg, 177.8 µmol, 1.1 equiv) and $[Ru(bpy)_3]Cl_2$ (3.9 mg, 3.2 mol %) was irradiated for 6 h. After workup and column chromatography, 53% (30.7 mg, 85.4 µmol) product were isolated as colorless oil.

^{1}H NMR (600 MHz, $CDCl_3$) δ = 7.27 (ddd, *J*=12.4, 8.1, 6.7, 4H), 7.24 – 7.18 (m, 1H), 7.11 – 7.04 (m, 2H), 6.93 (d, *J*=7.8, 2H), 6.78 (t, *J*=7.1, 1H), 6.65 (s, 1H), 6.03 (s, 1H), 4.83 (dd, *J*=8.4, 5.1, 1H), 3.87 (s, 3H), 3.71 – 3.58 (m, 2H), 3.56 (s, 3H), 3.28 (dd, *J*=13.0, 4.6, 1H), 3.03 – 2.89 (m, 2H), 2.76 (dt, *J*=15.5, 5.0, 1H).

^{13}C NMR (151 MHz, $CDCl_3$) δ = 149.6, 147.7, 146.4, 139.2, 130.1, 129.4, 128.3, 126.8, 126.4, 117.6, 114.3, 111.1, 111.0, 61.3, 56.0, 55.7, 42.1, 29.8, 27.4.

Rf: 0.39 (LP/EA = 10/1 – UV)

GC-MS: STD14min; rt = 10.1 min; 268 (100), 252 (14), 224 (5), 91 (5), 77 (7).

HRMS: calculated for $C_{24}H_{26}NO_2$ $[M+H]^+$ 360.1958; found 360.1962; Δ = 1.22 ppm.

6,7-Dimethoxy-1-(4-methylbenzyl)-2-phenyl-1,2,3,4-tetrahydroisoquinoline (28)

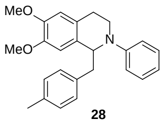

28

According to GP1. A solution of 6,7-dimethoxy-2-phenyl-1,2,3,4-tetrahydroisoquinoline (50.8 mg, 188.6 µmol, 1.0 equiv), 1-(4methylbenzyl)-2,4,6-triphenylpyridinium tetrafluoroborate (98.0 mg, 196.3 µmol, 1.0 equiv) and $[Ru(bpy)_3]Cl_2$ (2.9 mg, 2.0 mol %) was irradiated for 6 h. After workup and column chromatography, 56% (30.7 mg, 85.4 µmol) product were isolated as colorless oil.

^{1}H NMR (600 MHz, $CDCl_3$) δ = 7.48 (ddt, *J*=9.6, 7.2, 2.3, 2H), 7.27 (d, *J*=7.7, 2H), 7.14 (dd, *J*=12.1, 8.0, 4H), 7.00 – 6.93 (m, 1H), 6.84 (s, 1H), 6.25 (s, 1H), 5.00 (dd, *J*=8.4, 5.2, 1H), 4.07 (s, 3H), 3.87 – 3.75 (m, 5H), 3.44 (dd, *J*=13.3, 5.2, 1H), 3.19 – 3.12 (m, 1H), 3.09 (dd, *J*=13.2, 8.4, 1H), 2.94 (dt, *J*=15.7, 5.4, 1H), 2.52 (s, 3H).

^{13}C NMR (151 MHz, $CDCl_3$) δ = 149.6, 147.6, 146.4, 136.1, 135.8, 129.9, 129.4, 129.0, 126.8, 117.5, 114.2, 111.1 (d, *J*=8.1), 61.3, 55.9, 55.6, 42.1, 41.7, 27.3, 21.1.

Rf: 0.25 (LP/EA = 10/1 – UV)

GC-MS: STD14min; rt = 10.5 min; 268 (100), 252 (15), 224 (6), 105 (11), 77 (15).

HRMS: calculated for $C_{25}H_{28}NO_2$ $[M+H]^+$ 374.2115; found 374.2118 Δ = 1.07 ppm.

6,7-Dimethoxy-1-(2-methylbenzyl)-2-phenyl-1,2,3,4-tetrahydroisoquinoline (29)

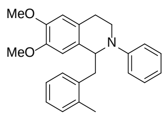

29

According to GP1. A solution of 6,7-dimethoxy-2-phenyl-1,2,3,4-tetrahydroisoquinoline (50.0 mg, 185.6 µmol, 1.0 equiv), 2,4,6-triphenyl-1-(3-methylbenzylbenzyl)pyridinium tetrafluoroborate (99.4 mg, 199.0 µmol, 1.1 equiv) and $[Ru(bpy)_3]Cl_2$ (2.8 mg, 2.0 mol %) was irradiated for 6 h. After workup and column chromatography, 42% (29.2 mg, 77.6 µmol) product were isolated as colorless oil.

^{1}H NMR (600 MHz, $CDCl_3$) δ = 7.45 – 7.38 (m, 2H), 7.33 – 7.23 (m, 3H), 7.21 (s, 1H), 7.07 (d, *J*=8.2, 2H), 6.92 (t, *J*=7.2, 1H), 6.77 (s, 1H), 6.00 (s, 1H), 4.94 (dd, *J*=9.0, 5.4, 1H), 3.99 (s, 3H), 3.84 (ddd, *J*=13.2, 8.5, 4.9, 1H), 3.76 (dt, *J*=11.9, 5.7, 1H), 3.61 (s, 3H), 3.33 (dd, *J*=13.2, 5.4, 1H), 3.18 (dd, *J*=13.2, 9.0, 1H), 3.13 (ddd, *J*=14.5, 8.4, 5.7, 1H), 2.95 (dt, *J*=15.8, 5.2, 1H), 2.08 (s, 3H).

^{13}C NMR (151 MHz, $CDCl_3$) δ = 149.6, 147.6, 146.3, 137.5, 137.2, 130.6, 130.2, 129.3, 129.1, 126.6, 126.3, 126.0, 117.7, 114.4, 110.9, 110.7, 60.9, 55.9, 55.5, 42.0, 38.1, 27.3, 19.4.

Rf: 0.21 (LP/EA = 10/1 – UV)

GC-MS: STD12min; rt = 10.4 min; 268 (100), 252 (15), 224 (5), 105 (11), 77 (18).

HRMS: calculated for $C_{25}H_{27}NO_2$ $[M+H]^+$ 374.2115; found 374.2118; Δ = 1.17 ppm.

6,7-Dimethoxy-1-(4-fluorobenzyl)-2-phenyl-1,2,3,4-tetrahydroisoquinoline (30)

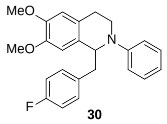

30

According to GP1. A solution of 6,7-dimethoxy-2-phenyl-1,2,3,4-tetrahydroisoquinoline (49.8 mg, 184.9 µmol, 1.0 equiv), 1-(4-fluorobenzyl)-2,4,6-triphenylpyridinium tetrafluoroborate (100.0 mg, 198.7 µmol, 1.1 equiv) and $[Ru(bpy)_3]Cl_2$ (3.7 mg, 2.6 mol %) was irradiated for 6 h. After workup and purification by column chromatography, 47% (33.1 mg, 87.7 µmol) product were isolated as colorless oil.

^{1}H NMR (600 MHz, $CDCl_3$) δ = 7.30 – 7.22 (m, 2H), 6.98 (ddd, *J*=8.2, 5.3, 2.4, 2H), 6.96 – 6.91 (m, 2H), 6.88 (d, *J*=8.0, 2H), 6.77 (t, *J*=7.2, 1H), 6.63 (s, 1H), 6.11 (s, 1H), 4.78 (dd, *J*=7.8, 5.5, 1H), 3.86 (s, 3H), 3.63 (s, 3H), 3.62 – 3.52 (m, 2H), 3.20 (dd, *J*=13.4, 5.4, 1H), 3.00 – 2.83 (m, 2H), 2.67 (dt, *J*=15.7, 5.4, 1H).

^{13}C NMR (151 MHz, $CDCl_3$) δ = 161.8 (d, *J*=244.4), 149.5, 147.8, 146.6, 134.8 (d, *J*=3.2), 131.4 (d, *J*=7.8), 129.4, 129.2, 127.0, 117.7, 115.0 (d, *J*=21.1), 114.2, 111.2, 110.9, 61.2, 55.9 (d, *J*=23.9), 42.2, 41.3, 27.3.

^{19}F NMR (565 MHz, $CDCl_3$) δ = -117.0.

Rf: 0.18 (LP/EA = 10/1 – UV)

GC-MS: STD14min; rt = 10.5 min; 268 (100), 252 (16), 224 (7), 109 (18), 77 (17).

6,7-Dimethoxy-2-phenyl-1-(4-(trifluoromethyl)benzyl)-1,2,3,4-tetrahydroisoquinoline (31)

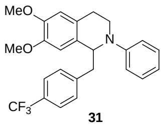

31

According to GP1. A solution of 6,7-dimethoxy-2-phenyl-1,2,3,4-tetrahydroisoquinoline (49.8 mg, 184.9 µmol, 1.0 equiv), 2,4,6-triphenyl-1-(4-(trifluoromethyl)benzyl)pyridinium tetrafluoroborate (104.8 mg, 189.4 µmol, 1.0 equiv) and $[Ru(bpy)_3]Cl_2$ (3.1 mg, 2.3 mol %) was irradiated for 6 h. After workup and column chromatography, 30% (23.3 mg, 54.5 µmol) product were isolated as colorless oil.

^{1}H NMR (600 MHz, $CDCl_3$) δ = 7.50 (d, *J*=8.0, 2H), 7.27 (td, *J*=7.3, 7.1, 2.1, 2H), 7.15 (d, *J*=8.0, 2H), 6.90 (d, *J*=8.1, 2H), 6.79 (t, *J*=7.2, 1H), 6.64 (s, 1H), 6.03 (s, 1H), 4.83 (dd, *J*=7.9, 5.6, 1H), 3.86 (s, 3H), 3.66 – 3.59 (m, 2H), 3.57 (s, 3H), 3.30 (dd, *J*=13.0, 5.1, 1H), 3.01 (dd, *J*=13.2, 8.0, 1H), 2.98 – 2.90 (m, 1H), 2.70 (dt, *J*=15.6, 5.1, 1H).

^{13}C NMR (151 MHz, $CDCl_3$) δ = 149.4, 147.8, 146.6, 143.3, 130.3, 129.4, 128.5 (q, *J*=32.9), 125.1 (q, *J*=3.7), 123.4 (q, *J*=272.3), 118.0, 114.4, 111.1, 110.6, 61.0, 55.9, 55.6, 42.2, 41.8, 27.1.

^{19}F NMR (565 MHz, $CDCl_3$) δ = -62.3.

Rf: 0.25 (LP/EA = 10/1 – UV)

GC-MS: STD12min; rt = 9.6 min; 268 (100), 252 (15), 224 (7), 109 (6), 77 (15).

HRMS: calculated for $C_{25}H_{25}F_3NO_2$ $[M+H]^+$ 428.1832; found 428.1845; Δ = 2.75 ppm.

6,7-Dimethoxy-1-(4-methoxybenzyl)-2-phenyl-1,2,3,4-tetrahydroisoquinoline (32)

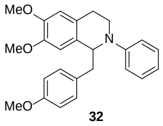

32

According to GP1. A solution of 6,7-dimethoxy-2-phenyl-1,2,3,4-tetrahydroisoquinoline (52.1 mg, 193.4 µmol, 1.0 equiv), 2,4,6-triphenyl-1-(4-methoxybenzylbenzyl)pyridinium tetrafluoroborat**e** (100.9 mg, 195.8 µmol, 1.0 equiv) and $[Ru(bpy)_3]Cl_2$ (2.9 mg, 2.0 mol %) was irradiated for 6 h. After workup and column chromatography, 40% (30.2 mg, 77.5 µmol) product were isolated as colorless oil.

^{1}H NMR (600 MHz, $CDCl_3$) δ = 7.32 – 7.21 (m, 2H), 6.96 – 6.93 (m, 2H), 6.90 (d, *J*=8.0, 2H), 6.81 – 6.77 (m, 2H), 6.76 (t, *J*=7.2, 1H), 6.63 (s, 1H), 6.08 (s, 1H), 4.77 (dd, *J*=8.2, 5.2, 1H), 3.86 (s, 3H), 3.77 (s, 2H), 3.64 – 3.51 (m and s, 5H), 3.19 (dd, *J*=13.4, 5.1, 1H), 2.97 – 2.90 (m, 1H), 2.88 (dd, *J*=13.4, 8.2, 1H), 2.70 (dt, *J*=15.7, 5.4, 1H).

^{13}C NMR (151 MHz, $CDCl_3$) δ = 158.3, 149.6, 147.6, 146.4, 131.2, 131.0, 129.5, 129.4, 126.9, 117.5, 114.1, 113.7, 111.1, 61.3, 55.9, 55.8, 55.4, 42.2, 41.2, 27.4.

Rf: 0.20 (LP/EA = 10/1 – UV)

GC-MS: STD12min; rt = 9.6 min; 268 (100), 252 (15), 224 (5), 121 (17), 104 (6), 77 (17).

HRMS: calculated for $C_{25}H_{27}NO_3$ $[M+H]^+$ 390.2064; found 390.2068; Δ = 1.12 ppm.

1-Isopropyl-2-phenyl-1,2,3,4-tetrahydroisoquinoline (34)

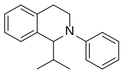

34

According to GP1. A solution of 2-phenyl-1,2,3,4-tetrahydroisoquinoline (41.2 mg, 196.9 µmol, 1.0 equiv), 1-isopropyl-2,4,6-triphenylpyridinium tetrafluoroborate (104.2 mg, 237.8 µmol, 1.0 equiv) and $[Ir(dtbbpy)(ppy)_2]PF_6$ (3.5 mg, 2.0 mol %) in degassed DMA was irradiated for 60 h. After workup, 42% (20.7 mg, 82.4 µmol) product were isolated as colorless oil.

Spectroscopic data were in accordance with literature [2].

^{1}H NMR (600 MHz, $CDCl_3$) δ = 7.24 (td, *J*=7.2, 2.0, 2H), 7.21 – 7.10 (m, 4H), 6.89 (d, *J*=8.2, 2H), 6.71 (t, *J*=7.2, 1H), 4.41 (d, *J*=8.1, 1H), 3.76 (dt, *J*=12.1, 6.0, 1H), 3.49 (dt, *J*=12.1, 7.0, 1H), 3.09 – 2.93 (m, 2H), 2.15 (dq, *J*=13.9, 6.9, 1H), 1.09 (d, *J*=6.9, 3H), 0.97 (d, *J*=6.7, 3H).

^{13}C NMR (151 MHz, $CDCl_3$) δ = 150.2, 137.9, 135.5, 129.2, 128.4, 128.3, 126.7, 125.4, 116.6, 113.3, 64.7, 43.1, 34.5, 27.5, 20.8, 20.2.

Rf: 0.53 (LP/DCM = 2/1 – UV)

GC-MS: STD12min; rt = 7.1 min; 208 (100), 115 (10), 104 (5), 77 (13).

1-(*sec*-Butyl)-2-phenyl-1,2,3,4-tetrahydroisoquinoline (35)

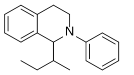

35

According to GP1. A solution of 2-phenyl-1,2,3,4-tetrahydroisoquinoline (40.0 mg, 191.1 µmol, 1.0 equiv), 1-(*sec*-butyl)-2,4,6-triphenylpyridiniumtetrafluoroborate (104.0 mg, 230.4 µmol, 1.2 equiv) and $[Ir(dtbbpy)(ppy)_2]PF_6$ (3.5 mg, 2.0 mol %) in degassed DMA was irradiated for 60 h. After workup and purification by column chromatography, 37% (20.7 mg, 82.4 µmol) product were isolated as colorless oil (diastereoisomers)

Spectroscopic data are in accordance with literature [3].

^{1}H NMR (600 MHz, $CDCl_3$) δ = 7.23 (td, *J*=8.1, 7.4, 1.6, 4H), 7.20 – 7.08 (m, 8H), 6.87 (d, *J*=7.9, 4H), 6.69 (t, *J*=6.6, 2H), 4.48 (d, *J*=8.3, 1H), 4.46 (d, *J*=8.1, 1H), 3.74 (dq, *J*=11.2, 5.6, 2H), 3.58 – 3.39 (m, 2H), 3.18 – 2.90 (m, 4H), 1.91 – 1.84 (m, 2H), 1.78 (ddd, *J*=13.4, 7.6, 3.4, 2H), 1.64 – 1.51 (m, 1H), 1.38 – 1.19 (m, 3H), 1.18 – 1.10 (m, 1H), 1.05 (d, *J*=6.9, 3H), 0.99 – 0.72 (m, 8H).

^{13}C NMR (151 MHz, $CDCl_3$) δ = 150.2, 138.3, 135.5, 129.2, 129.2, 128.4, 128.3, 128.3, 126.7, 125.4, 116.6, 113.3, 63.9, 63.4, 43.4, 43.3, 41.4, 41.3, 29.8, 27.5, 26.8, 26.4, 16.5, 16.5, 12.3, 11.9.

Rf: 0.50 (LP/DCM = 2/1 – UV)

GC-MS: STD12min; rt = 7.3 min; 208 (100), 115 (12), 104 (7), 77 (19).

HRMS: calculated for $C_{19}H_{24}N$ $[M+H]^+$ 266.1903; found 266.1905; Δ = 0.82 ppm.

1-Cyclohexyl-2-phenyl-1,2,3,4-tetrahydroisoquinoline (36)

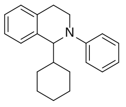

36

According to GP1. A solution of 2-phenyl-1,2,3,4-tetrahydroisoquinoline (40.8 mg, 194.9 µmol, 1.0 equiv), 1-cyclohexyl-2,4,6-triphenylpyridinium tetrafluoroborate (93.2 mg, 195.2 µmol, 1.0 equiv) and $[Ir(dtbbpy)(ppy)_2]PF_6$ (2.2 mg, 1.2 mol %) in degassed DMA was irradiated for 48 h. After workup and column chromatography, 26% (15.0 mg, 51.5 µmol) product were isolated as colorless oil.

Spectroscopic data were in accordance with literature [1].

^{1}H NMR (600 MHz, $CDCl_3$) δ = 7.25 – 7.21 (m, 2H), 7.20 – 7.11 (m, 3H), 7.08 (d, *J*=7.5, 1H), 6.87 (d, *J*=7.7, 2H), 6.69 (t, *J*=6.9, 1H), 4.44 (d, *J*=8.2, 1H), 3.74 (dt, *J*=11.7, 5.8, 1H), 3.48 (dt, *J*=12.0, 7.1, 1H), 3.09 – 2.95 (m, 2H), 1.99 (d, *J*=12.1, 1H), 1.79 – 1.71 (m, 4H), 1.67 – 1.60 (m, 1H), 1.24 – 1.00 (m, 5H).

^{13}C NMR (151 MHz, $CDCl_3$) δ = 150.1, 138.0, 135.4, 129.2, 128.5, 128.3, 126.7, 125.3, 116.4, 113.1, 63.9, 44.2, 43.1, 27.5, 26.8, 26.55, 26.52.

Rf: 0.70 (LP/DCM = 1/1 – UV)

GC-MS: STD12min; rt = 8.2 min; 208 (100), 130 (3), 115 (11), 77 (16).

1-Allyl-2-phenyl-1,2,3,4-tetrahydroisoquinoline (37)

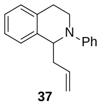

37

According to GP1. A solution of 2-phenyl-1,2,3,4-tetrahydroisoquinoline (41.0 mg, 195.9 µmol, 1.0 equiv), 2,4,6-triphenyl-1-(allyl)pyridinium tetrafluoroborate (87.4 mg, 200.8 µmol, 1.0 equiv) and $[Ru(bpy)_3]Cl_2$ (5.8 mg, 3.9 mol %) was irradiated for 6 h. After workup and purification by column chromatography, 66% (32.2 mg, 129.1 µmol) product were isolated as colorless oil.

Spectroscopic data were in accordance with literature [1].

^{1}H NMR (600 MHz, $CDCl_3$) δ = 7.33 – 7.27 (m, 2H), 7.23 – 7.11 (m, 4H), 6.94 (d, *J*=8.2, 2H), 6.78 (t, *J*=7.2, 1H), 5.90 (ddt, *J*=17.2, 10.1, 7.2, 1H), 5.18 – 5.02 (m, 2H), 4.79 (t, *J*=6.8, 1H), 3.74 – 3.59 (m, 2H), 3.06 (ddd, *J*=15.6, 8.1, 5.5, 1H), 2.92 (dt, *J*=15.9, 5.3, 1H), 2.78 (dt, *J*=13.8, 6.8, 1H), 2.53 (dt, *J*=14.3, 7.2, 1H).

^{13}C NMR (151 MHz, $CDCl_3$) δ = 149.6, 138.3, 135.8, 135.1, 129.4, 128.6, 127.5, 126.7, 125.9, 117.1, 114.0, 59.5, 42.0, 41.1, 27.5.

Rf: 0.51 (LP/DCM = 1/1 – UV)

GC-MS: STD12min; rt = 7.1 min; 208 (100), 165 (4), 128.6 (4), 77 (21).

1-Allyl-6,7-dimethoxy-2-phenyl-1,2,3,4-tetrahydroisoquinoline (38)

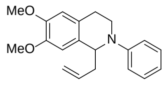

38

According to GP1. A solution of 6,7-dimethoxy-2-phenyl-1,2,3,4-tetrahydroisoquinoline (50.1 mg, 186.0 µmol, 1.0 equiv), 1-allyl-2,4,6-triphenylpyridinium tetrafluoroborate (91.2 mg, 209.5 µmol, 1.1 equiv) and $[Ru(bpy)_3]Cl_2$ (3.3 mg, 2.4 mol %) was irradiated for 3 h. After workup and column chromatography, 35% (19.9 mg, 64.3 µmol) product were isolated as colorless oil (diastereoisomers).

Spectroscopic data were in accordance with the literature [4].

^{1}H NMR (600 MHz, $CDCl_3$) δ = 7.26 – 7.22 (m, 2H), 6.90 (d, *J*=8.1, 2H), 6.74 (t, *J*=7.2, 1H), 6.63 (d, *J*=1.2, 2H), 6.03 – 5.62 (m, 1H), 5.13 – 4.97 (m, 2H), 4.68 (t, *J*=6.8, 1H), 3.86 (s, 3H), 3.86 (s, 3H), 3.67 – 3.56 (m, 2H), 2.96 (ddd, *J*=15.1, 8.9, 5.7, 1H), 2.83 – 2.68 (m, 2H), 2.57 – 2.46 (m, 1H).

^{13}C NMR (151 MHz, $CDCl_3$) δ = 149.8, 147.7, 147.1, 136.0, 130.3, 129.4, 127.0, 117.6, 117.1, 114.5, 111.5, 110.7, 59.1, 56.2, 56.0, 41.8, 41.0, 26.9.

Rf: 0.20 (LP/EA = 10/1 – UV)

GC-MS: STD14min; rt = 8.3 min; 268 (100), 252 (15), 224 (7), 104 (9), 77 (19).

5. *N*-Dearylation procedure

1-Benzyl-1,2,3,4-tetrahydroisoquinoline (33)

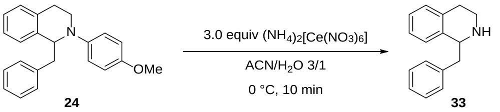

A solution of CAN in H_2O (250 µL) was added dropwise over a period of five minutes to a solution of 1-benzyl-2-(4-methoxyphenyl)-1,2,3,4-tetrahydroisoquinoline (**24**, 19.5 mg, 59.2 µmol) in ACN (600 µL, 0.1 M solution) at 0 °C (ice bath). During addition, the colour changed immediately to deep red and turned yellow after complete addition. A reaction control showed full conversion of the starting material. The solution was diluted with EA (20 mL) and water (5 mL). After separation of the two phases, the aqueous phase was extracted twice with EA (15 mL each). The combined organic phases were washed with brine and dried over sodium sulphate, and the volatiles removed, giving a crude red oil. The pH of the aqueous phase was adjusted to 10 using NaOH (2 M) and the yellow solution was extracted with DCM (3 × 20 mL). After washing (brine) and drying (sodium sulphate), the solvents were removed, giving a crude yellow oil. The organic extracts were combined and purified by column chromatography using hex/EA/TEA 1:1:3% affording the title compound as pale yellow oil (50%, 6.6 mg, 29.6 µmol)

Spectroscopic data were in accordance with literature [5].

^{1}H NMR (600 MHz, $CDCl_3$) δ = 7.38 – 7.29 (m, 2H), 7.29 – 7.23 (m, 3H), 7.20 – 7.06 (m, 4H), 4.47 – 4.27 (m, 1H), 3.32 – 3.19 (m, 2H), 3.02 (dd, *J*=14.2, 8.7, 2H), 2.94 – 2.80 (m, 2H).

^{13}C NMR (151 MHz, $CDCl_3$) δ = 138.6, 137.4, 134.7, 129.6, 129.4, 128.8, 126.8, 126.7, 126.5, 126.0, 57.0, 42.3, 40.3, 29.2.

Rf: 0.50 (hex/EA /TEA= 1/1/3% – UV)

GC-MS: STD12min; rt = 6.9 min; 132 (100), 105 (10), 91 (5), 77 (4).

6. Synthesis of THIQs

6.1. General procedures for the arylation of THIQ (GP2 and GP3)

Generally, literature procedures were followed and were not optimized.

General procedure 2 (GP2)[6, 7]

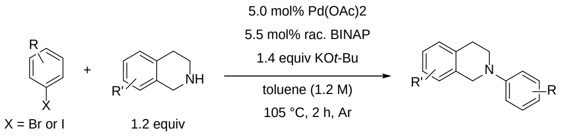

A suspension of (±)-BINAP (5.5 mol %) in dry toluene (amount calculated for a 1.2 M solution of phenyl bromide) was degassed by applying 3 quick Schlenk cycles to the stirring solution and heated to 105 °C (preheated oil bath) under vigorous stirring for 5–10 min to obtain an almost homogeneous solution, which was cooled to rt. Then, $Pd(OAc)_2$ (5.5 mol %) was added and the solution was stirred for a few minutes until the solution was deep red. Then, phenyl bromide (1.0 equiv), 1,2,3,4-tetrahydroisoquinoline derivative (1.2 equiv) and KO*t*-Bu (1.4 equiv) were added sequentially in this order (at last, the mixture became black), and the mixture was heated at 105 °C. After 2 h, the mixture was cooled to rt. TLC and/or GC–MS showed full conversion of the starting material.

The solution was filtered through Celite and rinsed with DCM. After evaporation of all volatiles the material was subjected to column chromatography using LP/EA as the eluent.

General procedure 3 (GP3)[8]

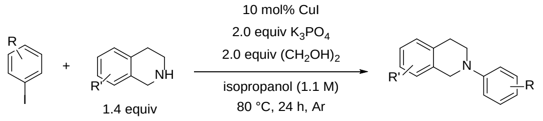

CuI (10 mol %) and K_3PO_4 (2.0 equiv) were placed in a Schlenk tube, and the flask put under argon. Then, ethylene glycol (2.0 equiv) and isopropanol (amount calculated for a 1.1 M solution based on the aryl iodide) were added, and the solution degassed. Then, aryl iodide (1.0 equiv) and the 1,2,3,4-tetrahydroisoquinoline derivative (1.4 equiv) were added in this order, and the mixture was stirred at 80 °C for 24 h. The reaction was quenched by adding water and 3.5 M NH_3 until the tetraamminecopper complex hads formed. The aqueous phase was extracted three times with Et_2O. The combined organic phases were washed with a small portion of water and brine. After drying over sodium sulphate, the crude material was purified by column chromatography using LP/EA as the eluent.

6.2. Analytical data of substrates

2-Phenyl-1,2,3,4-tetrahydroisoquinoline (1)

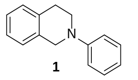

1

According to GP2 with 5.95 g bromobenzene (37.97 mmol), 6.15 g 1,2,3,4-tetrahydroisoquinoline (46.17 mmol), 1.31 g racemic BINAP (2.11 mmol, 5.5 mol %), 425.7 mg palladium(II) acetate (1.90 mmol,

5.0 mol %), 6.1 g KO*t*-Bu (54.36 mmol), and 60 mL dry toluene. After purification, 67% (5.31 g, 25.37 mmol) of an off-white solid were isolated.

Spectroscopic data were in accordance with literature [6].

^{1}H NMR (400 MHz, $CDCl_3$) δ = 7.32 (td, *J*=7.4, 1.8, 2H), 7.28 – 7.14 (m, 4H), 7.02 (d, *J*=8.1, 2H), 6.86 (t, *J*=7.3, 1H), 4.44 (s, 2H), 3.59 (t, *J*=5.9, 2H), 3.02 (t, *J*=5.9, 2H).

^{13}C NMR (101 MHz, $CDCl_3$) δ = 150.7, 135.0, 134.6, 129.3, 128.6, 126.7, 126.5, 126.2, 118.8, 115.3, 50.9, 46.7, 29.2.

Rf: 0.40 (LP/EA = 10/1 – UV)

GC-MS: STD17min; rt = 11.1 min; 208 (100), 181 (6), 104 (93), 77 (36); STD10; rt.=7.1 min.

2-(4-Chlorophenyl)-1,2,3,4-tetrahydroisoquinoline (14)

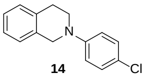

14

According to GP3 with 1.21 g 1-chloro-4-iodobenzene (5.1 mmol), 1.0 g 1,2,3,4-tetrahydroisoquinoline (7.51 mmol), 95.8 mg CuI (503 µmol), 2.16 g K_3PO_4 (10.2 mmol), 570 µL ethylene glycol (10.2 mmol) and 5 mL isopropanol. After workup and purification by column chromatography, 51% pure product (632 mg, 2.59 mmol) were obtained as white solid.

^{1}H NMR (200 MHz, $CDCl_3$) δ = 7.33 – 7.11 (m, 6H), 6.99 – 6.85 (m, 2H), 4.40 (s, 2H), 3.55 (t, *J*=5.9, 2H), 3.00 (t, *J*=5.8, 2H).

^{13}C NMR (50 MHz, $CDCl_3$) δ = 149.2, 134.8, 134.2, 129.1, 128.6, 126.6 (d, *J*=1.0), 126.3, 123.5, 116.3, 50.8, 46.6, 29.1.

Rf: 0.43 (LP/EA = 40/1 – UV)

GC-MS: STD12min; rt = 11.1 min; 242 (48), 138 (7), 104 (100), 77 (12).

HRMS: calculated for $C_{16}H_{15}N_2$ $[M+H]^+$ 235.1230; found 235.1234; Δ = 1.92 ppm.

2-(4-Bromophenyl)-1,2,3,4-tetrahydroisoquinoline (15)

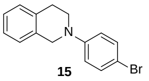

15

According to GP3 with 1.42 g 1-bromo-4-iodobenzene (5.01 mmol, 1.0 equiv), 1.0 g 1,2,3,4-tetrahydroisoquinoline (7.51 mmol, 1.5 equiv), 104.2 mg CuI (547 µmol, 10mol %), 2.13 g K_3PO_4 (10.1 mmol), 560 µL ethylene glycol (10.0 mmol), and 5 mL isopropanol. After workup, 50% pure product (720.0 mg mg, 2.50 mmol) were obtained as white solid.

Spectroscopic data were in accordance with literature [9].

^{1}H NMR (400 MHz, $CDCl_3$) δ = 7.26 (ddd, *J*=9.2, 3.3, 2.2, 2H), 7.15 – 7.01 (m, 4H), 6.73 (ddd, *J*=9.1, 3.4, 2.3, 2H), 4.28 (s, 2H), 3.43 (t, *J*=5.9, 2H), 2.88 (t, *J*=5.9, 2H).

^{13}C NMR (101 MHz, $CDCl_3$) δ = 149.5, 134.8, 134.2, 132.0, 128.6, 126.6, 126.3, 116.6, 50.5, 46.4, 29.1.

4-(3,4-Dihydroisoquinolin-2(1*H*)-yl)benzonitrile (16)

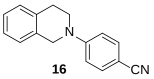

16

According to GP3 with 1.15 g 4-iodobenzonitrile (5.02 mmol), 1.0 g 1,2,3,4-tetrahydroisoquinoline (7.53 mmol), 95.8 mg CuI (502 µmol), 2.13 g K_3PO_4 (10.0 mmol), 560 µL ethylene glycol (10.0 mmol), and 5 mL isopropanol. After workup, 31% pure product (632 mg, 2.59 mmol) were obtained as white solid.

Spectroscopic data were in accordance with literature [10].

^{1}H NMR (400 MHz, $CDCl_3$) δ = 7.51 (dt, *J*=9.0, 2.8, 2.1, 2H), 7.32 – 7.15 (m, 4H), 6.86 (dt, *J*=9.1, 2.8, 2.0, 2H), 4.50 (s, 2H), 3.63 (t, *J*=5.9, 2H), 3.00 (t, *J*=5.9, 2H).

^{13}C NMR (101 MHz, $CDCl_3$) δ = 152.2, 134.9, 133.5, 128.2, 126.9, 126.5, 126.5, 120.5, 112.6, 98.5, 48.7, 44.6, 28.9.

Rf: 0.29 (LP/EA = 10/1 – UV)

GC-MS: STD12min; rt = 8.3 min; 234 (37), 133 (4), 104 (100), 78 (22).

2-(4-Methoxyphenyl)-1,2,3,4-tetrahydroisoquinoline (17)

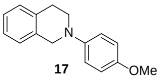

17

According to GP3 with 2.00 g 4-iodoanisole (8.55 mmol), 1.71 g 1,2,3,4-tetrahydroisoquinoline (12.58 mmol), 162.8 mg CuI (855 µmol), 3.63 g K_3PO_4 (17.0 mmol), 955 µL ethylene glycol (17.0 mmol), and 12 mL isopropanol. After workup, 27% pure product (632 mg, 2.59 mmol) were obtained as white solid.

Spectroscopic data were in accordance with literature [6].

^{1}H NMR (400 MHz, $CDCl_3$) δ = 7.23 – 7.09 (m, 4H), 7.04 – 6.96 (m, 2H), 6.93 – 6.80 (m, 2H), 4.31 (s, 2H), 3.78 (s, 3H), 3.46 (t, *J*=5.9, 2H), 2.99 (t, *J*=5.8, 2H).

^{13}C NMR (101 MHz, $CDCl_3$) δ = 153.6, 145.5, 134.7 (d, *J*=7.0), 128.8, 126.6, 126.4, 126.0, 118.2, 114.7, 55.8, 52.8, 48.6, 29.3.

Rf: 0.30 (LP/EA = 10/1 – UV)

GC-MS: STD17min; rt = 10.3 min; 239 (34), 135 (64), 120 (81), 104 (100), 77 (48).

Methyl 4-(3,4-dihydroisoquinolin-2(1*H*)-yl)benzoate (18)

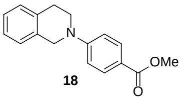

18

According to GP2 with 1.00 g methyl 4-iodobenzoate (3.82 mmol), 610 mg 1,2,3,4-tetrahydroisoquinoline (4.6 mmol, 1.2 equiv), 142.6 mg racemic BINAP (229 µmol, 5.5 mol %), 51.4 mg palladium(II) acetate (229.0 mmol, 5.5 mol %), 600 mg KO*t*-Bu (5.34 mmol), and 5 mL dry toluene. After purification, 35% (215.0 mg, 0.80 mmol) of the product as off-white solid were isolated.

Spectroscopic data were in accordance with literature [9].

^{1}H NMR (400 MHz, $CDCl_3$) δ = 7.96 (dt, *J*=9.1, 2.9, 2.1, 2H), 7.25 – 7.11 (m, 4H), 6.88 (dt, *J*=9.1, 2.8, 2.1, 2H), 4.52 (s, 2H), 3.87 (s, 3H), 3.65 (t, *J*=5.9, 2H), 2.99 (t, *J*=5.9, 2H).

^{13}C NMR (101 MHz, $CDCl_3$) δ = 167.4, 153.1, 135.2, 133.9, 131.5, 128.3, 126.8, 126.6, 126.5, 118.4, 112.2, 51.7, 49.2, 44.9, 29.2.

7-Fluoro-2-phenyl-1,2,3,4-tetrahydroisoquinoline (19)

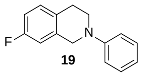

19

According to GP2 with 104.2 mg bromobenzene (663.7 µmol), 130.0 mg 7-fluoro-1,2,3,4-tetrahydroisoquinoline (**S3**, 859.9 µmol), 23.5 mg racemic BINAP (37.7 mmol, 5.7 mol %), 8.5 mg palladium(II) acetate (37.7 µmol, 5.7 mol %), 107.0 mg KO*t*-Bu (953.4 µmol), and 1 mL dry toluene. After purification, 54% (81.8 mg, 359.9 µmol) of the product as off-white solid were isolated.

^{1}H NMR (600 MHz, $CDCl_3$) δ = 7.31 (tt, *J*=7.4, 2.2, 2H), 7.12 (dd, *J*=8.1, 5.8, 1H), 7.02 – 6.96 (m, 2H), 6.93 – 6.84 (m, 3H), 4.39 (s, 2H), 3.57 (t, *J*=5.9, 2H), 2.95 (t, *J*=5.8, 2H).

^{13}C NMR (151 MHz, $CDCl_3$) δ = 161.3 (d, *J*=243.9), 150.4, 136.4 (d, *J*=7.0), 130.5 (d, *J*=2.8), 130.1 (d, *J*=7.9), 129.4, 119.2, 115.5, 113.6 (d, *J*=21.3), 113.1 (d, *J*=21.4), 50.9 (d, *J*=1.7), 46.9, 28.4.

^{19}F NMR (565 MHz, $CDCl_3$) δ = -117.01.

Rf: 0.51 (LP/EA = 20/1 – UV)

GC-MS: STD10min; rt = 6.9 min; 227 (72), 199 (6), 122 (100), 104 (16), 77 (25).

HRMS: calculated for $C_{15}H_{15}FN$ $[M+H]^+$ 228.1183; found 228.1185; Δ = 1.95 ppm.

6,7-Dimethoxy-2-phenyl-1,2,3,4-tetrahydroisoquinoline (20)

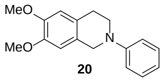

20

Prepared according to GP3 with 550.0 mg iodobenzene (2.70 mmol), 713.2 mg 6,7-dimethoxy-1,2,3,4-tetrahydroisoquinoline (**S4**, 3.69 mmol), 54.0 mg CuI (284 µmol), 1.08 g K_3PO_4 (5.09 mmol), 280 µL ethylene glycol (5.0 mmol), and 2.5 mL isopropanol. After workup, 52% pure product (376.0 mg, 1.40 mmol) were obtained as white solid.

Spectroscopic data were in accordance with literature [6].

^{1}H NMR (600 MHz, $CDCl_3$) δ = 7.29 (tt, *J*=7.4, 2.2, 2H), 6.99 (dd, *J*=8.7, 0.8, 2H), 6.83 (t, *J*=7.3, 1H), 6.65 (d, *J*=6.3, 2H), 4.34 (s, 2H), 3.88 (s, 3H), 3.87 (s, 3H), 3.55 (t, *J*=5.8, 2H), 2.90 (t, *J*=5.7, 2H).

^{13}C NMR (151 MHz, $CDCl_3$) δ = 150.7, 147.7, 129.3, 126.8, 126.3, 118.9, 115.5, 111.5, 109.5, 56.12, 56.07, 50.6, 46.9, 28.7.

Rf: 0.25 (LP/EA = 10/1 – UV)

GC-MS: STD12min; rt = 8.2 min; 269 (59), 252 (8), 164 (100), 149 (19), 77 (14).

6.3. Synthesis and analytical data of THIQ derivatives S3 and S4

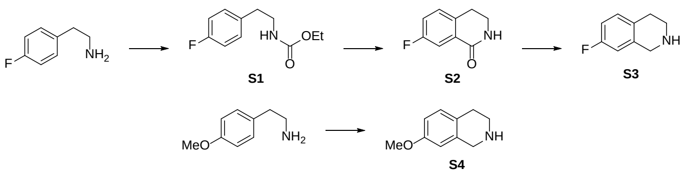

Ethyl (4-fluorophenethyl)carbamate (S1)

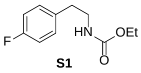

Ethyl chloroformate (1.03 g, 9.49 mmol, 1.09 equiv) was slowly added to a solution of triethylamine (968.0 mg, 9.57 mmol, 1.1 equiv) and 4-fluorophenethylamine (1.21 g, 8.69 mmol, 1.0 equiv) in dry DCM (15 mL) at 0 °C (icebath), and the mixture stirred at 25 °C for 1 hour. Then, 80 mL of water were added and the mixture was extracted with EA several times (250 mL in total). The combined organic phases were dried with brine and over sodium sulphate. The solvent was removed in vacuo, giving the desired product as an off-white solid quantitatively (1.84 g, 8.69 mmol). The compound was used without further purification.

^{1}H NMR (600 MHz, $CDCl_3$) δ = 7.14 (dd, *J*=8.3, 5.5, 2H), 7.08 – 6.89 (m, 2H), 4.65 (s, 1H), 4.10 (q, *J*=6.9, 2H), 3.52 – 3.35 (m, 2H), 2.78 (t, *J*=6.9, 2H), 1.22 (t, *J*=7.0, 3H).

^{13}C NMR (151 MHz, $CDCl_3$) δ = 161.7 (d, *J*=244.3), 156.6, 134.5, 130.2 (d, *J*=7.9), 115.4 (d, *J*=21.2), 60.8, 42.2, 35.4, 14.6.

^{19}F NMR (565 MHz, $CDCl_3$) δ = -116.7.

Rf: 0.60 (LP/EA = 3/1 – UV and $KMnO_4$)

HRMS: calculated for $C_{11}H_{15}FNO_2$ $[M+H]^+$ 212.1081; found 212.1084; Δ = 1.60 ppm.

7-Fluoro-3,4-dihydroisoquinolin-1(2*H*)-one (S2)

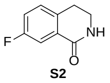

TfOH (4.20 mL, 47.86 mmol, 20.2 equiv) was added to ethyl (4-fluorophenethyl)carbamate (**S1**), which dissolved the material and caused heavy smoke formation. Then, the solution was heated to 70 °C (oil bath) overnight. The material was put on ice (100 mL) and was extracted with DCM. The combined organic phases were washed with water and brine. The solvents were dried over MgSO4 and removed giving 417.9 mg of crude material, which contained a mixture of substrate and product and was separated by column chromatography using LP/EA 2:1 to 1:2 for eluation. The product was isolated in 63% (244.9 mg, 1.48 mmol) yield as white solid. Additionally, 29% (145.5 mg, 688.8 µmol) of unreacted starting material were also recovered.[§]

Spectroscopic data are in accordance with literature [11].

^{1}H NMR (600 MHz, $CDCl_3$) δ = 7.74 (dd, *J*=9.1, 2.8, 1H), 7.19 (dd, *J*=8.3, 5.2, 1H), 7.14 (td, *J*=8.3, 2.8, 1H), 6.88 (s, 1H), 3.57 (td, *J*=6.7, 2.8, 2H), 2.96 (t, *J*=6.6, 2H).

§ Longer reaction times or increased temperatures only decreased the recovery of the reaction, without improvement of the yield

^{13}C NMR (151 MHz, $CDCl_3$) δ = 165.5, 161.9 (d, *J*=245.6), 134.5 (d, *J*=3.2), 130.8 (d, *J*=7.3), 129.0 (d, *J*=7.4), 119.2 (d, *J*=21.9), 114.6 (d, *J*=23.1), 40.2, 27.6.

^{19}F NMR (565 MHz, $CDCl_3$) δ = -114.6 – -114.7.

Rf: 0.10 (LP/EA = 1/1 – UV)

HRMS: calculated for C_9H_9FNO $[M+H]^+$ 166.0663; found 166.0664; Δ = 1.11 ppm.

7-Fluoro-1,2,3,4-tetrahydroisoquinoline (S3)

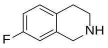

S3

A solution of 7-fluoro-3,4-dihydroisoquinolin-1(2*H*)-one (**S2**, 602.0 mg, 3.64 mmol, 1.0 equiv) in 13 mL THF was added dropwise to a slurry of LAH (207.0 mg, 5.45 mmol, 1.50 equiv) and THF (10 mL). The reaction was stirred at 65 °C (oil bath) for 4 h. The reaction mixture was diluted with diethyl ether and 2 N NaOH, and a small amount of water was added until the reaction was quenched. Then, additional Et_2O was added and the mixture was vigorously stirred, and dried with MgSO4. The solids were filtered off and the solvents removed, giving the product as pale yellow liquid in quantitative yield (551.0 mg, 3.83 mmol).

^{1}H NMR (600 MHz, $CDCl_3$) δ = 7.03 (dd, *J*=8.3, 5.9, 1H), 6.83 (td, *J*=8.5, 2.5, 1H), 6.71 (dd, *J*=9.3, 2.3, 1H), 3.99 (s, 2H), 3.12 (td, *J*=6.0, 1.0, 2H), 2.75 (t, *J*=5.8, 2H).

^{13}C NMR (151 MHz, $CDCl_3$) δ = 161.3 (d, *J*=243.9), 150.4, 136.4 (d, *J*=7.0), 130.5 (d, *J*=2.8), 130.1 (d, *J*=7.9), 129.4, 119.2, 115.5, 113.6 (d, *J*=21.3), 113.1 (d, *J*=21.4), 50.9 (d, *J*=1.7), 46.9, 28.4.

^{19}F NMR (565 MHz, $CDCl_3$) δ = -117.3.

Rf: 0.17 (DCM/MeOH = 10/1 – UV, $KMnO_4$)

HRMS: calculated for $C_9H_{11}FN$ $[M+H]^+$ 152.0870; found 152.0872; Δ = 1.00 ppm.

6,7-Dimethoxy-1,2,3,4-tetrahydroisoquinoline (S4)

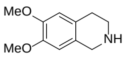

S4

2-(3,4-Dimethoxyphenyl)ethan-1-amine (2.04 g, 11.26 mmol, 1.0 equiv) was cooled to 0 °C and formic acid (4.8 mL) was added. After stirring for 5–10 min and dissolving the formed precipitate (sonic bath), paraformaldehyde (340.0 mg, 11.32 mmol, 1.0 equiv) was added, and the suspension was stirred at 50 °C overnight.

NaOH (first 2N, then conc.) was added to the solution, which was extracted three times with DCM (70 mL in total). The combined organic phases were washed with brine and dried over sodium sulphate. After evaporation of the solvent, 99% product (2.15 g, 11.14 mmol) were collected as off white solid, which was used without further purification.

Spectroscopic data are in accordance with literature [12].

^{1}H NMR (400 MHz, $CDCl_3$) δ = 6.56 (s, 1H), 6.48 (s, 1H), 3.93 (s, 2H), 3.83 (s, 3H), 3.82 (s, 3H), 3.12 (t, *J*=6.0, 2H), 2.90 (s, 1H), 2.71 (t, *J*=5.9, 2H).

^{13}C NMR (101 MHz, $CDCl_3$) δ = 147.5 (d, J=17.0), 127.2, 126.4, 112.0, 109.2, 56.0, 47.7, 43.8, 28.5.GC-MS: STD14min; rt = 6.2 min; 193 (71), 164 (100), 149 (25), 121 (24), 77 (13).

7. Synthesis of *N*-pyridinium salts (GP4)

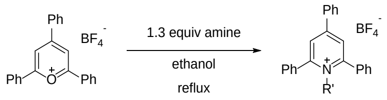

These compounds were synthesized according to literature procedures and are not optimized. [13-16]

The amine (1.2 – 1.4 equiv) was added to a suspension of 2,4,6-triphenylpyrylium tetrafluoroborate in EtOH (1 M), which led to an immediate colour change to deep red, and dissolving of the material. Then, the solution was refluxed for the indicated time and cooled to rt. Then, Et_2O was added. If a precipitate formed, the suspension was stirred for 1 h, the material was filtered off, and washed with Et_2O. After drying under vacuum at 40 °C, the product was obtained. If no precipitation had formed, the flask was put in a sonic bath, or was kept at –25 °C overnight to help precipitation. In cases where still no precipitate, but an oily/sticky material had formed, all solvents were removed, and the crude material was dissolved in DCM. Then, MTBE was added until the solution was just clear and the solution was stirred overnight in an open flask. Eventually, the product precipitated. In cases where all the above measures did not lead to a solid product, the crude material was purified by column chromatography (DCM/acetone).

7.1. Analytical data of pyridinium salts

1-Benzyl-2,4,6-triphenylpyridinium tetrafluoroborate (2)

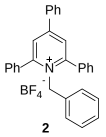

2

According to GP4 using 2,4,6-triphenylpyrylium tetrafluoroborate (4.02 g, 10.14 mmol), benzylamine (2.0 mL, 18.31 mmol) and 10 mL EtOH. The product was isolated after precipitation in 87% yield (4.30 g, 8.87 mmol) as white powder.

Spectroscopic data are in accordance with literature [15].

^{1}H NMR (400 MHz, $CDCl_3$) δ = 7.91 (s, 2H), 7.78 (d, *J*=7.2, 2H), 7.64 (d, *J*=6.9, 4H), 7.59 – 7.37 (m, 9H), 7.15 (t, *J*=7.2, 1H), 7.09 (t, *J*=7.3, 2H), 6.45 (d, *J*=7.4, 2H), 5.76 (s, 2H).

^{13}C NMR (101 MHz, $CDCl_3$) δ = 157.7, 156.4, 134.2, 133.9, 132.9, 132.5, 131.1, 129.9, 129.3, 129.2, 128.9, 128.4, 128.3, 126.7, 126.4, 58.4.

^{19}F NMR (376 MHz, $CDCl_3$) δ = -152.94 (minor, $^{10}BF_4^-$), -152.99 (major, $^{11}BF_4^-$).

2,4,6-Triphenyl-1-(4fluorobenzyl)pyridin-1-ium tetrafluoroborate (S5)

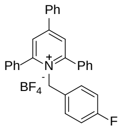

According to GP4 using 2,4,6-triphenylpyrylium tetrafluoroborate (2.0 g, 5.05 mmol), 4-fluorobenzylamine (863 µL, 6.1 mmol) and 5 mL EtOH. The product was recrystallized from EtOH and isolated as white solid in 67% yield (1.87 g, 3.38 mmol).

Spectroscopic data are in accordance with literature [15].

^{1}H NMR (400 MHz, $CDCl_3$) δ = 7.95 (s, 2H), 7.81 (dt, J=8.5, 1.2, 2H), 7.65 (dd, J=7.9, 1.2, 4H), 7.61 – 7.45 (m, 9H), 6.84 – 6.70 (m, 2H), 6.44 (dd, J=8.7, 5.1, 2H), 5.77 (s, 2H).

^{13}C NMR (151 MHz, $CDCl_3$) δ = 162.4 (d, J=248.6), 157.5, 156.6, 133.8, 132.5, 131.2, 129.9, 129.8 (d, J=3.2), 129.4, 129.2, 128.4 (d, J=8.4), 128.3, 126.8, 116.0 (d, J=21.9), 57.7.

^{19}F NMR (565 MHz, $CDCl_3$) δ = -112.57, -152.71 (minor, $^{10}BF_4^-$), -152.76 (major, $^{11}BF_4^-$).

2,4,6-Triphenyl-1-(4-(trifluoromethyl)benzyl)pyridinium tetrafluoroborate (S6)

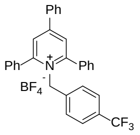

According to GP4 using 2,4,6-triphenylpyrylium tetrafluoroborate (2.0 g, 5.05 mmol), 4-(trifluoromethyl)benzylamine (695 µL, 6.1 mmol) and 5 mL EtOH. The product was purified by column chromatography and isolated as white solid in 67% yield (1.70 g, 3.38 mmol).

Spectroscopic data are in accordance with literature [16].

^{1}H NMR (400 MHz, $CDCl_3$) δ = 7.94 (s, 2H), 7.79 (dt, J=7.0, 1.6, 1.1, 2H), 7.64 (d, J=6.9, 4H), 7.61 – 7.43 (m, 9H), 7.36 (d, J=8.2, 2H), 6.64 (d, J=8.1, 2H), 5.84 (s, 2H).

^{13}C NMR (101 MHz, $CDCl_3$) δ = 157.5, 156.8, 138.0, 133.7, 132.6, 132.5, 131.2, 130.5 (q, J=32.8), 129.8, 129.3, 129.1, 128.2, 126.7, 126.7, 125.7 (q, J=3.8), 122.2 (q, J=272.5, 271.7).

^{19}F NMR (376 MHz, $CDCl_3$) δ = -62.81, -152.67 (minor, $^{10}BF_4^-$), -152.72 (major, $^{11}BF_4^-$).

HRMS: calculated for $C_{31}H_{23}F_3N$ $[M-BF_4^-]^+$ 466.1777; found 466.1783; Δ = 1.19 ppm.

2,4,6-Triphenyl-1-(4-methoxybenzyl)pyridin-1-ium tetrafluoroborate (S7)

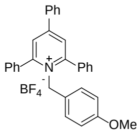

According to GP4 using 2,4,6-triphenylpyrylium tetrafluoroborate (1.94 g, 4.90 mmol), 4-methoxybenzylamine (767 µL, 5.9 mmol) and 20 mL EtOH at room temperature overnight. The product was isolated as white solid after precipitation from solution in 70% yield (1.50 g, 3.41 mmol).

Spectroscopic data are in accordance with literature [17].

^{1}H NMR (400 MHz, $CDCl_3$) δ = 7.90 (s, 2H), 7.79 (dt, J=6.8, 1.7, 1.2, 2H), 7.65 (d, J=6.5, 4H), 7.62 – 7.43 (m, 9H), 6.60 (dd, J=8.9, 1.9, 2H), 6.34 (d, J=8.7, 2H), 5.70 (s, 2H), 3.70 (s, 3H).

^{13}C NMR (101 MHz, $CDCl_3$) δ = 159.6, 157.6, 156.3, 133.9, 133.0, 132.5, 131.1, 129.9, 129.3, 129.2, 128.3, 127.9, 126.7, 126.1, 114.3, 58.0, 55.4.

^{19}F NMR (376 MHz, $CDCl_3$) δ = -152.26 (minor, $^{10}BF_4^-$), -152.31 (major, $^{11}BF_4^-$).

HRMS: calculated for $C_{31}H_{26}NO$ $[M-BF_4^-]^+$ 428.2009; found 428.2013; Δ = 1.03 ppm.

2,4,6-Triphenyl-1-(4-bromobenzyl)pyridin-1-ium tetrafluoroborate (S8)

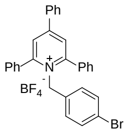

According to GP4 using 2,4,6-triphenylpyrylium tetrafluoroborate (1.50 g, 3.80 mmol), 4-bromobenzylamine hydrochloride (1.0 g, 4.6 mmol) and 3.8 mL EtOH at reflux. The product was isolated as white solid in 14% yield after precipitation from an ethanolic solution after purification by column chromatography (289.0 mg, 0.5 mmol).

Spectroscopic data are in accordance with literature [16].

^{1}H NMR (600 MHz, $CDCl_3$) δ = 7.95 (s, 2H), 7.85 – 7.77 (m, 2H), 7.65 (d, *J*=7.0, 4H), 7.59 (dd, *J*=8.3, 6.4, 1H), 7.56 – 7.45 (m, 8H), 7.23 (d, *J*=8.5, 2H), 6.36 (d, *J*=8.4, 2H), 5.74 (s, 2H).

^{13}C NMR (151 MHz, $CDCl_3$) δ = 157.7, 156.7, 133.8, 133.2, 132.7, 132.6, 132.1, 131.3, 130.0, 129.4, 129.2, 128.3, 128.1, 126.8, 122.5, 57.8.

^{19}F NMR (565 MHz, $CDCl_3$) δ = -152.92 (minor, $^{10}BF_4^-$), -152.97 (major, $^{11}BF_4^-$).

HRMS: calculated for $C_{30}H_{23}BrN$ $[M-BF_4^-]^+$ 476.1008; found 476.1013; Δ = 0.78 ppm.

2,4,6-Triphenyl-1-(2-methylbenzyl)pyridin-1-ium tetrafluoroborate (S9)

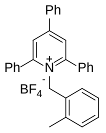

According to GP4 using 2,4,6-triphenylpyrylium tetrafluoroborate (1.50 g, 3.80 mmol), 2-methylbenzylamine (550.6 mg, 4.5 mmol) and 3.8 mL EtOH at reflux. The product was isolated as white solid in 58% yield after precipitation (1.09 g, 2.19 mmol).

^{1}H NMR (600 MHz, $CDCl_3$) δ = 7.95 (s, 2H), 7.88 – 7.78 (m, 2H), 7.64 – 7.56 (m, 5H), 7.53 (t, *J*=7.6, 6.6, 2H), 7.48 (t, *J*=7.5, 2H), 7.42 (t, *J*=7.6, 4H), 7.15 – 7.06 (m, 2H), 6.92 – 6.84 (m, 1H), 6.50 – 6.37 (m, 1H), 5.66 (s, 2H), 1.62 (s, 3H).

^{13}C NMR (151 MHz, $CDCl_3$) δ = 157.7, 156.4, 135.4, 133.9, 133.1, 132.6, 132.5, 131.0, 130.6, 129.9, 129.1, 129.0, 128.3, 128.3, 126.8, 126.5, 124.9, 56.1, 18.7.

^{19}F NMR (565 MHz, $CDCl_3$) δ = -153.07 (minor, $^{10}BF_4^-$), -153.13 (major, $^{11}BF_4^-$).

HRMS: calculated for $C_{31}H_{26}N$ $[M-BF_4^-]^+$ 412.2060; found 412.2065; Δ = 1.22 ppm.

2,4,6-Triphenyl-1-(3-methylbenzyl)pyridin-1-ium tetrafluoroborate (S10)

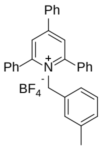

According to GP4 using 2,4,6-triphenylpyrylium tetrafluoroborate (577.0 mg, 1.46 mmol), 3-methylbenzylamine (211.0 mg, 1.75 mmol) and 1.5 mL EtOH at reflux. The product was isolated as white solid in 69% yield after precipitation (499.4 mg, 1.00 mmol).

^{1}H NMR (600 MHz, $CDCl_3$) δ = 7.93 (s, 2H), 7.85 – 7.77 (m, 2H), 7.64 (d, *J*=6.8, 4H), 7.60 – 7.52 (m, 3H), 7.50 (d, *J*=7.2, 2H), 7.47 (t, *J*=7.3, 4H), 7.00 (t, *J*=7.6, 1H), 6.96 (d, *J*=7.6, 1H), 6.28 (d, *J*=7.5, 1H), 6.18 (s, 1H), 5.72 (s, 2H), 2.12 (s, 3H).

^{13}C NMR (151 MHz, $CDCl_3$) δ = 157.7, 156.3, 138.8, 134.2, 133.9, 132.9, 132.5, 131.0, 129.9, 129.3, 129.1, 128.8, 128.3, 127.5, 126.7, 123.3, 58.4, 21.3.

^{19}F NMR (376 MHz, $CDCl_3$) δ = -153.13 (minor, $^{10}BF_4^-$), -153.18 (major, $^{11}BF_4^-$).

HRMS: calculated for $C_{31}H_{26}N$ $[M-BF_4^-]^+$ 412.2060; found 412.2066; Δ = 1.67 ppm.

2,4,6-Triphenyl-1-(4-methylbenzyl)pyridin-1-ium tetrafluoroborate (S11)

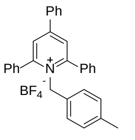

According to GP4 using 2,4,6-triphenylpyrylium tetrafluoroborate (1.0 g, 2.52 mmol), 4-methylbenzylamine (367.0 mg, 3.04 mmol) and 2.5 mL EtOH at reflux. The product was isolated as white solid in 47% yield after precipitation (590.4 mg, 1.18 mmol).

Spectroscopic data are in accordance with literature [15].

^{1}H NMR (600 MHz, $CDCl_3$) δ = 7.93 (s, 2H), 7.81 (d, *J*=7.4, 2H), 7.64 (d, *J*=5.4, 4H), 7.60 – 7.42 (m, 9H), 6.90 (d, *J*=7.8, 2H), 6.33 (d, *J*=7.9, 2H), 5.72 (s, 2H), 2.23 (s, 3H).

^{13}C NMR (151 MHz, $CDCl_3$) δ = 157.7, 156.3, 138.3, 134.0, 132.9, 132.5, 131.3, 131.1, 129.9, 129.6, 129.3, 128.3, 126.7, 126.3, 58.3, 21.2.

^{19}F NMR (565 MHz, CDCl3) δ = -153.08 (minor, $^{10}BF_4^-$), -153.13 (major, $^{11}BF_4^-$).

HRMS: calculated for $C_{31}H_{26}N$ $[M-BF_4^-]^+$ 412.2060; found 412.2065; Δ = 1.40 ppm.

1-(Naphthalen-2-ylmethyl)-2,4,6-triphenylpyridin-1-ium tetrafluoroborate (S12)

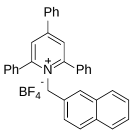

According to GP4 using 2,4,6-triphenylpyrylium tetrafluoroborate (1.5 g, 3.81 mmol), naphthalen-2-ylmethanamine (716.0 mg, 4.55 mmol) and 4.0 mL EtOH at reflux. The product was isolated as white solid in 33% yield after column chromatography (674.0 mg, 1.26 mmol).

Spectroscopic data are in accordance with literature [15].

^{1}H NMR (600 MHz, $CDCl_3$) δ = 7.98 (s, 2H), 7.86 – 7.79 (m, 2H), 7.73 – 7.69 (m, 2H), 7.62 (dd, *J*=5.9, 1.9, 4H), 7.59 – 7.50 (m, 2H), 7.44 – 7.34 (m, 3H), 7.32 – 7.19 (m, 8H), 6.68 – 6.62 (m, 1H), 6.18 (s, 2H).

^{13}C NMR (151 MHz, $CDCl_3$) δ = 157.8, 156.4, 133.8, 133.1, 132.4, 132.3, 130.9, 130.8, 129.8, 129.3, 129.2, 129.1, 129.0, 128.9, 128.8, 128.5, 128.2, 127.0, 126.7, 126.5, 124.7, 123.1, 121.9, 55.9.

^{19}F NMR (565 MHz, CDCl3) δ = -152.77 (minor, $^{10}BF_4^-$), -152.82 (major, $^{11}BF_4^-$).

HRMS: calculated for $C_{34}H_{26}N$ $[M\text{-}BF_4^-]^+$ 448.2060; found 412.2068; Δ = 1.94 ppm.

2,4,6-Triphenyl-1-(pyridin-2-ylmethyl)pyridin-1-ium tetrafluoroborate (S13)

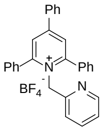

According to GP4 using 2,4,6-triphenylpyrylium tetrafluoroborate (1.0 g, 2.5 mmol), 2-picoylamine (273.0 mg, 2.5 mmol) and 2.5 mL EtOH at reflux. The product was isolated as golden foam in 73% yield after column chromatography (905.0 mg, 1.9 mmol).

Spectroscopic data are in accordance with literature [13].

^{1}H NMR (600 MHz, $CDCl_3$) δ = 8.44 (ddd, *J*=4.9, 1.6, 0.9, 1H), 7.95 (s, 2H), 7.86 – 7.80 (m, 2H), 7.65 – 7.53 (m, 5H), 7.49 – 7.44 (m, 2H), 7.43 – 7.35 (m, 5H), 7.26 (s, 1H), 7.20 – 7.12 (m, 2H), 6.55 (d, *J*=7.9, 1H), 5.80 (s, 2H).

^{13}C NMR (151 MHz, $CDCl_3$) δ = 157.7, 156.1, 153.4, 149.1, 137.1, 134.2, 133.1, 132.3, 130.9, 129.9, 129.0, 128.3, 126.3, 123.2, 122.1, 59.1.

^{19}F NMR (565 MHz, CDCl3) δ = -153.23 (minor, $^{10}BF_4^-$), -153.28 (major, $^{11}BF_4^-$).

2,4,6-Triphenyl-1-(pyridin-3-ylmethyl)pyridin-1-ium tetrafluoroborate (S14)

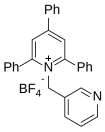

According to GP4 using 2,4,6-triphenylpyrylium tetrafluoroborate (2.0 g, 2.5 mmol), 3-picoylamine (273.0 mg, 5.05 mmol) and 5.0 mL EtOH at reflux. The product was isolated as off-white solid in 90% yield after precipitation**

Spectroscopic data are in accordance with literature [13].

^{1}H NMR (400 MHz, $CDCl_3$) δ = 8.36 – 8.32 (m, 1H), 7.90 (s, 2H), 7.74 (d, *J*=7.0, 2H), 7.69 – 7.58 (m, 5H), 7.56 – 7.29 (m, 8H), 7.18 (d, *J*=8.4, 1H), 6.98 (dd, *J*=7.8, 4.9, 1H), 6.79 (d, *J*=8.0, 1H), 5.78 (s, 2H).

^{13}C NMR (101 MHz, $CDCl_3$) δ = 157.6, 157.0, 149.7, 147.5, 134.7, 133.8, 132.7, 132.7, 131.5, 130.1, 130.0, 129.6, 129.2, 128.3, 126.9, 123.8, 56.1.

^{19}F NMR (376 MHz, CDCl3) δ = -152.75 (minor, $^{10}BF_4^-$), -152.80 (major, $^{11}BF_4^-$).

** The material was used without further purification, but was not analytically pure

1-Allyl-2,4,6-triphenylpyridin-1-ium tetrafluoroborate tetrafluoroborate (S15)

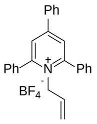

According to GP4 using 2,4,6-triphenylpyrylium tetrafluoroborate (3.0 g, 7.6 mmol), allylamine (1.4 g, 24.2 mmol) and 7.5 mL EtOH at reflux. The product was isolated as off-white solid in 71% yield (2.3 g, 5.3 mmol) after precipitation from a pink solution after the addition of diethyl ether, and facilitating precipitation over weekend at −20 °C.

Spectroscopic data are in accordance with literature [16].

^{1}H NMR (600 MHz, $CDCl_3$) δ = 7.86 (s, 2H), 7.78 – 7.72 (m, 6H), 7.62 – 7.52 (m, 7H), 7.49 (t, *J*=7.5, 2H), 5.52 (ddt, *J*=15.8, 10.6, 5.4, 1H), 5.17 – 4.96 (m, 3H), 4.51 (d, *J*=17.1, 1H).

^{13}C NMR (151 MHz, $CDCl_3$) δ = 157.1, 156.2, 134.0, 132.6, 132.3, 131.2, 130.4, 129.8, 129.3, 129.2, 128.3, 126.7, 120.3, 57.1.

^{19}F NMR (565 MHz, CDCl3) δ = -153.20 (minor, $^{10}BF_4^-$), -153.25 (major, $^{11}BF_4^-$).

HRMS: calculated for $C_{26}H_{22}N$ $[M\text{-}BF_4^-]^+$ 348.1747; found 348.1752; Δ = 1.64 ppm.

8. 2,4,6-Triphenylpyrylium tetrafluoroborate (S16)

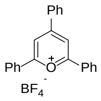

2,4,6-Triphenylpyrylium tetrafluoroborate was prepared according to a modified literature-known procedure [18]. HBF_4 etherate (23 mL, 167.60 mmol, 2.0 equiv) was added dropwise to a solution of acetophenone (10.0 g, 83.23 mmol, 1.0 equiv) and chalcone (34.40 g, 165.18 mmol, 1.98 equiv) in DCE (60 mL). After addition, the black solution was refluxed for 1.5 h. The solution was cooled to rt and Et_2O was added to precipitate the product. The yellow product was filtered off, washed with Et2O, and dried under reduced pressure at 40 °C to afford 72% of pure material (23.8 g, 60.07 mmol).

Spectroscopic data are in accordance with literature [19].

^{1}H NMR (400 MHz, DMSO) δ = 9.18 (s, 2H), 8.70 – 8.55 (m, 6H), 7.94 – 7.84 (m, 3H), 7.83 – 7.76 (m, 6H).

^{13}C NMR (101 MHz, DMSO) δ = 170.1, 165.1, 135.2, 135.0, 132.5, 130.1, 129.9, 129.9, 129.2, 128.8, 115.2.

^{19}F NMR (376 MHz, DMSO) δ = -148.24 (minor, $^{10}BF_4$), -148.30 (major, $^{11}BF_4^-$).

9. References

1. Wang, T.; Schrempp, M.; Berndhauser, A.; Schiemann, O.; Menche, D. *Org. Lett.* **2015**, *17*, 3982-5.
2. Barham, J. P.; John, M. P.; Murphy, J. A. *Beilstein J. Org. Chem.* **2014**, *10*, 2981-8.
3. Tian, H.; Xu, W.; Liu, Y.; Wang, Q. *Chem. Commun.* **2019**, *55*, 14813-14816.
4. Möhlmann, L.; Blechert, S. *Adv. Synth. Catal.* **2014**, *356*, 2825-2829.
5. Lewis, F. D.; Bassani, D. M.; Burch, E. L.; Cohen, B. E.; Engleman, J. A.; Reddy, G. D.; Schneider, S.; Jaeger, W.; Gedeck, P.; Gahr, M. *J. Am. Chem. Soc.* **1995**, *117*, 660-669.
6. Alagiri, K.; Kumara, G. S.; Prabhu, K. R. *Chem. Commun.* **2011**, *47*, 11787-9.
7. Basle, O.; Li, C. J. *Org. Lett.* **2008**, *10*, 3661-3.
8. Brzozowski, M.; Forni, J. A.; Paul Savage, G.; Polyzos, A. *Chem. Commun.* **2015**, *51*, 334-7.
9. Ueda, H.; Yoshida, K.; Tokuyama, H. *Org. Lett.* **2014**, *16*, 4194-4197.
10. Xie, W.; Liu, N.; Gong, B.; Ning, S.; Che, X.; Cui, L.; Xiang, J. *Eur. J. Org. Chem.* **2019**, *2019*, 2498-2501.
11. Kurouchi, H.; Kawamoto, K.; Sugimoto, H.; Nakamura, S.; Otani, Y.; Ohwada, T. *J. Org. Chem.* **2012**, *77*, 9313-9328.
12. Lahm, G.; Opatz, T. *Org. Lett.* **2014**, *16*, 4201-3.
13. Liao, J.; Guan, W.; Boscoe, B. P.; Tucker, J. W.; Tomlin, J. W.; Garnsey, M. R.; Watson, M. P. *Org. Lett.* **2018**, *20*, 3030-3033.
14. Klauck, F. J. R.; James, M. J.; Glorius, F. *Angew. Chem. Int. Ed.* **2017**, *56*, 12336-12339.
15. Klauck, F. J. R.; Yoon, H.; James, M. J.; Lautens, M.; Glorius, F. *ACS Catal.* **2019**, *9*, 236-241.
16. Ociepa, M.; Turkowska, J.; Gryko, D. *ACS Catal.* **2018**, *8*, 11362-11367.
17. Pitzer, L.; Schäfers, F.; Glorius, F. *Angew. Chem., Int. Ed.* **2019**, *58*, 8572-8576.
18. Baldoví, M. V.; García, H.; Miranda, M. A.; Primo, J. *Monatsh. Chem.* **1990**, *121*, 371-375.
19. Shen, J.; Li, N.; Yu, Y.; Ma, C. *Org. Lett.* **2019**, *21*, 7179-7183.

10. Spectra

1-Benzyl-2-phenyl-1,2,3,4-tetrahydroisoquinoline (3)

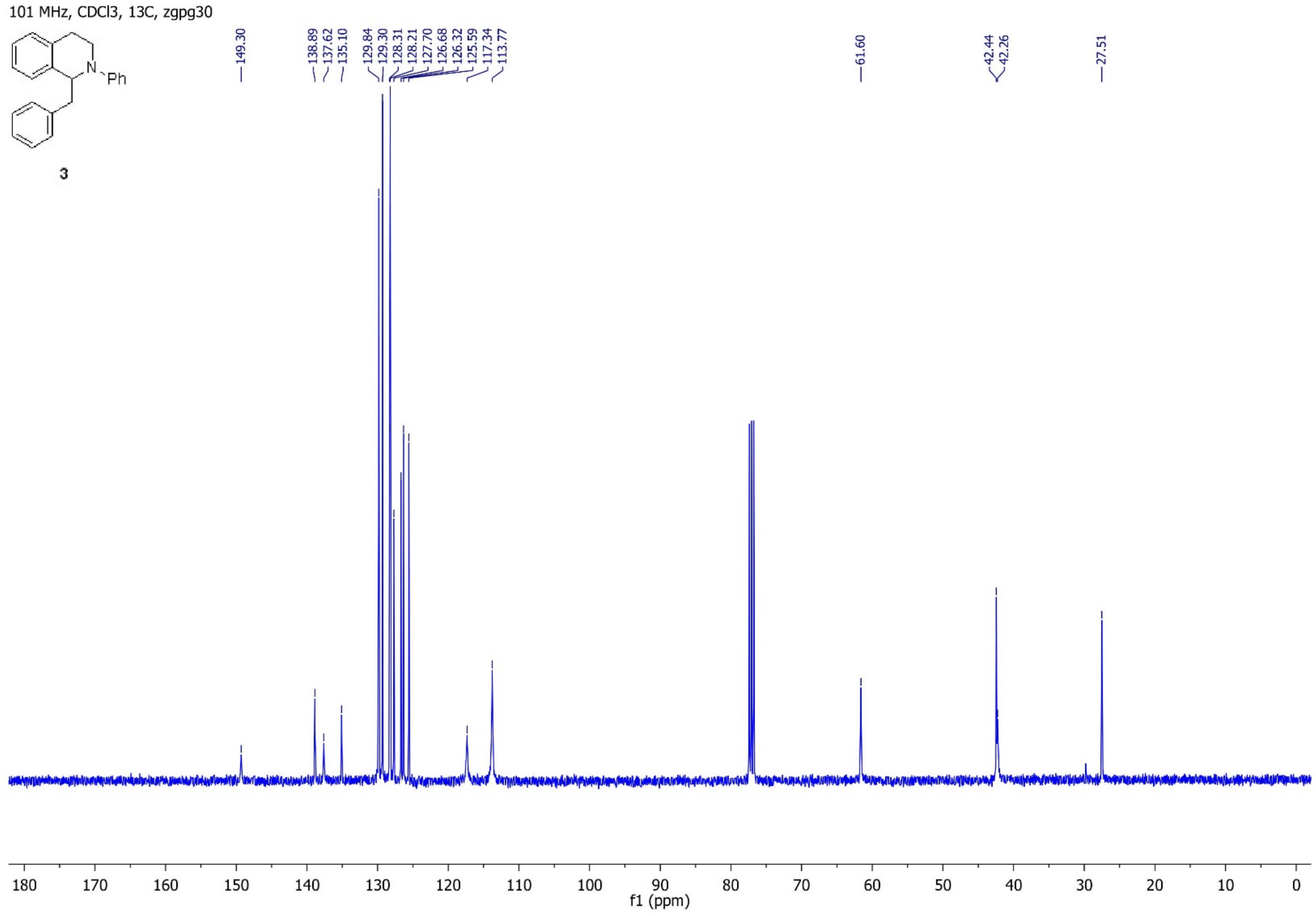

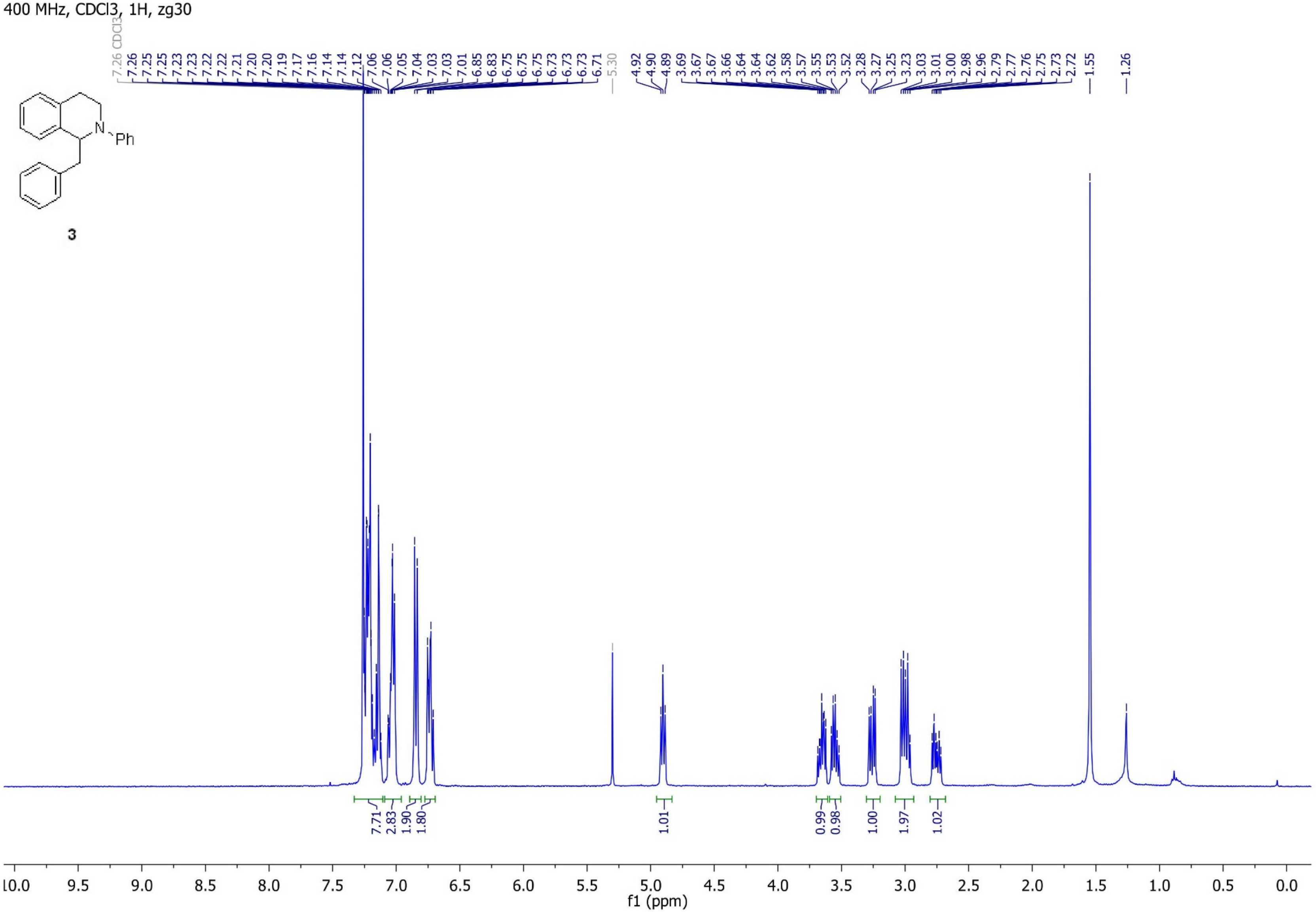
400 MHz, CDCl3, 1H, zg30
7.26 CDCl3
7.26 7.25 7.25 7.23 7.23 7.22 7.22 7.21 7.20 7.20 7.19 7.17 7.16 7.14 7.14 7.12 7.06 7.06 7.05 7.04 7.03 7.03 7.01 6.85 6.83 6.75 6.75 6.75 6.73 6.73 6.73 6.71
5.30
4.92 4.90 4.89 3.69 3.67 3.67 3.66 3.64 3.64 3.62 3.58 3.57 3.55 3.53 3.52 3.28 3.27 3.25 3.23 3.03 3.01 3.00 2.98 2.96 2.79 2.77 2.76 2.75 2.73 2.72
1.55
1.26
N
Ph
3
7.71 2.83 1.90 1.80 1.01 0.99 0.98 1.00 1.97 1.02
10.0 9.5 9.0 8.5 8.0 7.5 7.0 6.5 6.0 5.5 5.0 4.5 4.0 3.5 3.0 2.5 2.0 1.5 1.0 0.5 0.0
f1 (ppm)

1-(2-Methylbenzyl)-2-phenyl-1,2,3,4-tetrahydroisoquinoline (4)

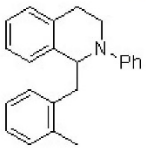

4

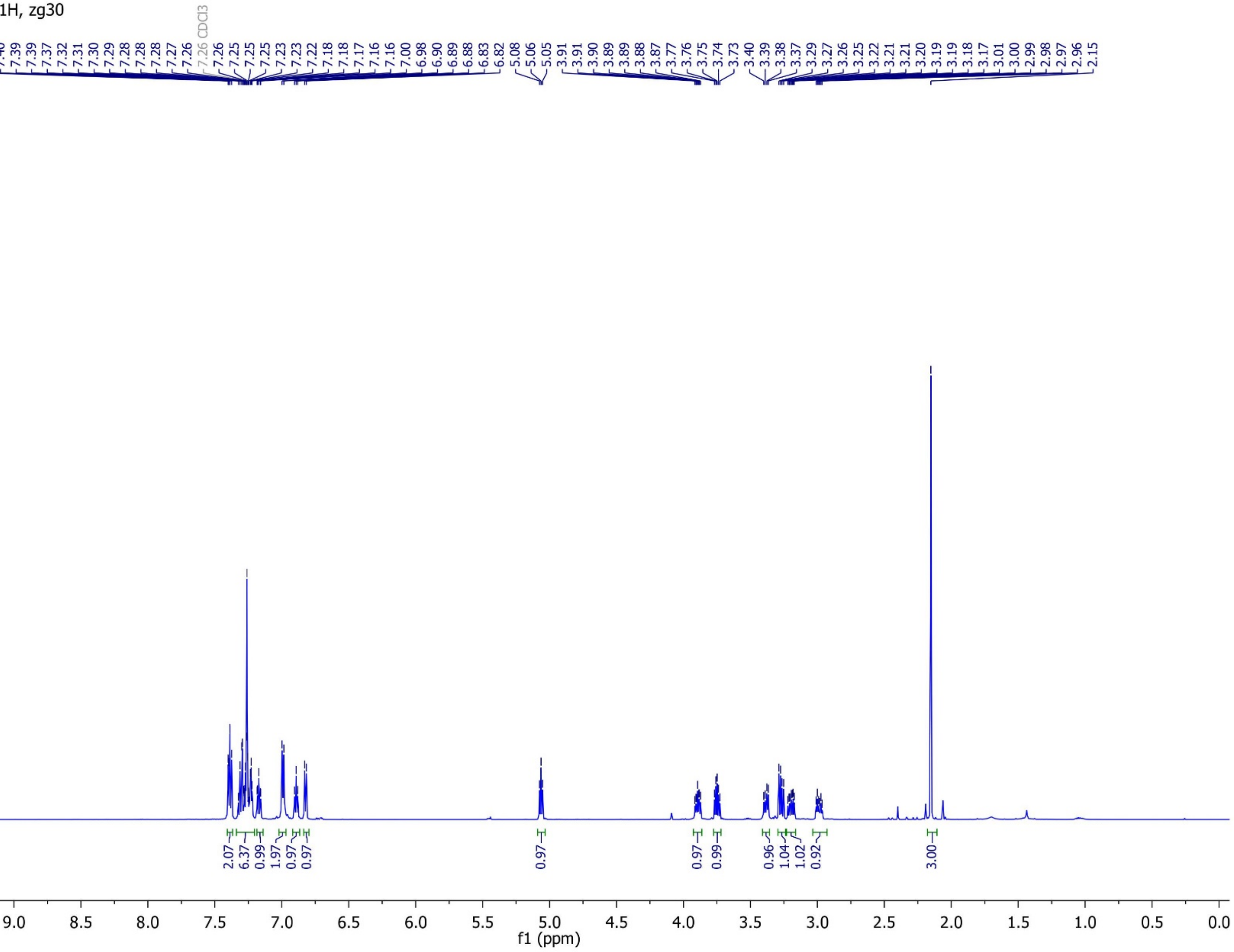

151 MHz, CDCl3, 13C, deptqgpsp

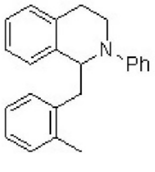

4

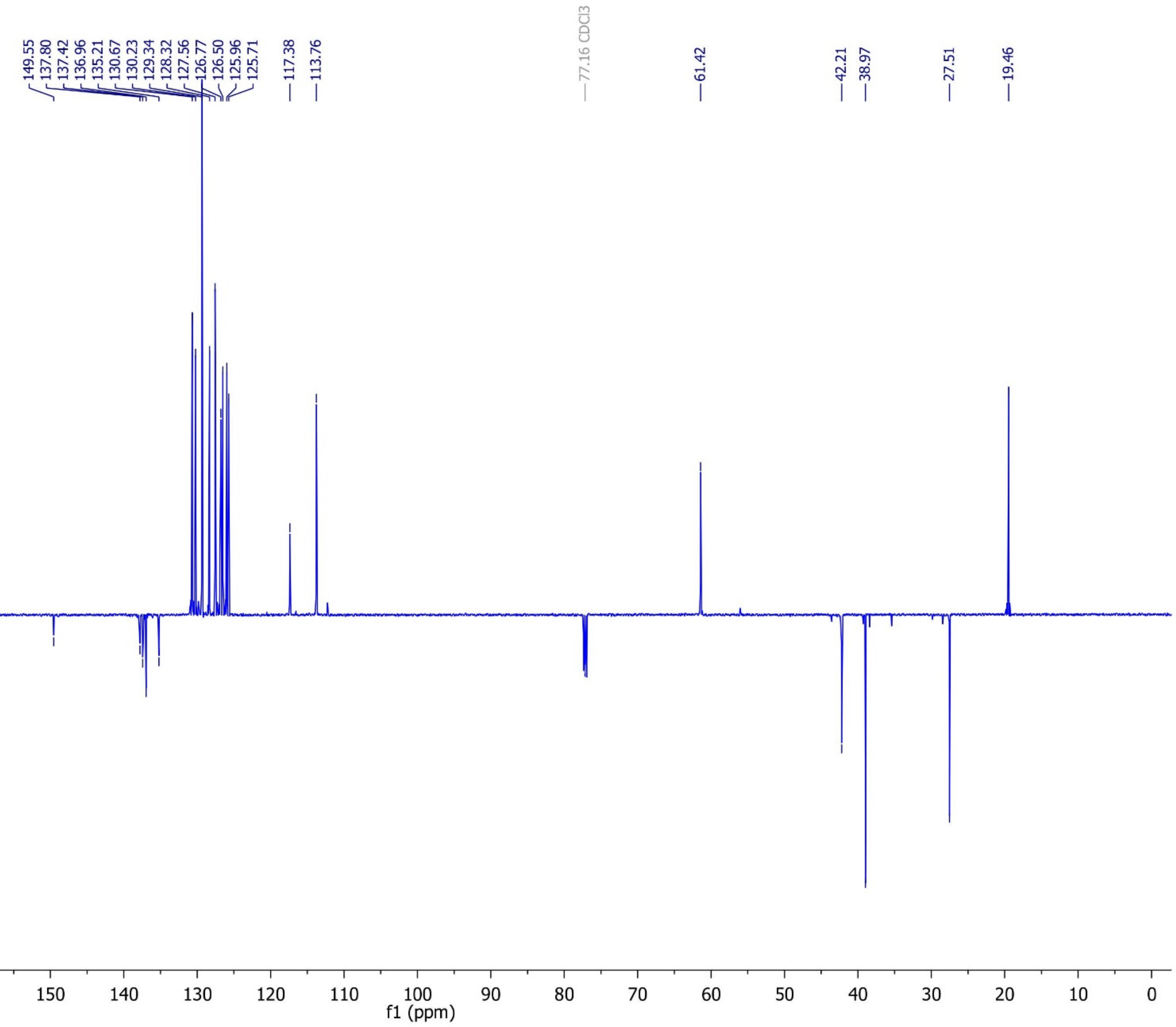

1-(3-Methylbenzyl)-2-phenyl-1,2,3,4-tetrahydroisoquinoline (5)

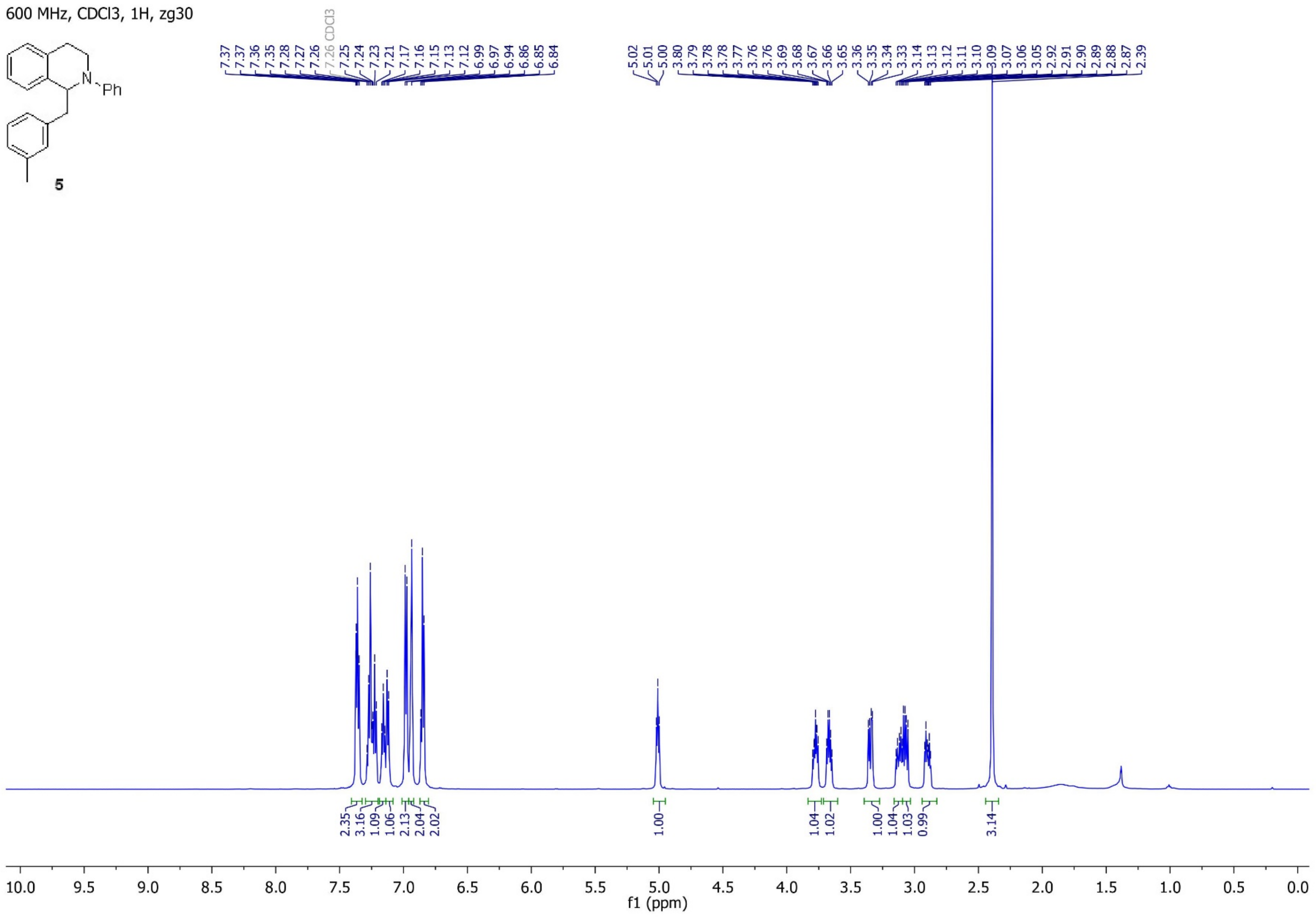

151 MHz, CDCl3, 13C, deptqgpsp

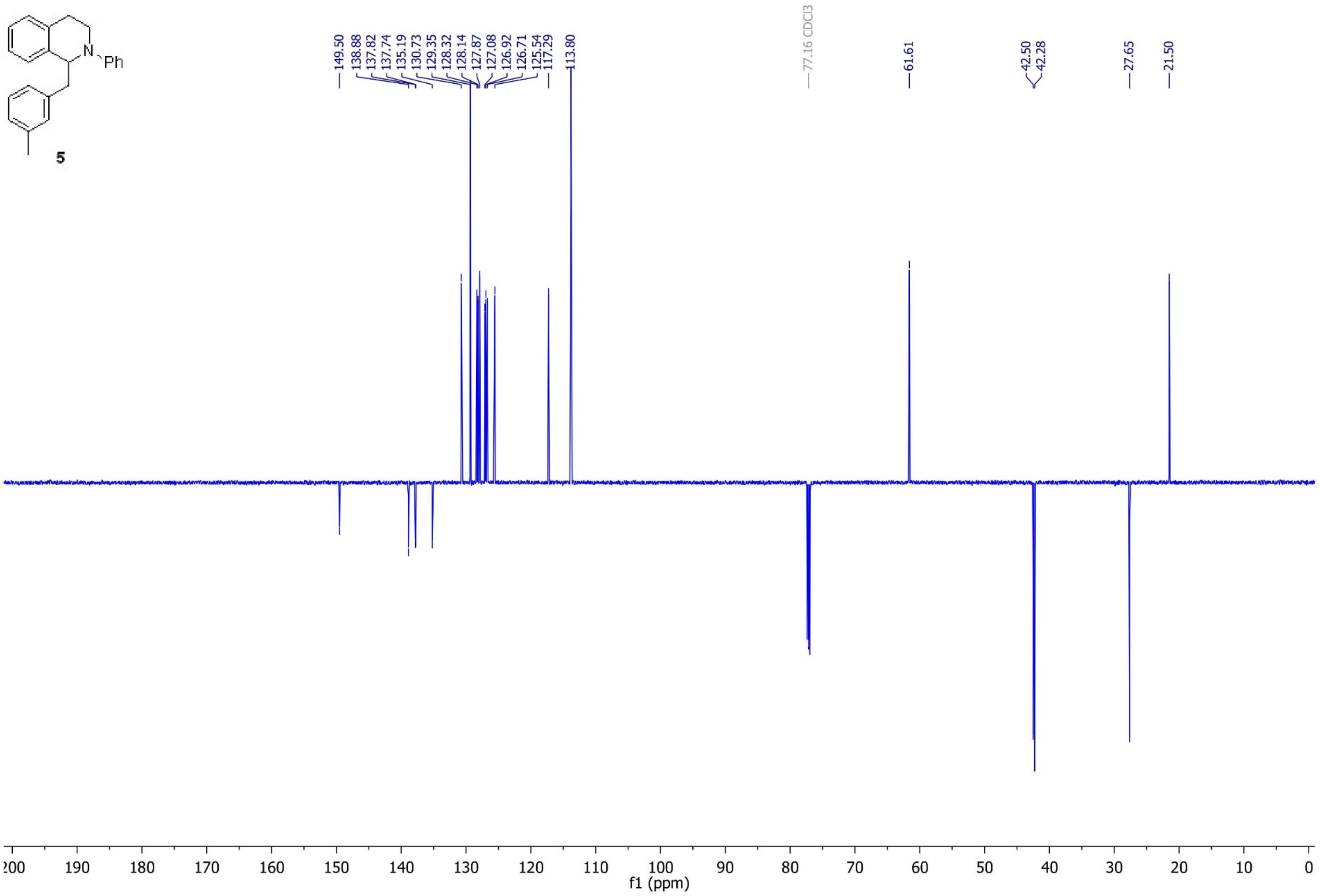

1-(4-Methylbenzyl)-2-phenyl-1,2,3,4-tetrahydroisoquinoline (6)

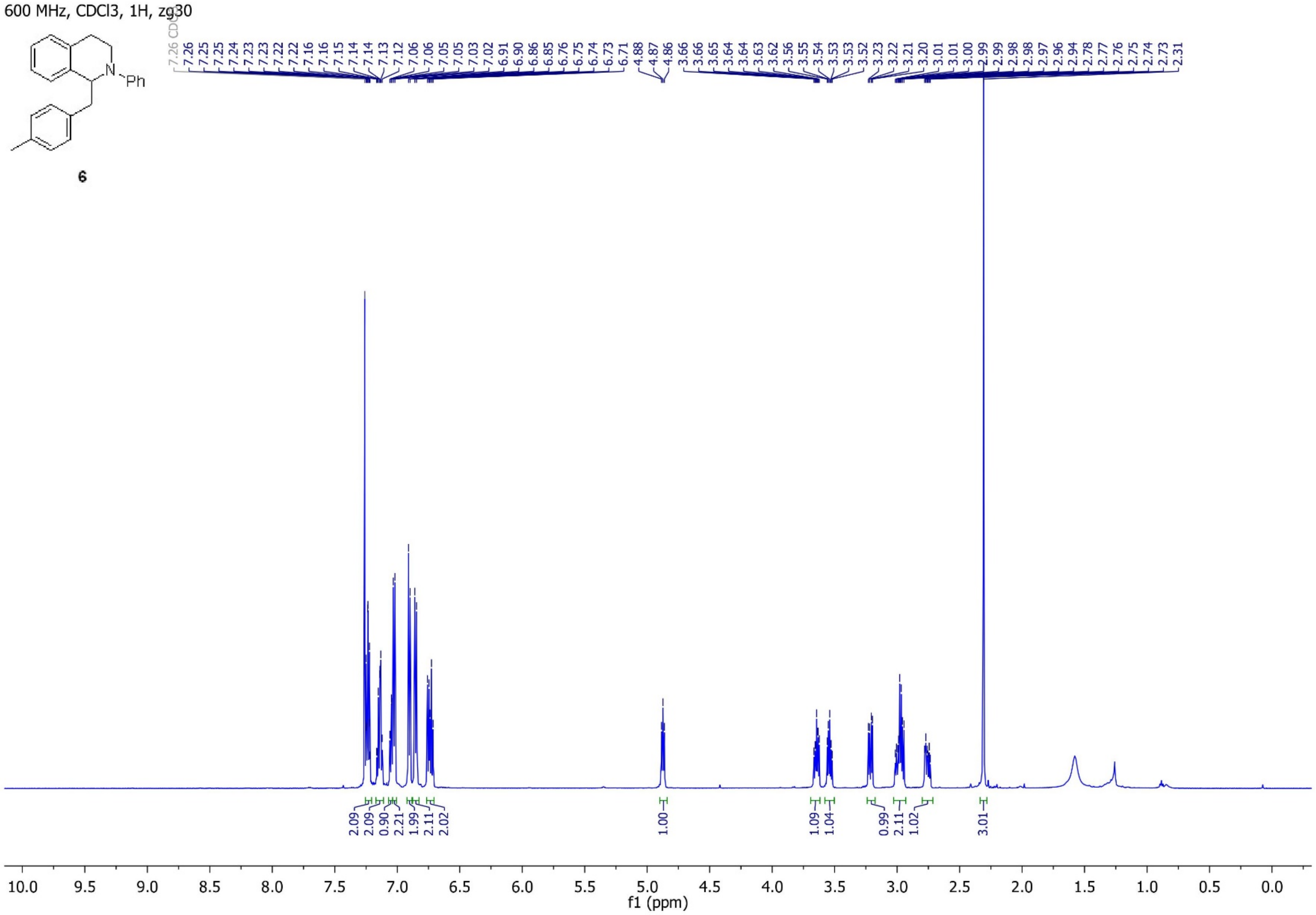

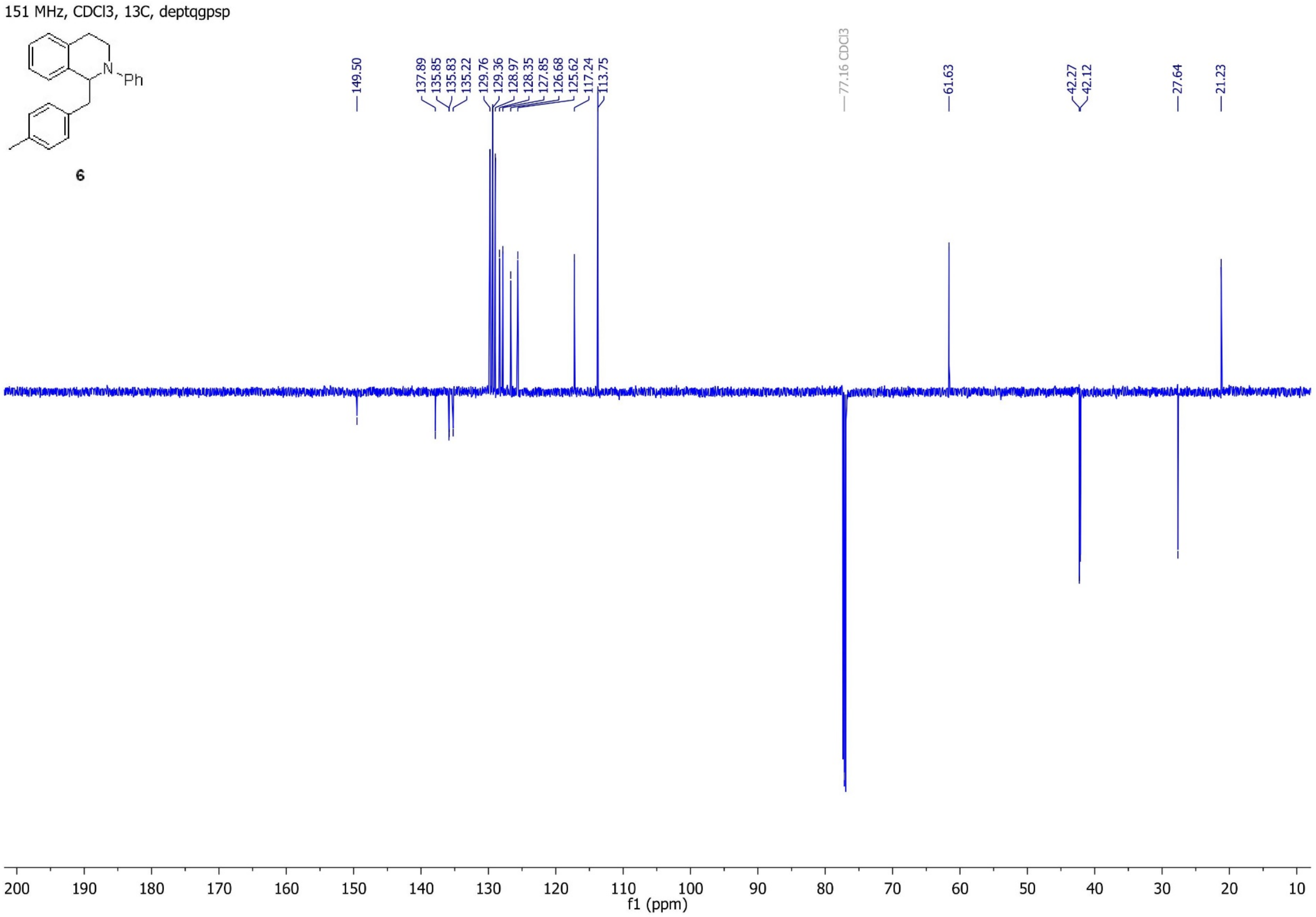

151 MHz, CDCl3, 13C, deptqgpsp
N
Ph
6
149.50
137.89
135.85
135.83
135.22
129.76
129.36
128.97
128.35
127.85
126.68
125.62
117.24
113.75
77.16 CDCl3
61.63
42.27
42.12
27.64
21.23
200
190
180
170
160
150
140
130
120
110
100
90
80
70
60
50
40
30
20
10
f1 (ppm)

1-(Naphthalen-2-ylmethyl)-2-phenyl-1,2,3,4-tetrahydroisoquinoline (7)

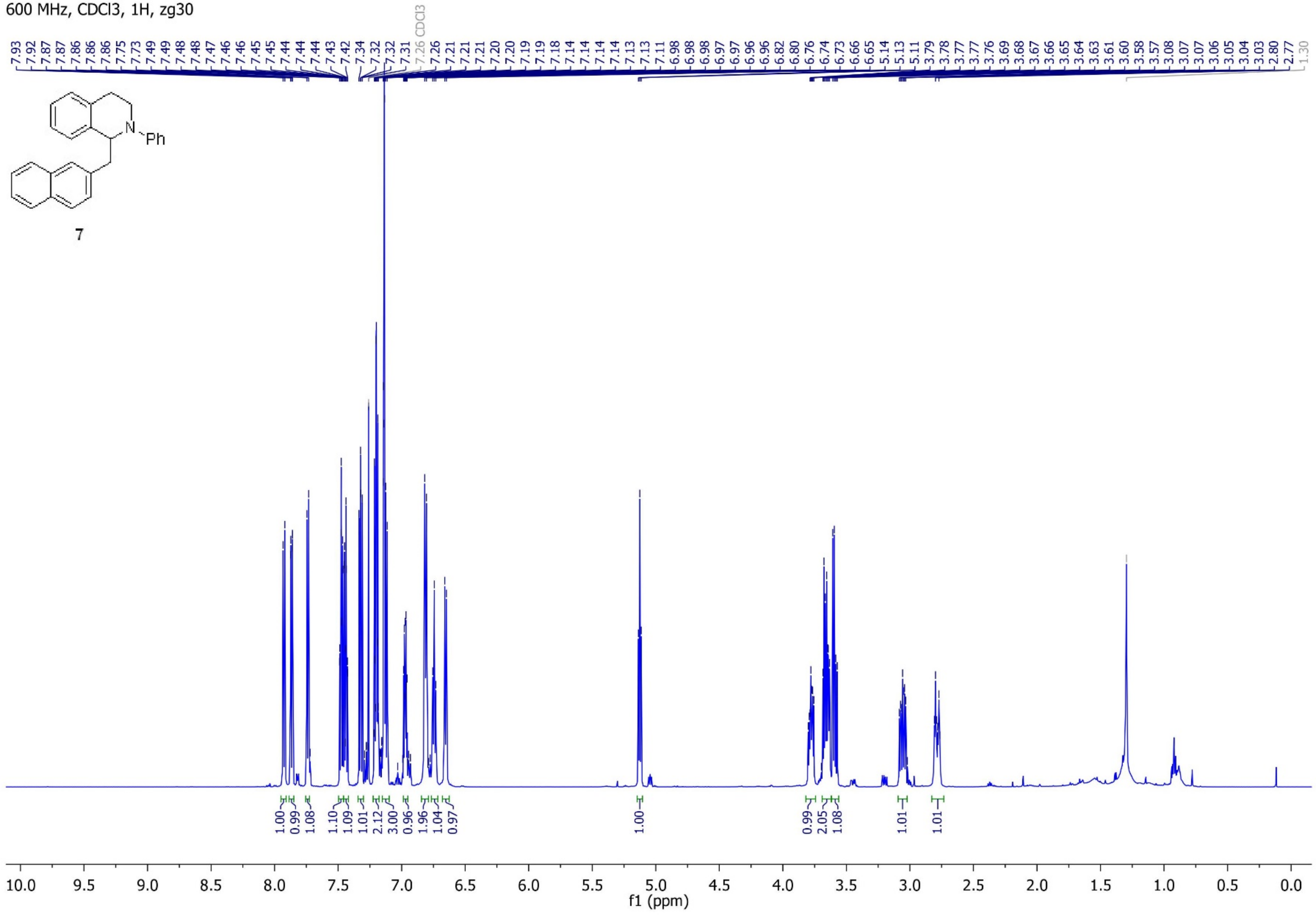

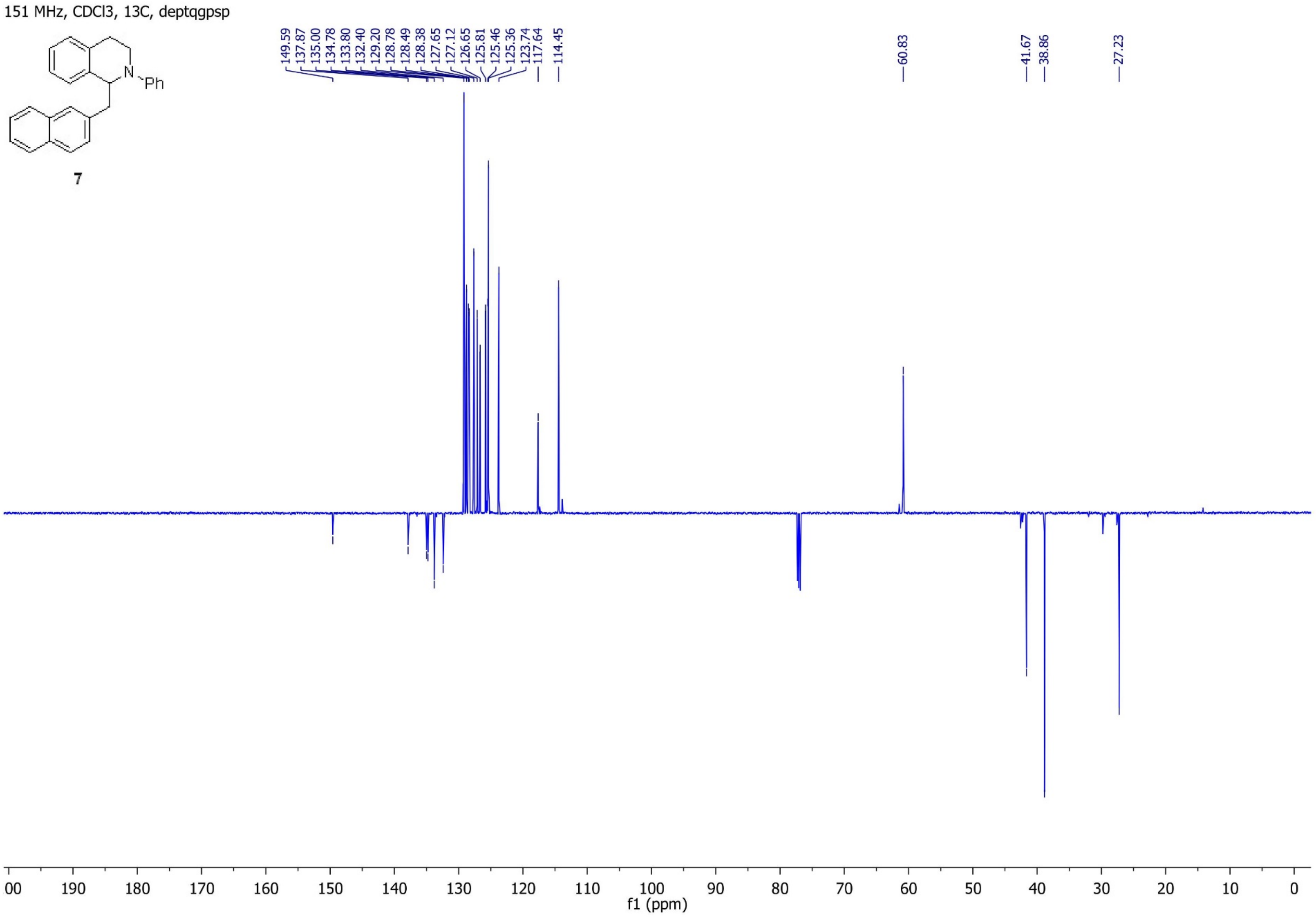
151 MHz, CDCl3, 13C, deptqgpsp
N
Ph
7
149.59
137.87
135.00
134.78
133.80
132.40
129.20
128.78
128.49
128.38
127.65
127.12
126.65
125.81
125.46
125.36
123.74
117.64
114.45
60.83
41.67
38.86
27.23
00 190 180 170 160 150 140 130 120 110 100 90 80 70 60 50 40 30 20 10 0
f1 (ppm)

1-(4-Bromobenzyl)-2-phenyl-1,2,3,4-tetrahydroisoquinoline (8)

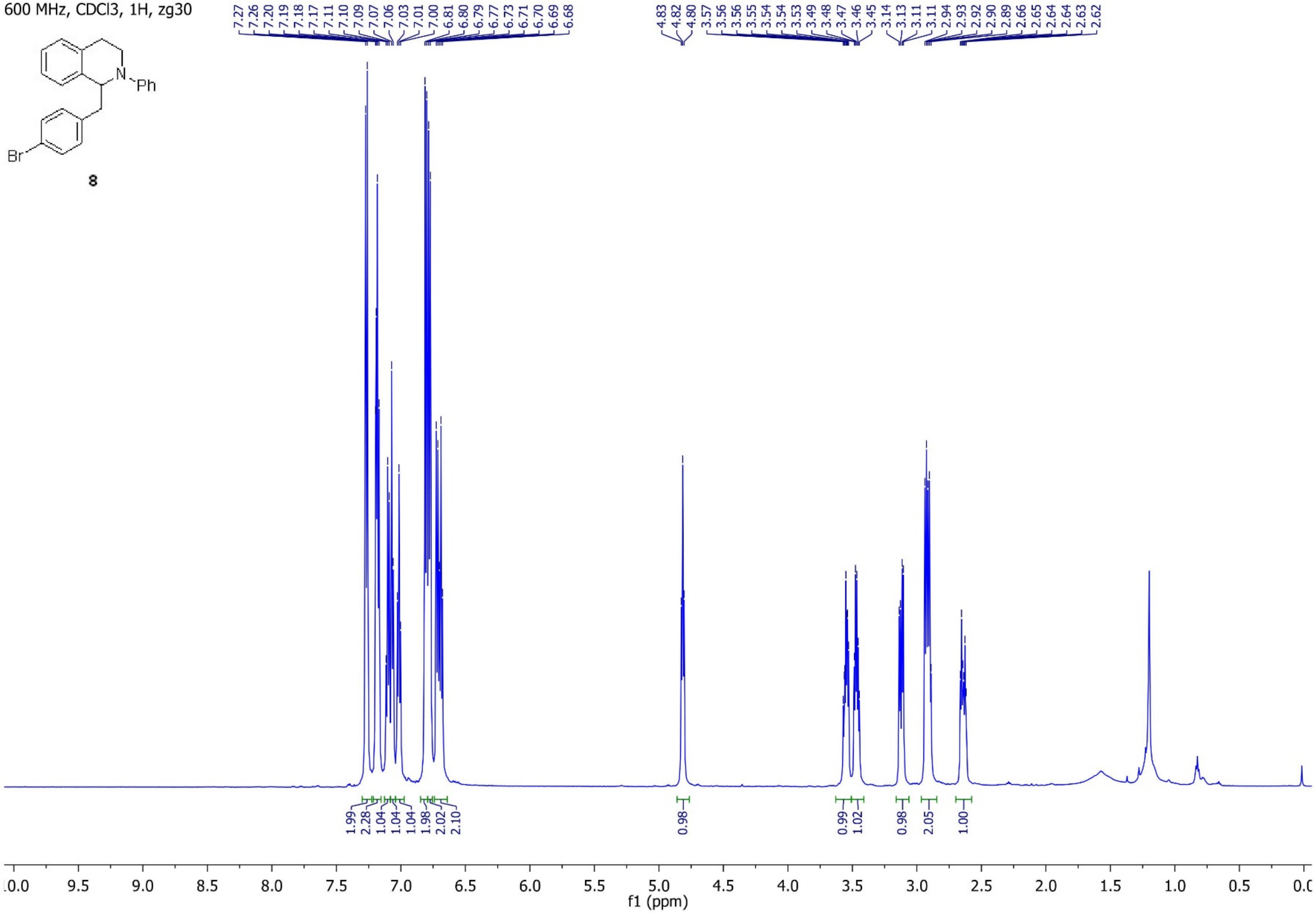

151 MHz, CDCl3, 13C, deptqgpsp

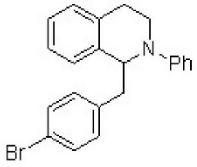

8

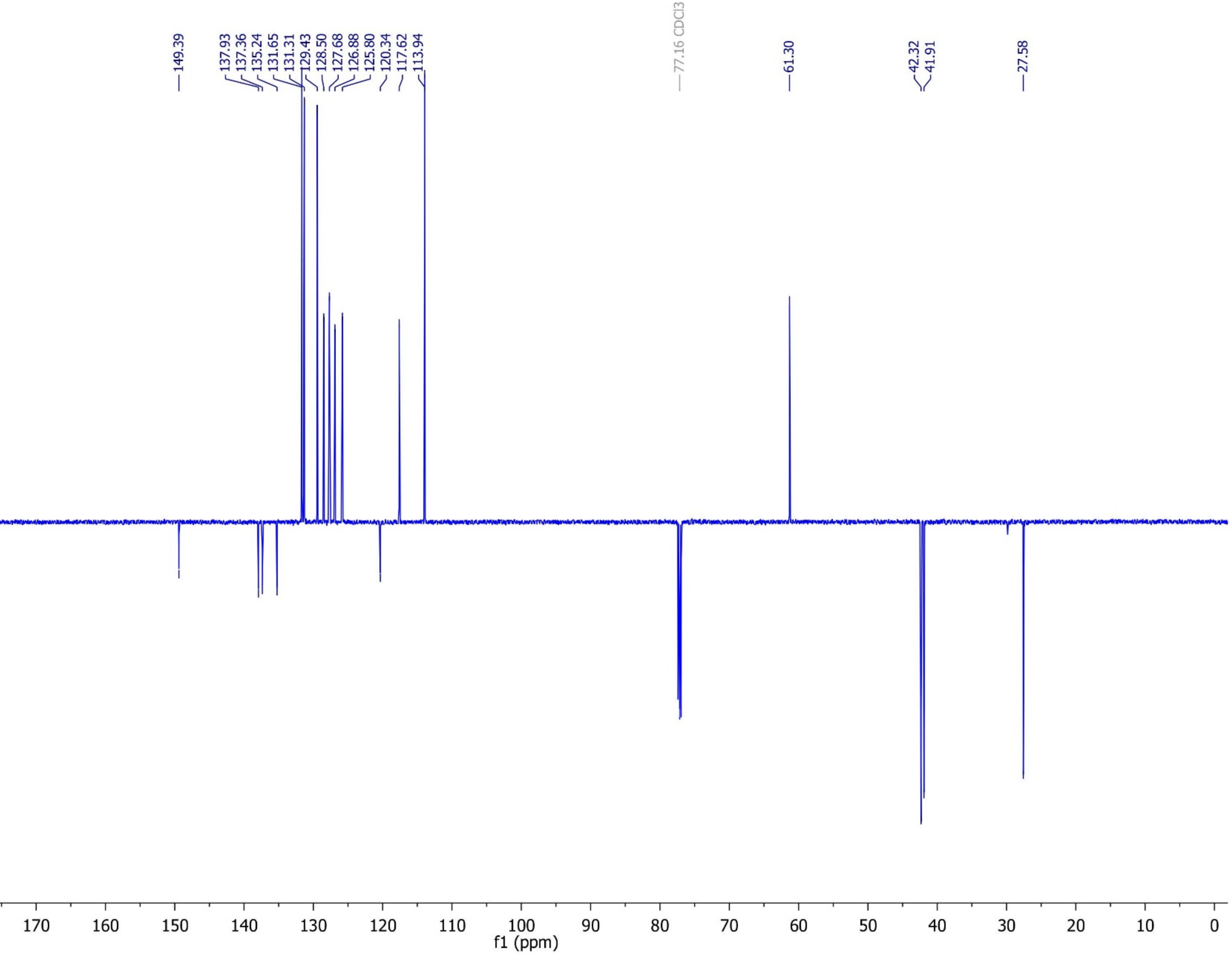

1-(4-Fluorobenzyl)-2-phenyl-1,2,3,4-tetrahydroisoquinoline (9)

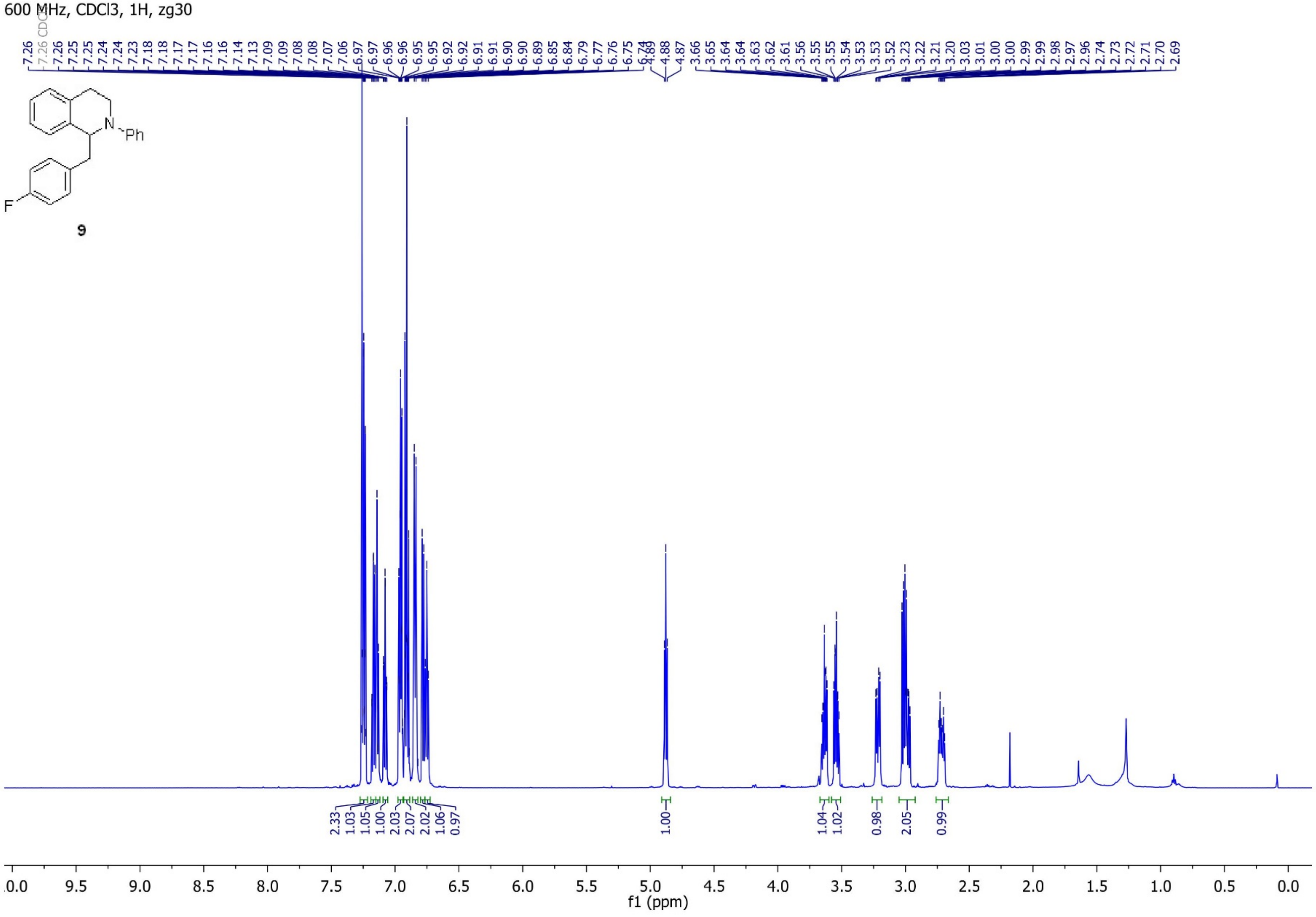

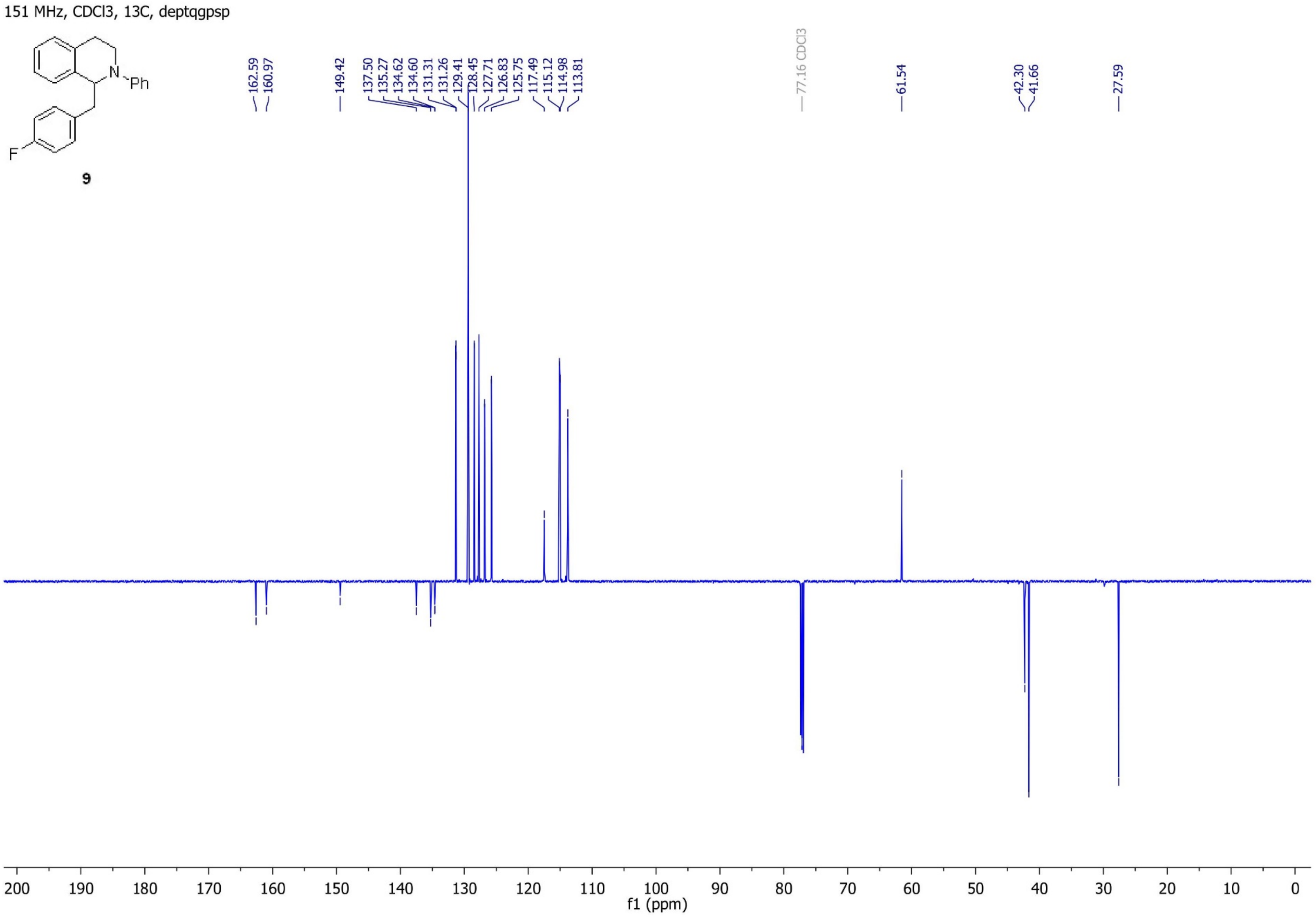
151 MHz, CDCl3, 13C, deptqgpsp
N
Ph
F
9
162.59
160.97
149.42
137.50
135.27
134.62
134.60
131.31
131.26
129.41
128.45
127.71
126.83
125.75
117.49
115.12
114.98
113.81
77.16 CDCl3
61.54
42.30
41.66
27.59
200
190
180
170
160
150
140
130
120
110
100
90
80
70
60
50
40
30
20
10
0
f1 (ppm)

565 MHz, CDCl3, 19F, zgflqn

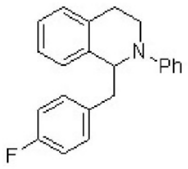

9

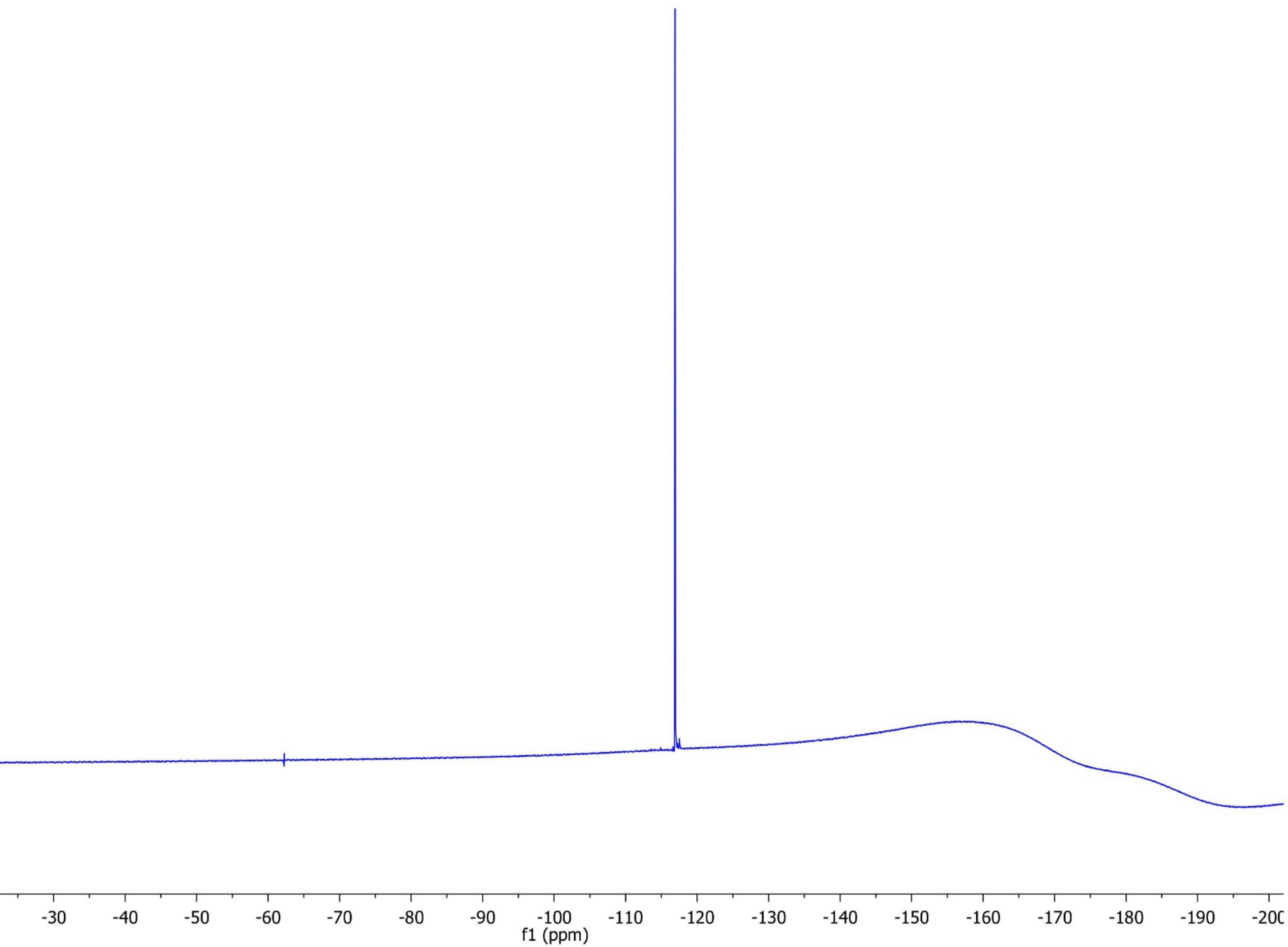

2-Phenyl-1-(4-(trifluoromethyl)benzyl)-1,2,3,4-tetrahydroisoquinoline (10)

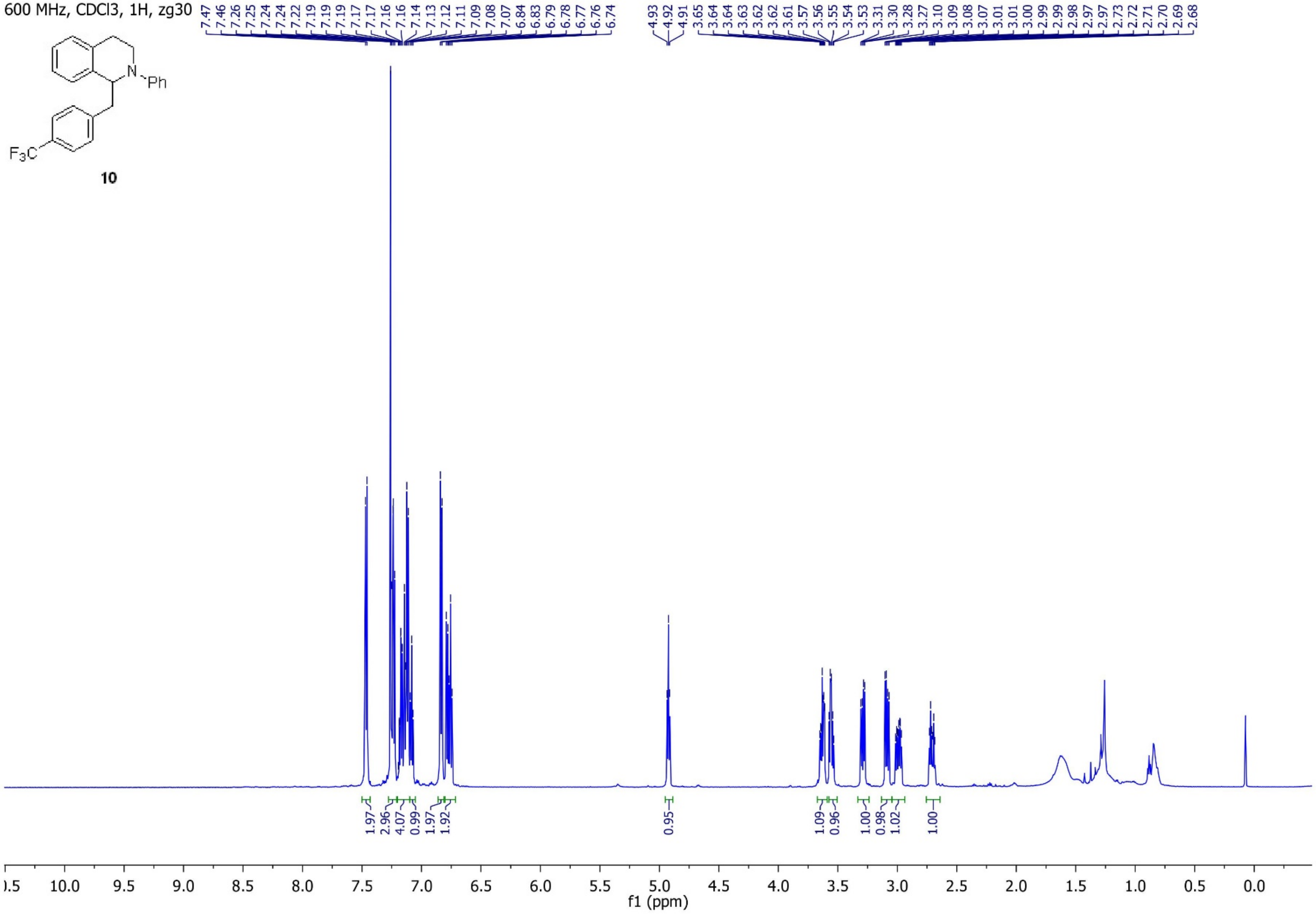

151 MHz, CDCl3, 13C, deptqgpsp

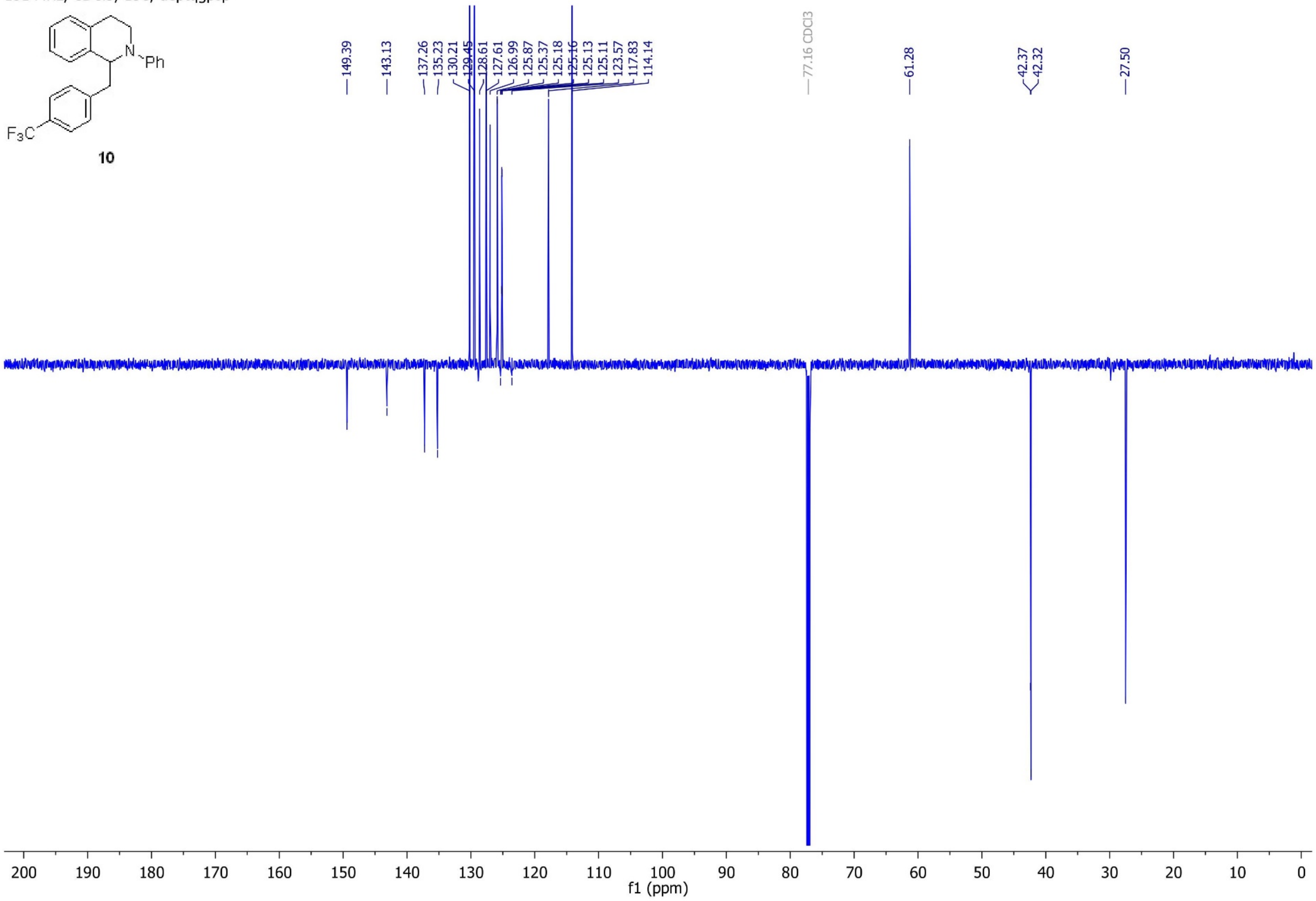

10

565 MHz, CDCl3, 19F, zgflqn

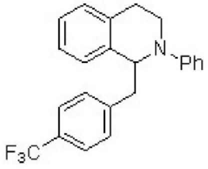

10

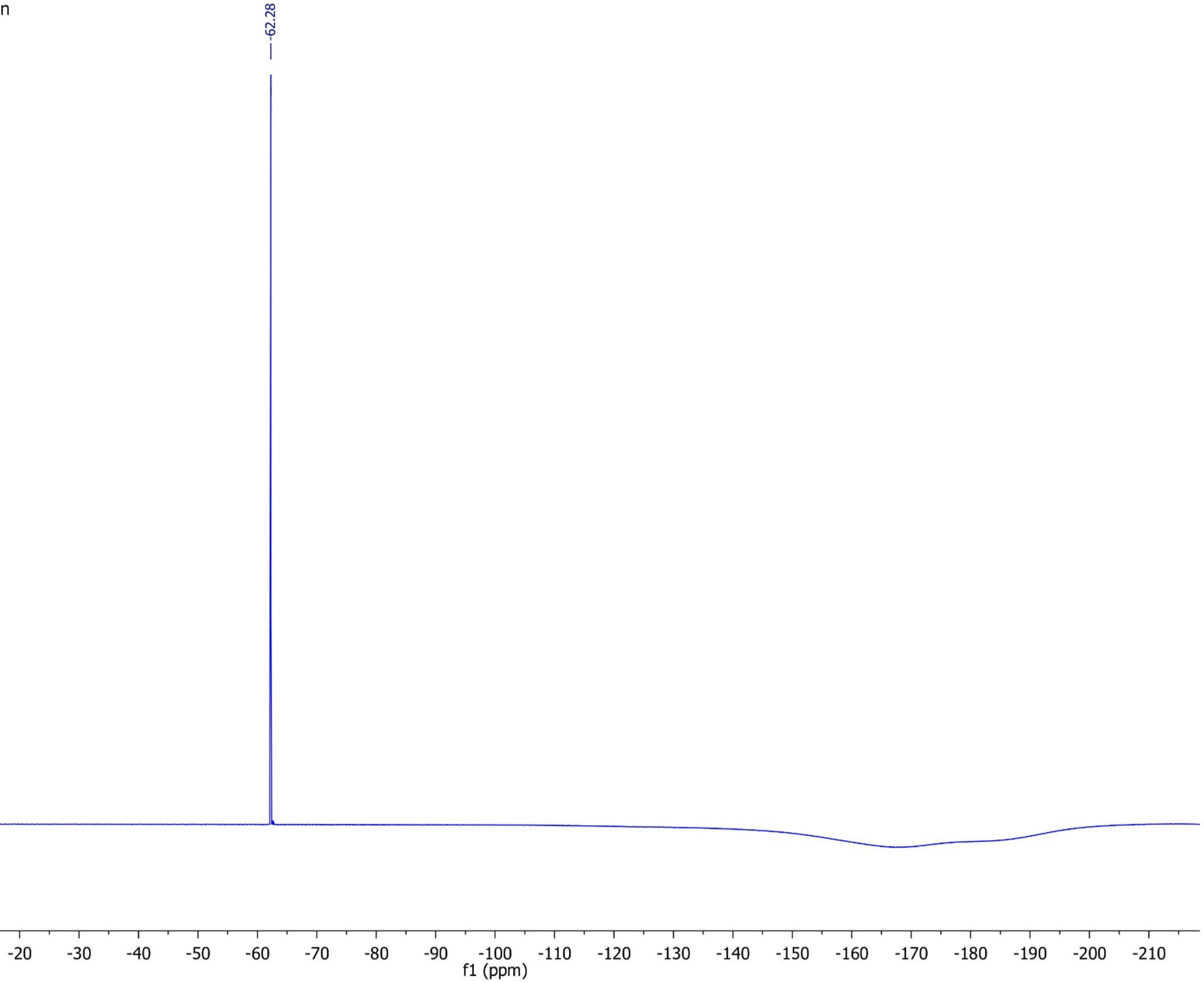

1-(4-Methoxybenzyl)-2-phenyl-1,2,3,4-tetrahydroisoquinoline (11)

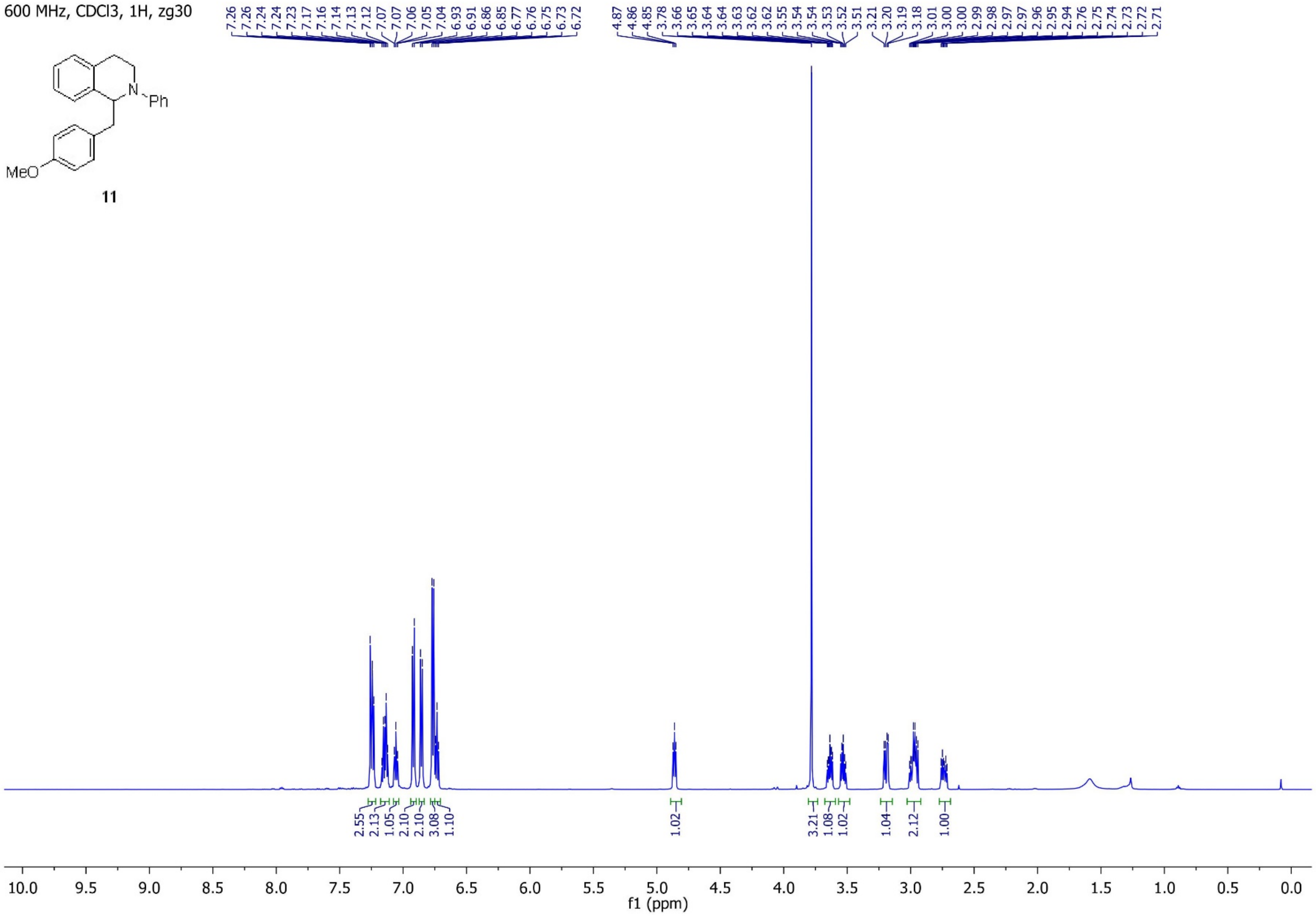

151 MHz, CDCl3, 13C, deptqgpsp

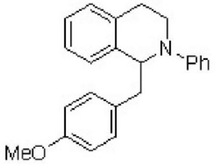

11

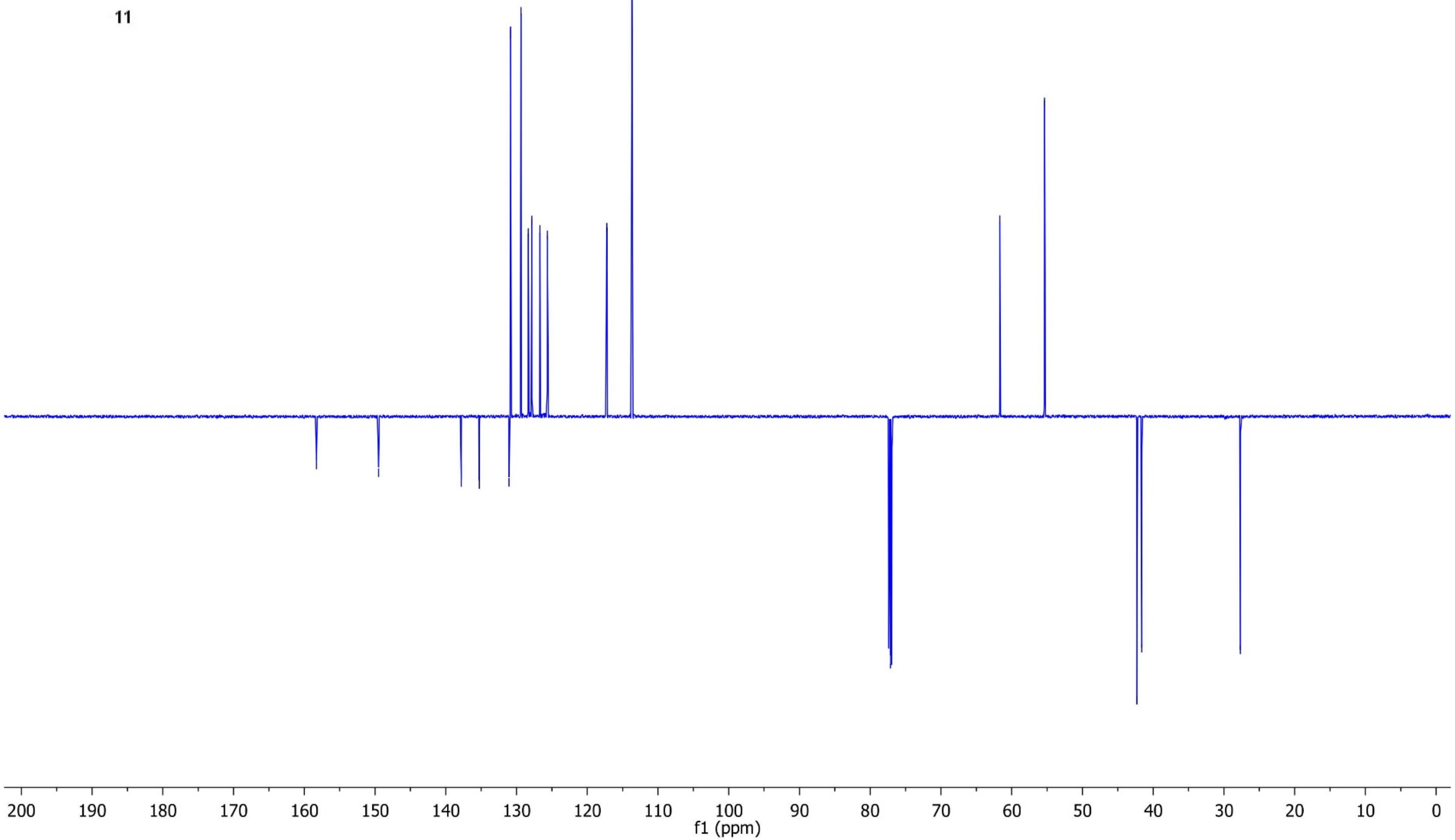

2-Phenyl-1-(pyridin-3-ylmethyl)-1,2,3,4-tetrahydroisoquinoline (12)

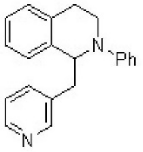

12

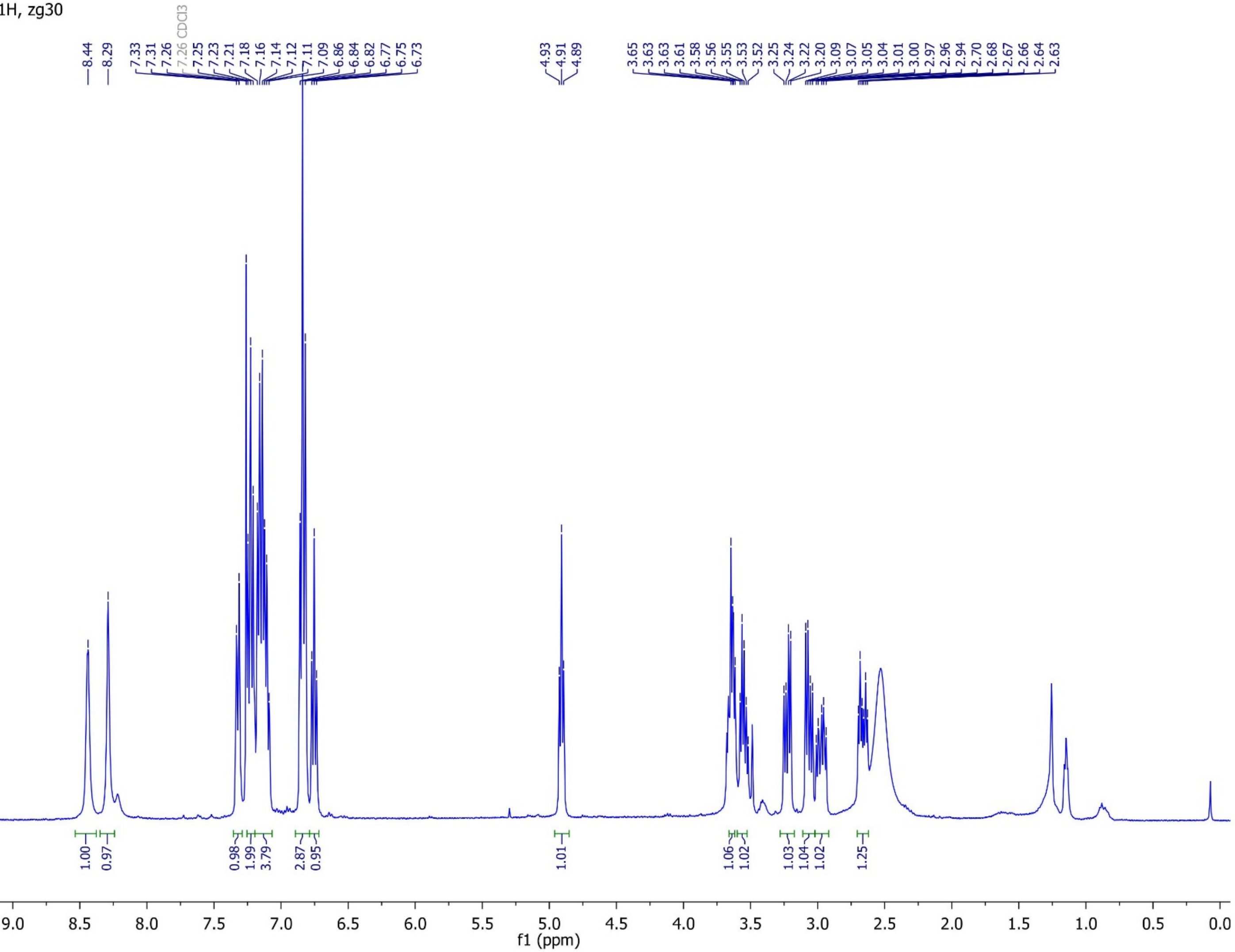

101 MHz, CDCl3, 13C, jmod

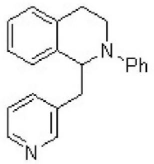

12

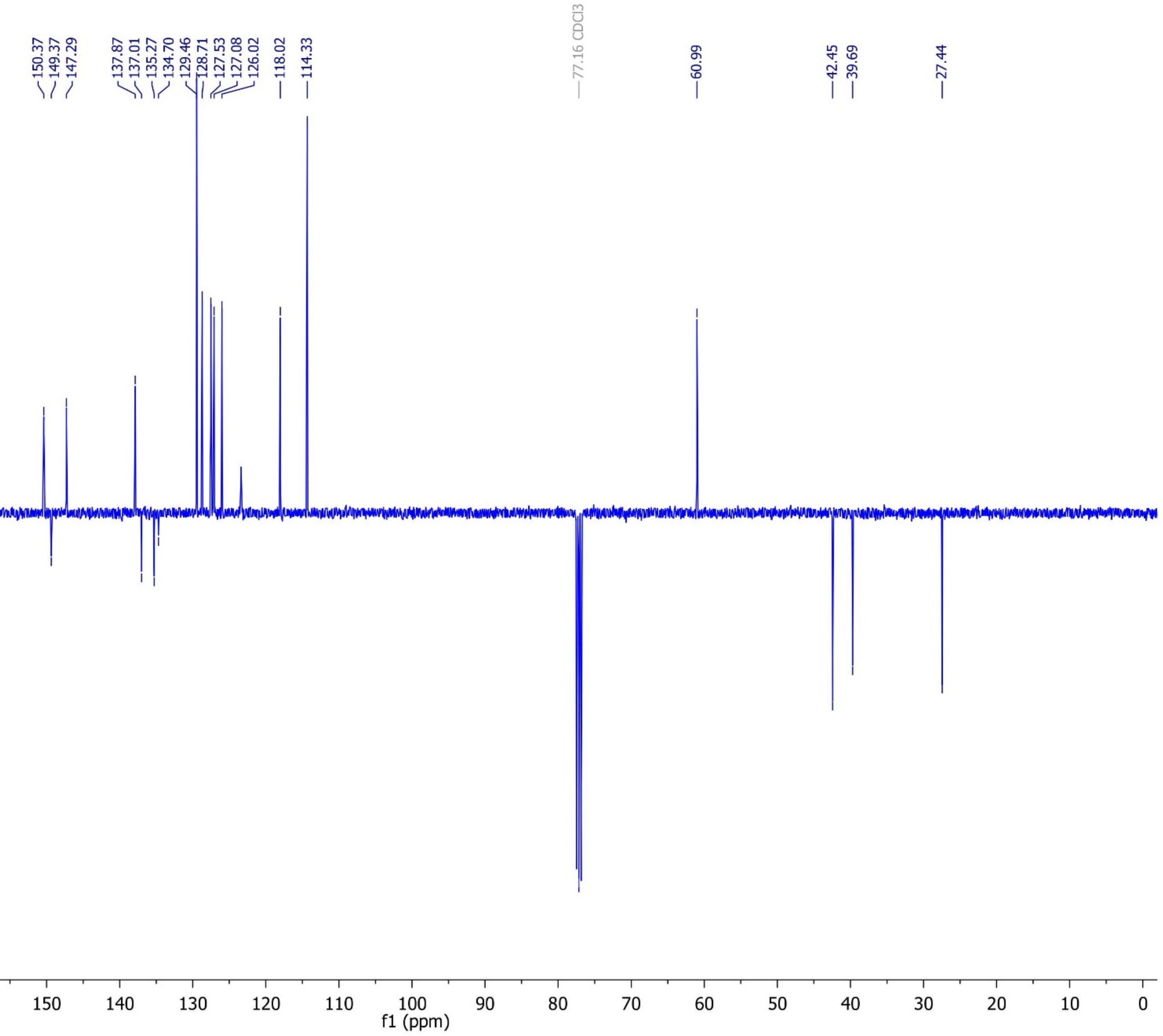

2-Phenyl-1-(pyridin-2-ylmethyl)-1,2,3,4-tetrahydroisoquinoline (13)

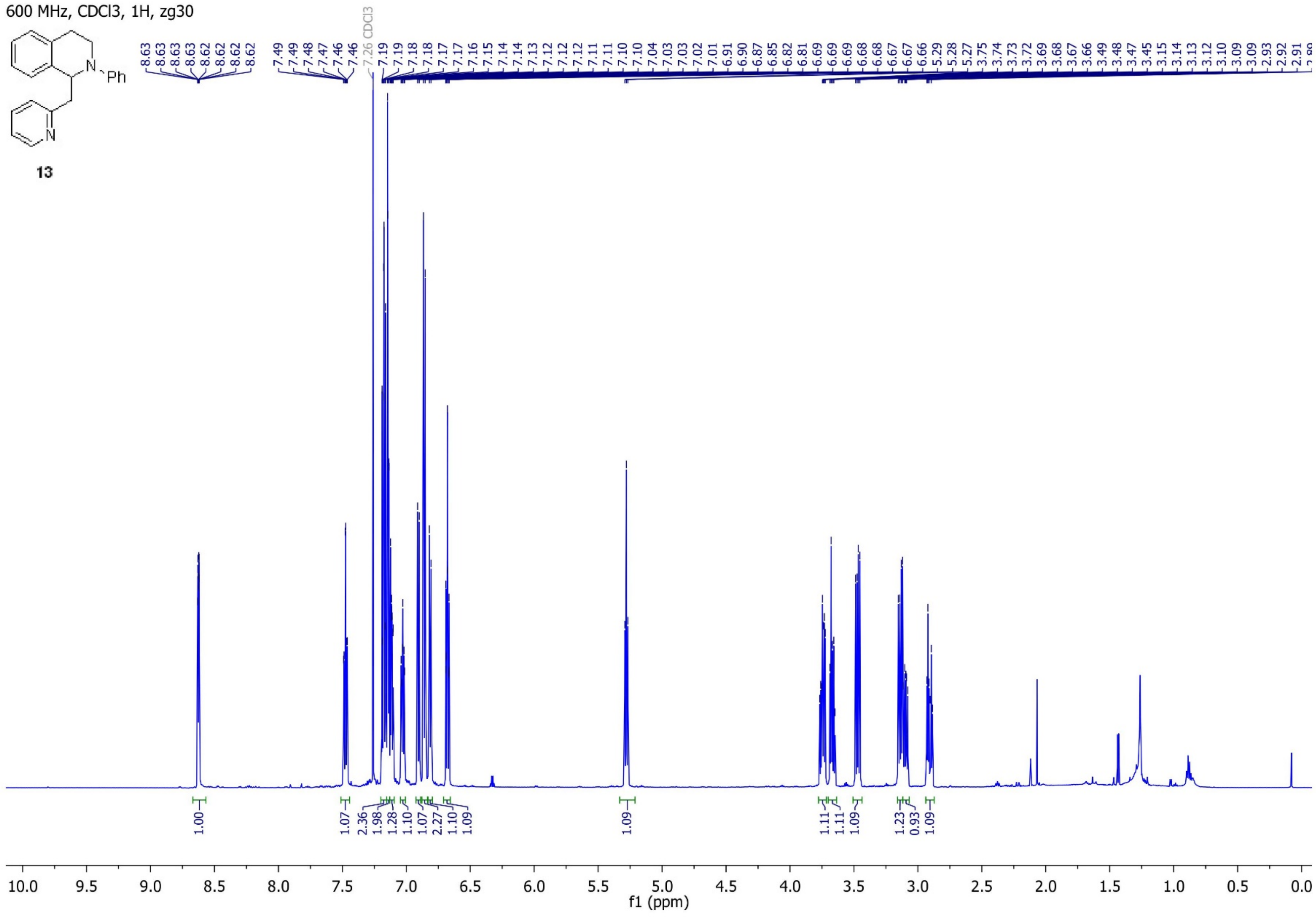

151 MHz, CDCl3, 13C, deptqgpsp

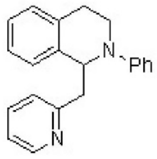

13

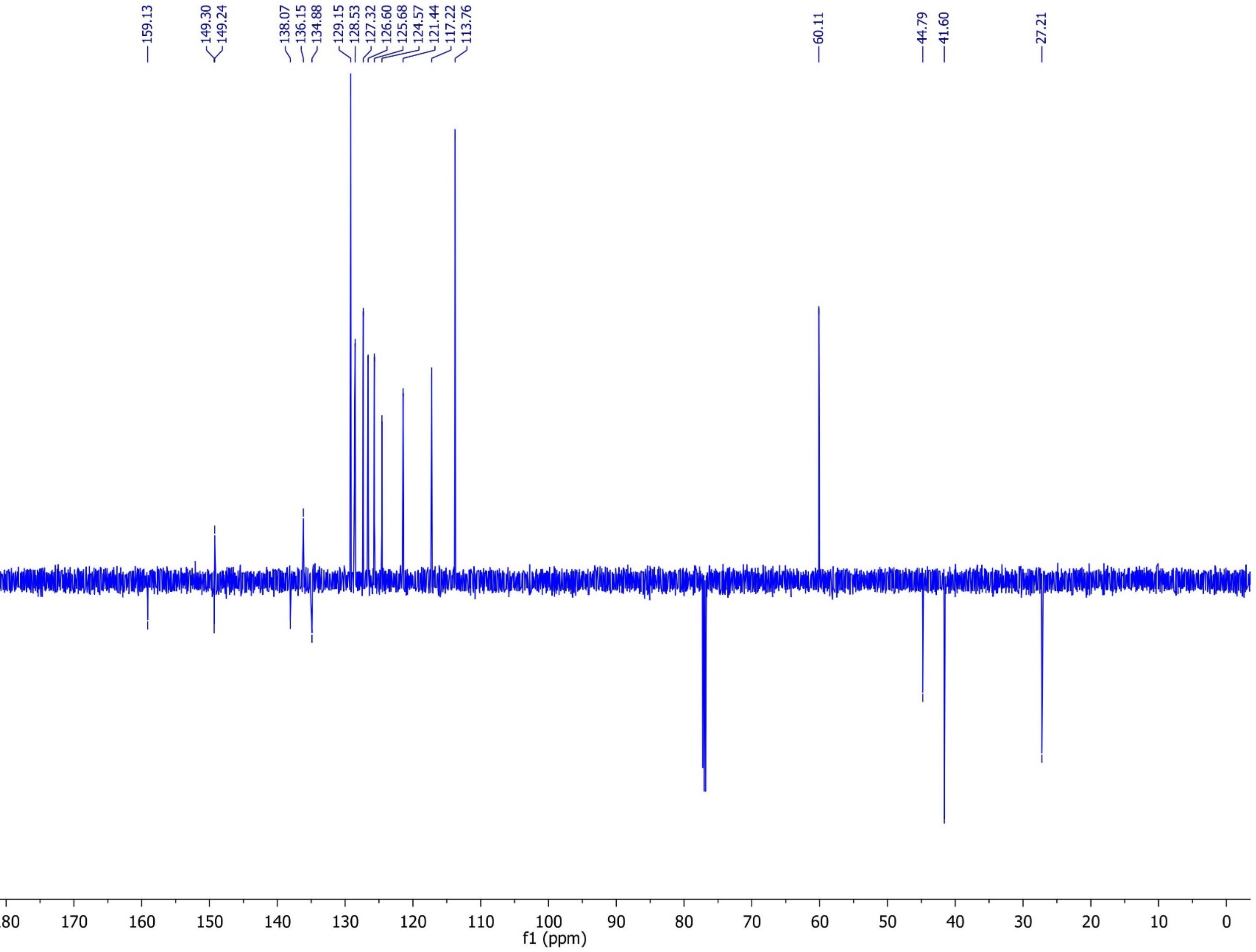

1-Benzyl-2-(4-chlorophenyl)-1,2,3,4-tetrahydroisoquinoline (21)

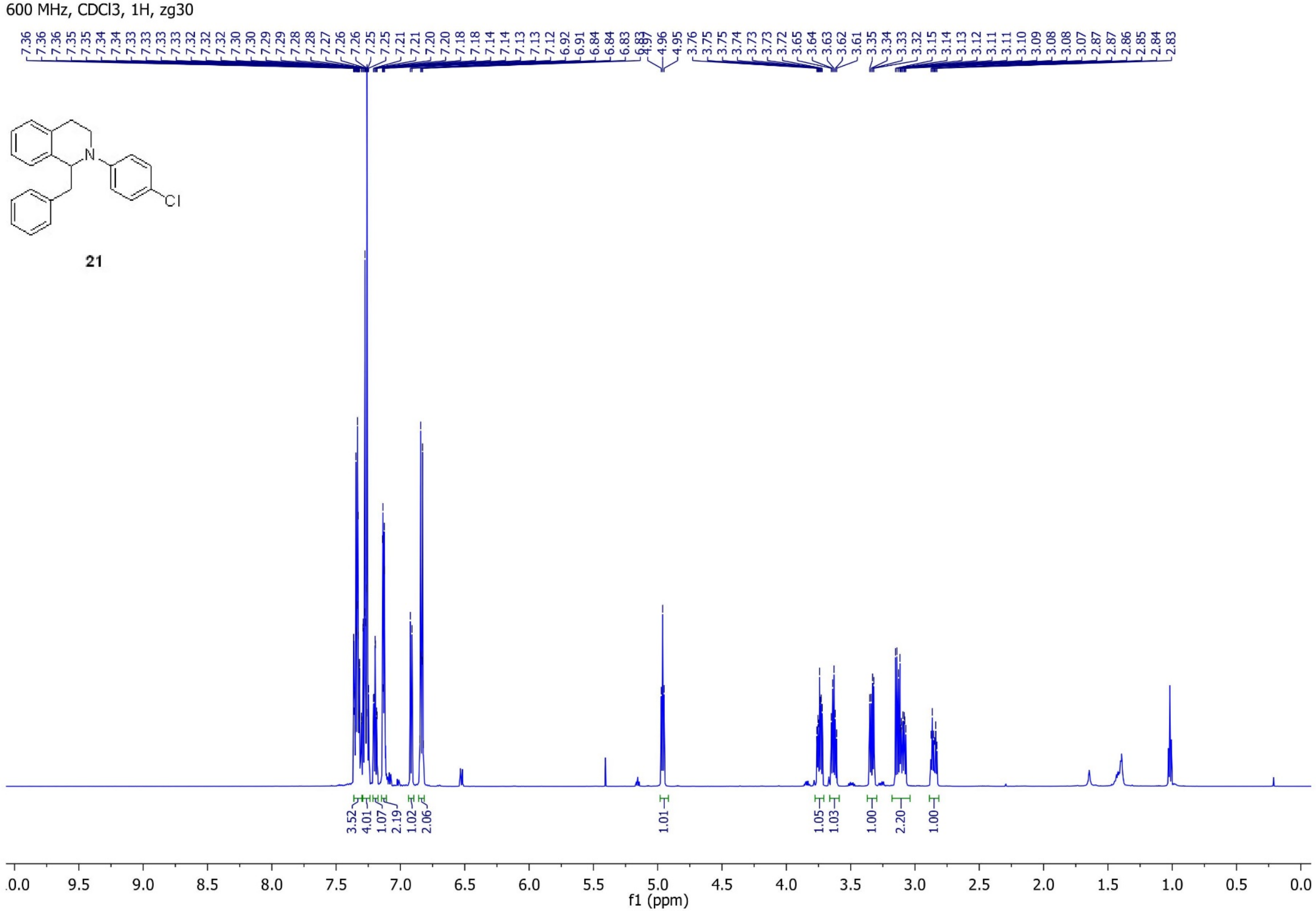

151 MHz, CDCl3, 13C, deptqgpsp

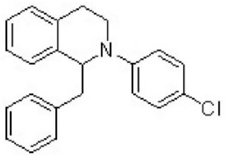

21

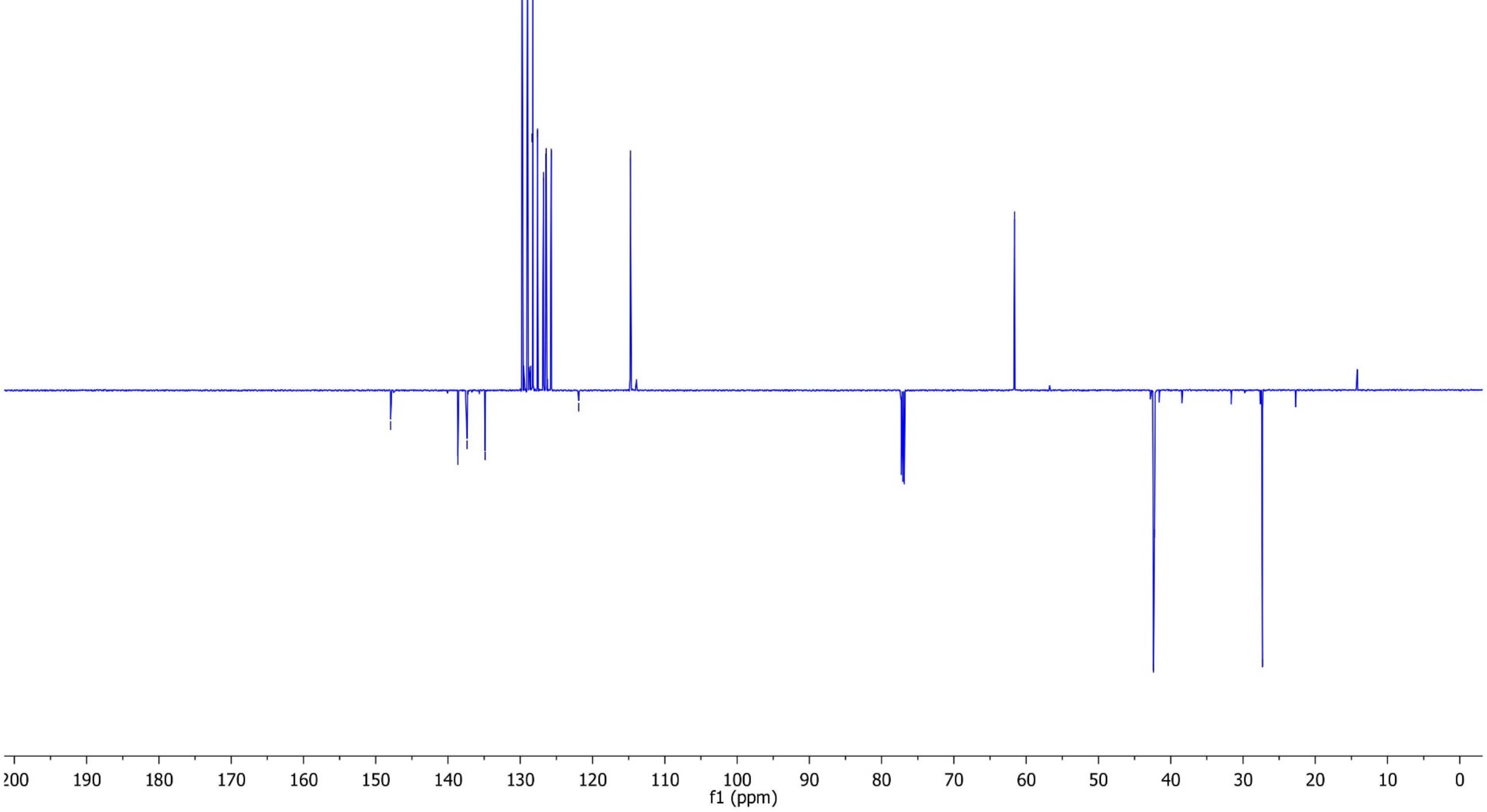

1-Benzyl-2-(4-bromophenyl)-1,2,3,4-tetrahydroisoquinoline (22)

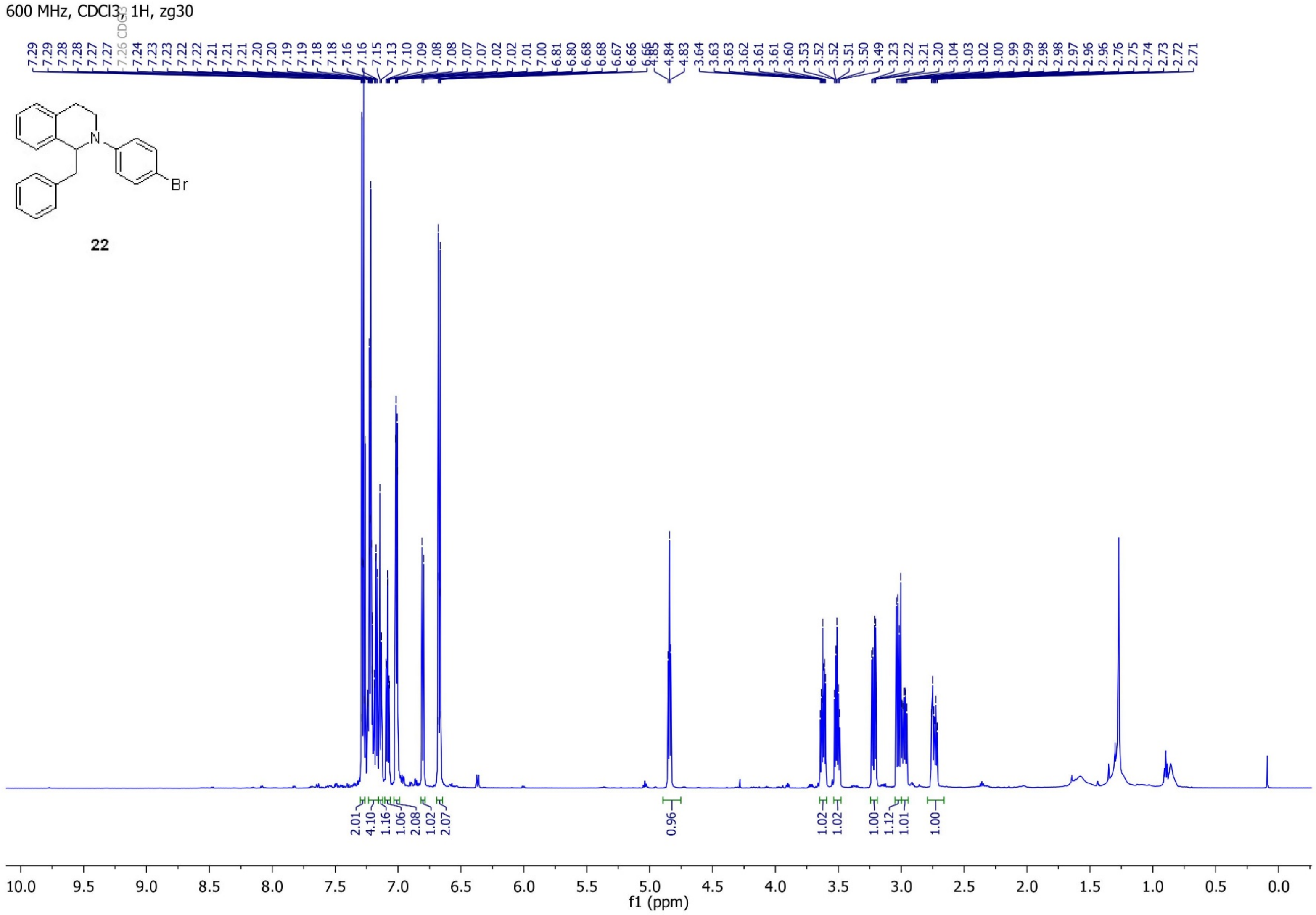

151 MHz, CDCl3, 13C, deptqgpsp

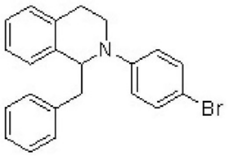

22

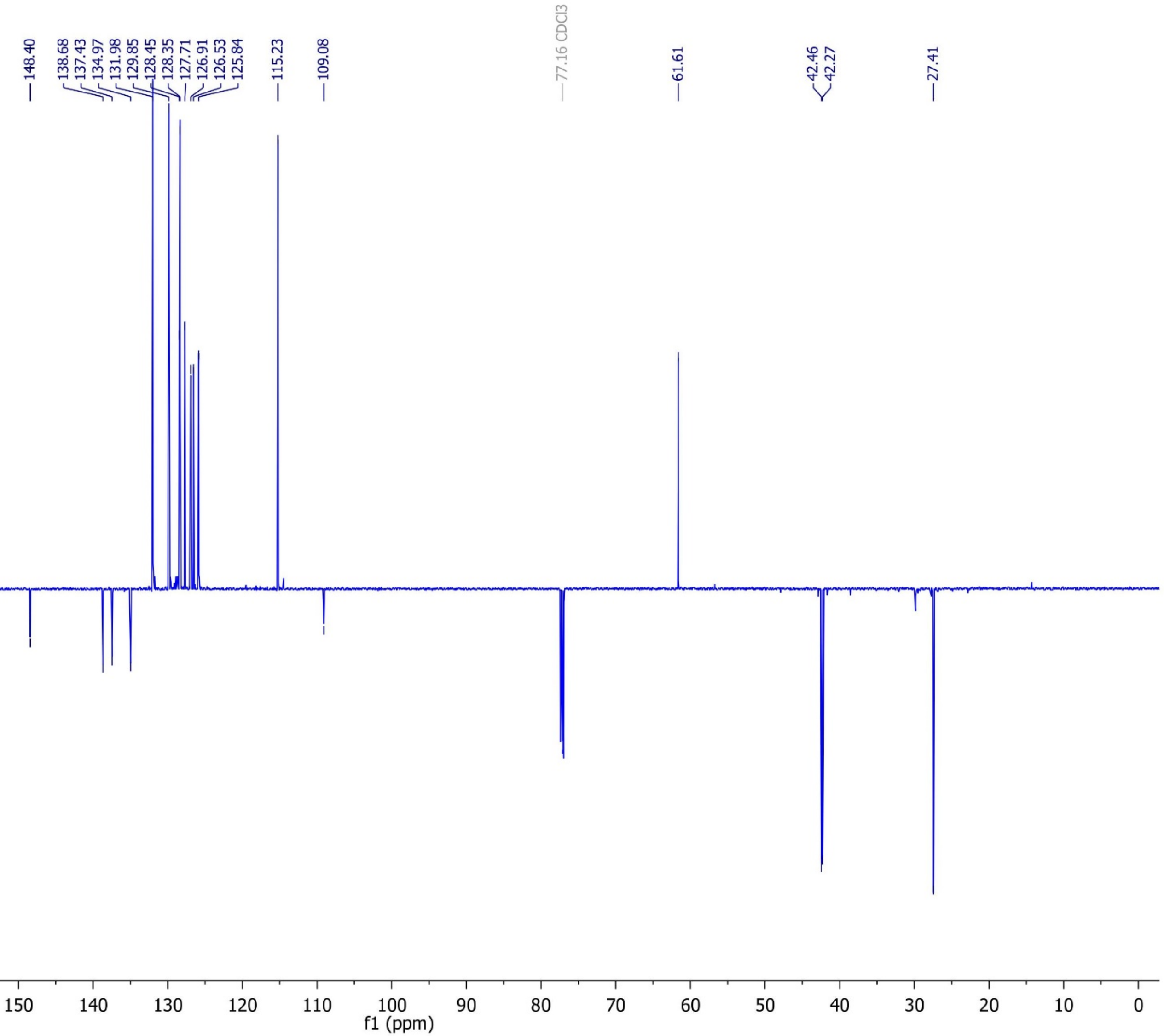

4-(1-Benzyl-3,4-dihydroisoquinolin-2(1H)-yl)benzonitrile (23)

400 MHz, CDCl3, 1H, zg30

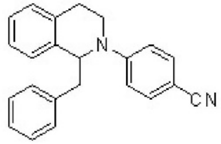

23

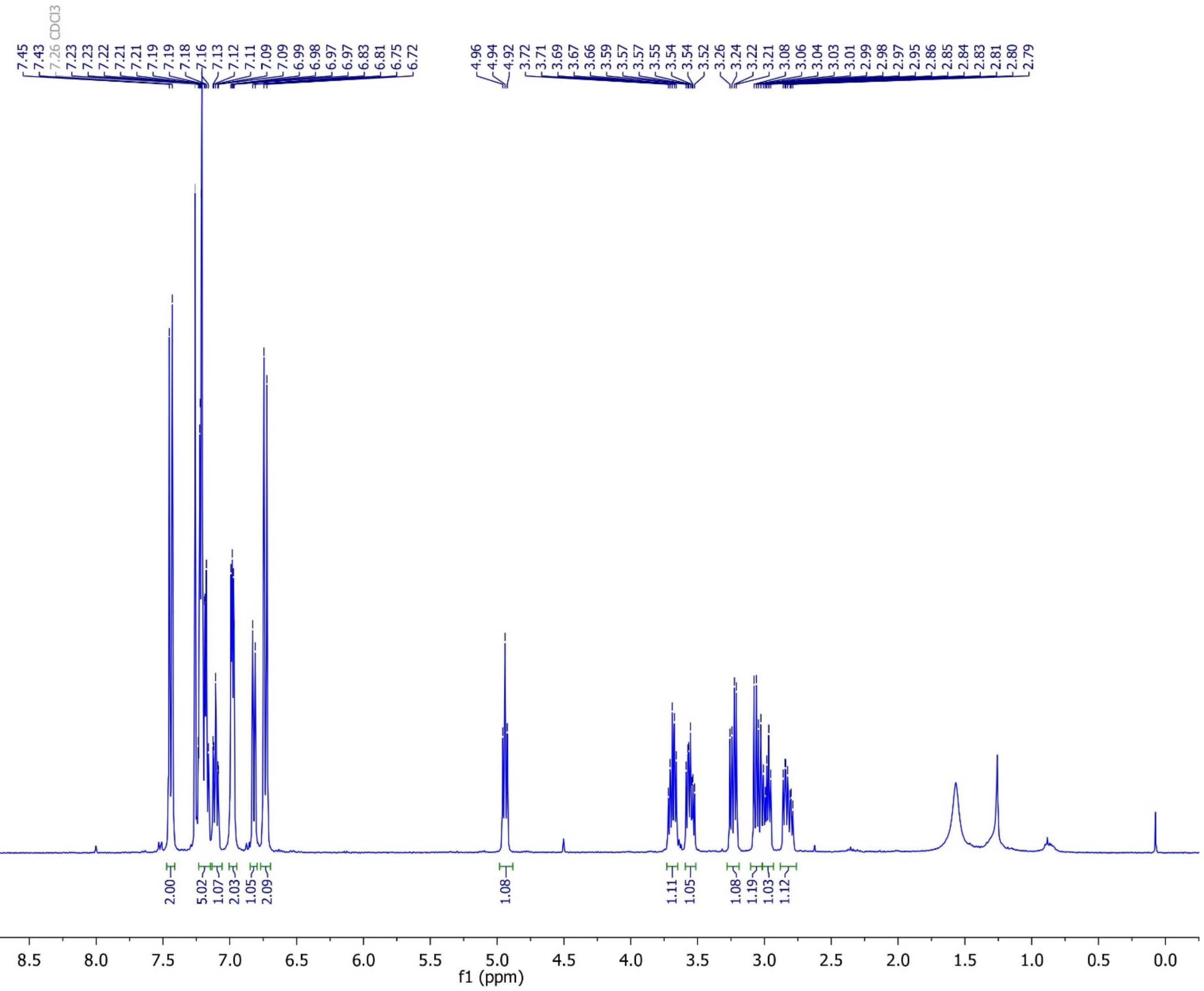

101 MHz, CDCl3, 13C, jmod

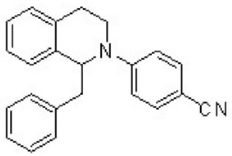

23

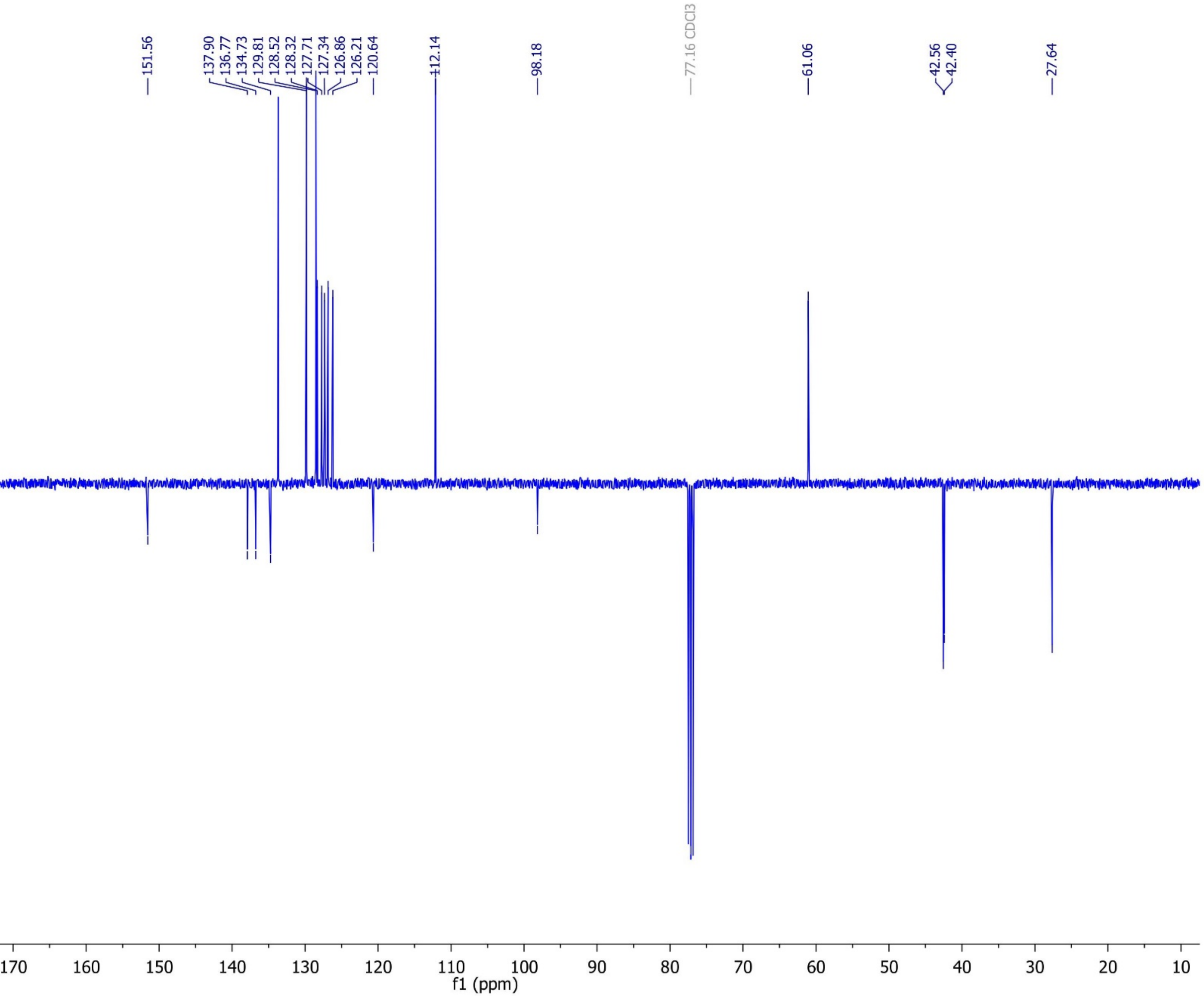

1-Benzyl-2-(4-methoxyphenyl)-1,2,3,4-tetrahydroisoquinoline (24)

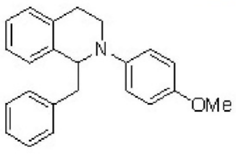

24

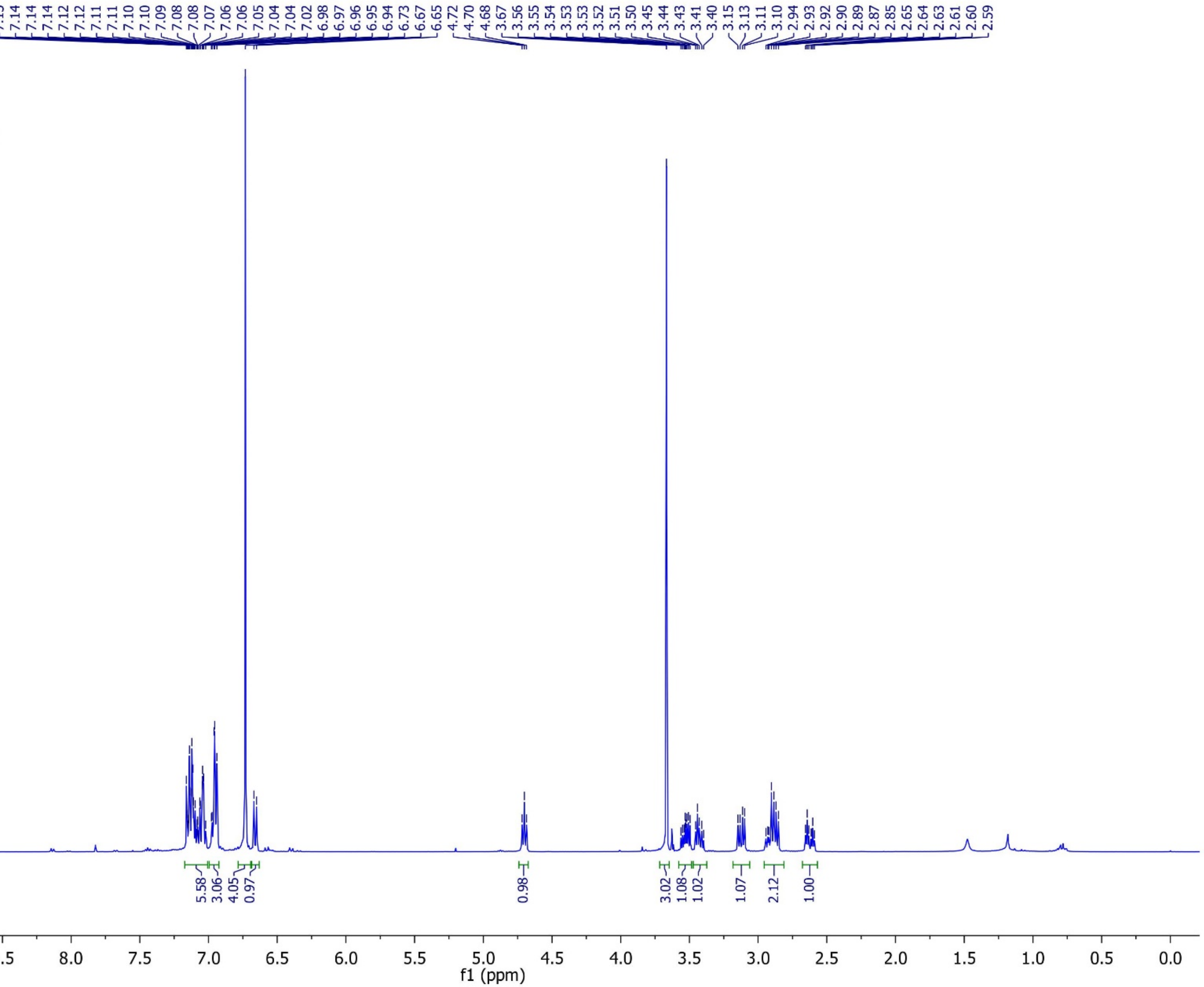

101 MHz, CDCl3, 13C, jmod

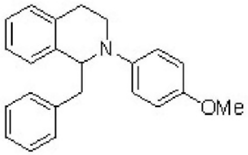

24

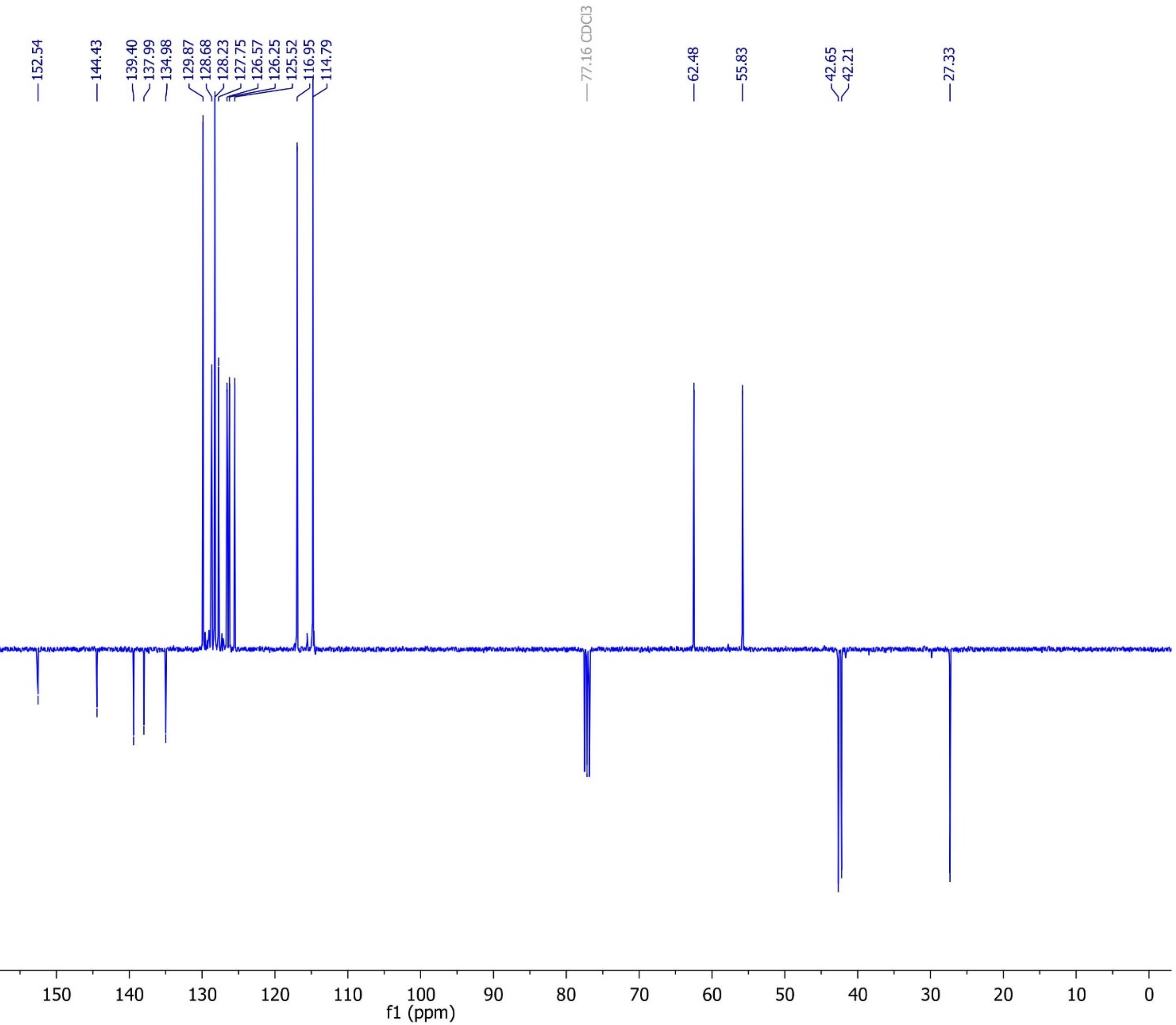

Methyl 4-(1-benzyl-3,4-dihydroisoquinolin-2(1H)-yl)benzoate (25)

400 MHz, CDCl3, 1H, zg30

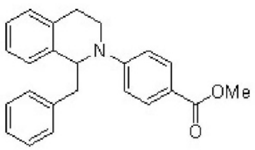

25

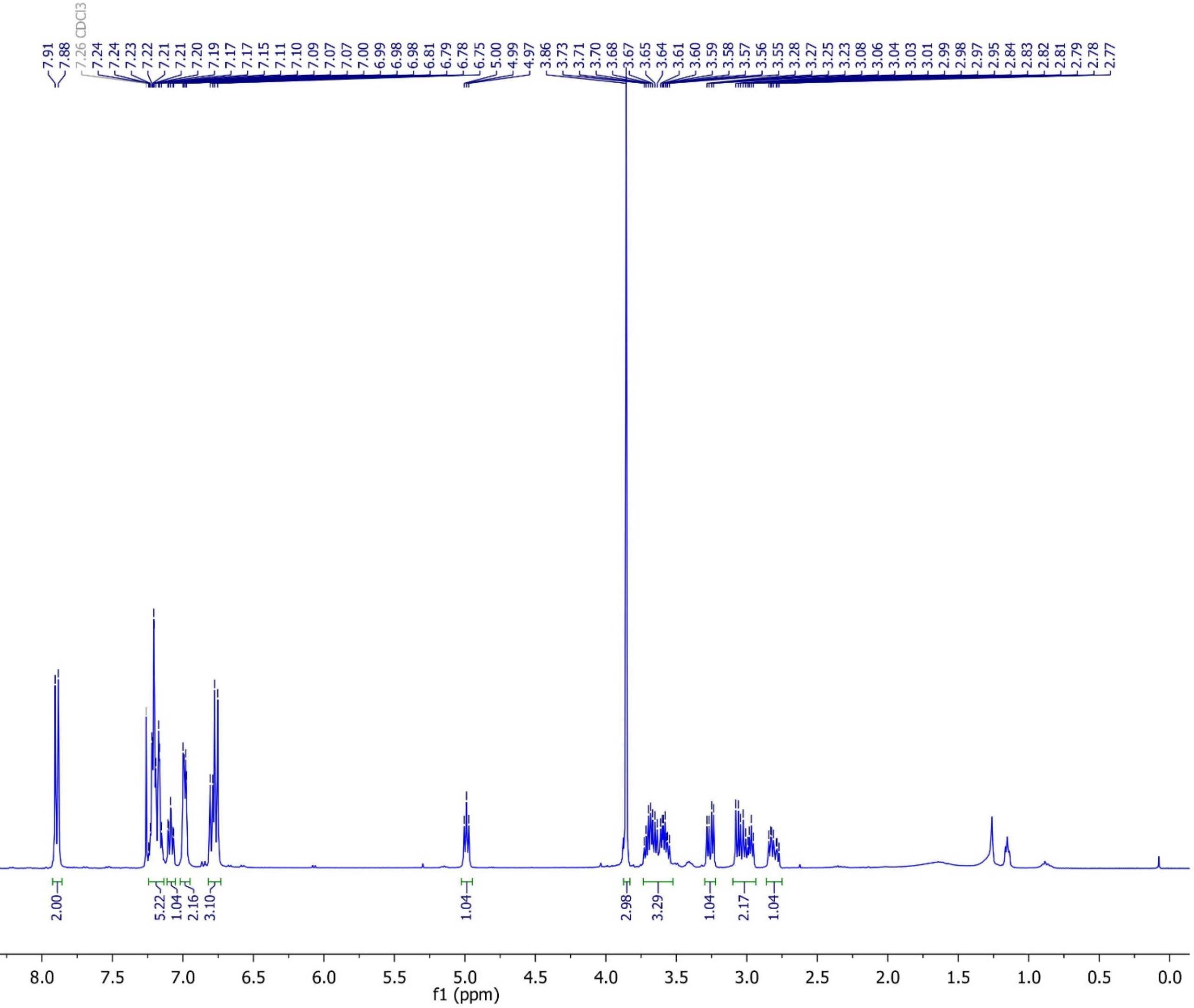

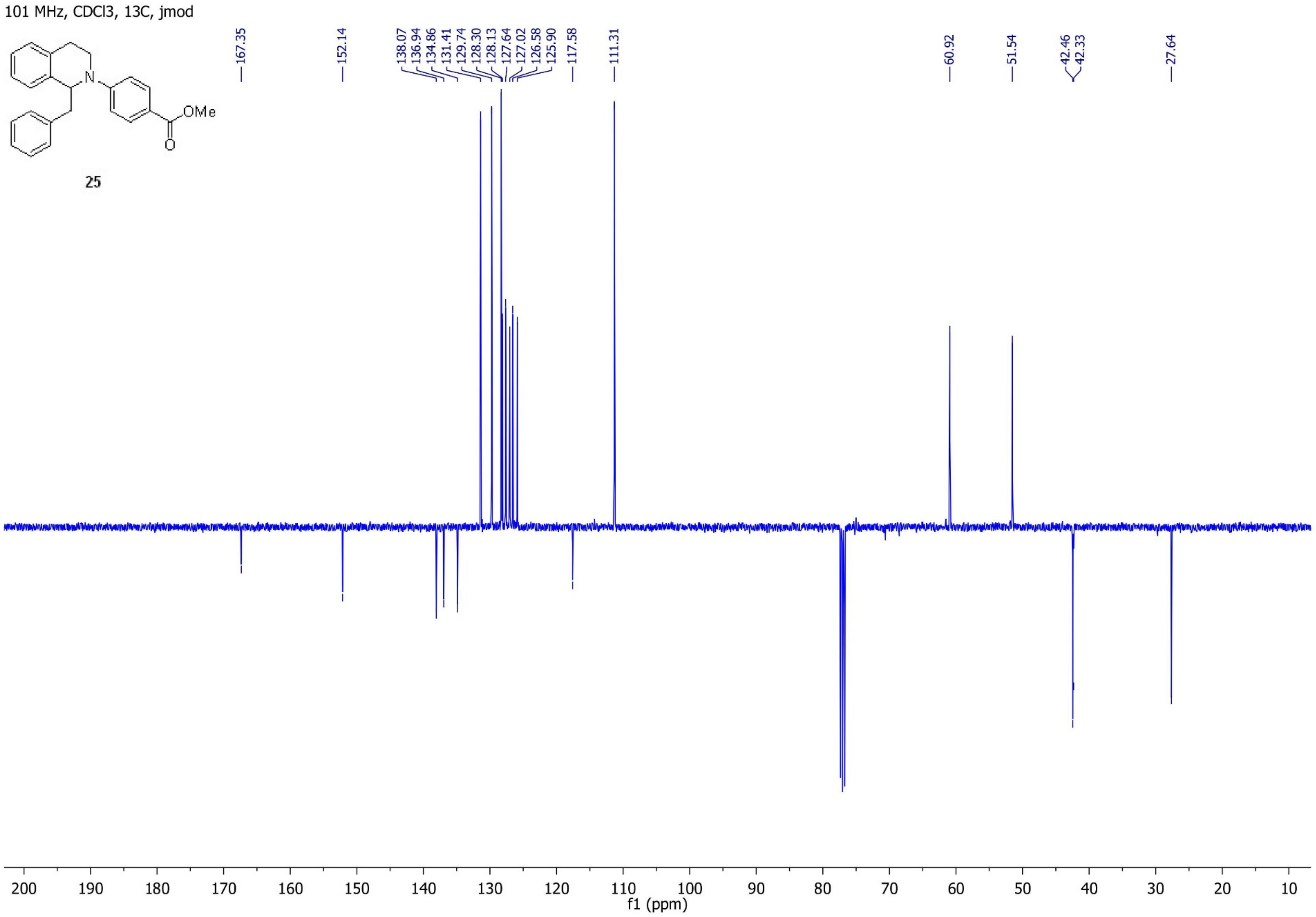
101 MHz, CDCl3, 13C, jmod
N
OMe
O
25
167.35
152.14
138.07
136.94
134.86
131.41
129.74
128.30
128.13
127.64
127.02
126.58
125.90
117.58
111.31
60.92
51.54
42.46
42.33
27.64
200
190
180
170
160
150
140
130
120
110
100
90
80
70
60
50
40
30
20
10
f1 (ppm)

1-Benzyl-7-fluoro-2-phenyl-1,2,3,4-tetrahydroisoquinoline (26)

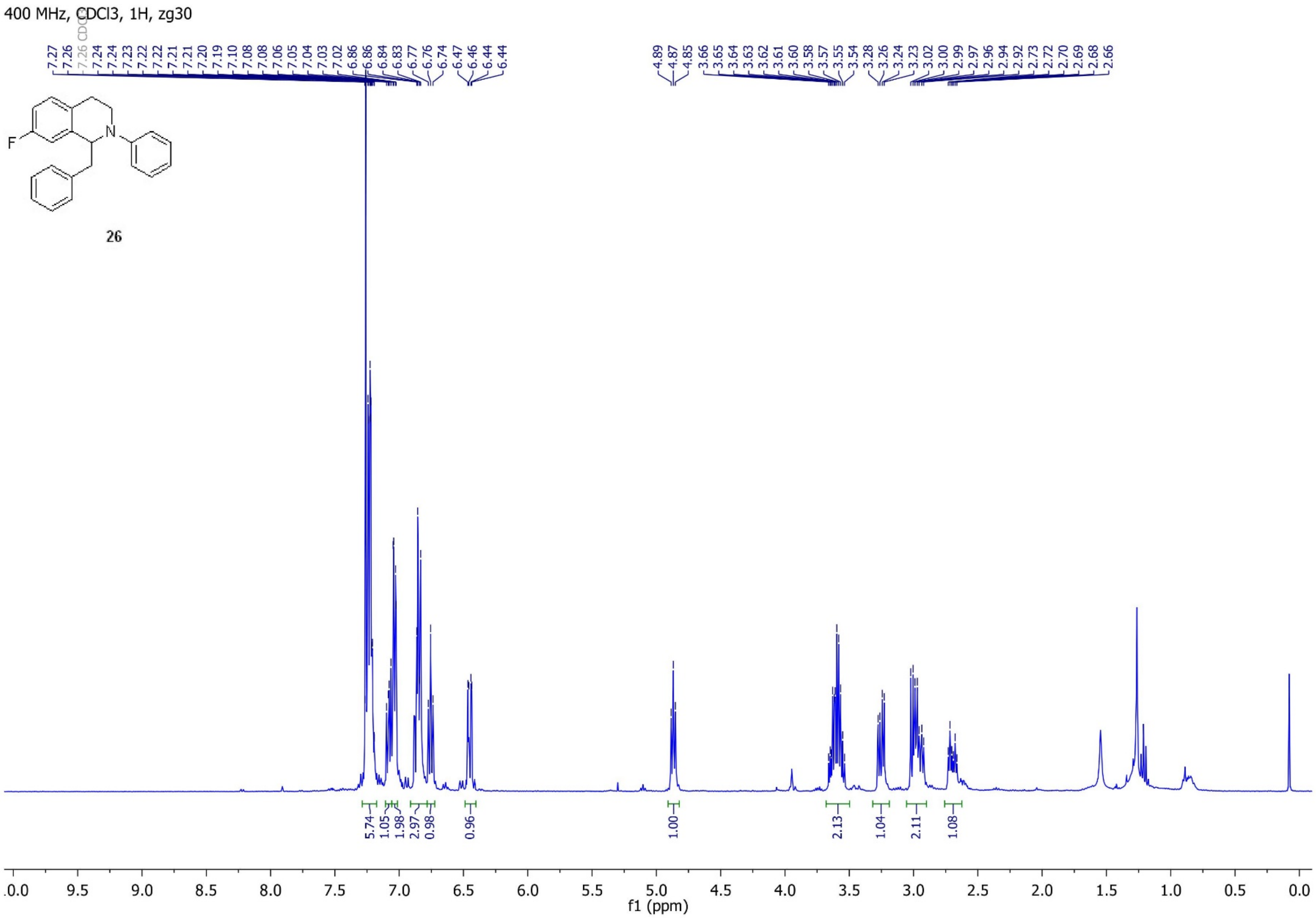

101 MHz, CDCl3, 13C, zgpg30

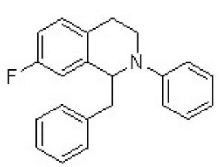

26

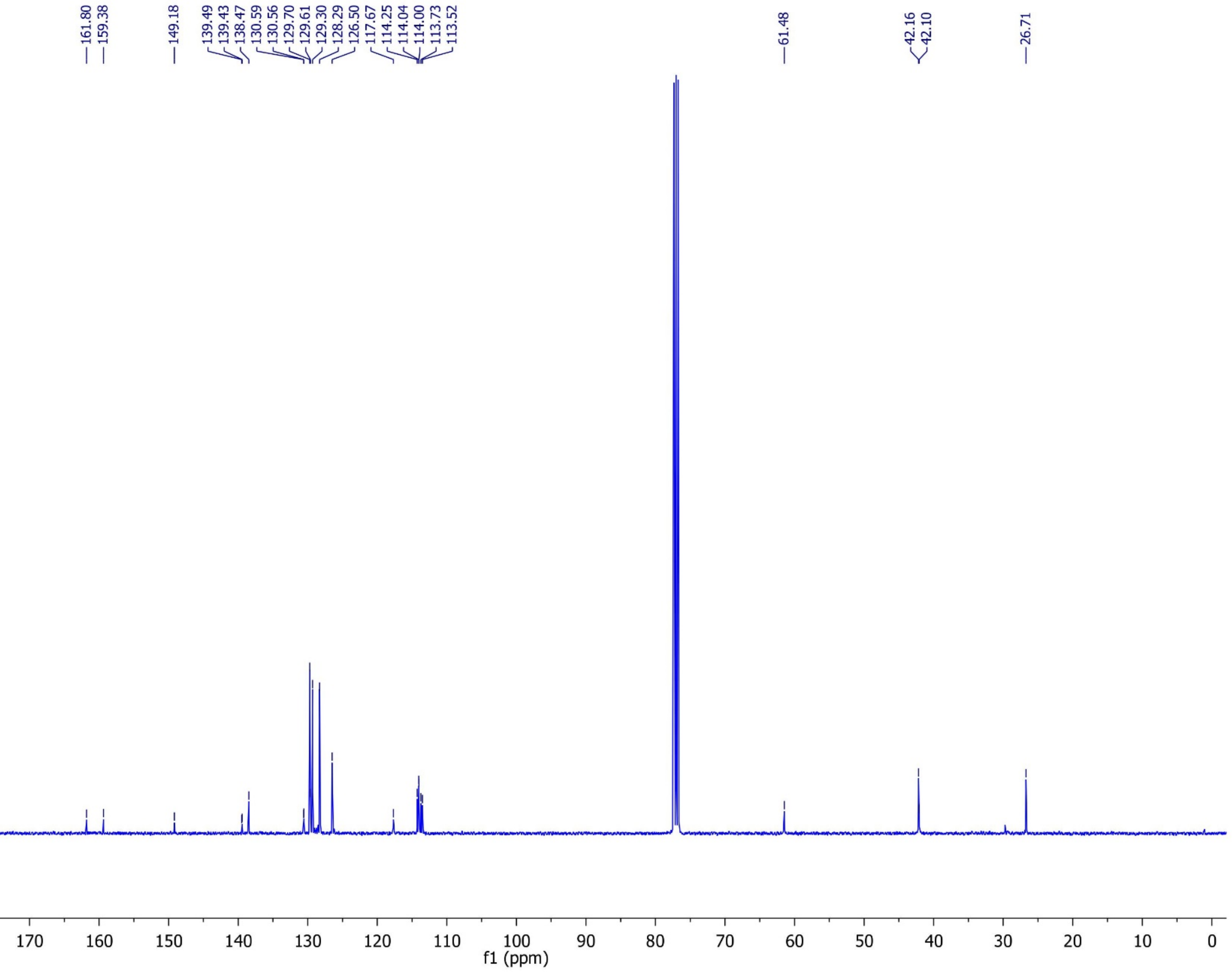

376 MHz, CDCl3, 19F, zgflqn

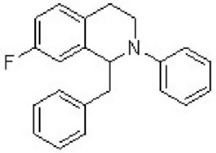

26

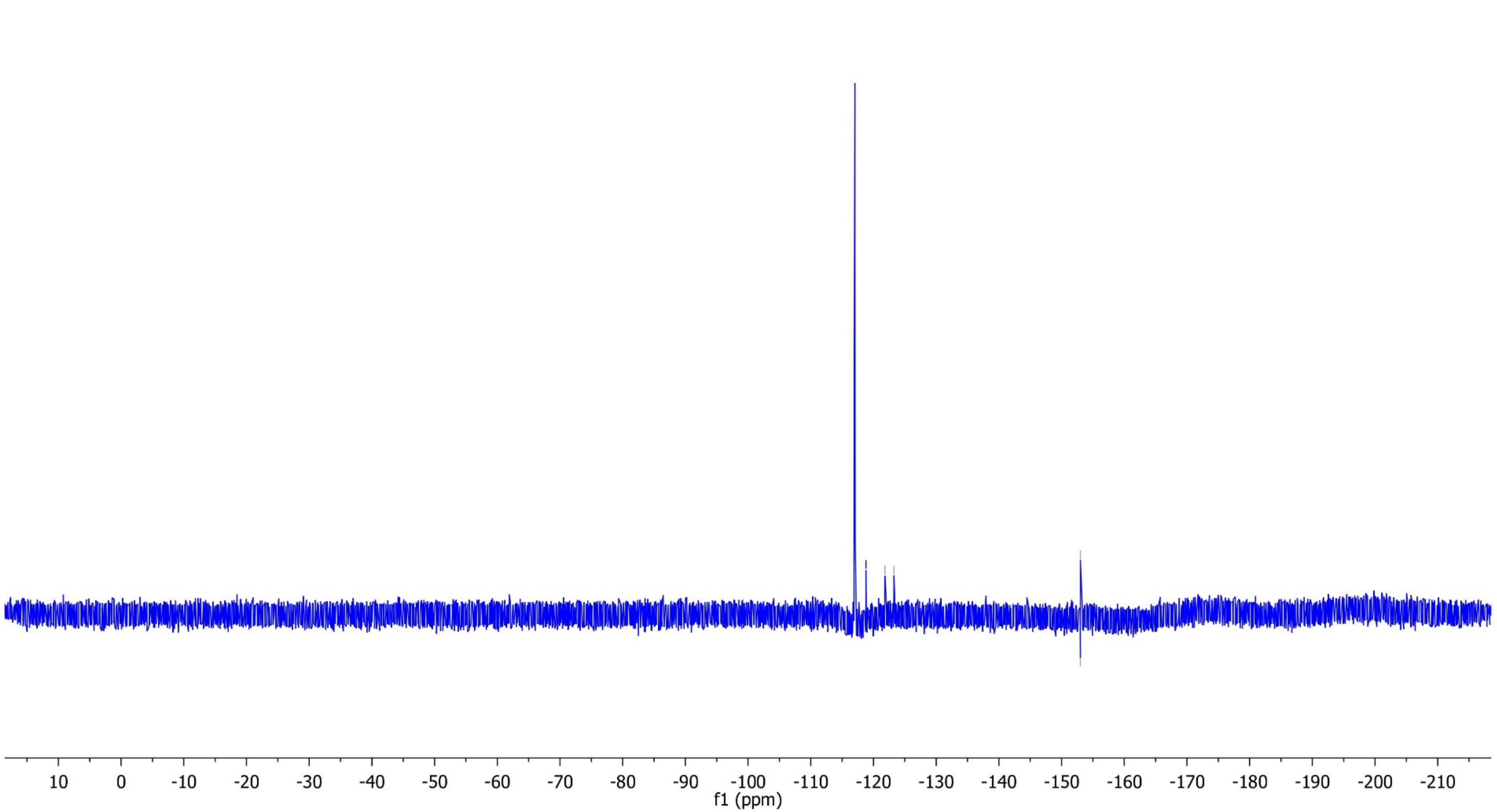

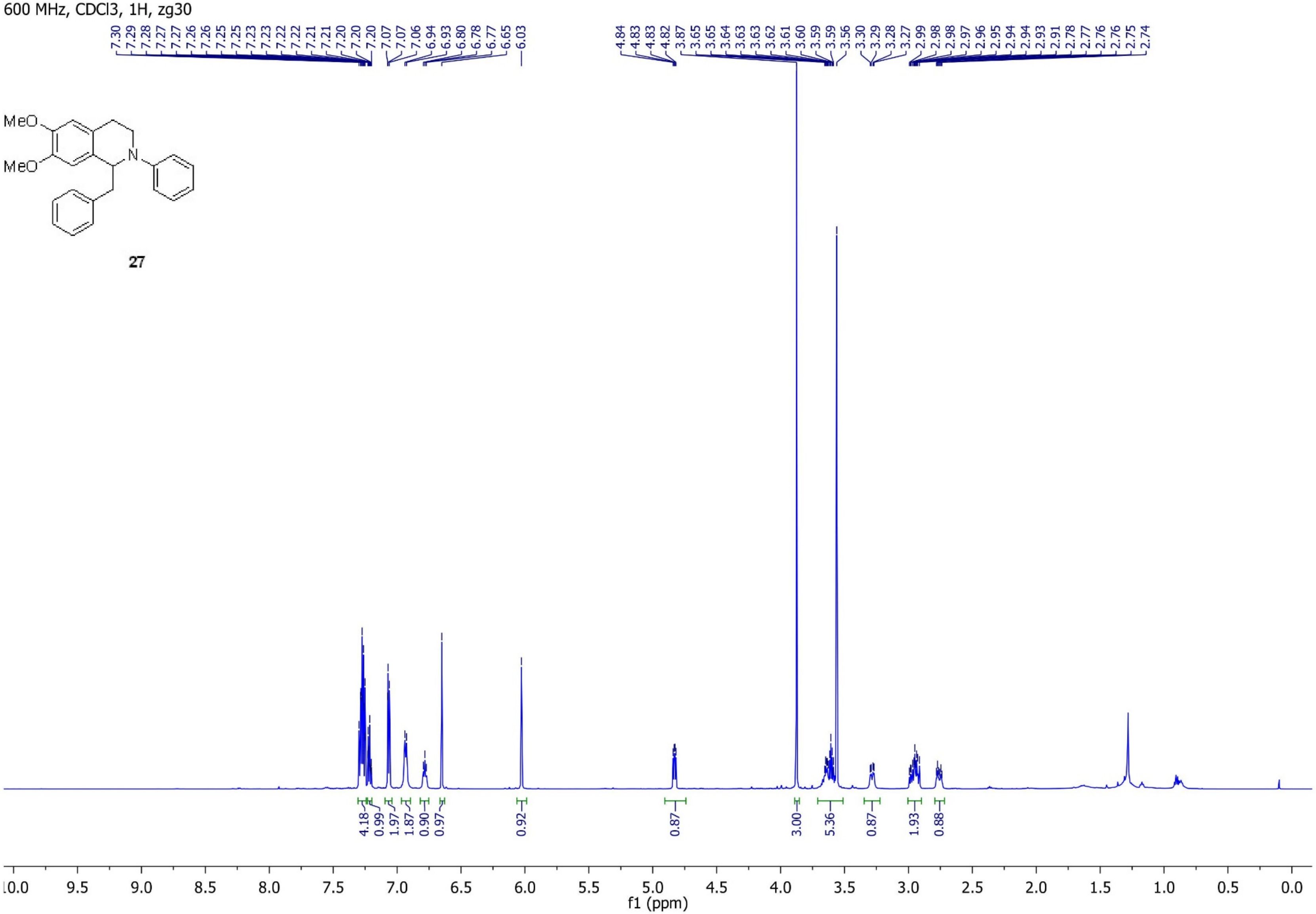
600 MHz, CDCl3, 1H, zg30
7.30 7.29 7.28 7.27 7.27 7.26 7.26 7.25 7.25 7.23 7.23 7.22 7.22 7.21 7.21 7.20 7.20 7.20 7.07 7.07 7.06 6.94 6.93 6.80 6.78 6.77 6.65 6.03
4.84 4.83 4.83 4.82 3.87 3.65 3.65 3.64 3.63 3.63 3.62 3.61 3.60 3.59 3.59 3.56 3.30 3.29 3.28 3.27 2.99 2.98 2.98 2.97 2.96 2.95 2.94 2.94 2.93 2.91 2.78 2.77 2.76 2.75 2.74
MeO
MeO
N
27
4.18 0.99 1.97 1.87 0.90 0.97 0.92 0.87 3.00 5.36 0.87 1.93 0.88
10.0 9.5 9.0 8.5 8.0 7.5 7.0 6.5 6.0 5.5 5.0 4.5 4.0 3.5 3.0 2.5 2.0 1.5 1.0 0.5 0.0
f1 (ppm)

151 MHz, CDCl3, 13C, jmod

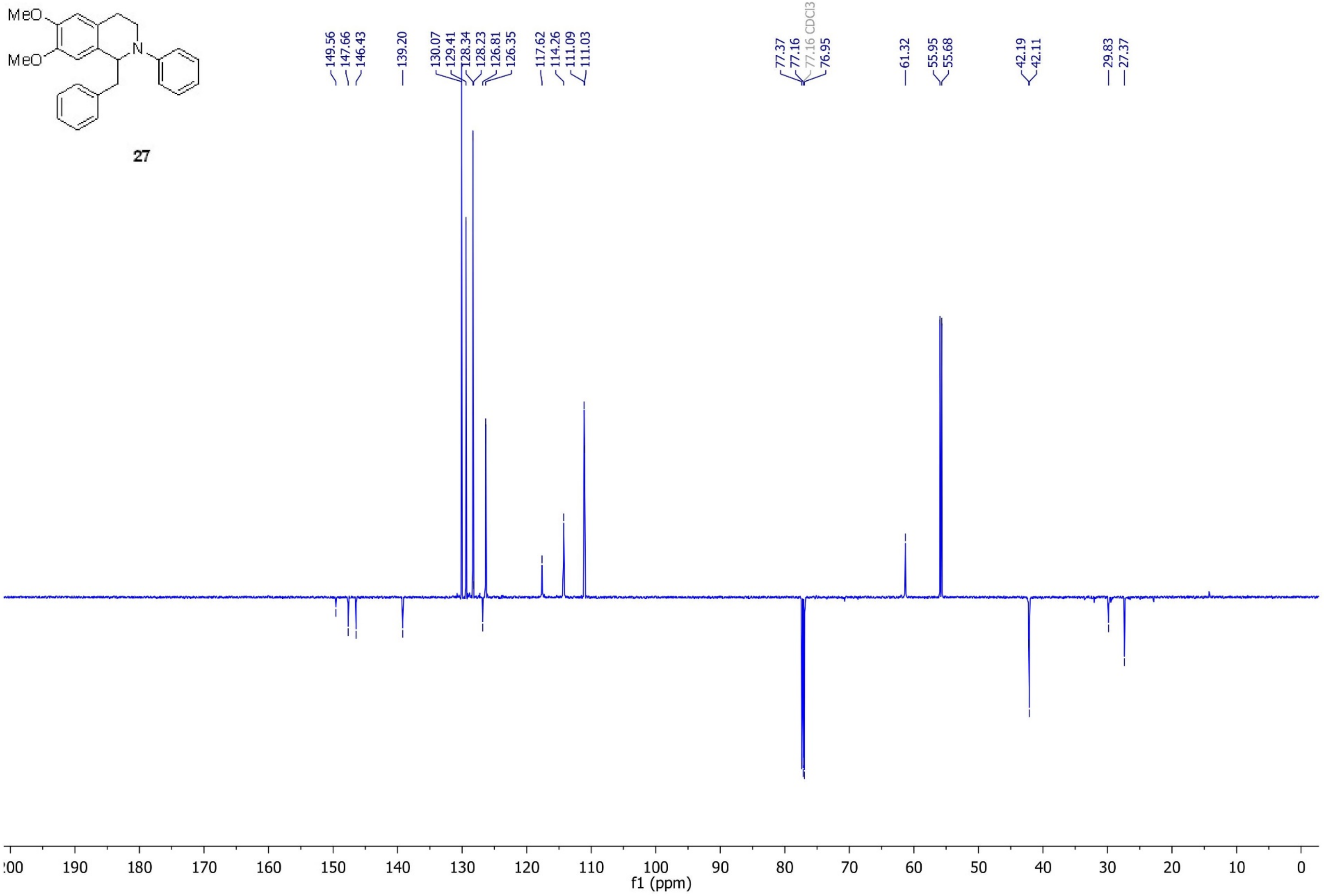

6,7-Dimethoxy-1-(4-methylbenzyl)-2-phenyl-1,2,3,4-tetrahydroisoquinoline (28)

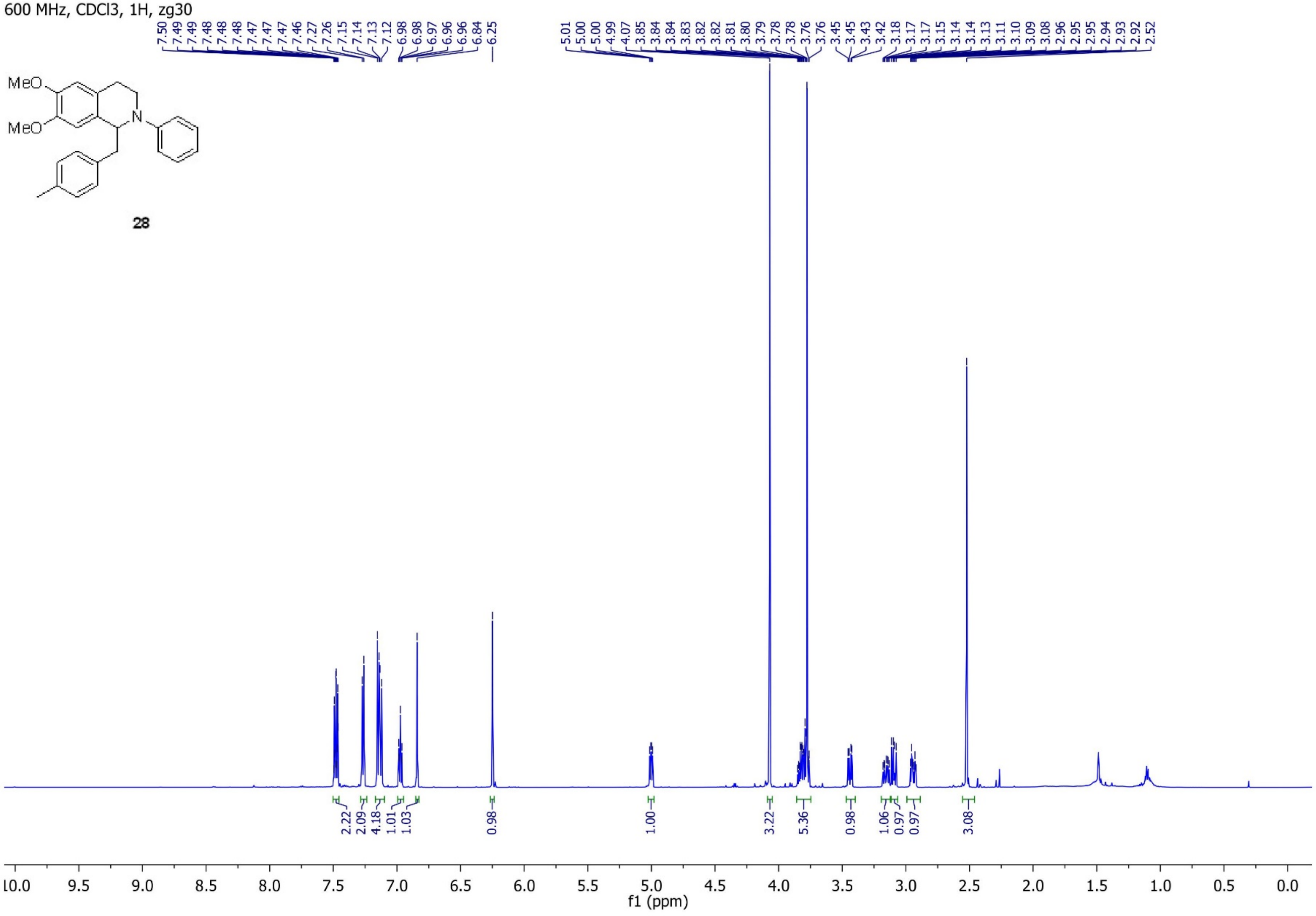

151 MHz, CDCl3, 13C, deptqgpsp

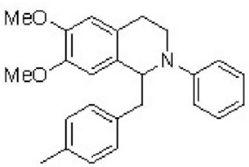

28

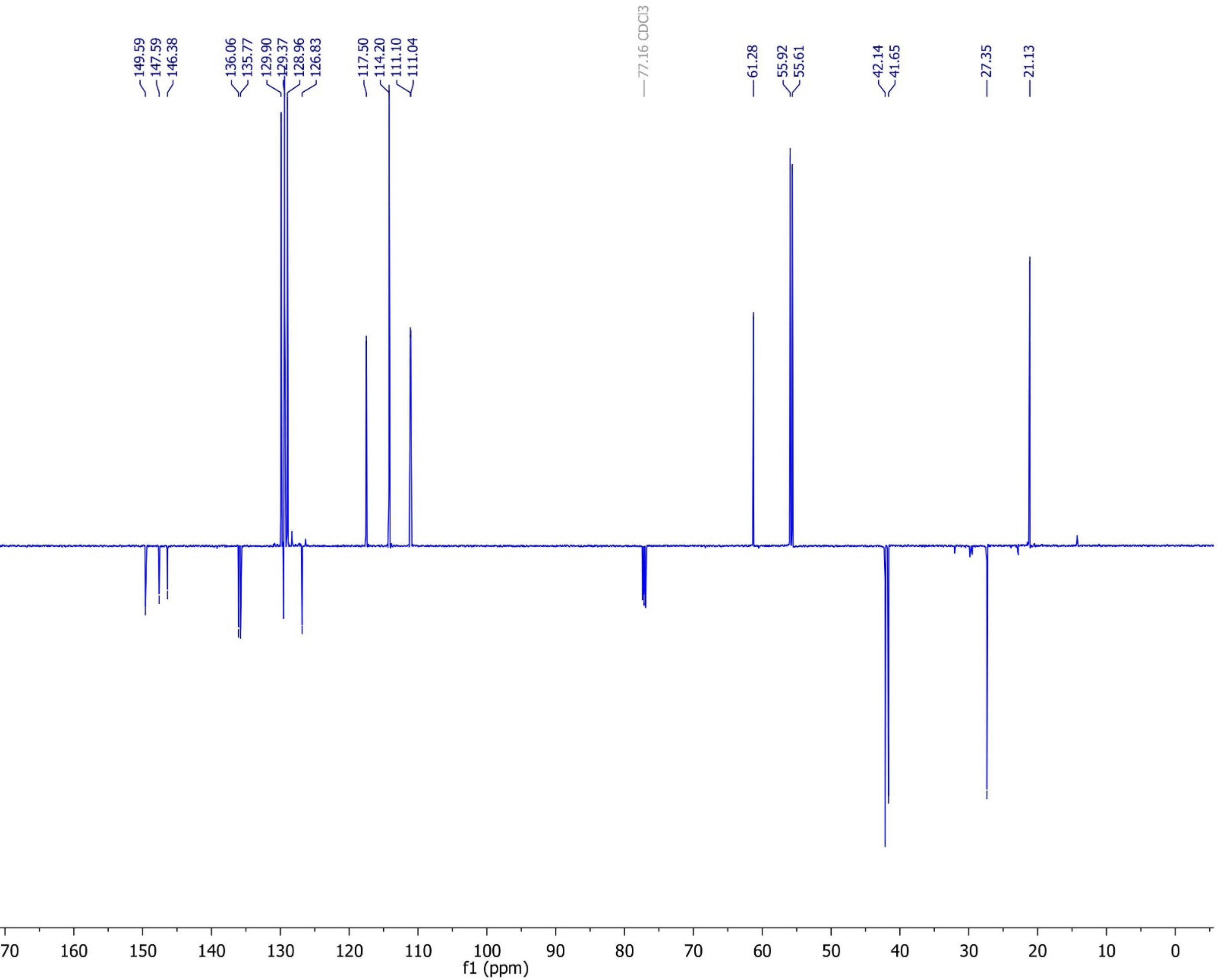

6,7-Dimethoxy-1-(2-methylbenzyl)-2-phenyl-1,2,3,4-tetrahydroisoquinoline (29)

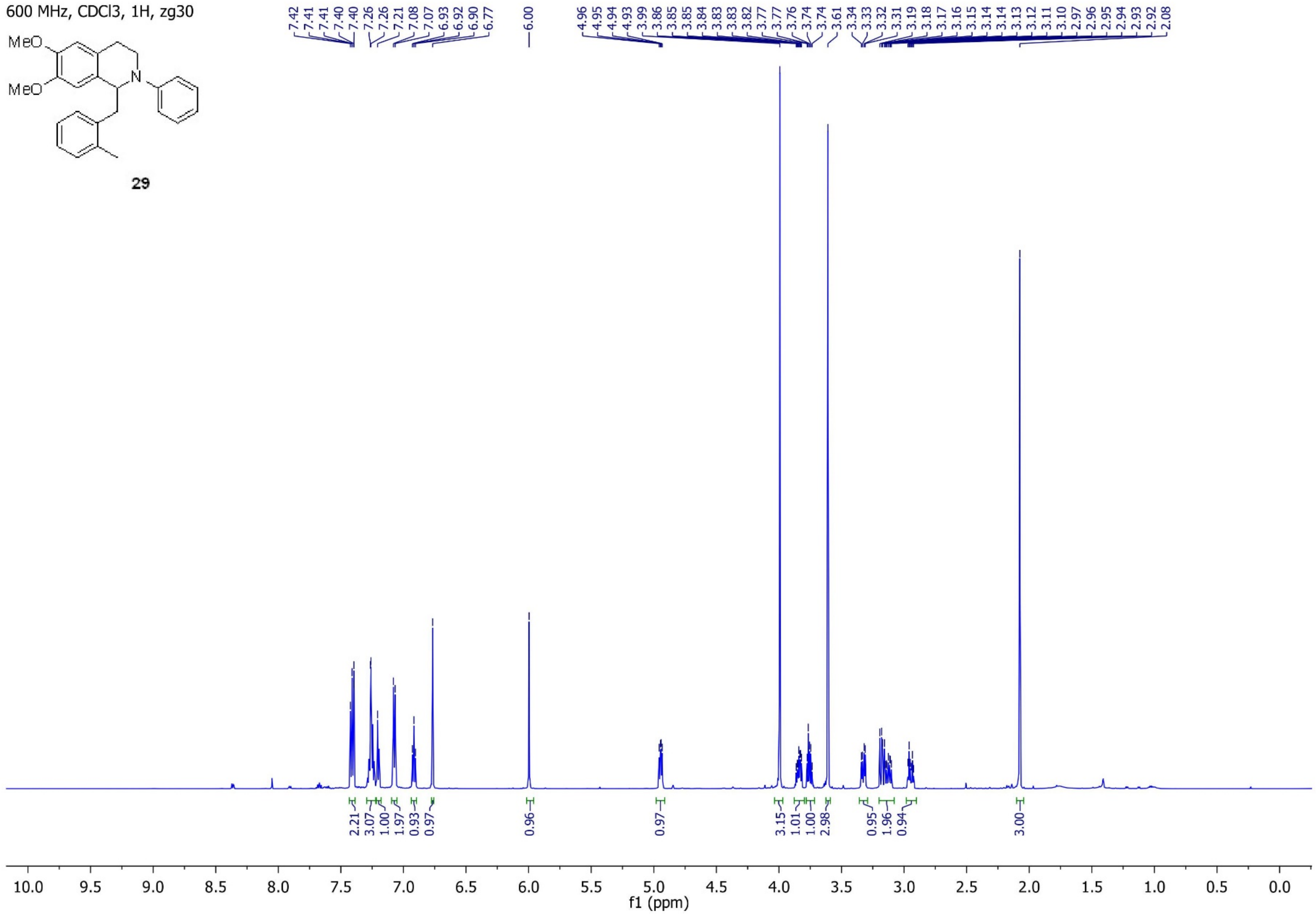

151 MHz, CDCl3, 13C, deptqgpsp

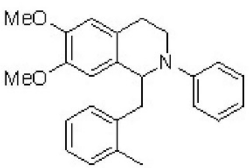

29

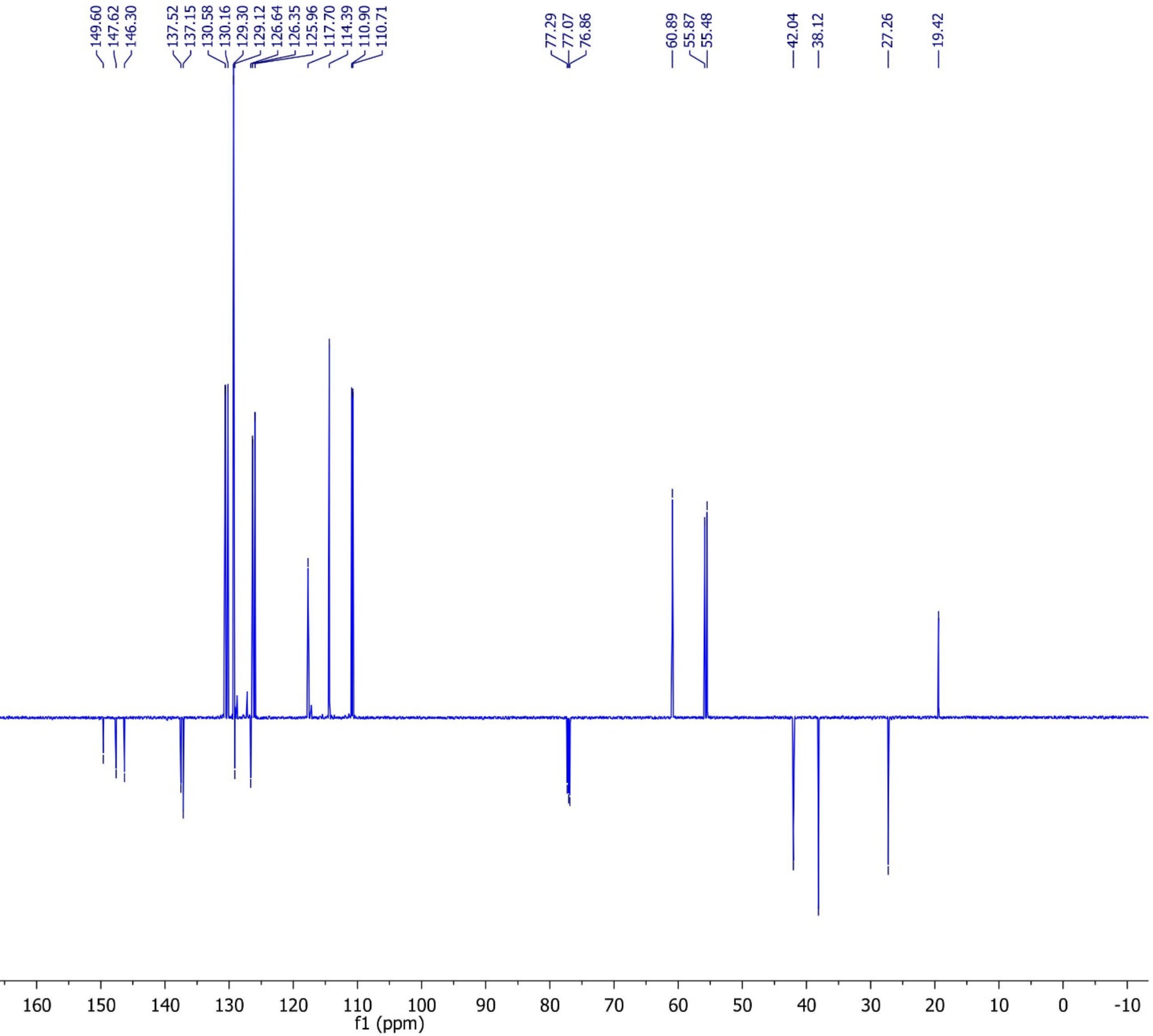

6,7-Dimethoxy-1-(4-fluorobenzyl)-2-phenyl-1,2,3,4-tetrahydroisoquinoline (30)

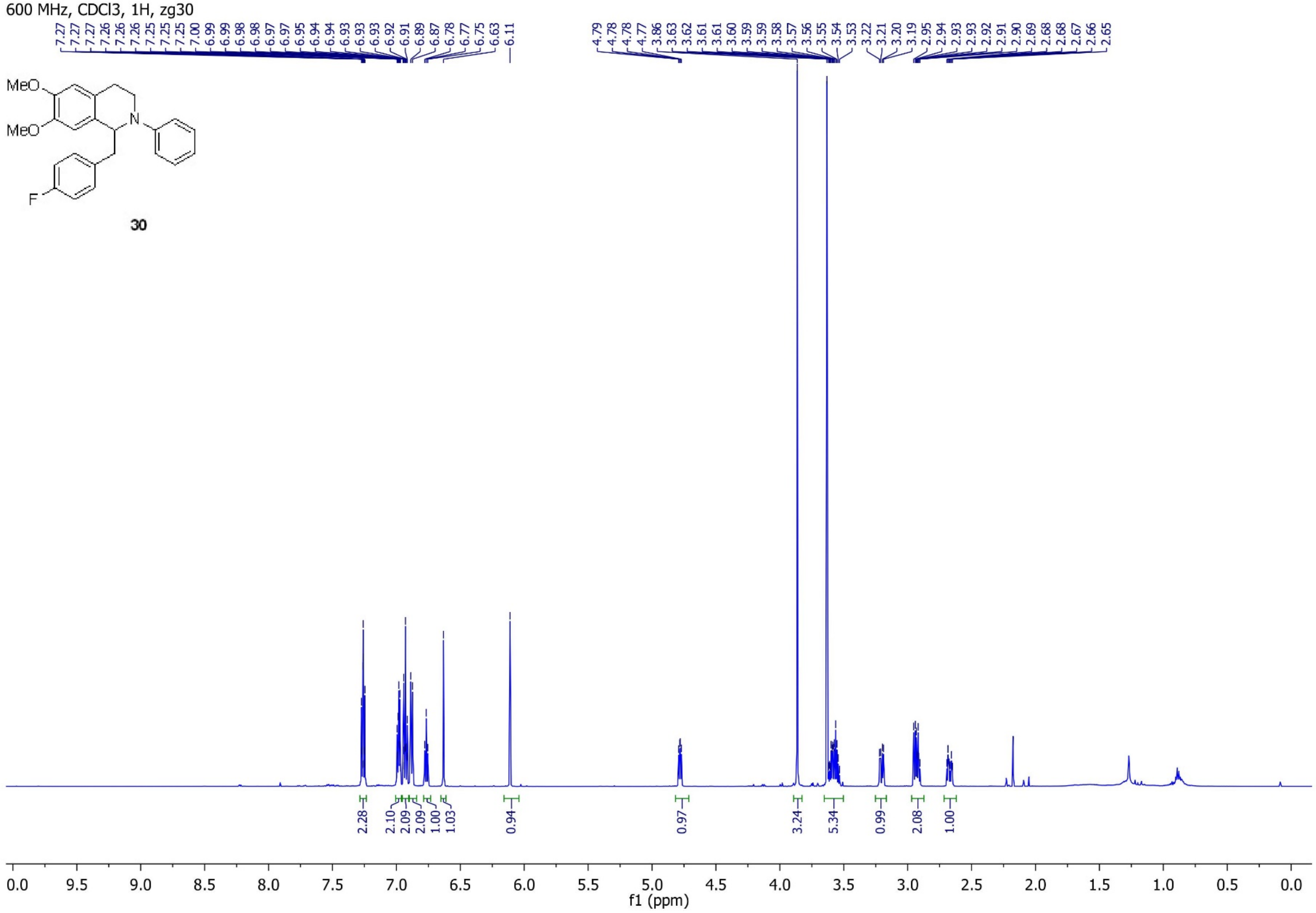

151 MHz, CDCl3, 13C, deptqgpsp

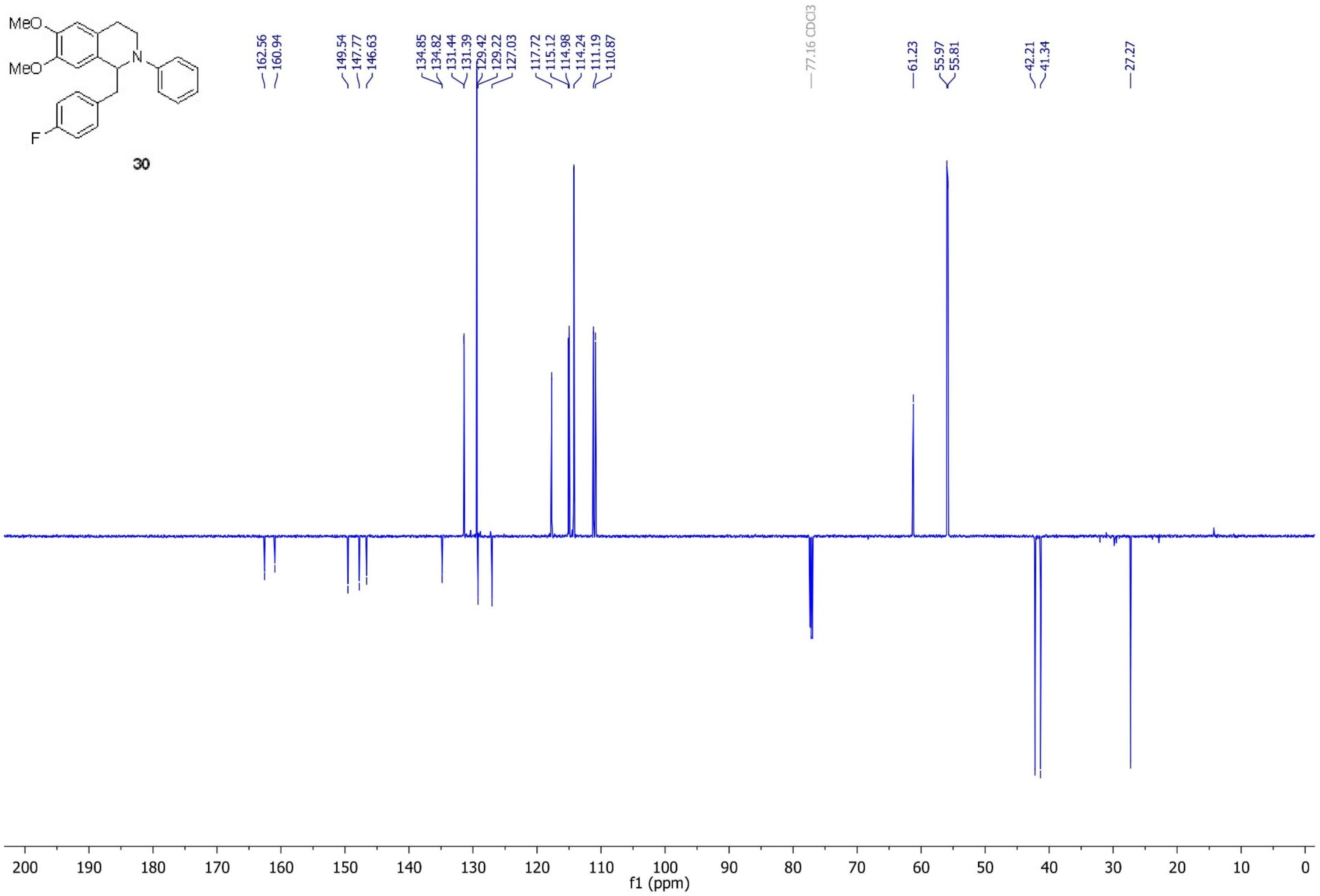

565 MHz, CDCl3, 19F, zgflqn

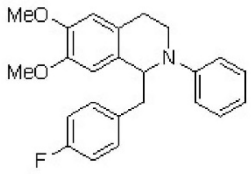

30

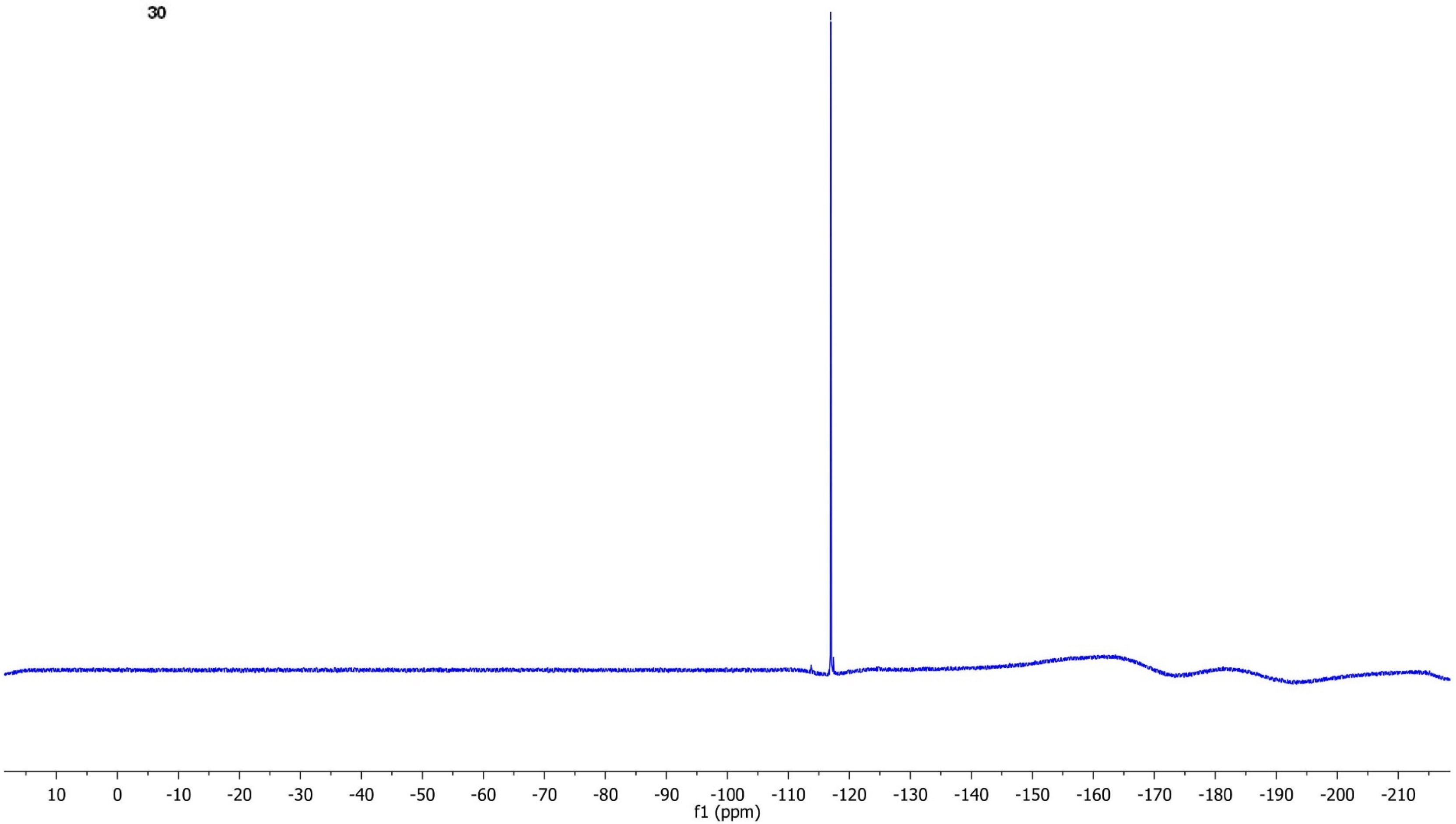

6,7-Dimethoxy-2-phenyl-1-(4-(trifluoromethyl)benzyl)-1,2,3,4-tetrahydroisoquinoline (31)

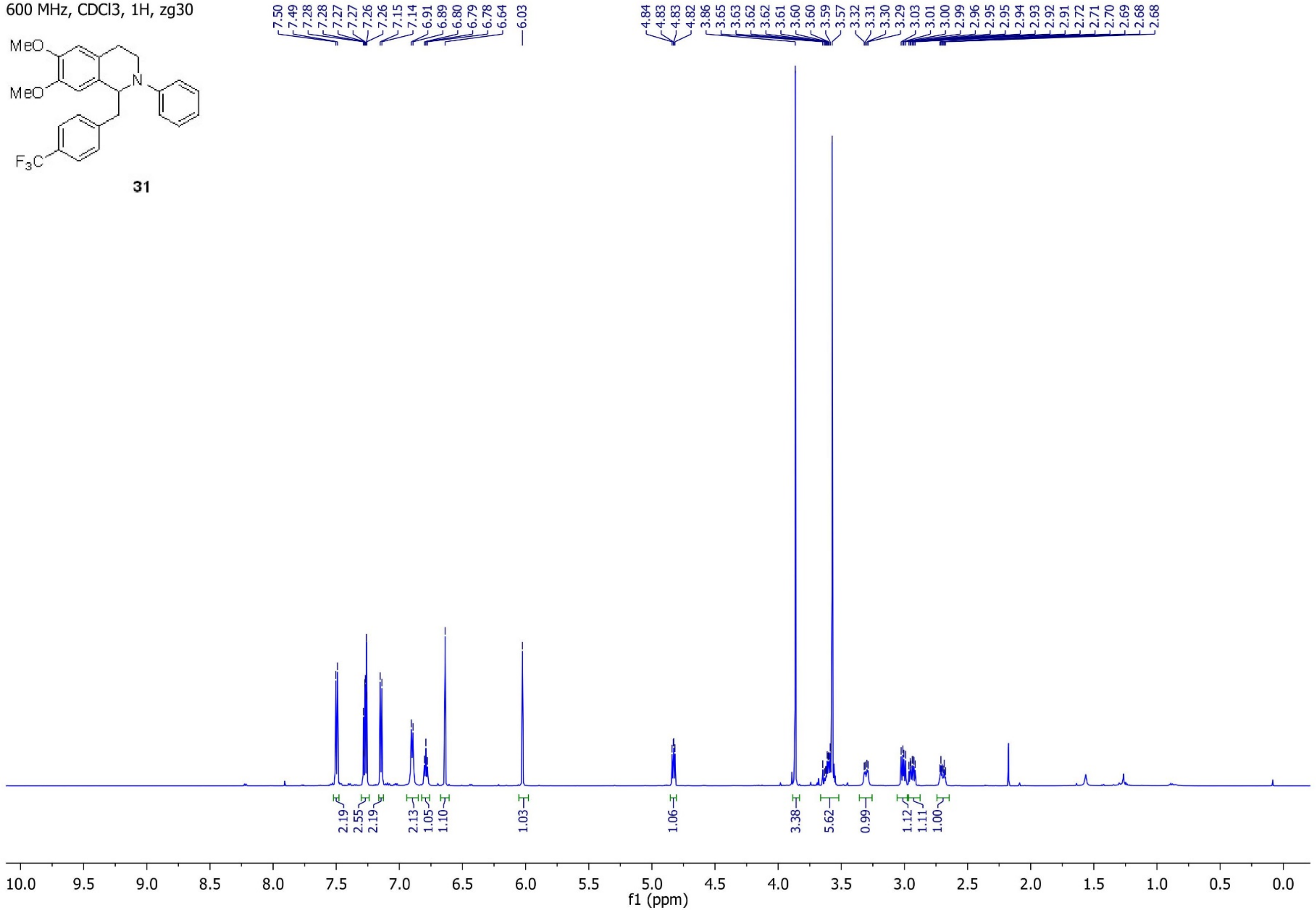

151 MHz, CDCl3, 13C, deptqgpsp

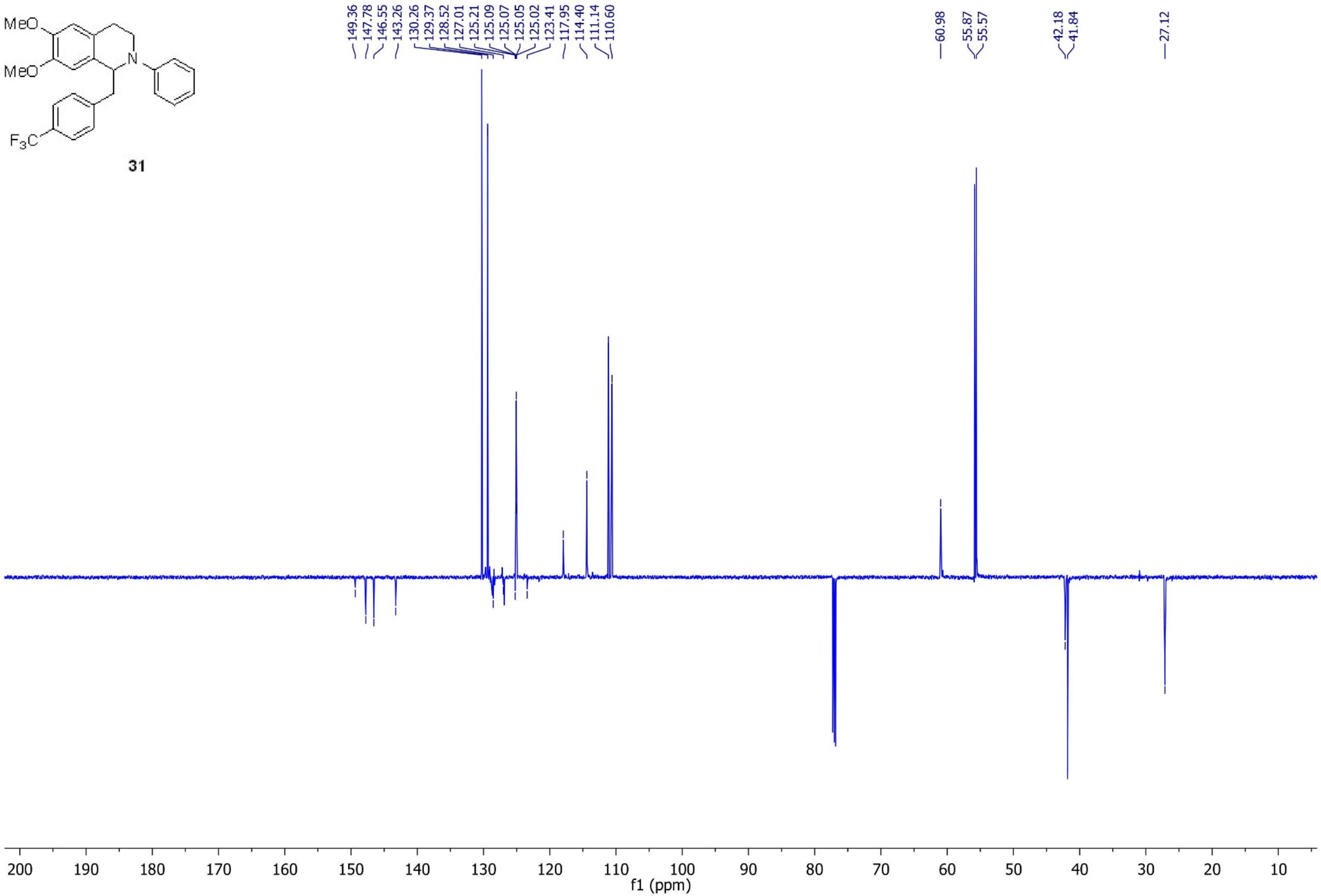

565 MHz, CDCl3, 19F, zgflqn

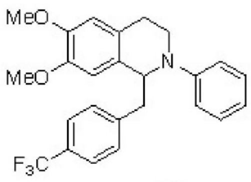

31

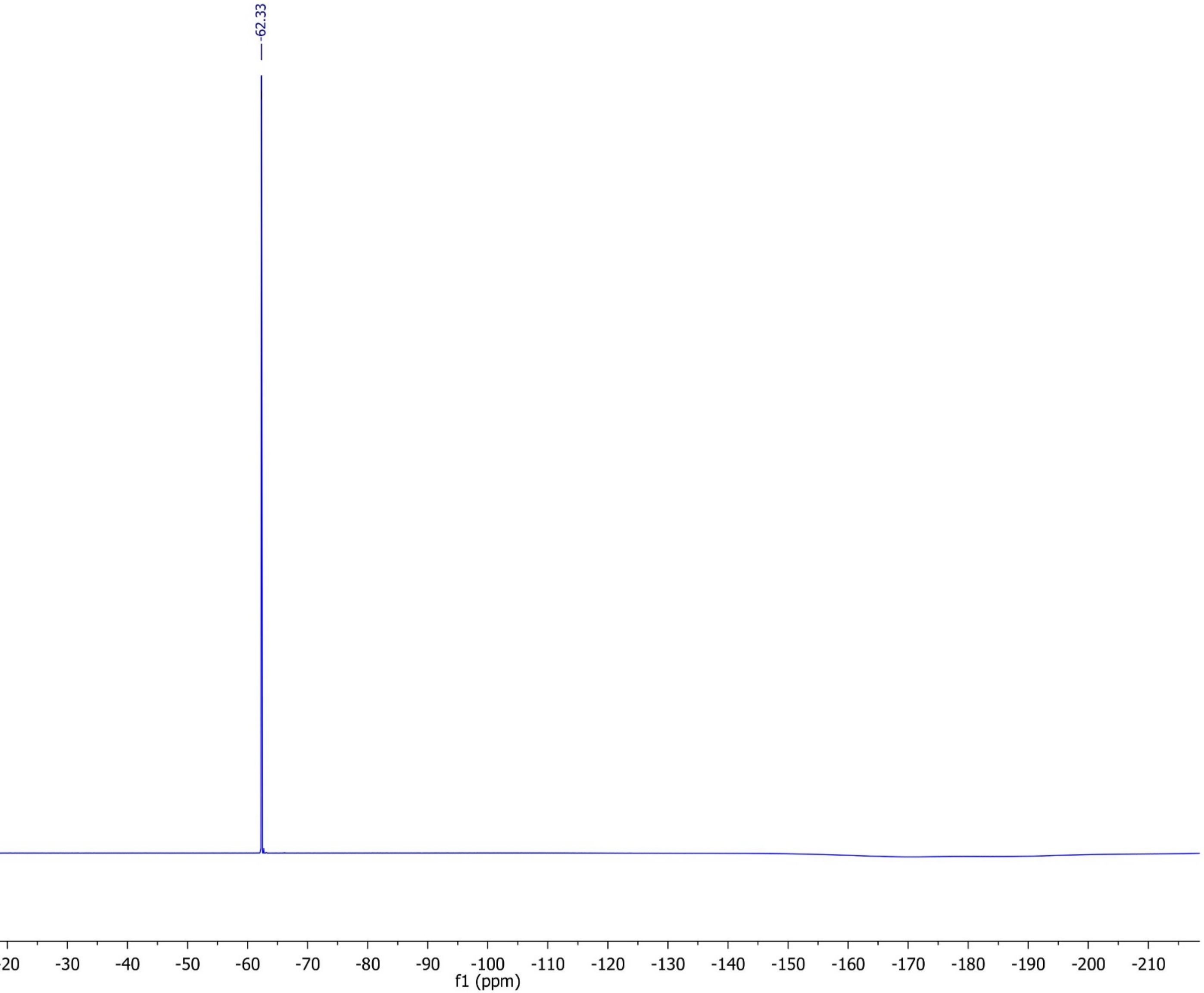

6,7-Dimethoxy-1-(4-methoxybenzyl)-2-phenyl-1,2,3,4-tetrahydroisoquinoline (32)

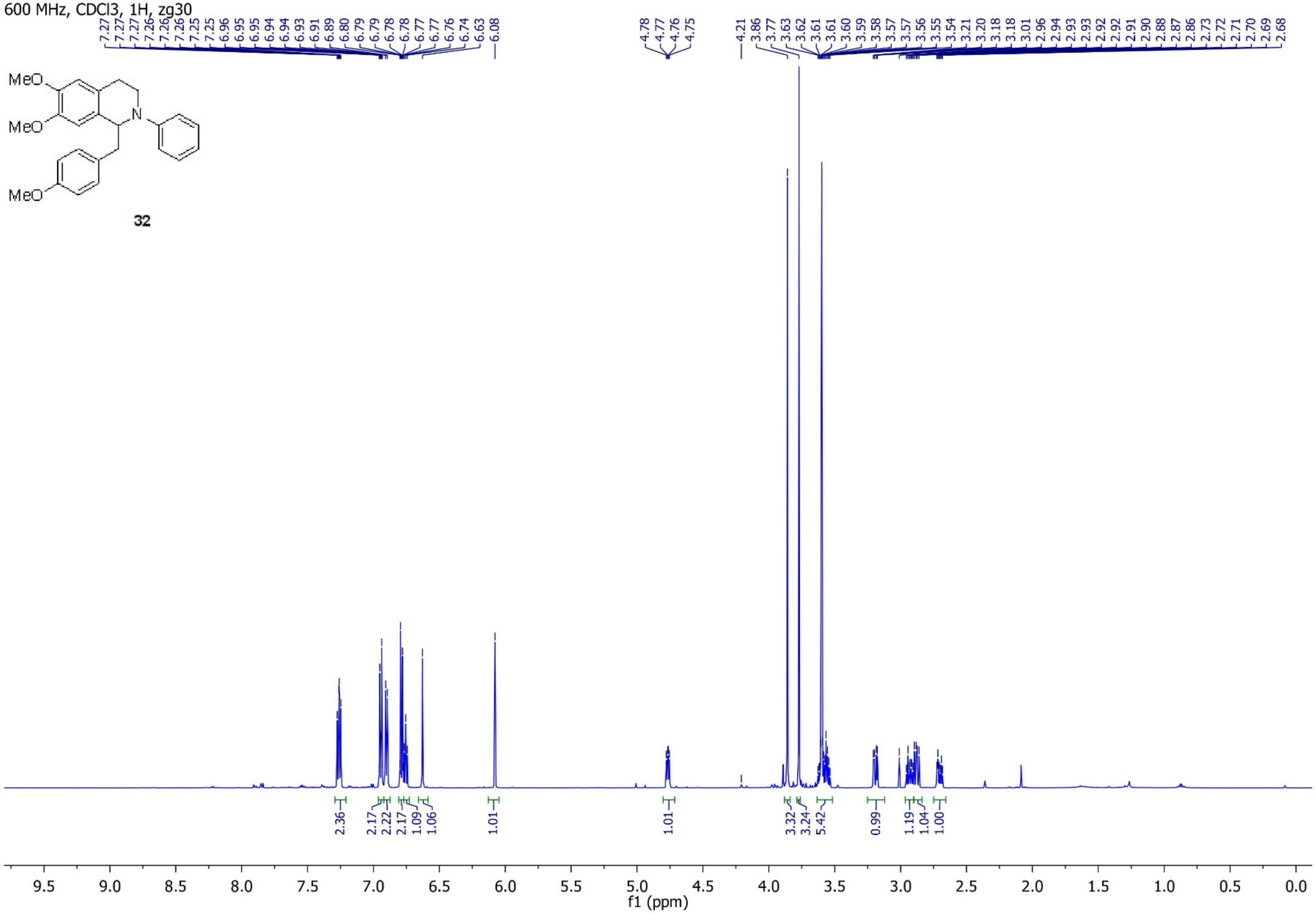

32

151 MHz, CDCl3, 13C, deptqgpsp

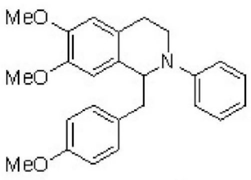

32

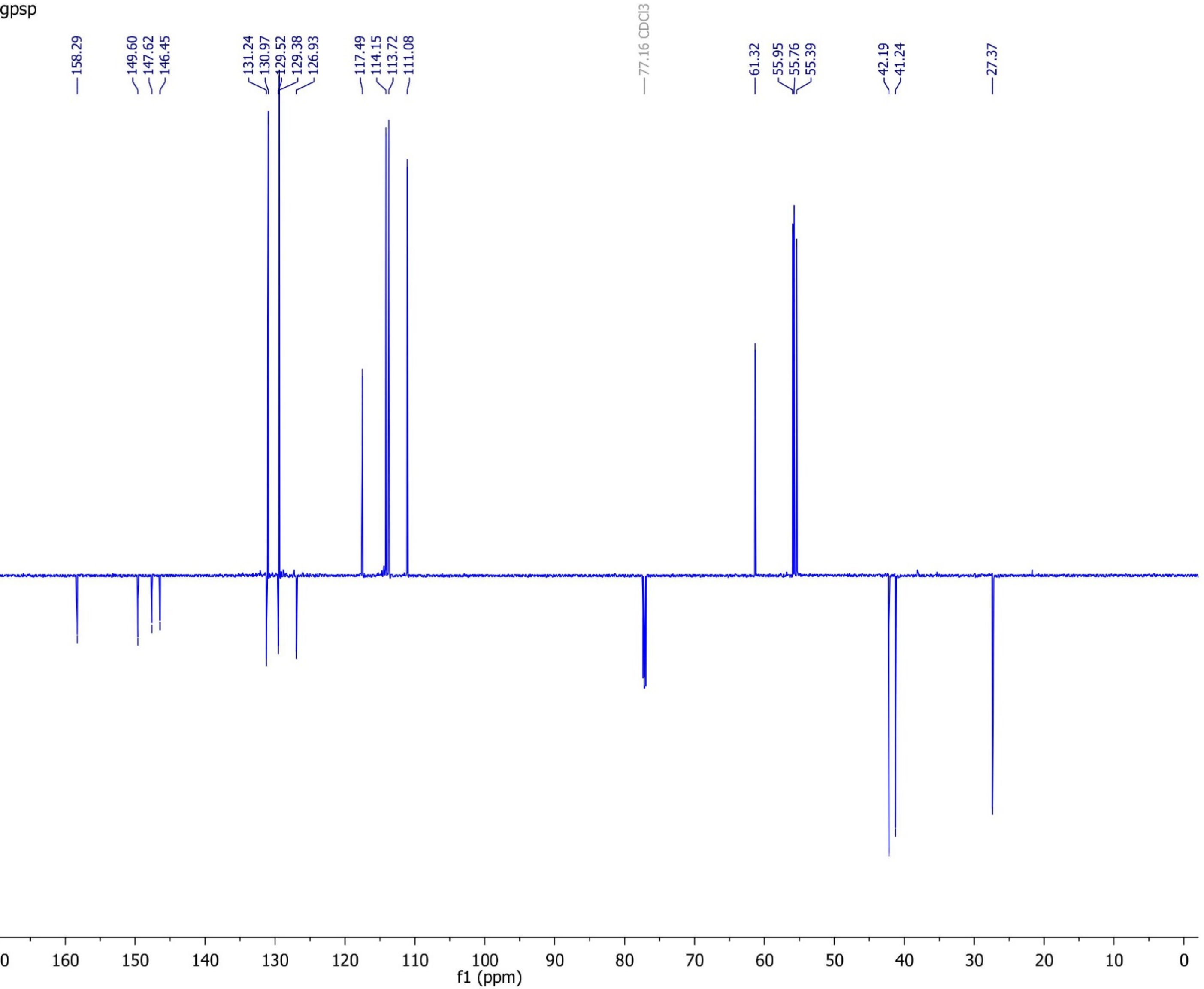

1-Isopropyl-2-phenyl-1,2,3,4-tetrahydroisoquinoline (34)

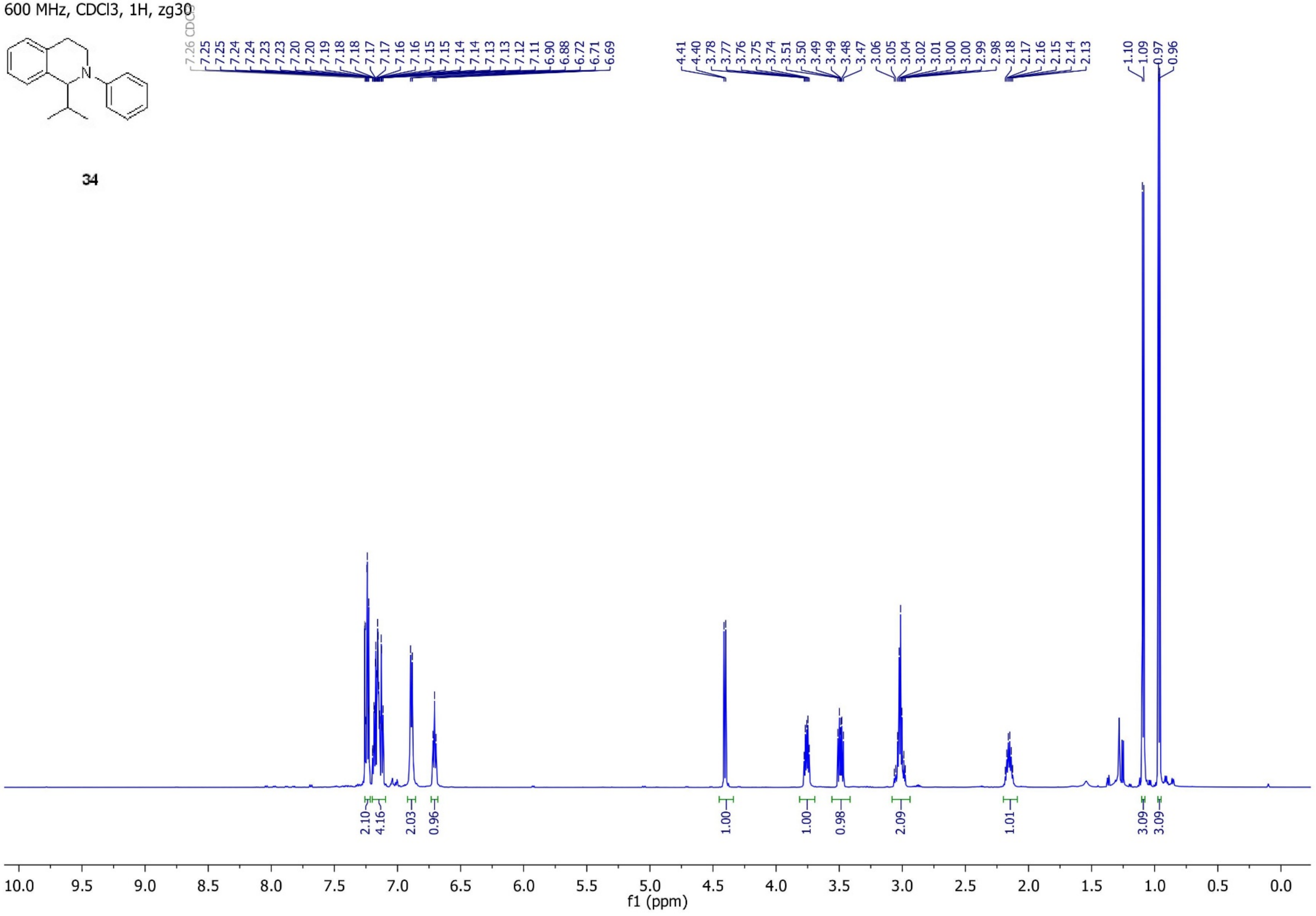

151 MHz, CDCl3, 13C, deptqgpsp

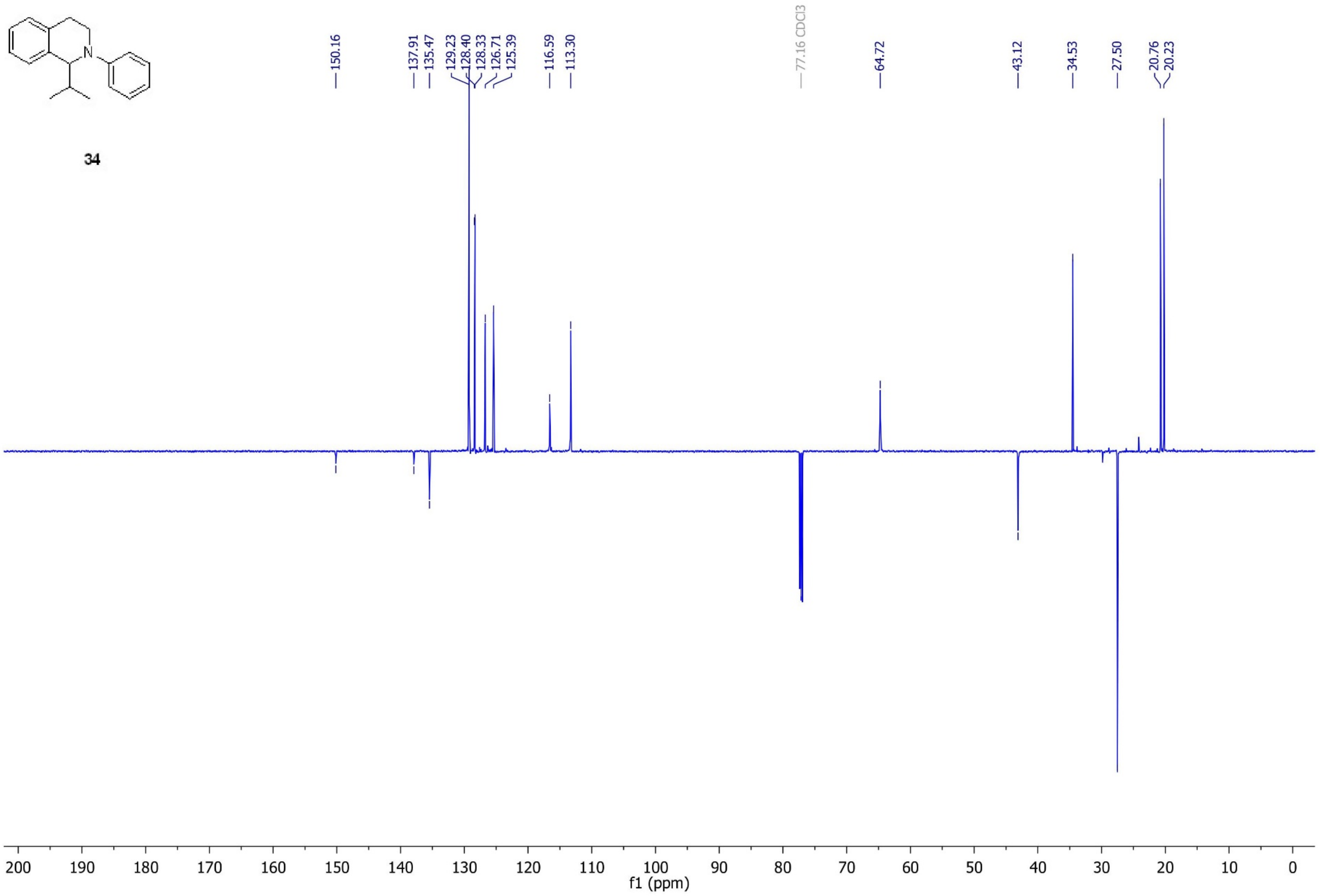

1-(*sec*-Butyl)-2-phenyl-1,2,3,4-tetrahydroisoquinoline (35)

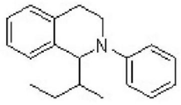

35

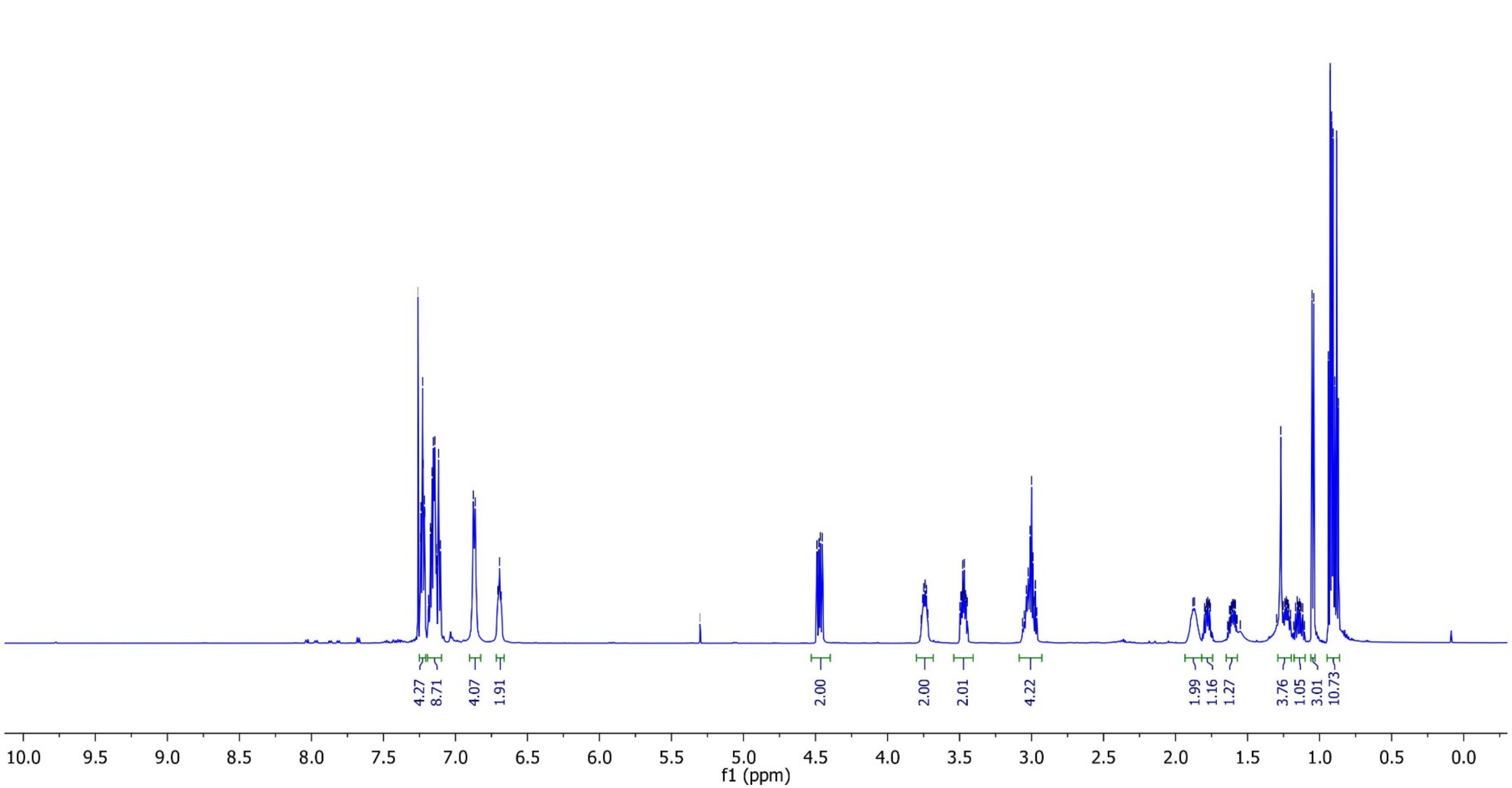

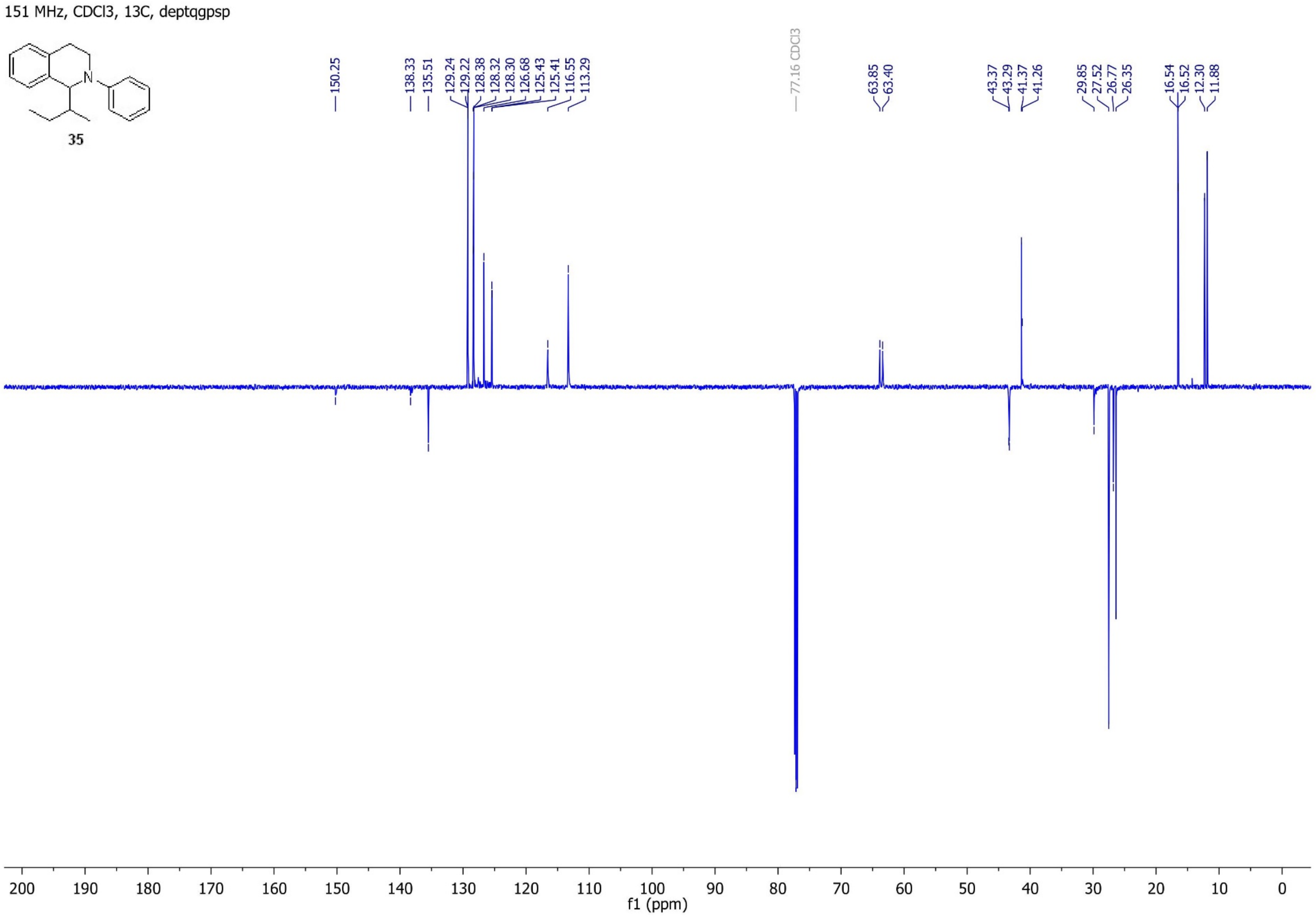
151 MHz, CDCl3, 13C, deptqgpsp
N
150.25
138.33
135.51
129.24
129.22
128.38
128.32
128.30
126.68
125.43
125.41
116.55
113.29
77.16 CDCl3
63.85
63.40
43.37
43.29
41.37
41.26
29.85
27.52
26.77
26.35
16.54
16.52
12.30
11.88
200 190 180 170 160 150 140 130 120 110 100 90 80 70 60 50 40 30 20 10 0
f1 (ppm)

35

1-Cyclohexyl-2-phenyl-1,2,3,4-tetrahydroisoquinoline (36)

600 MHz, CDCl3, 1H, zg30

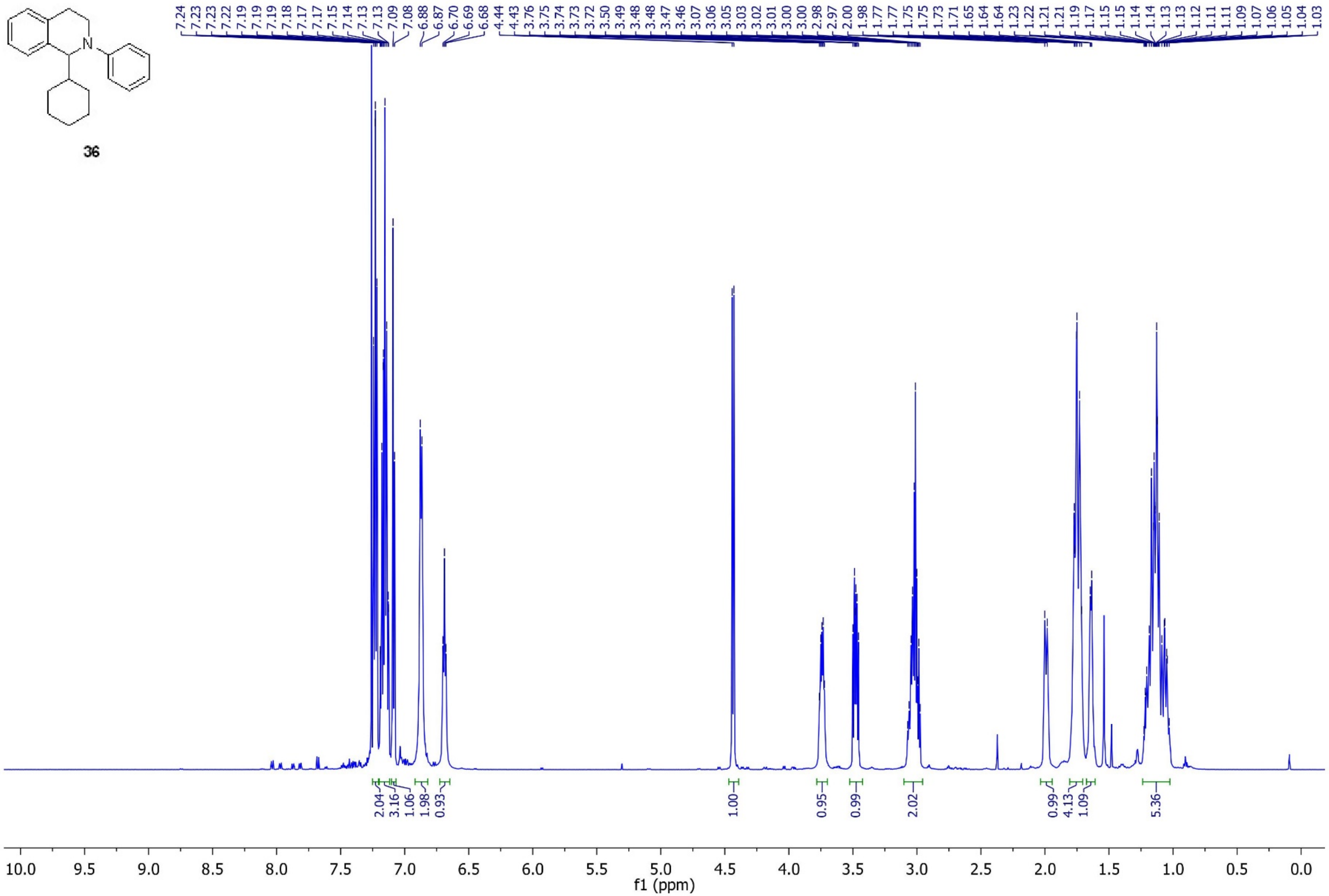

151 MHz, CDCl3, 13C, deptqgpsp

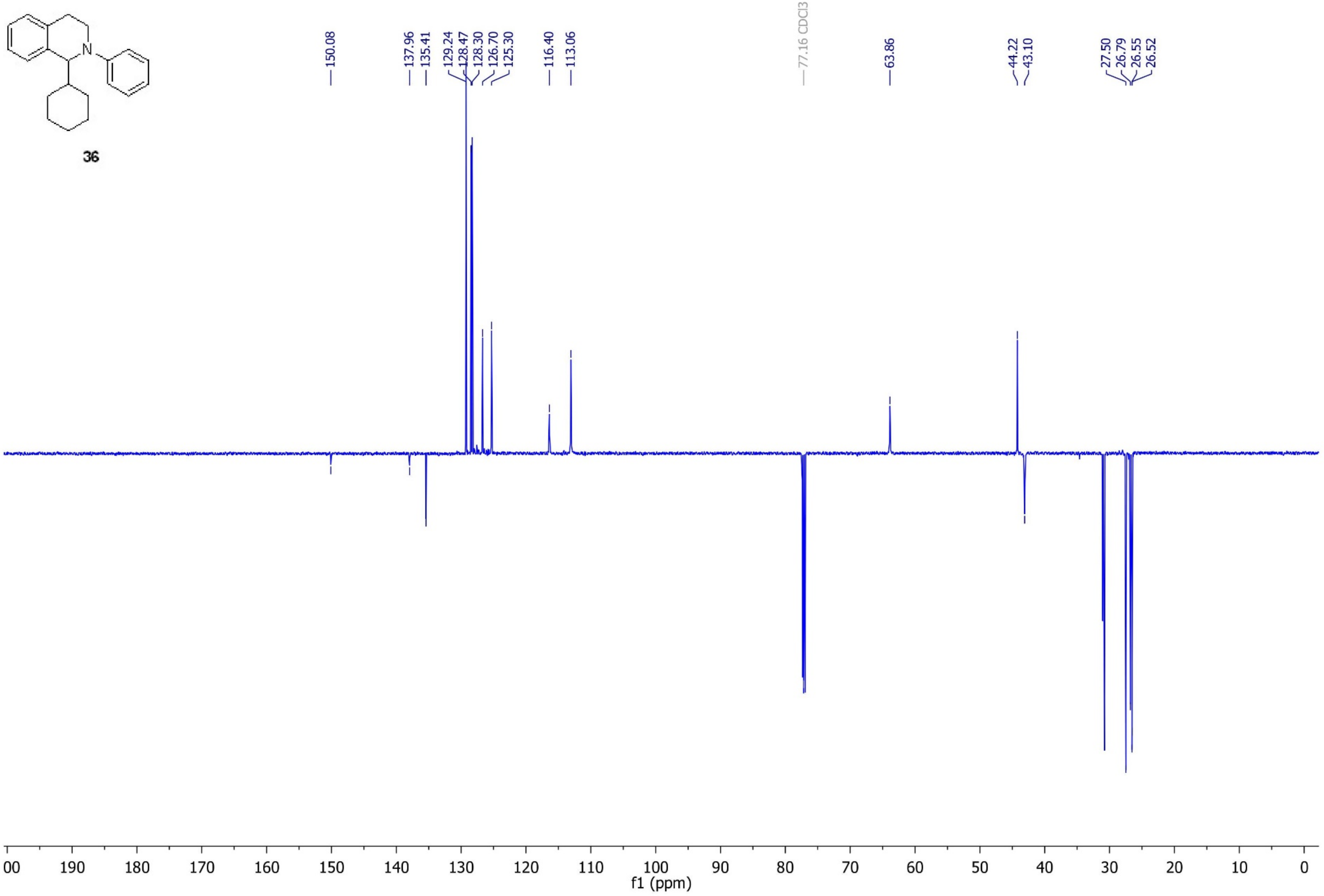

1-Allyl-2-phenyl-1,2,3,4-tetrahydroisoquinoline (37)

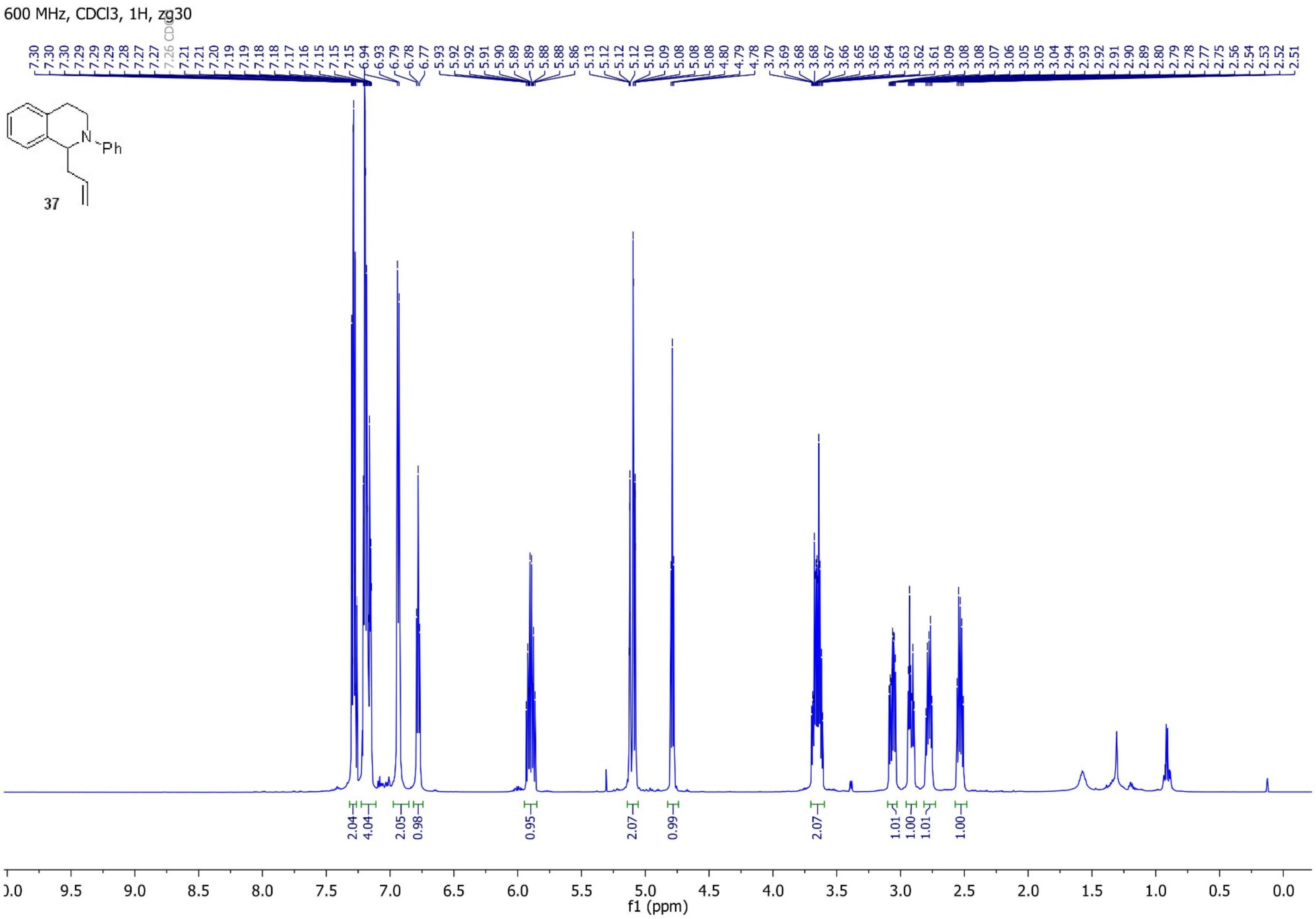

151 MHz, CDCl3, 13C, jmod

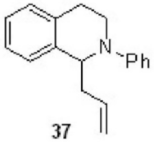

37

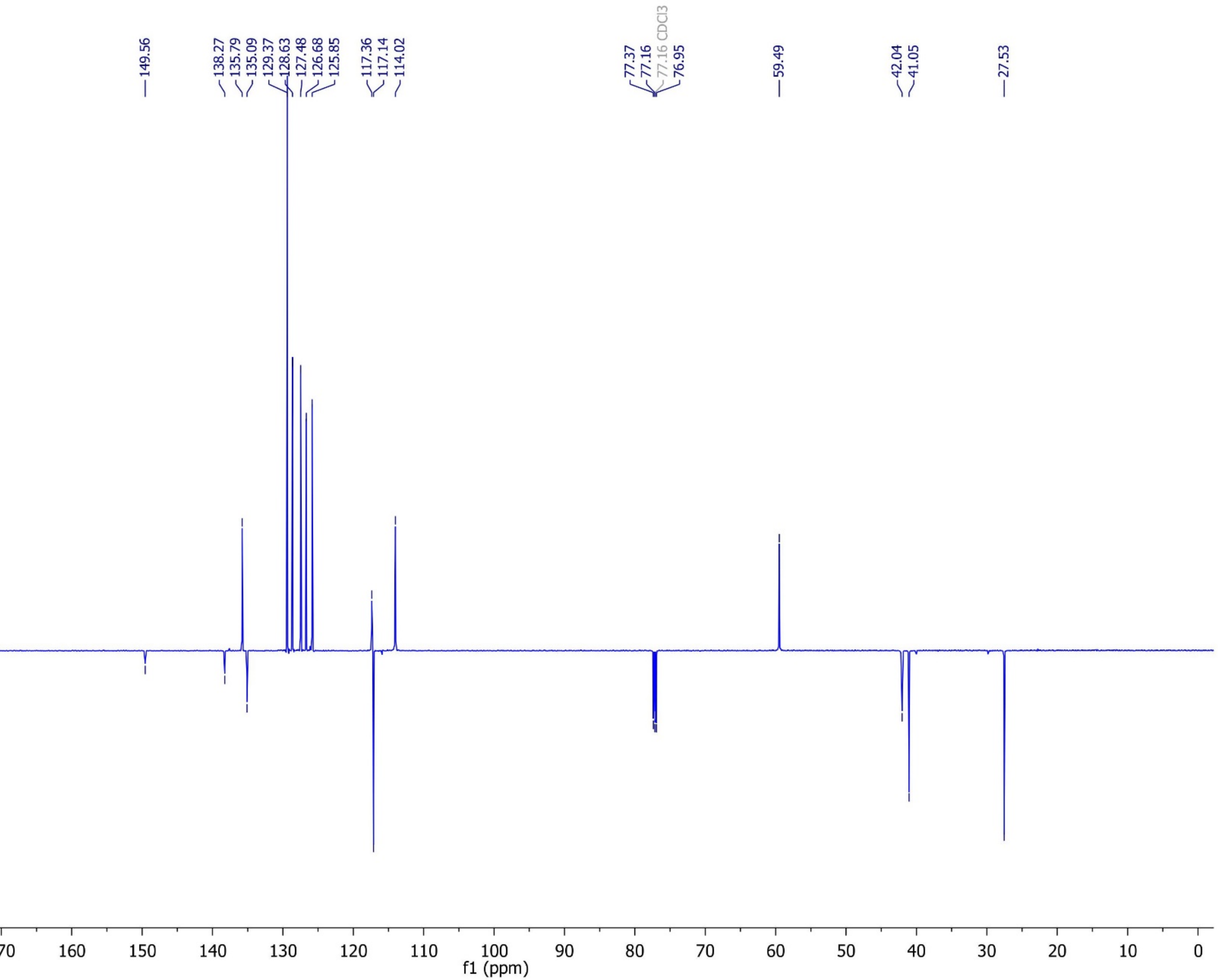

1-Allyl-6,7-dimethoxy-2-phenyl-1,2,3,4-tetrahydroisoquinoline (38)

600 MHz, CDCl3, 1H, zg30

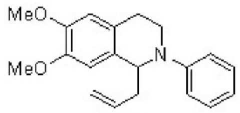

38

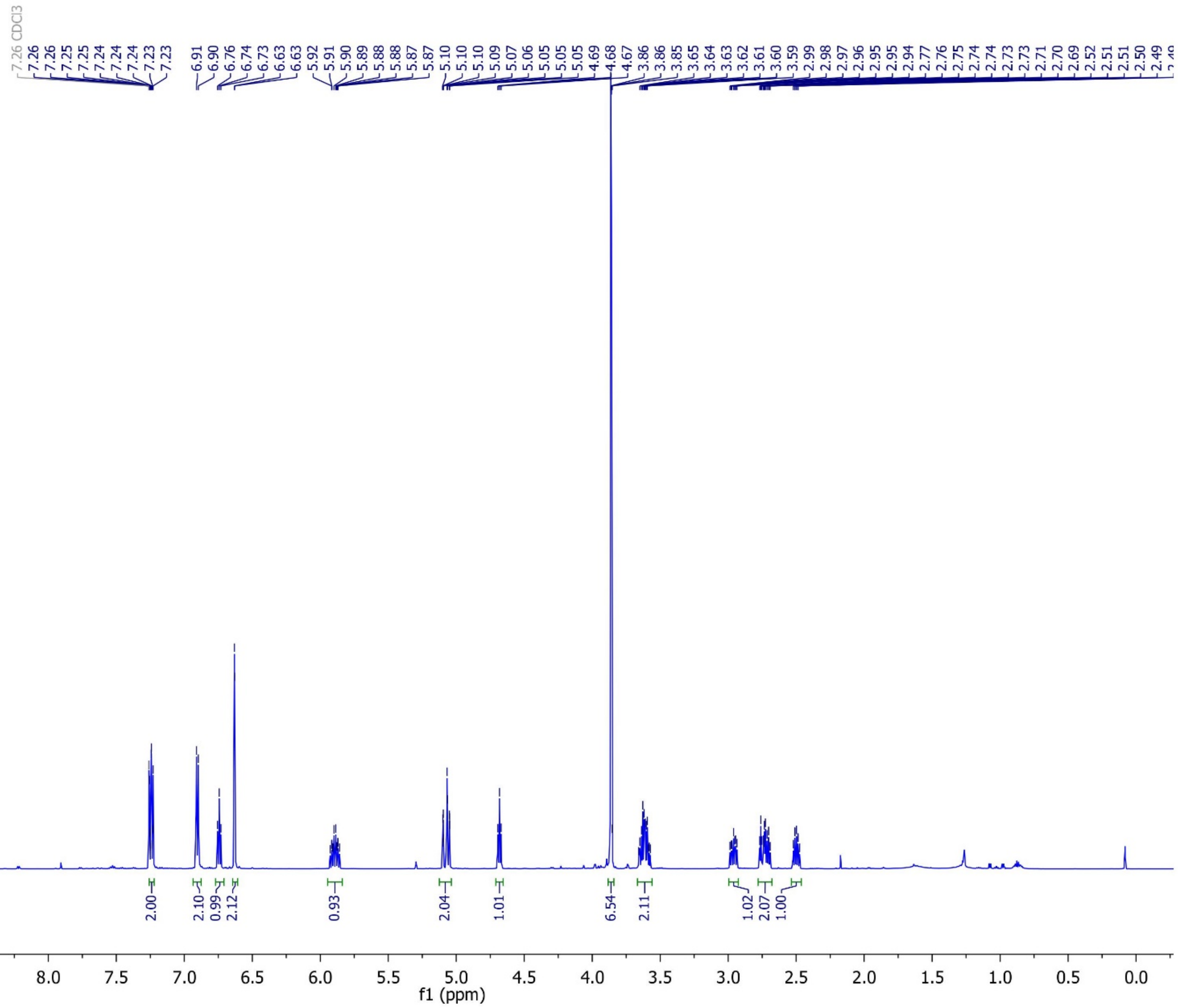

151 MHz, CDCl3, 13C, deptqgpsp

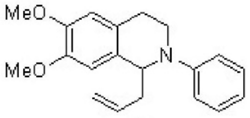

38

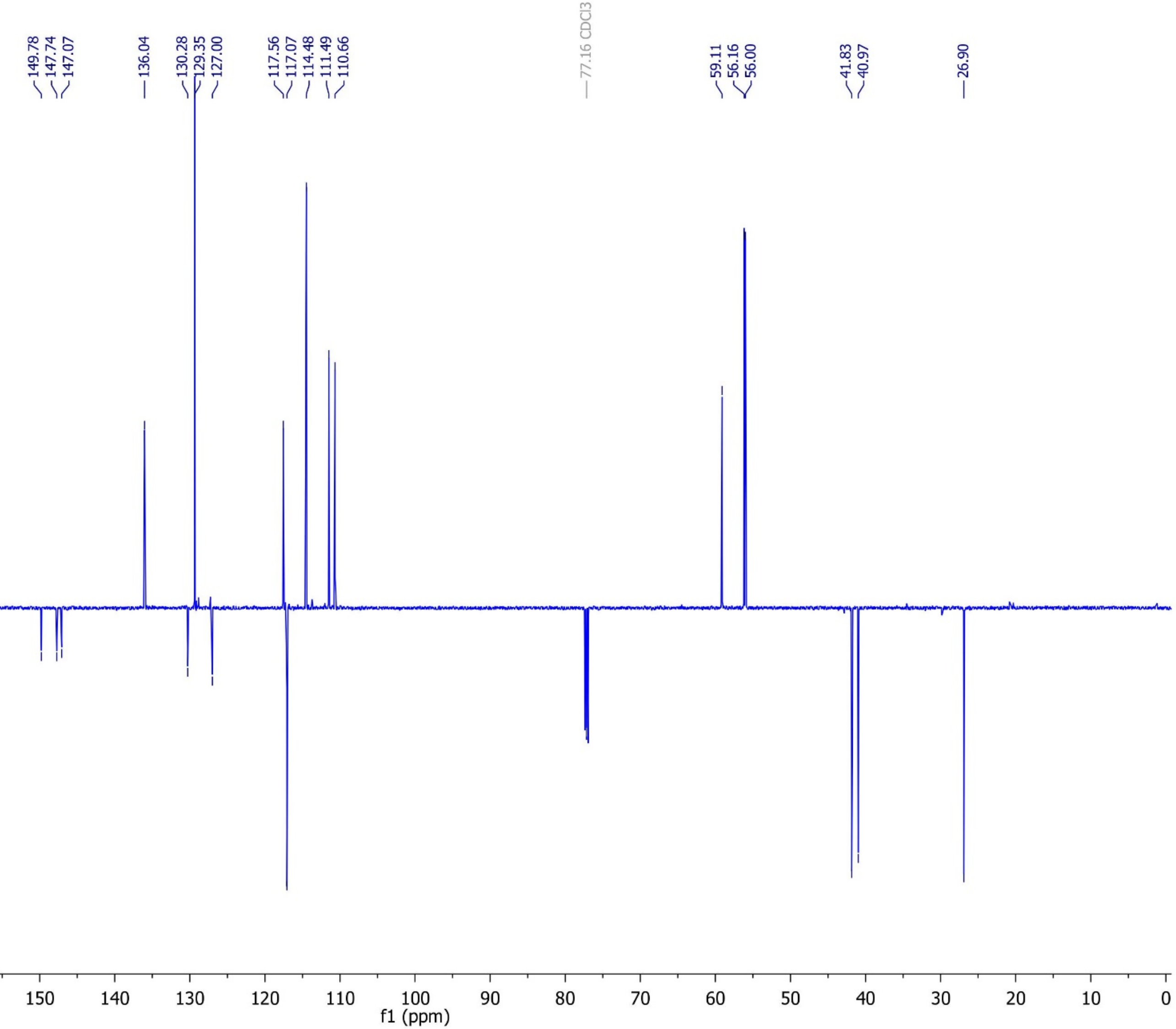

1-Benzyl-1,2,3,4-tetrahydroisoquinoline (33)

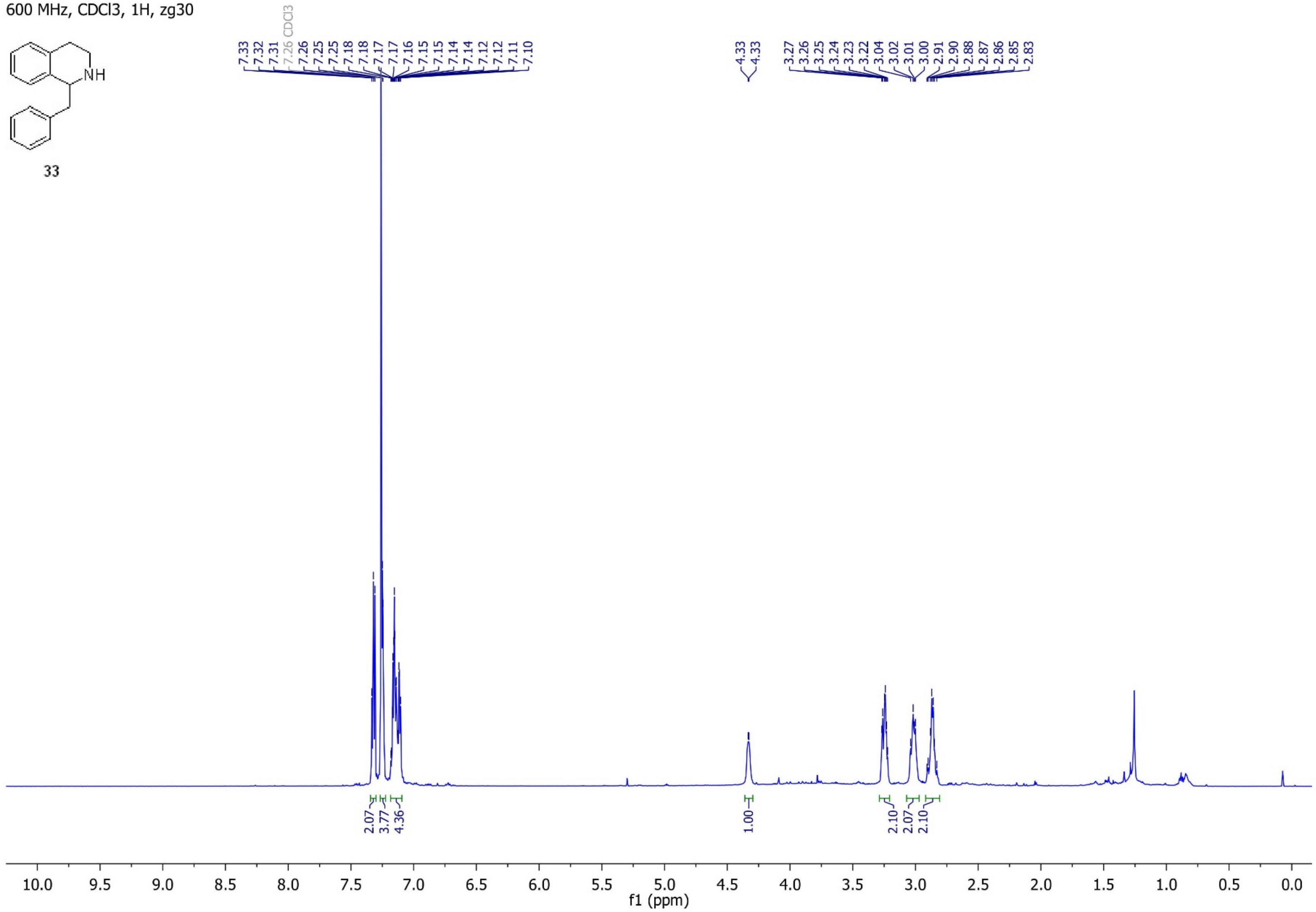

151 MHz, CDCl3, 13C, zgpg30

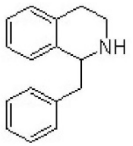

33

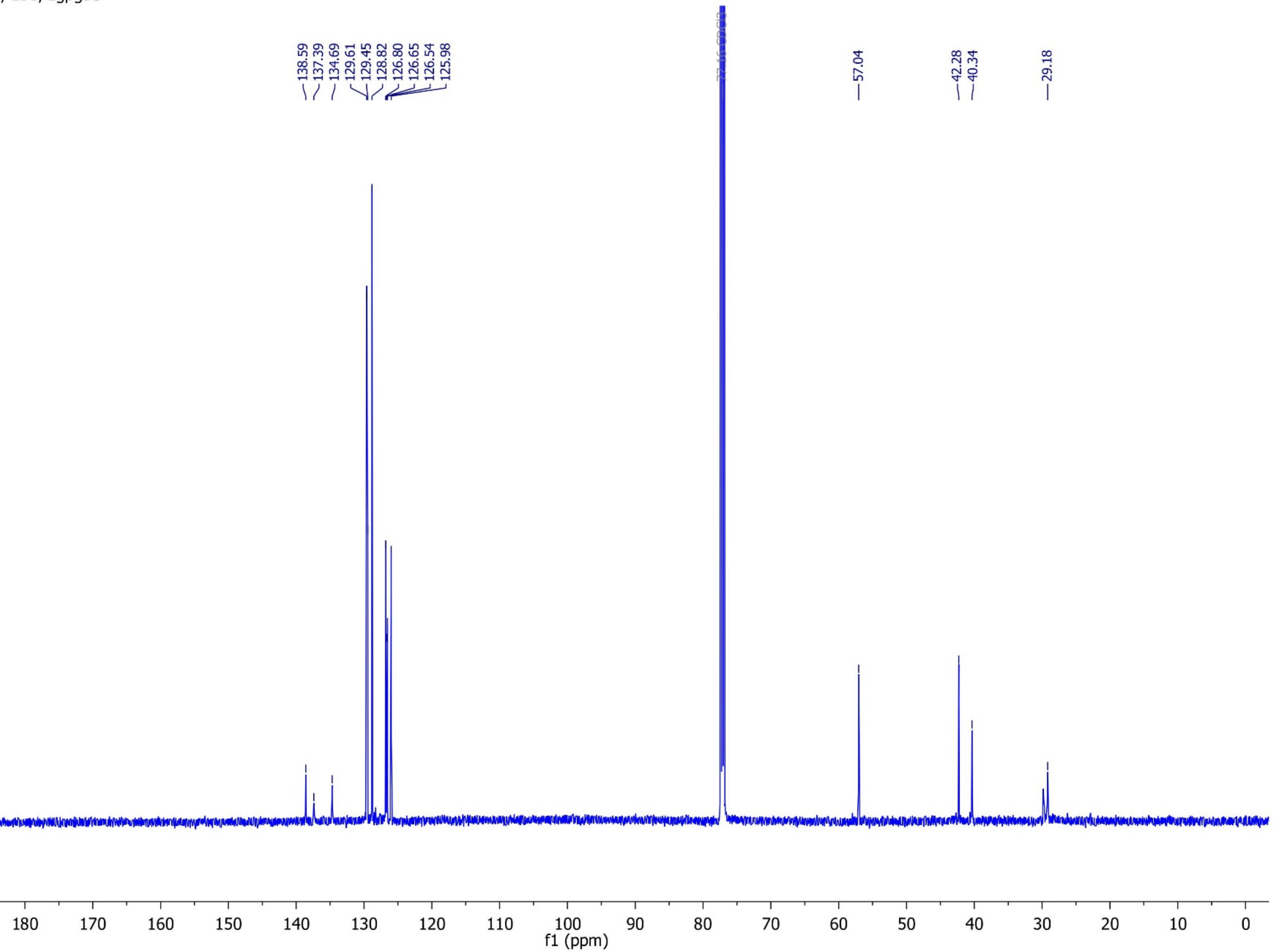

2-Phenyl-1,2,3,4-tetrahydroisoquinoline (1)

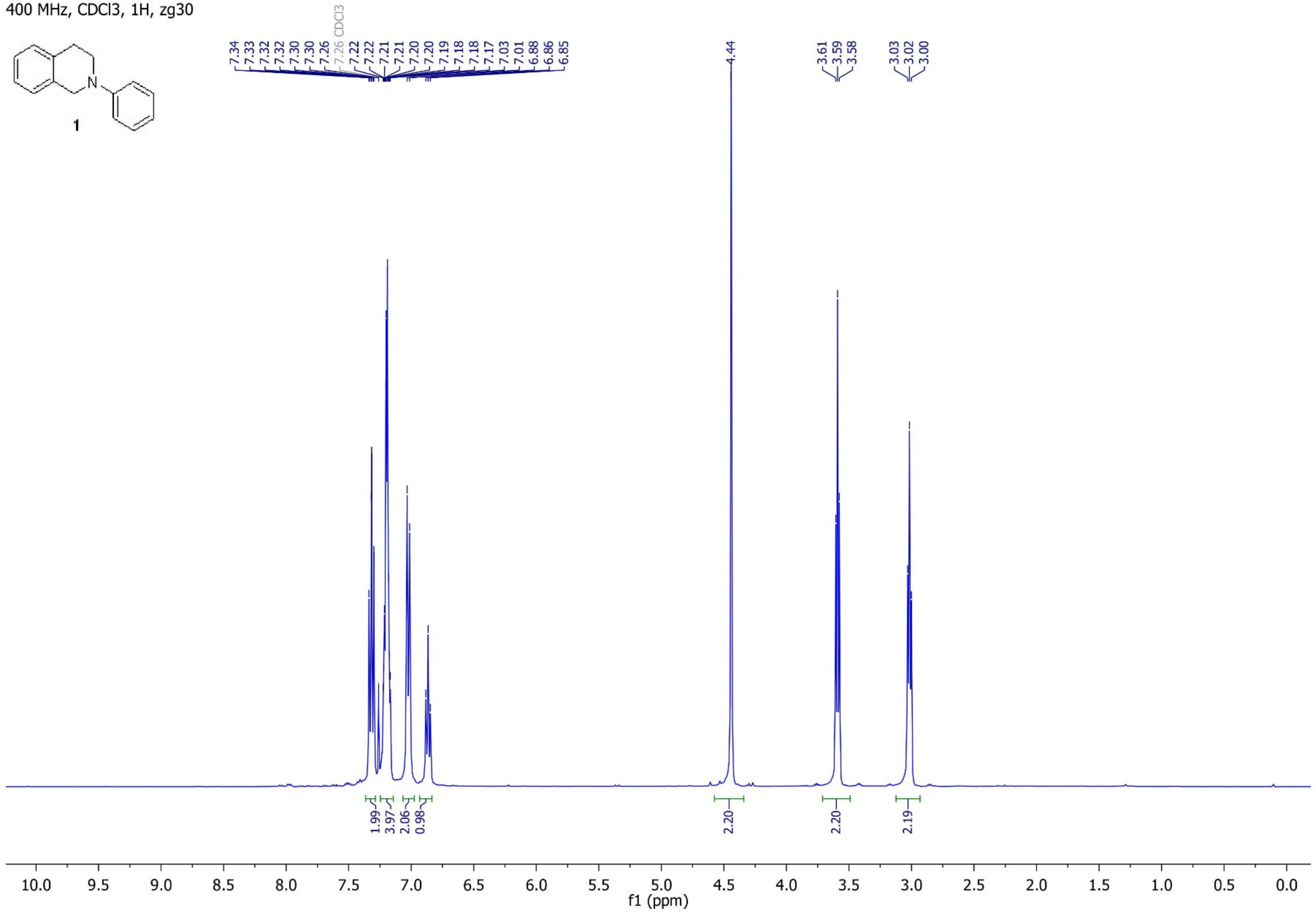

101 MHz, CDCl3, 13C, zgpg30

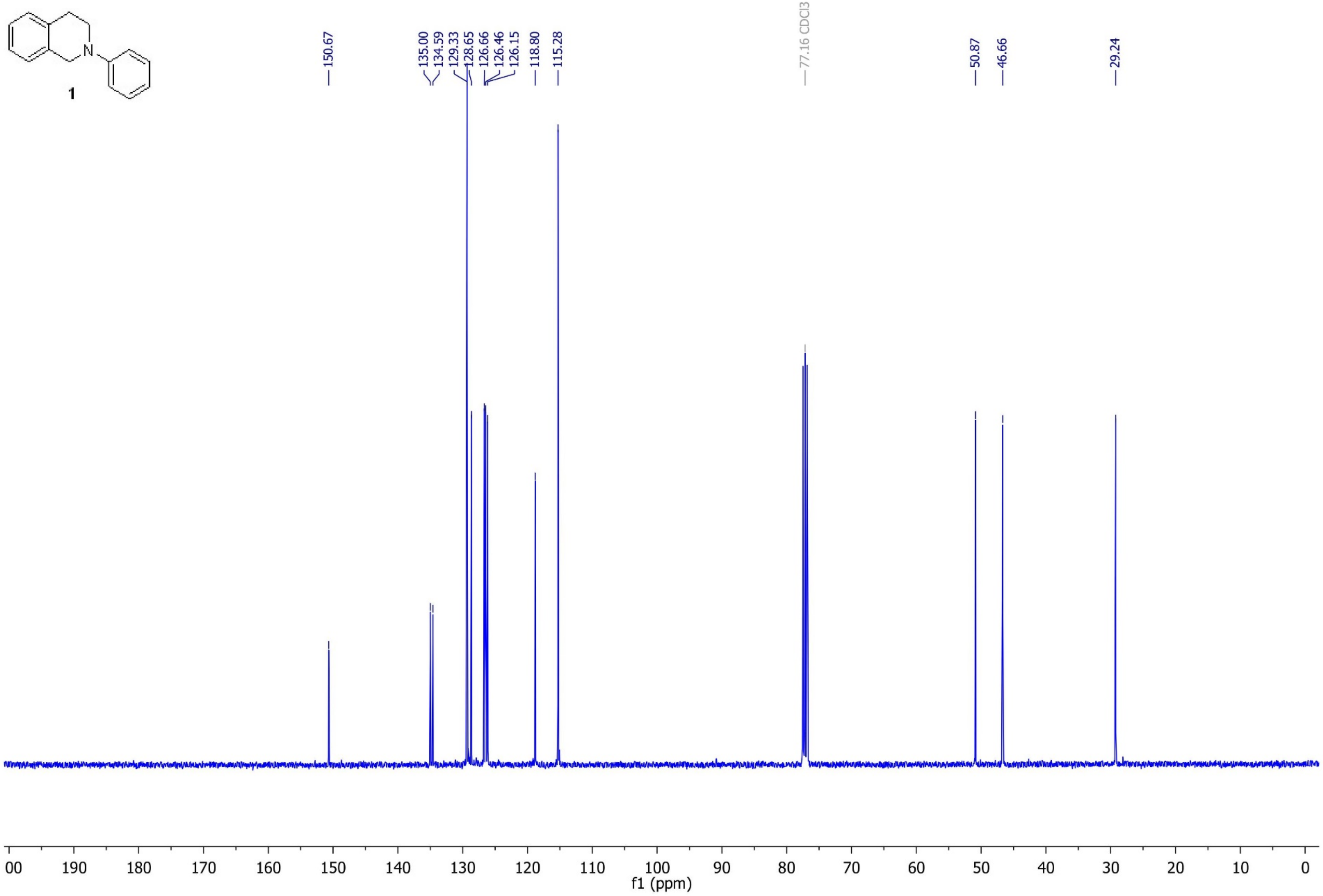

2-(4-Chlorophenyl)-1,2,3,4-tetrahydroisoquinoline (14)

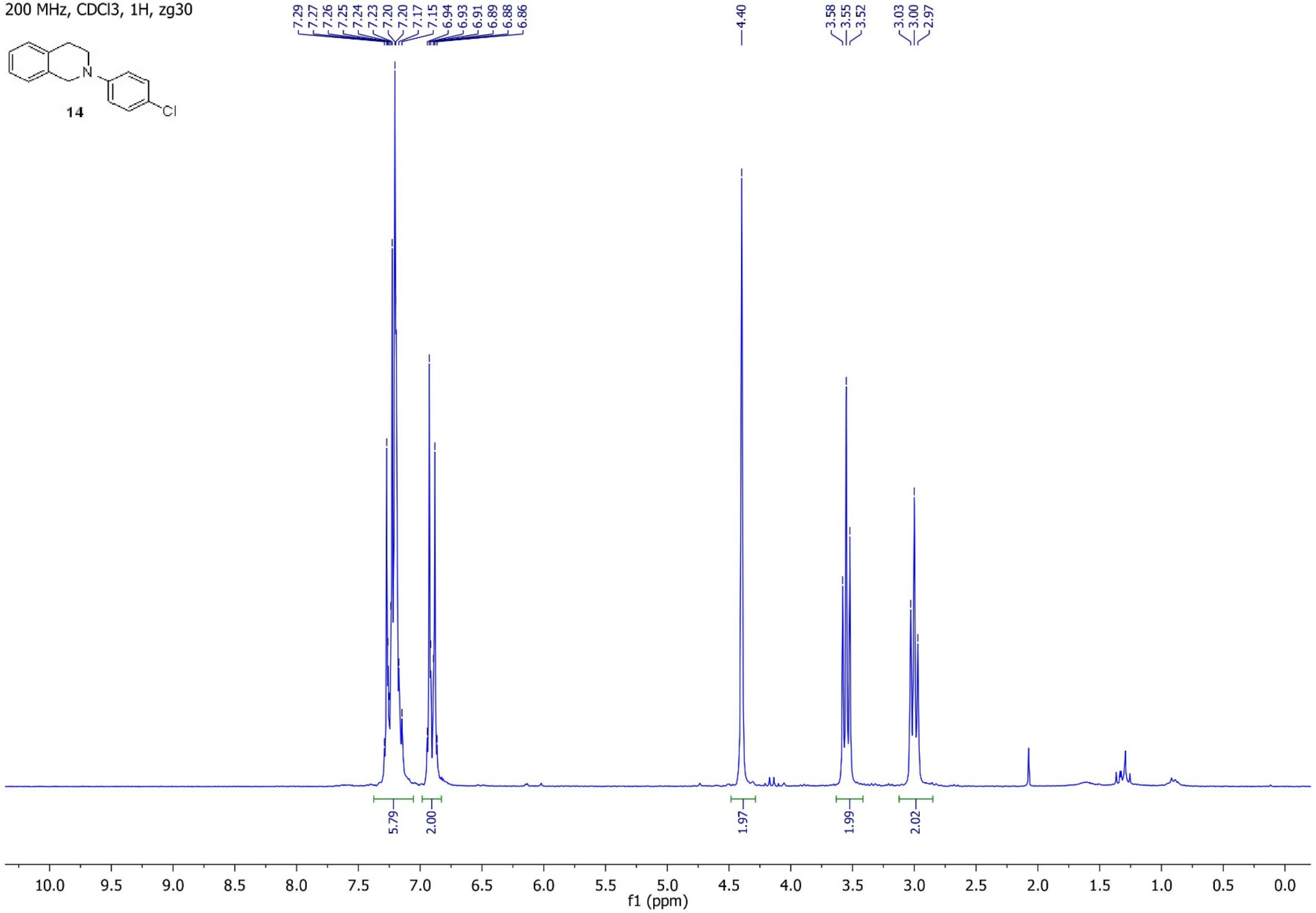

50 MHz, CDCl3, 13C, jmod

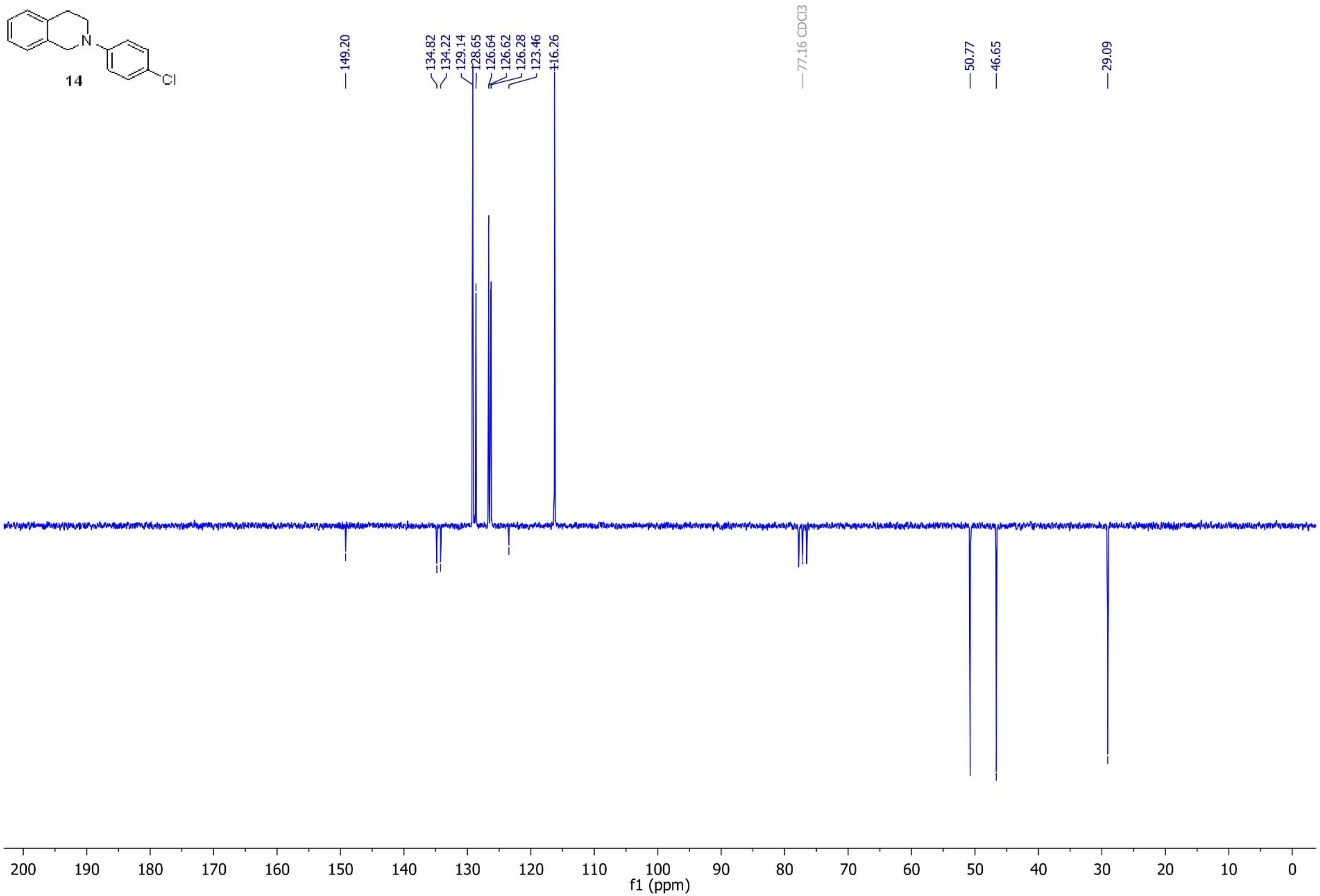

2-(4-Bromophenyl)-1,2,3,4-tetrahydroisoquinoline (15)

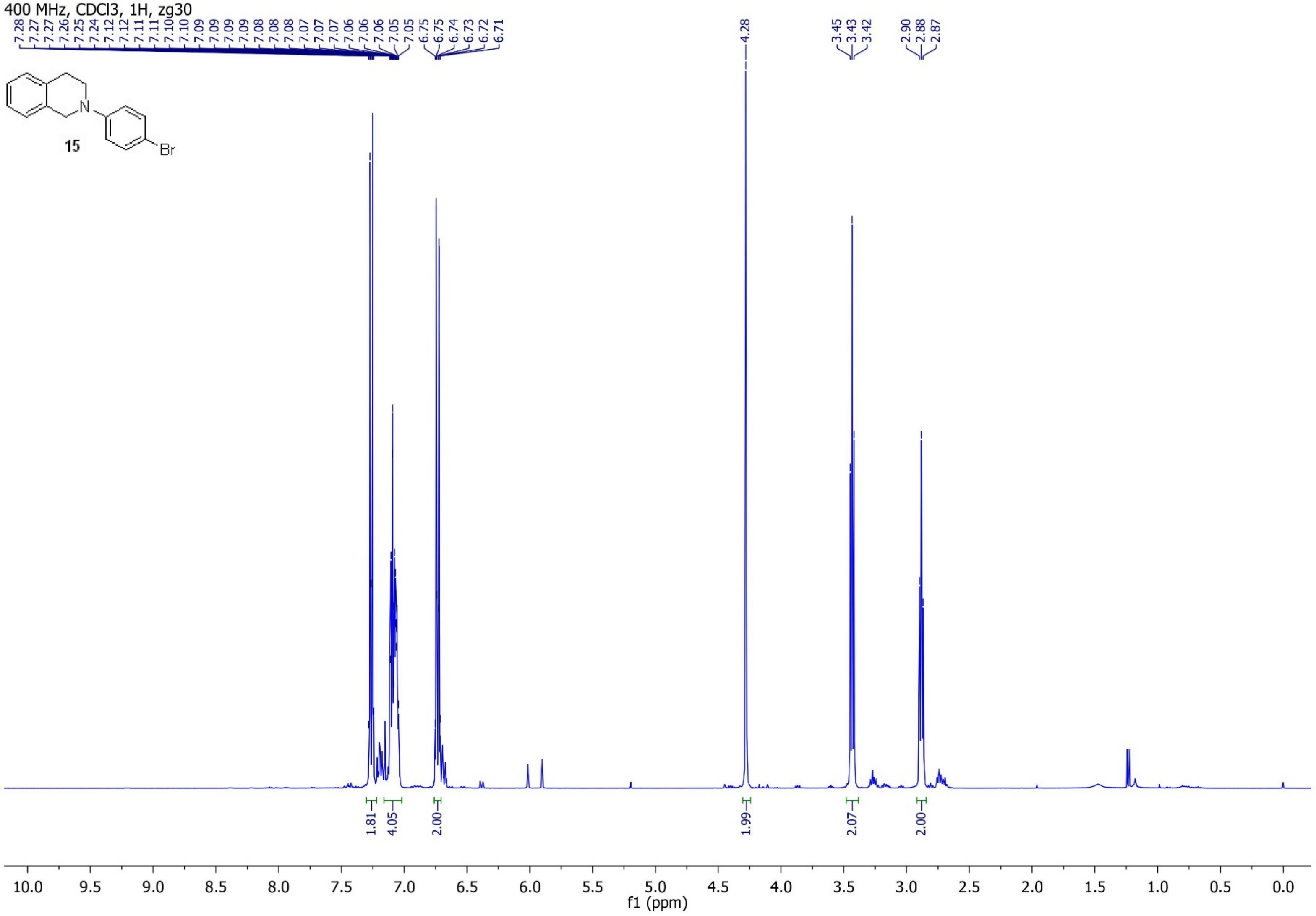

101 MHz, CDCl3, 13C, zgpg30

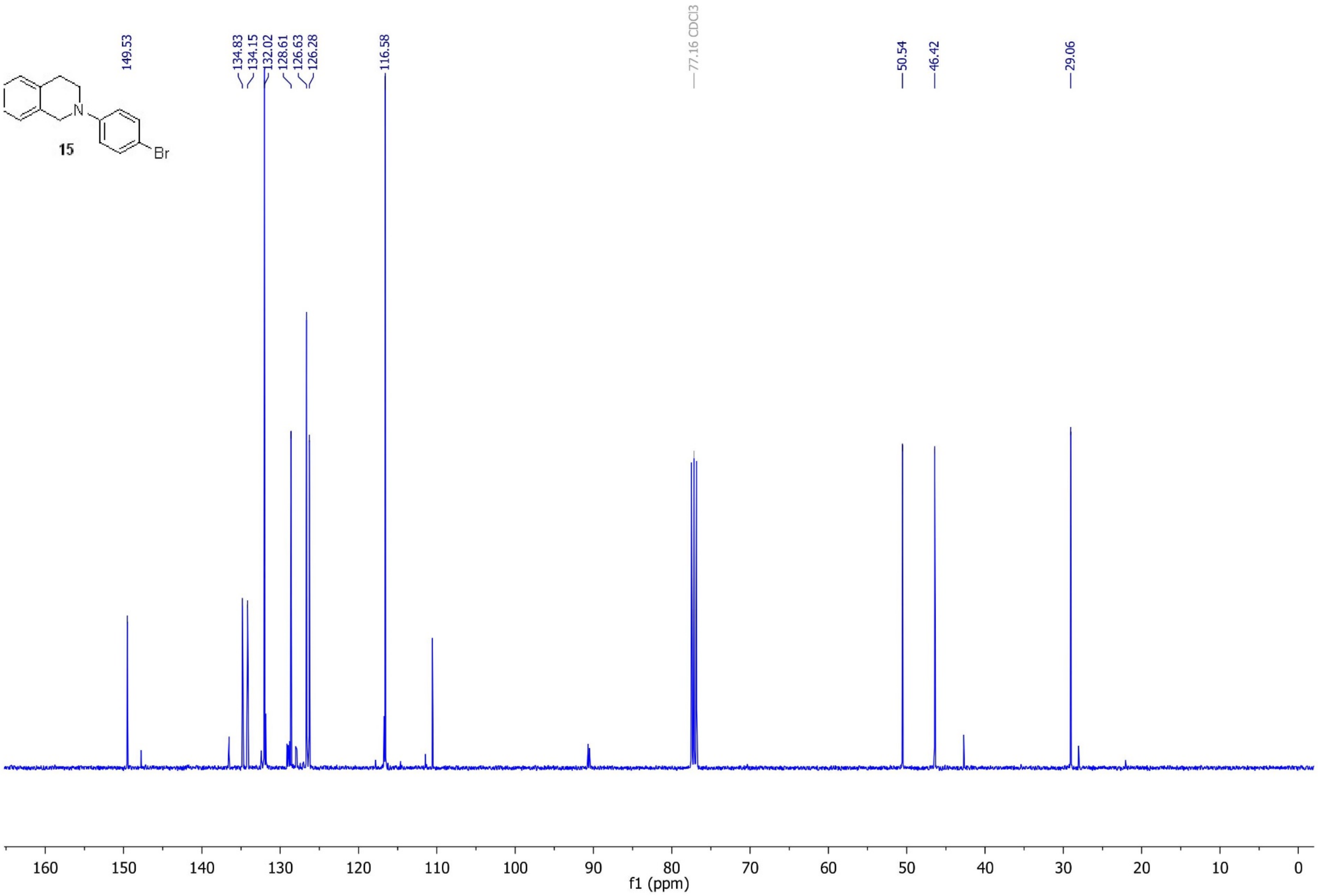

4-(3,4-Dihydroisoquinolin-2(1*H*)-yl)benzonitrile (16)

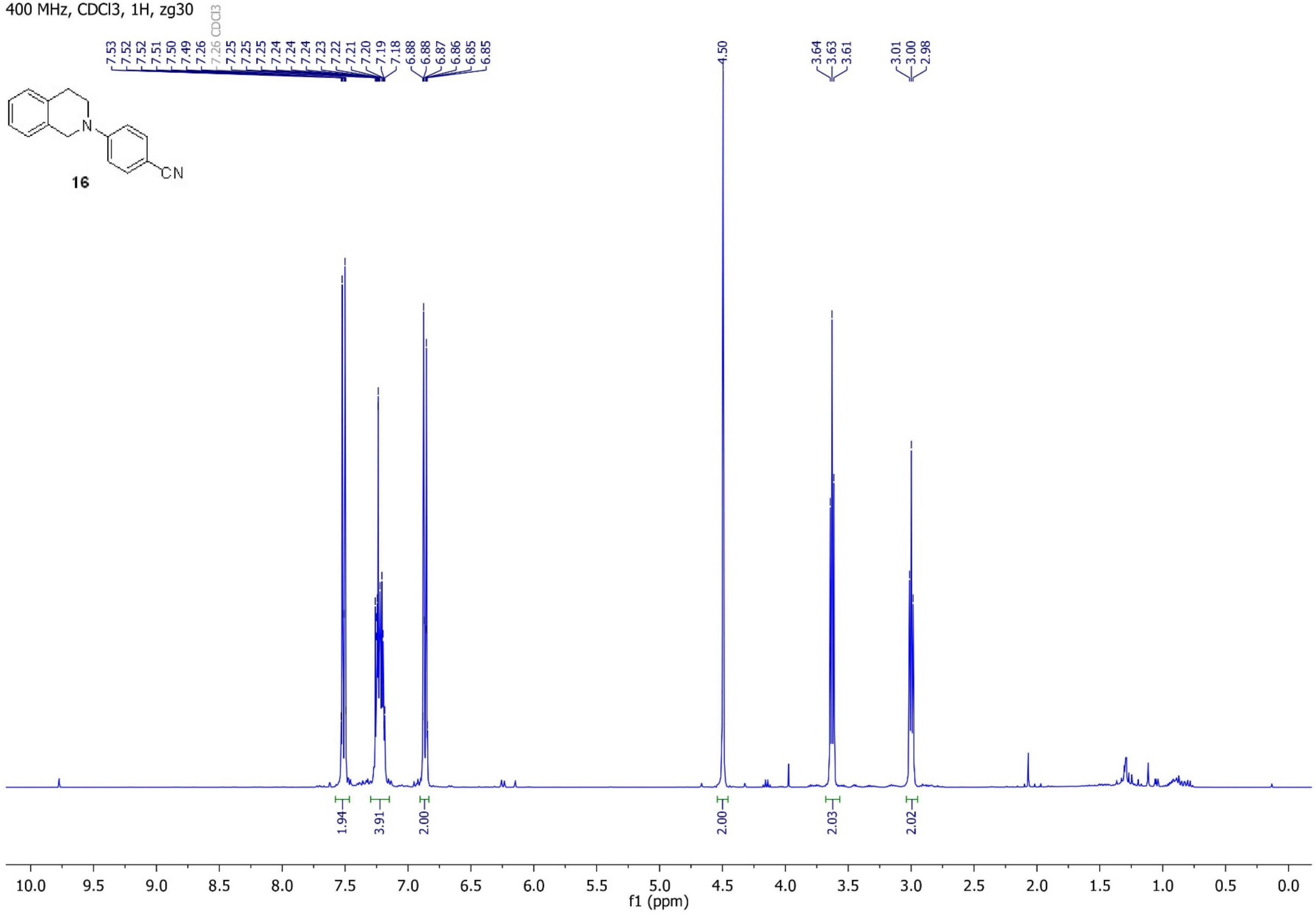

101 MHz, CDCl3, 13C, jmod

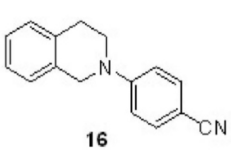

16

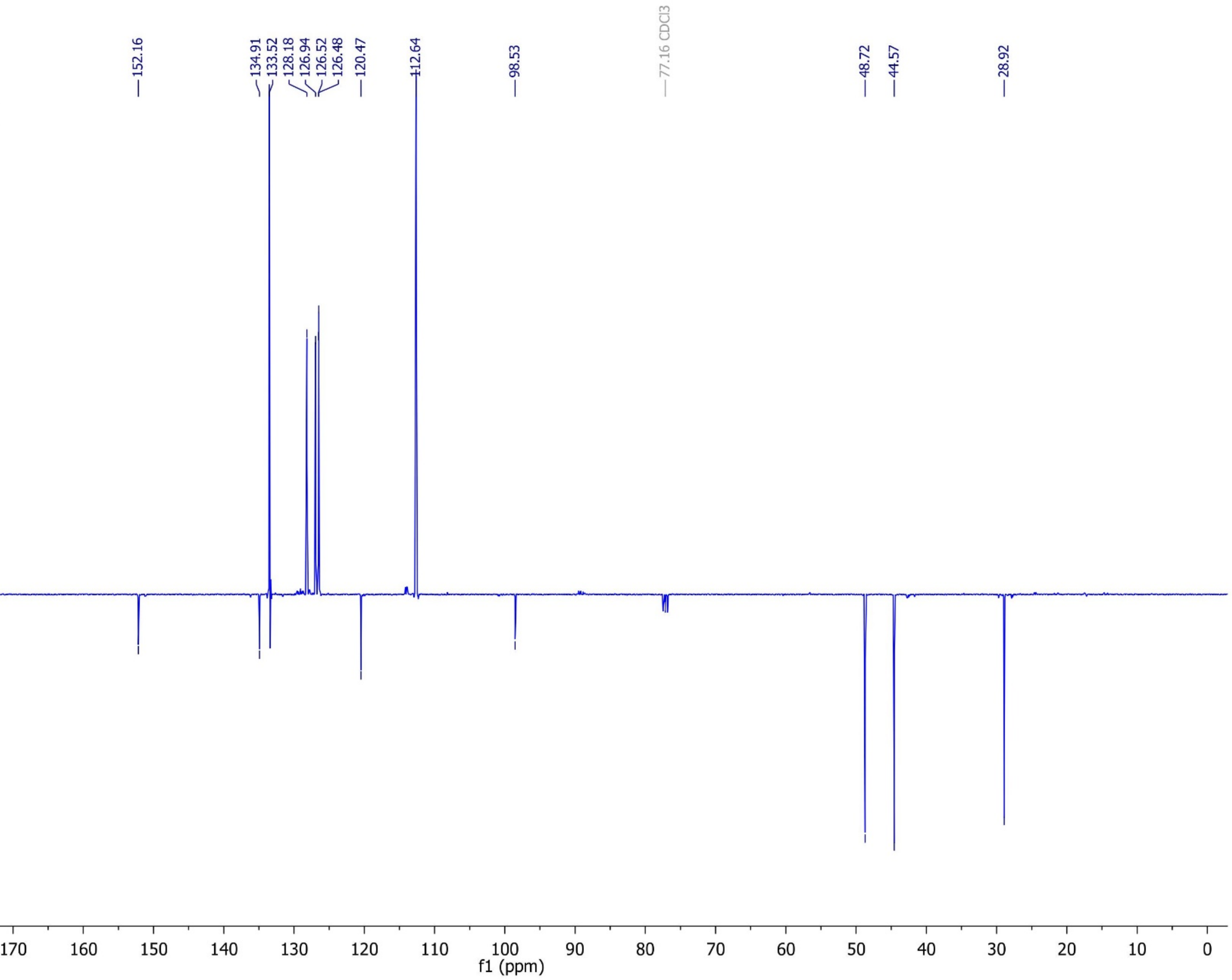

2-(4-Methoxyphenyl)-1,2,3,4-tetrahydroisoquinoline (17)

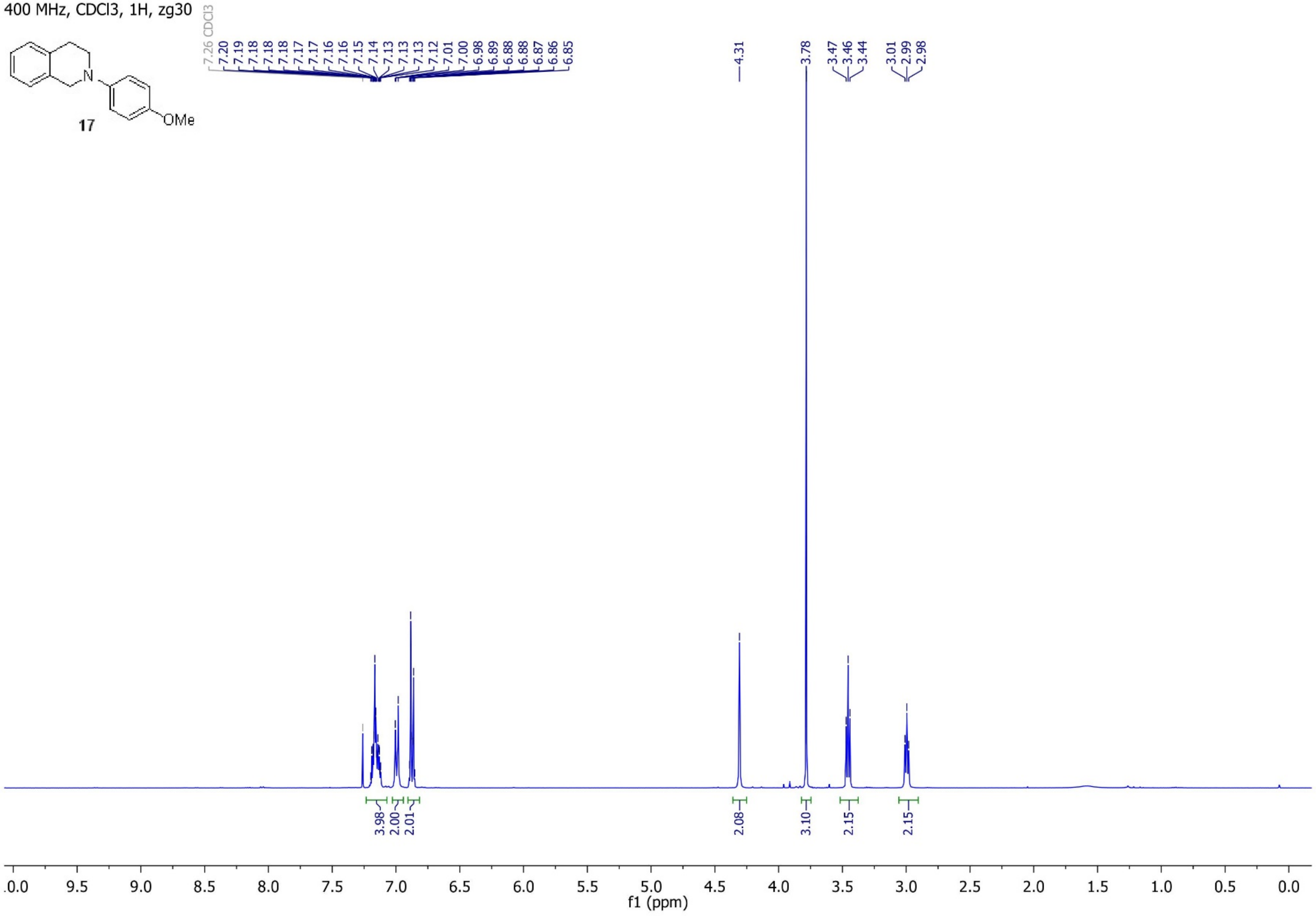

101 MHz, CDCl3, 13C, jmod

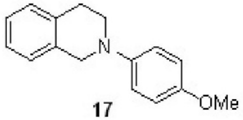

17

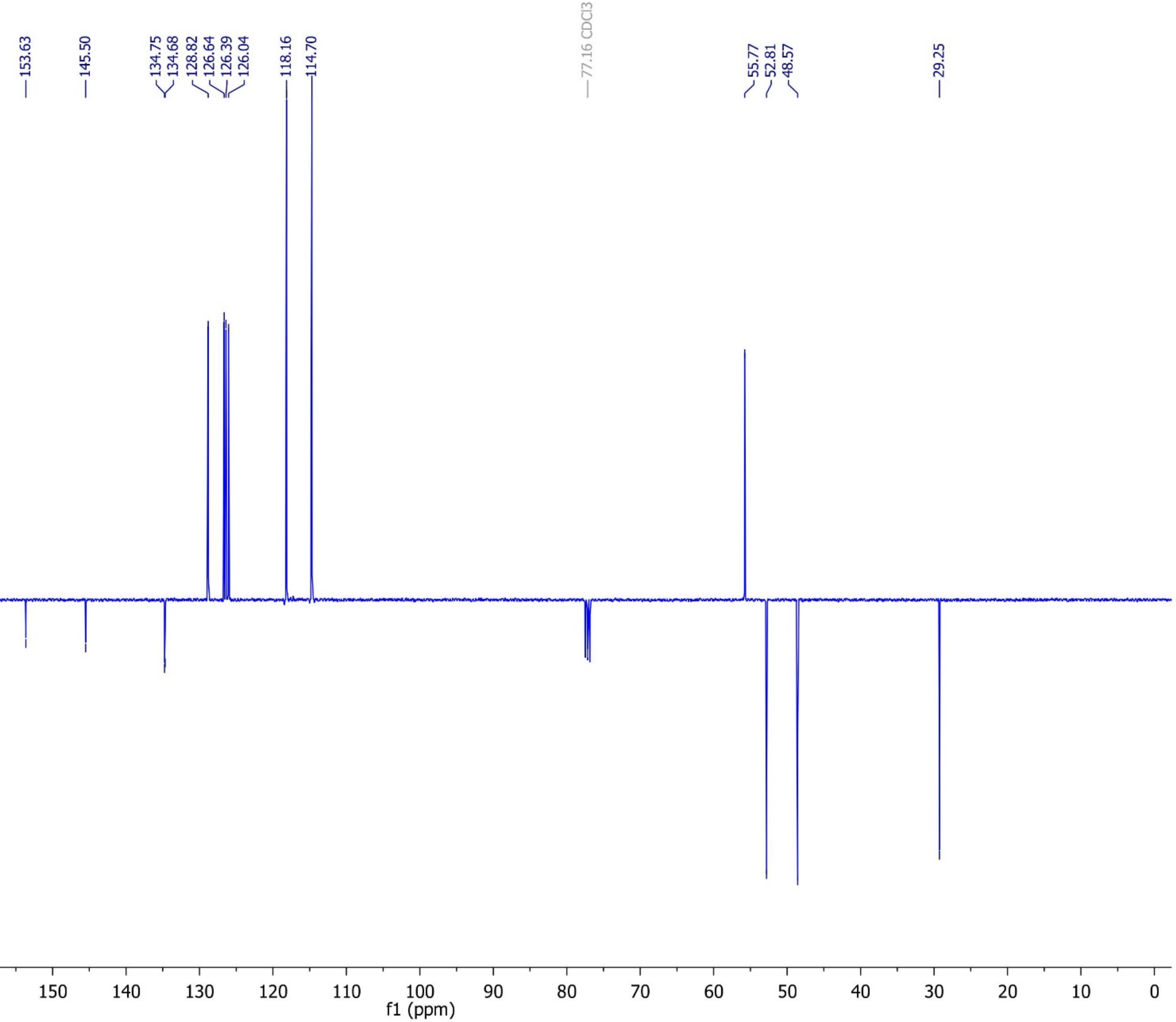

Methyl 4-(3,4-dihydroisoquinolin-2(1*H*)-yl)benzoate (18)

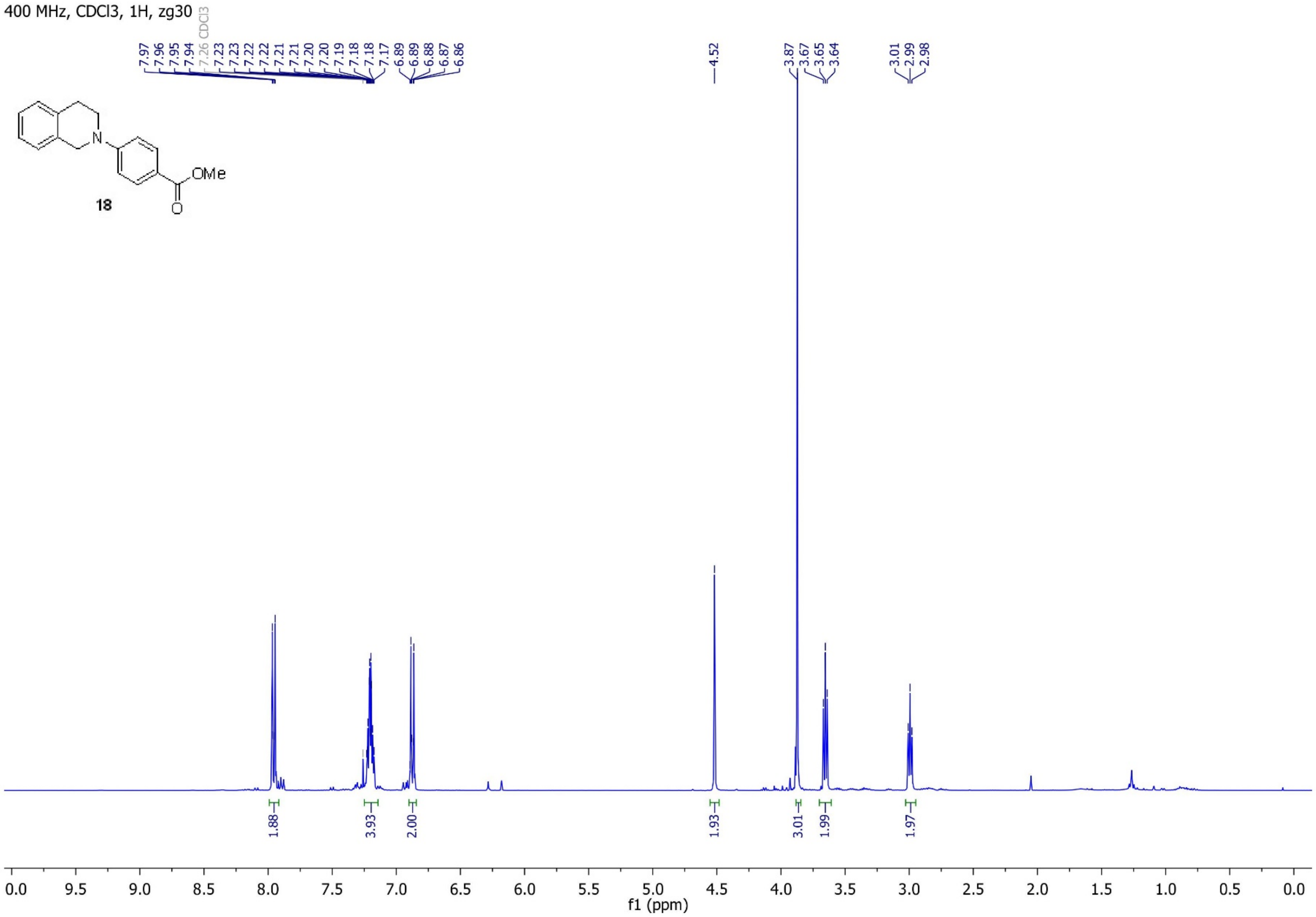

101 MHz, CDCl3, 13C, zgpg30

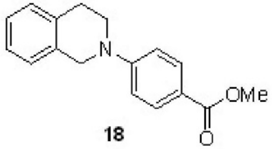

18

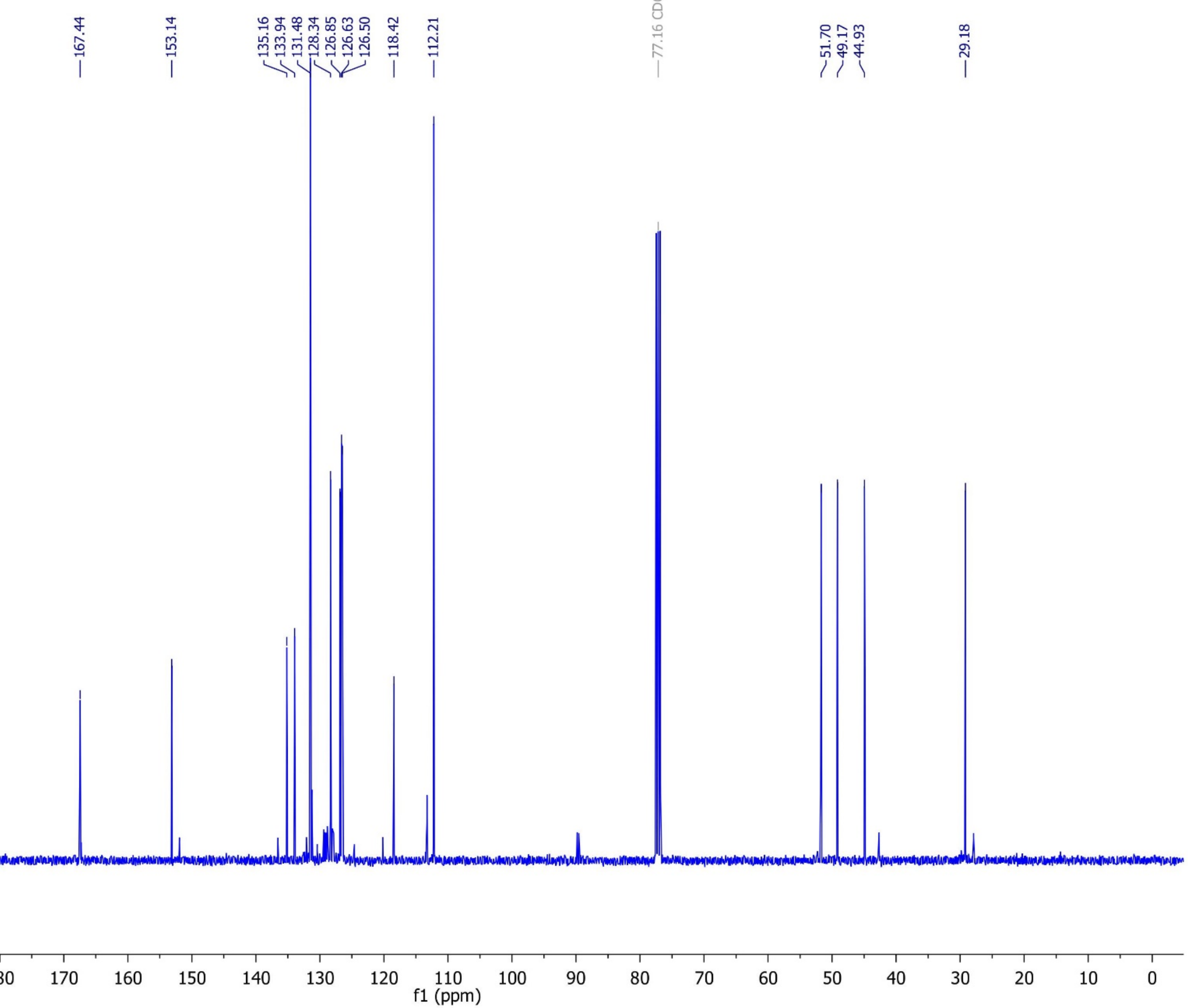

7-Fluoro-2-phenyl-1,2,3,4-tetrahydroisoquinoline (19)

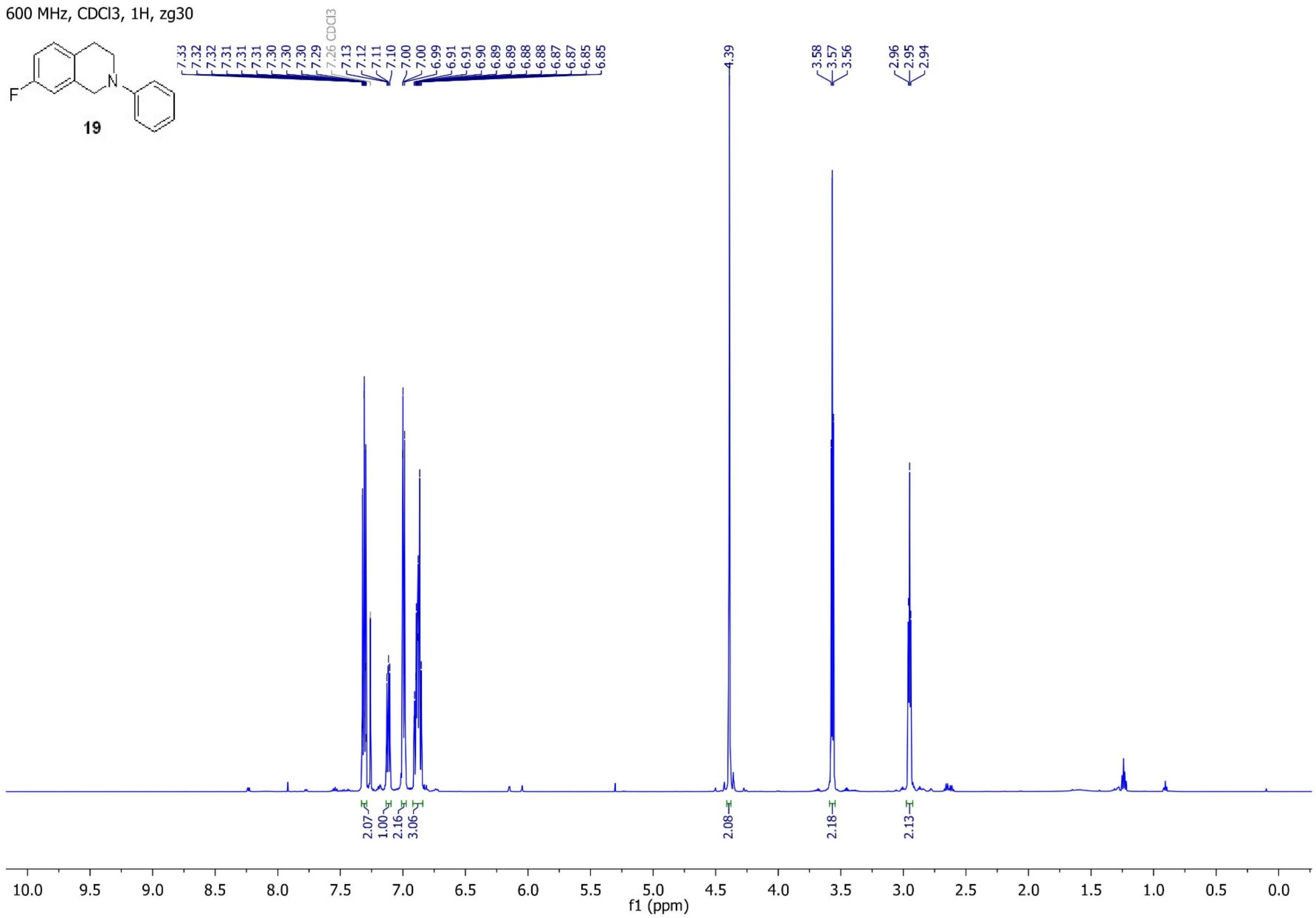

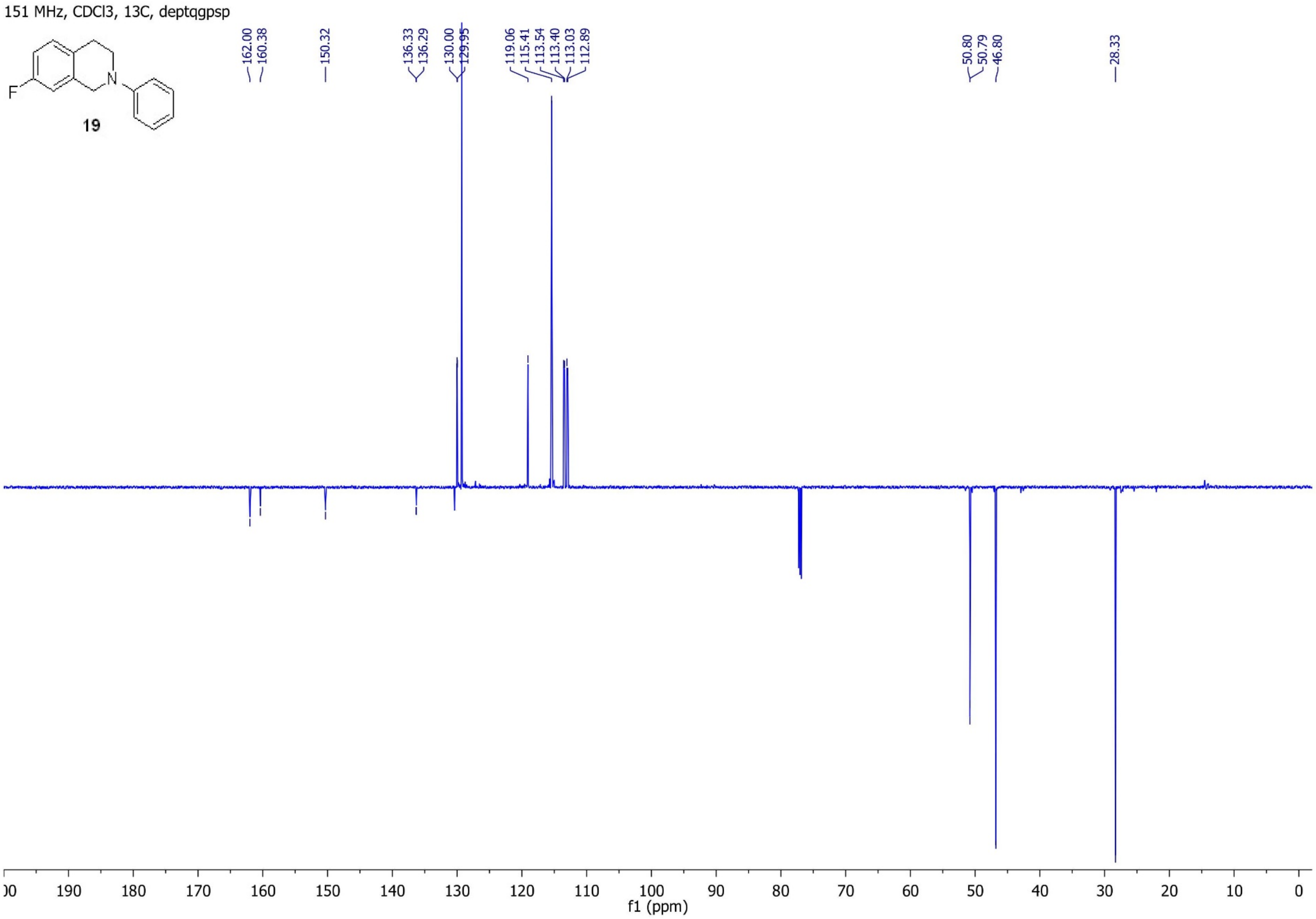
151 MHz, CDCl3, 13C, deptqgpsp
F
N
19
162.00
160.38
150.32
136.33
136.29
130.00
129.95
119.06
115.41
113.54
113.40
113.03
112.89
50.80
50.79
46.80
28.33
f1 (ppm)

565 MHz, CDCl3, 19F, zgflqn

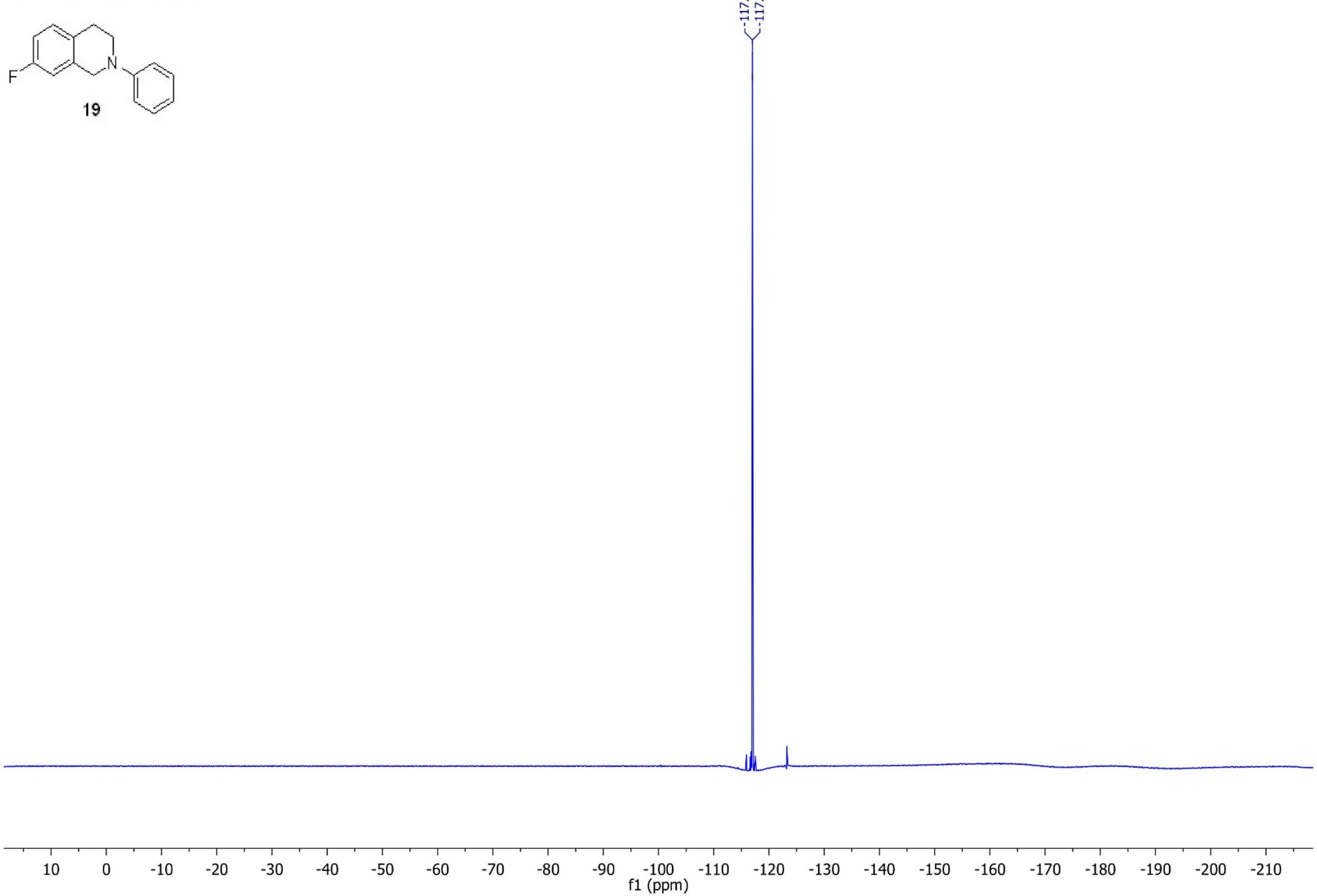

6,7-Dimethoxy-2-phenyl-1,2,3,4-tetrahydroisoquinoline (20)

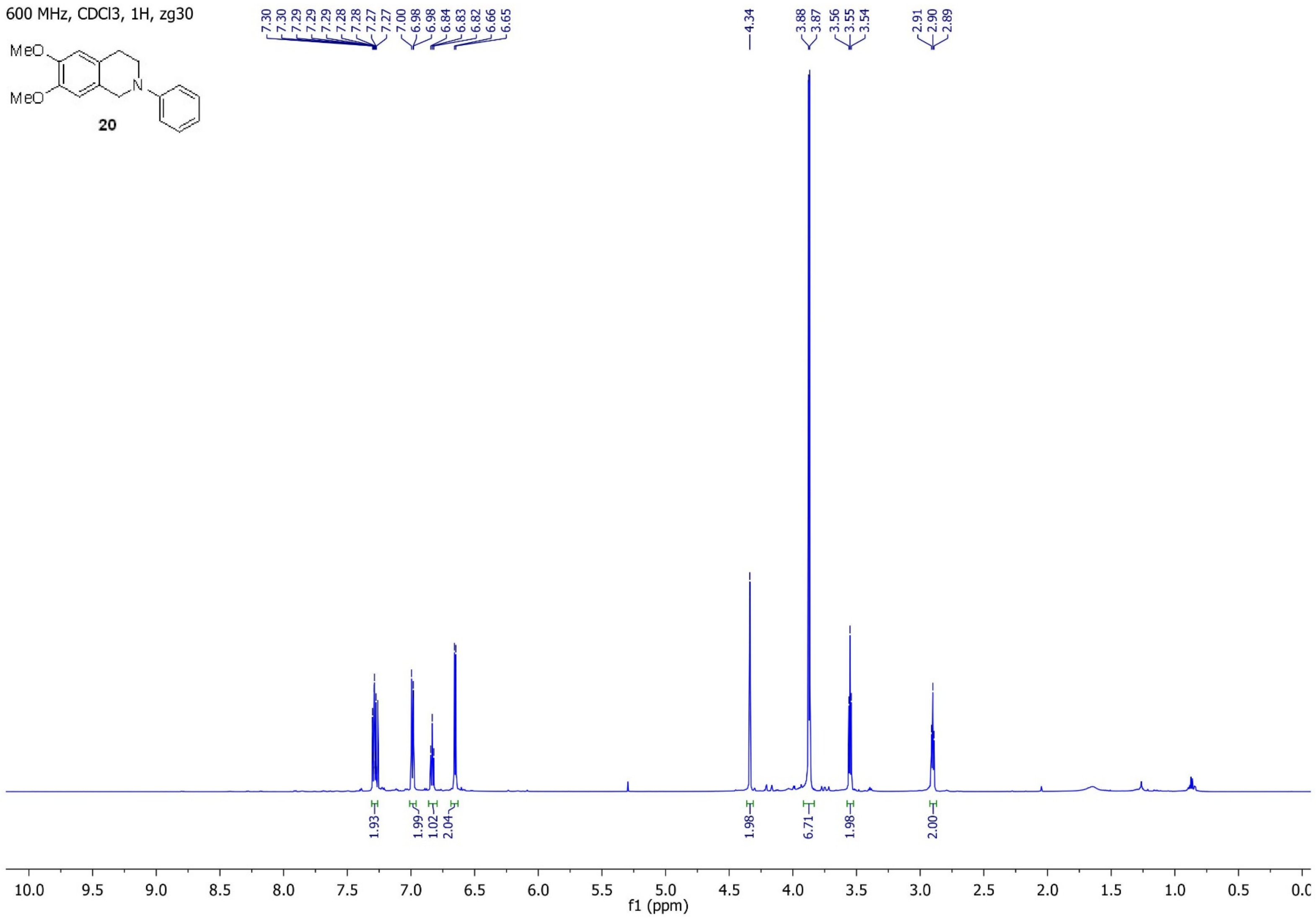

151 MHz, CDCl3, 13C, jmod

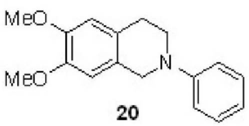

20

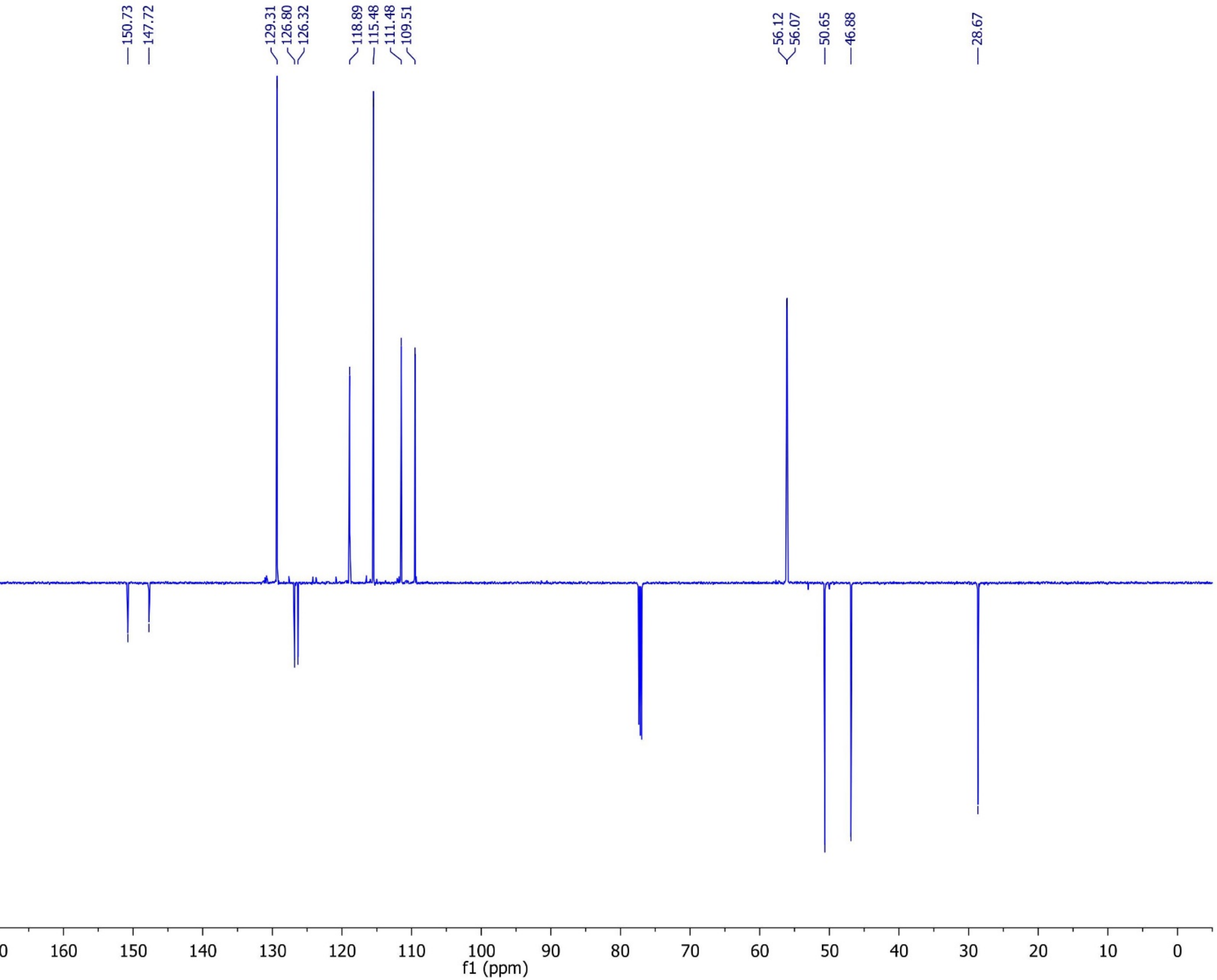

Ethyl (4-fluorophenethyl)carbamate (S1)

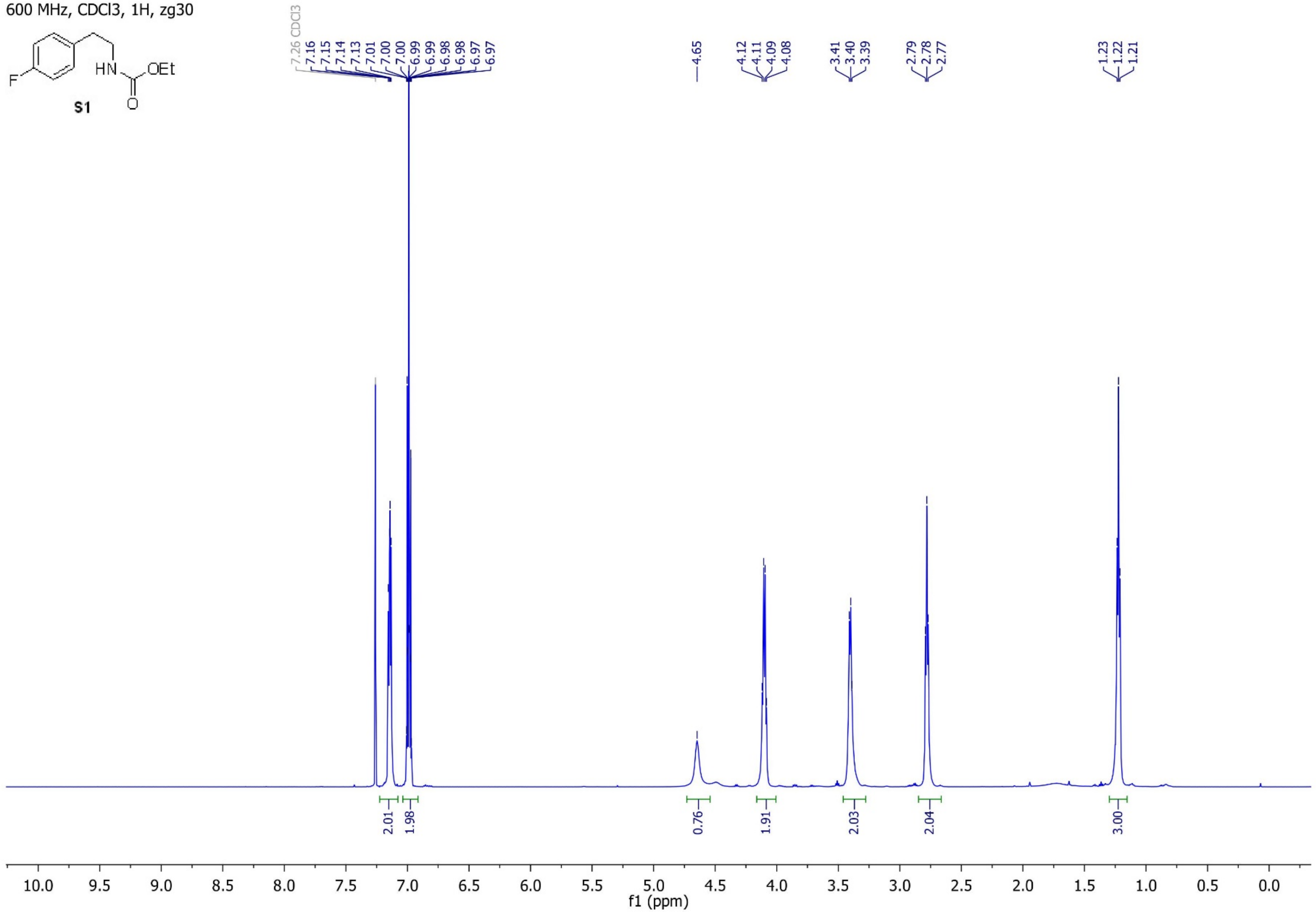

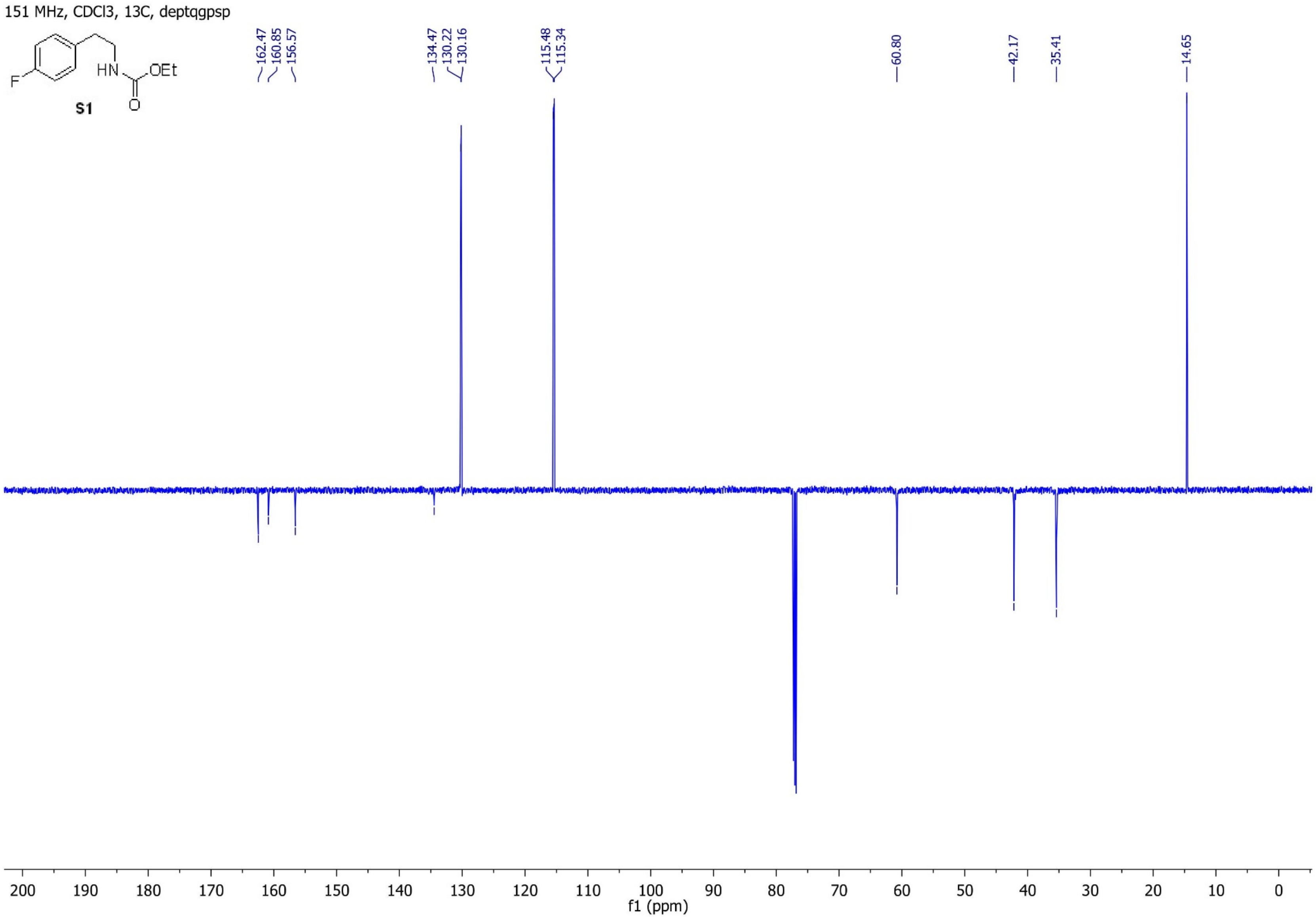

151 MHz, CDCl3, 13C, deptqgpsp
F
HN
OEt
O
S1
162.47
160.85
156.57
134.47
130.22
130.16
115.48
115.34
60.80
42.17
35.41
14.65
200 190 180 170 160 150 140 130 120 110 100 90 80 70 60 50 40 30 20 10 0
f1 (ppm)

565 MHz, CDCl3, 19F, zgflqn

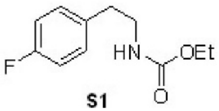

S1

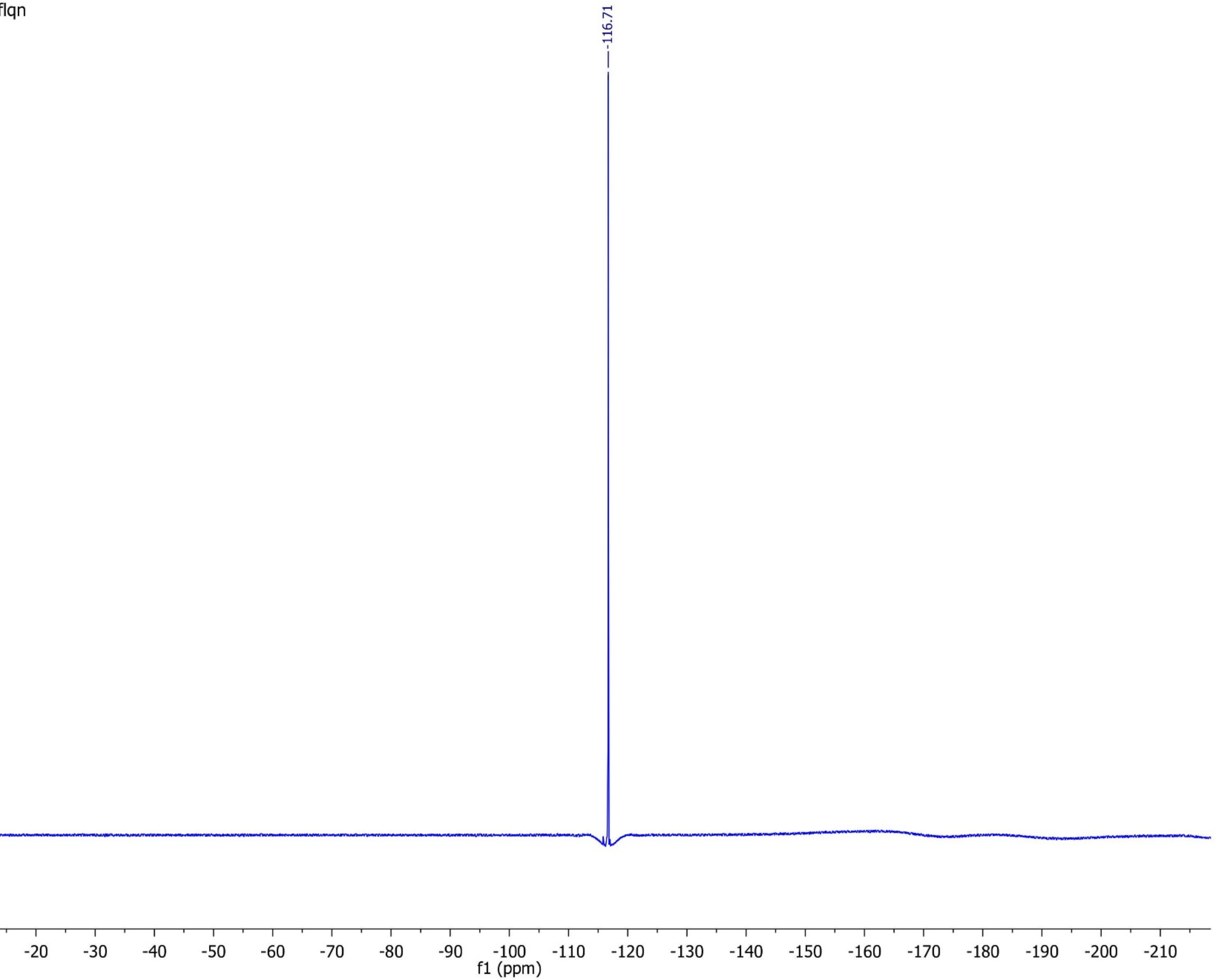

7-Fluoro-3,4-dihydroisoquinolin-1(2*H*)-one (S2)

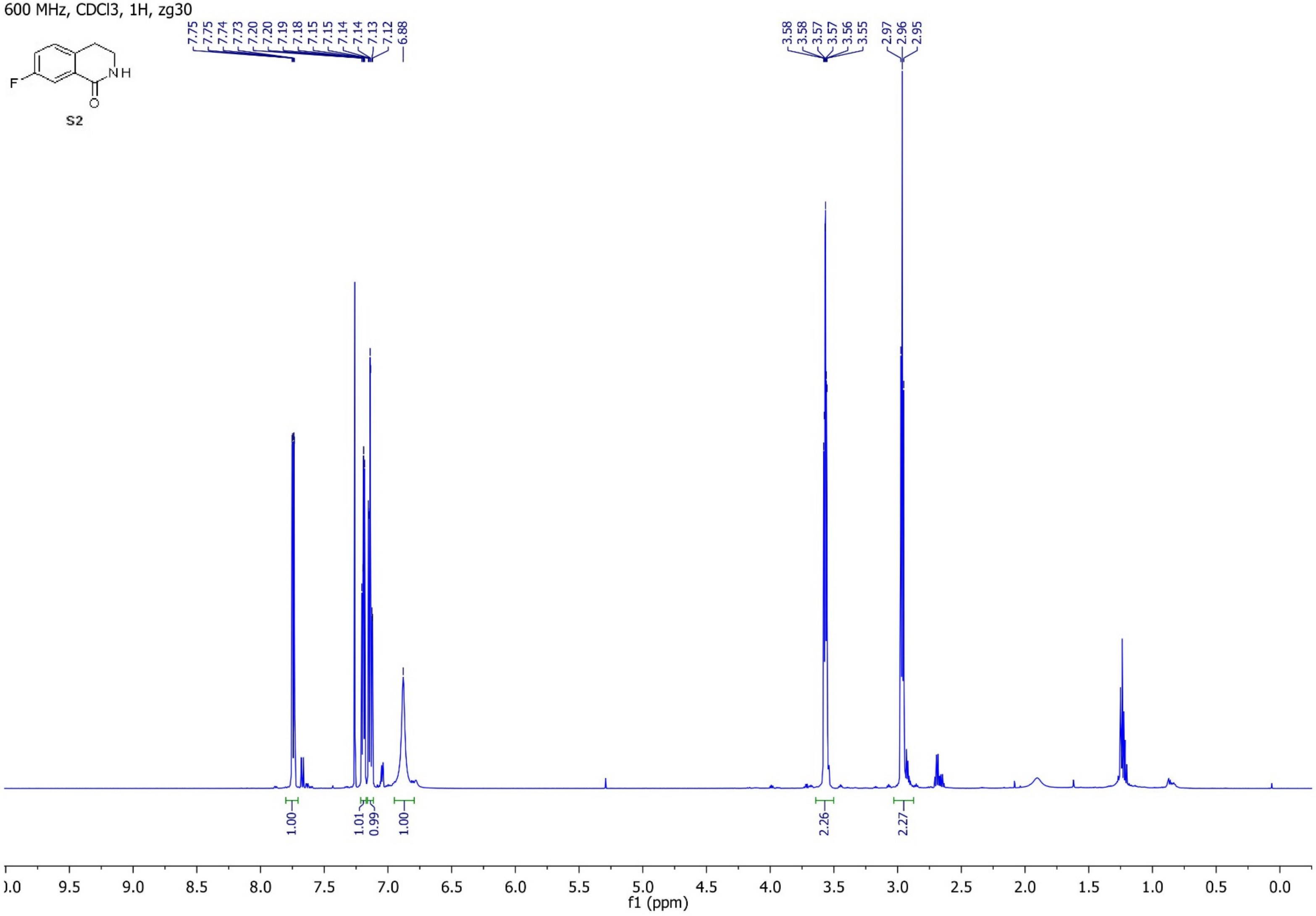

151 MHz, CDCl3, 13C, deptqgpsp

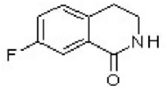

S2

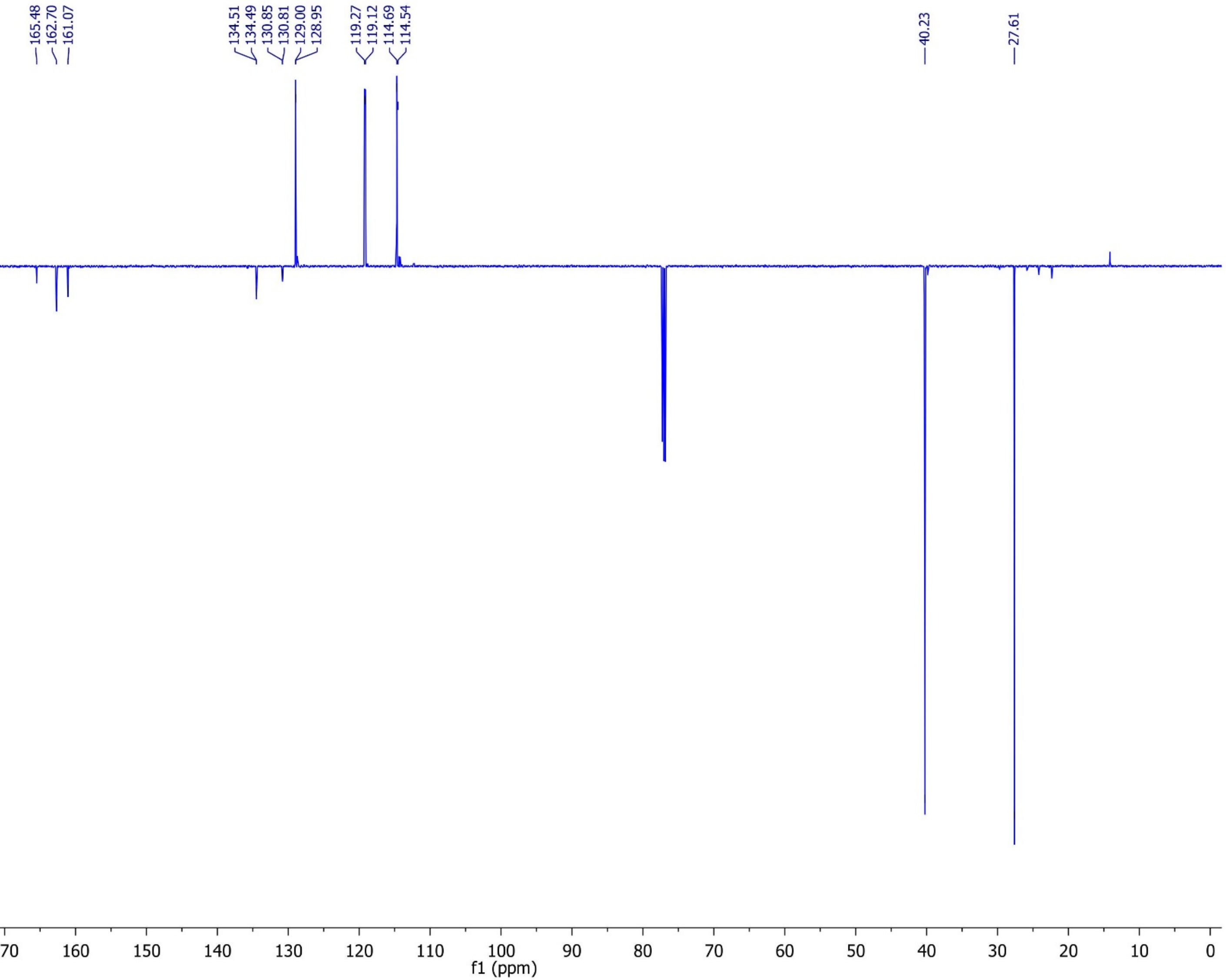

565 MHz, CDCl3, 19F, zgflqn

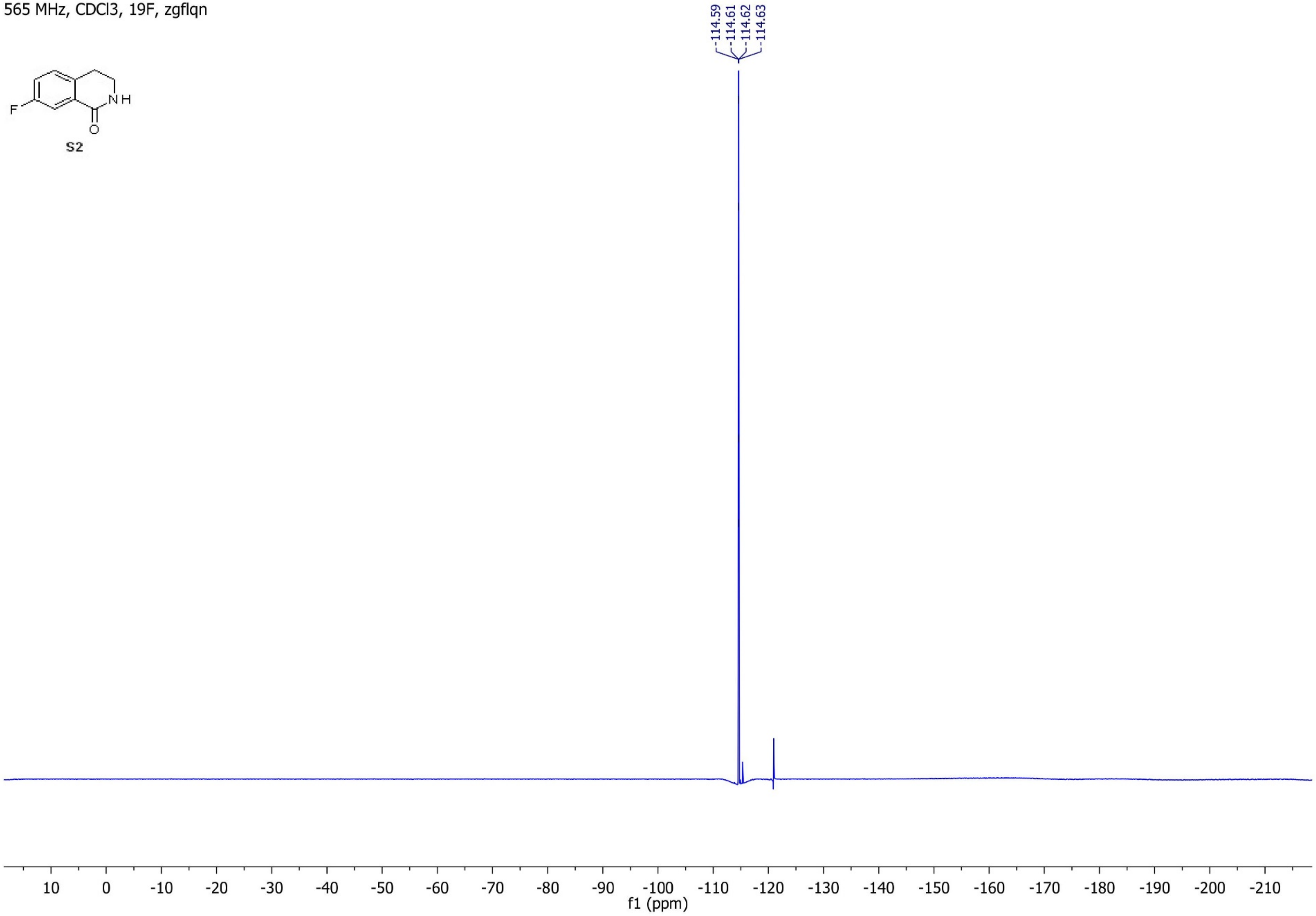

7-Fluoro-1,2,3,4-tetrahydroisoquinoline (S3)

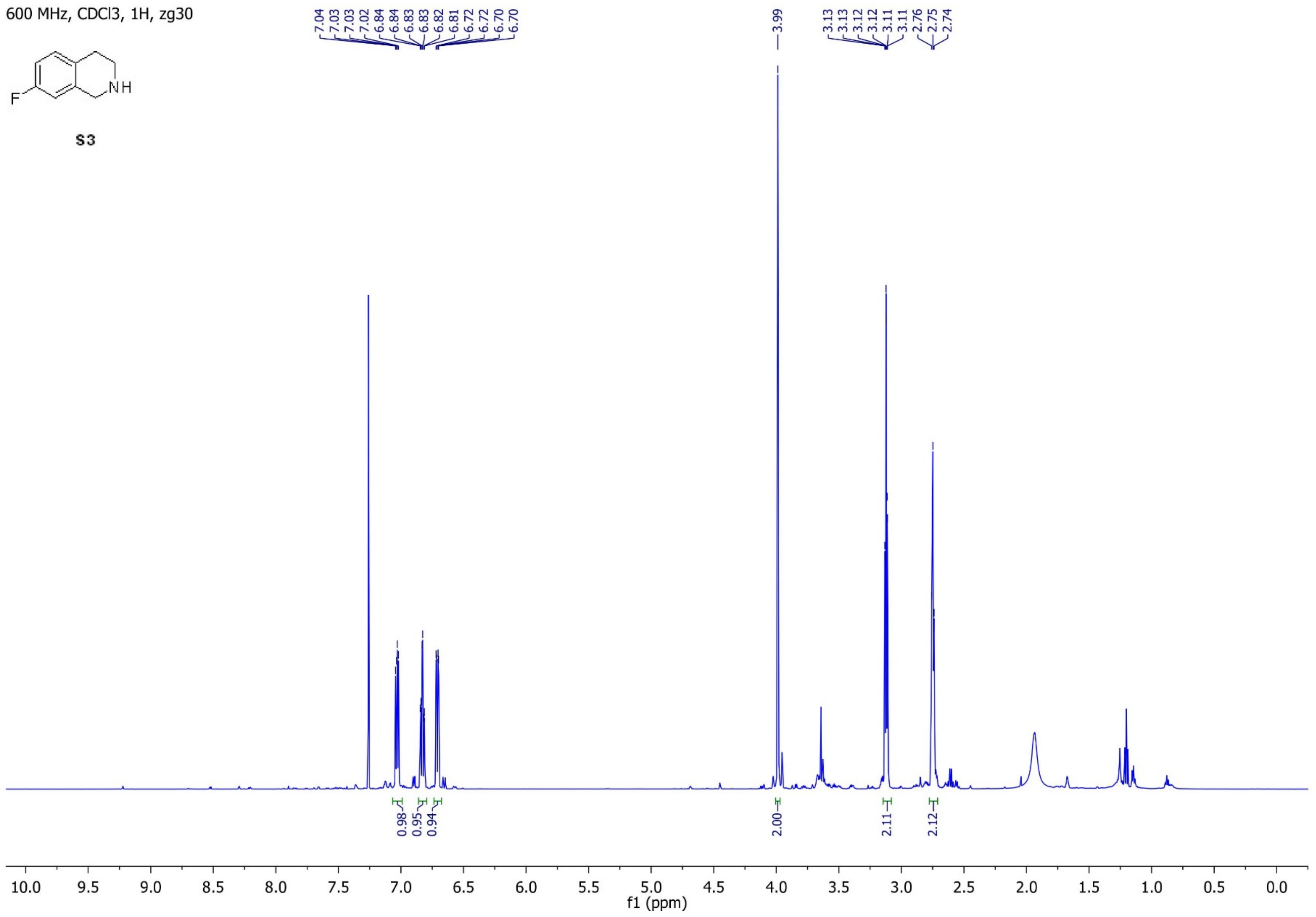

151 MHz, CDCl3, 13C, deptqgpsp

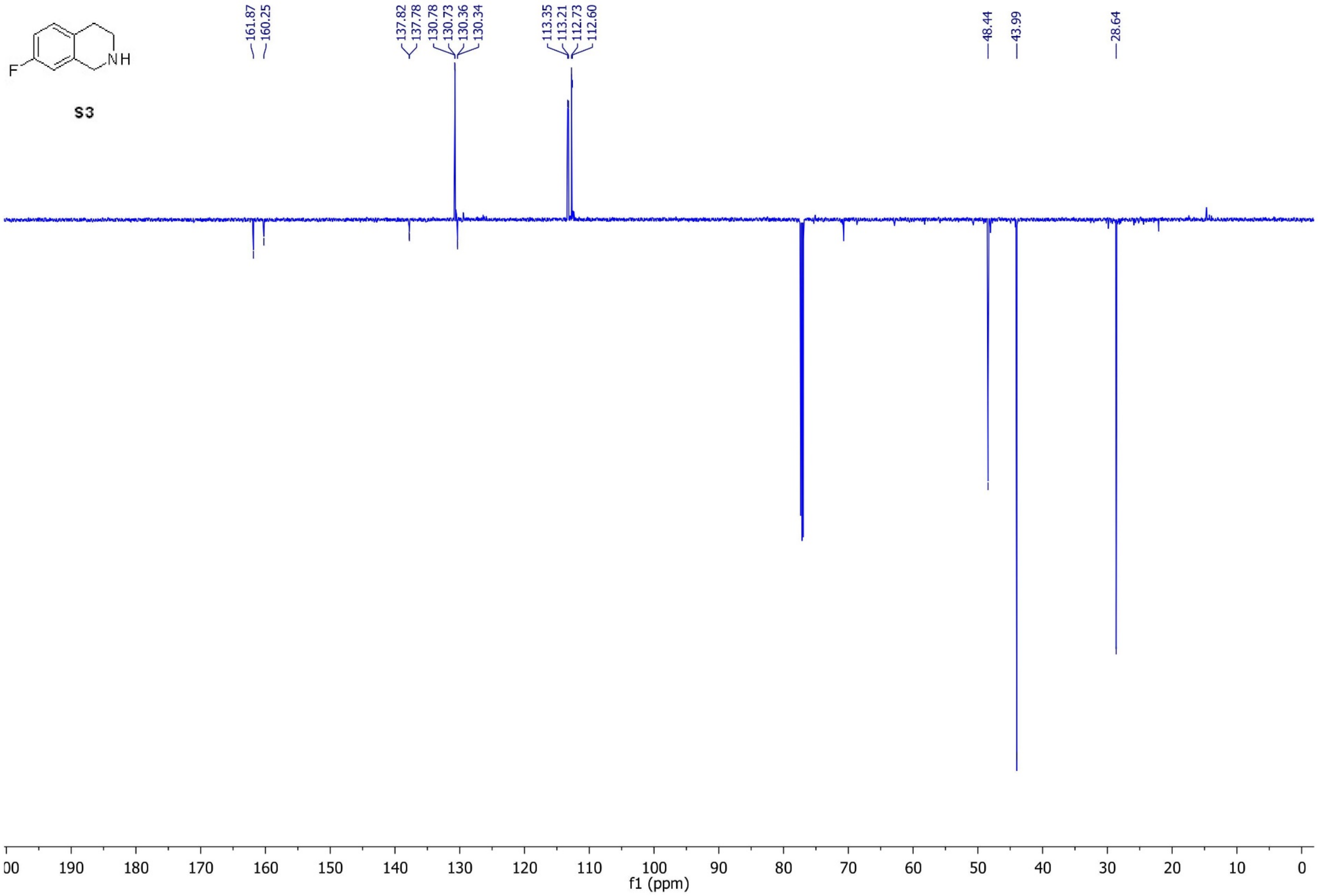

565 MHz, CDCl3, 19F, zgflqn

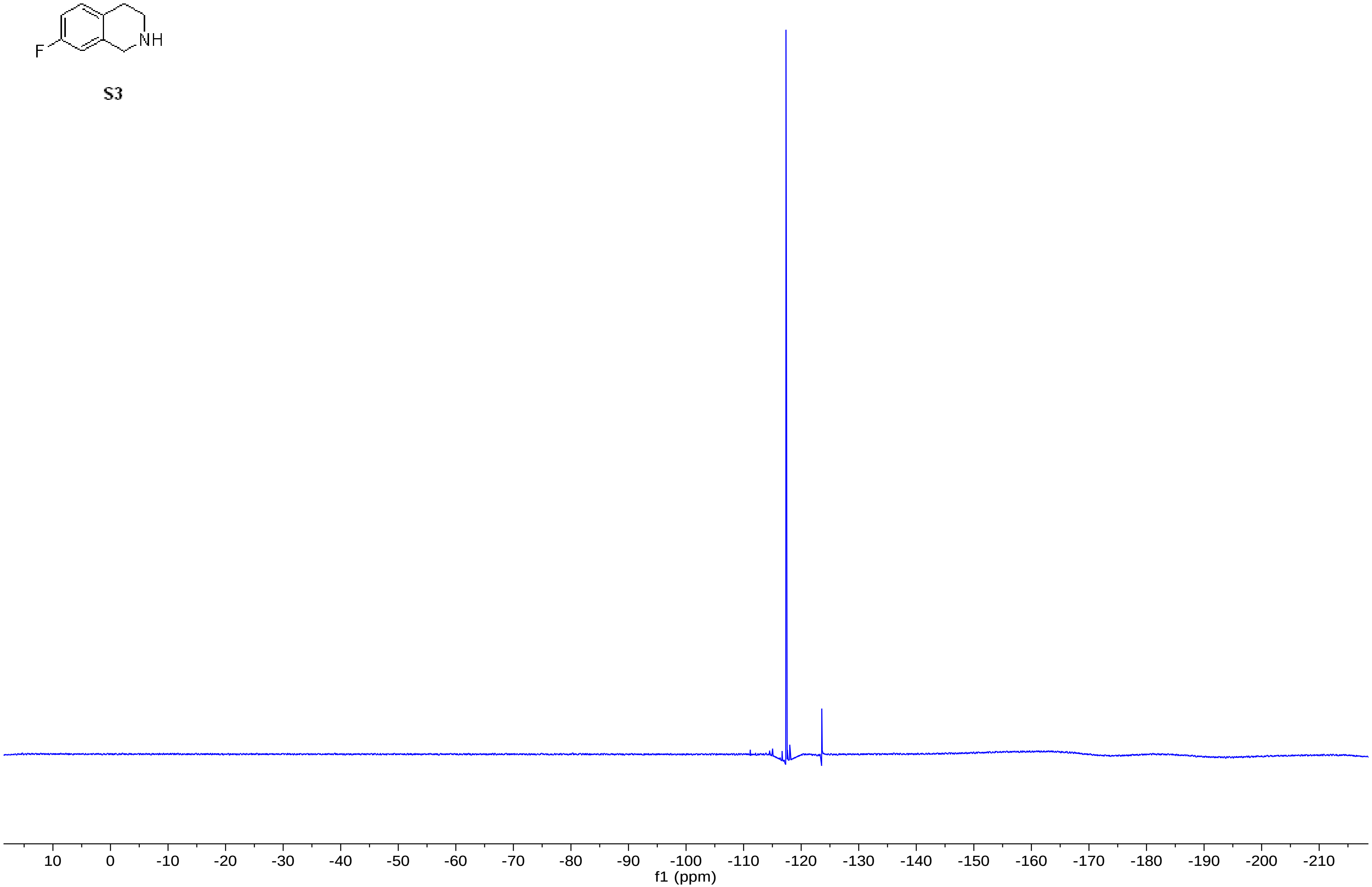

S3

6,7-Dimethoxy-1,2,3,4-tetrahydroisoquinoline (S4)

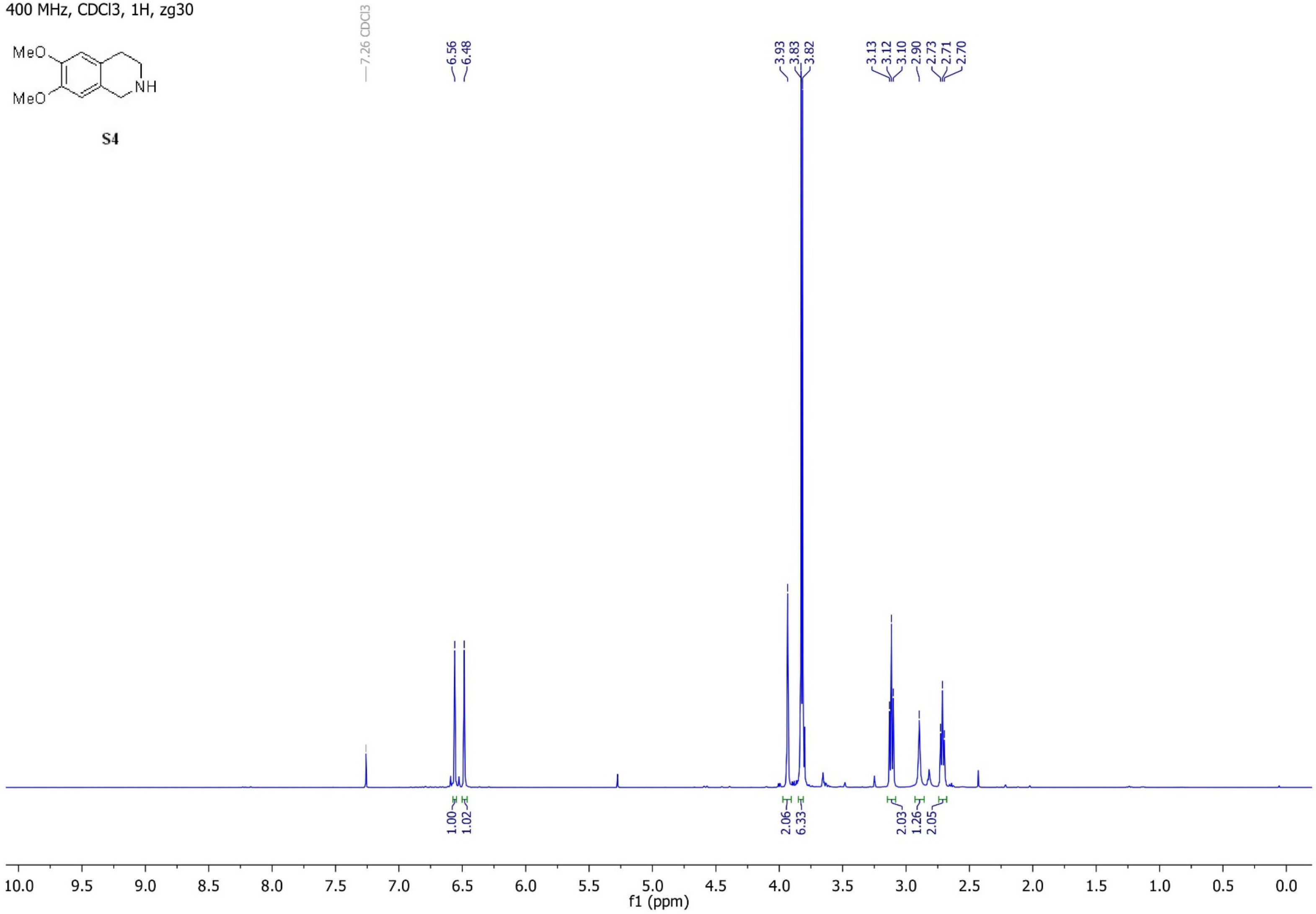

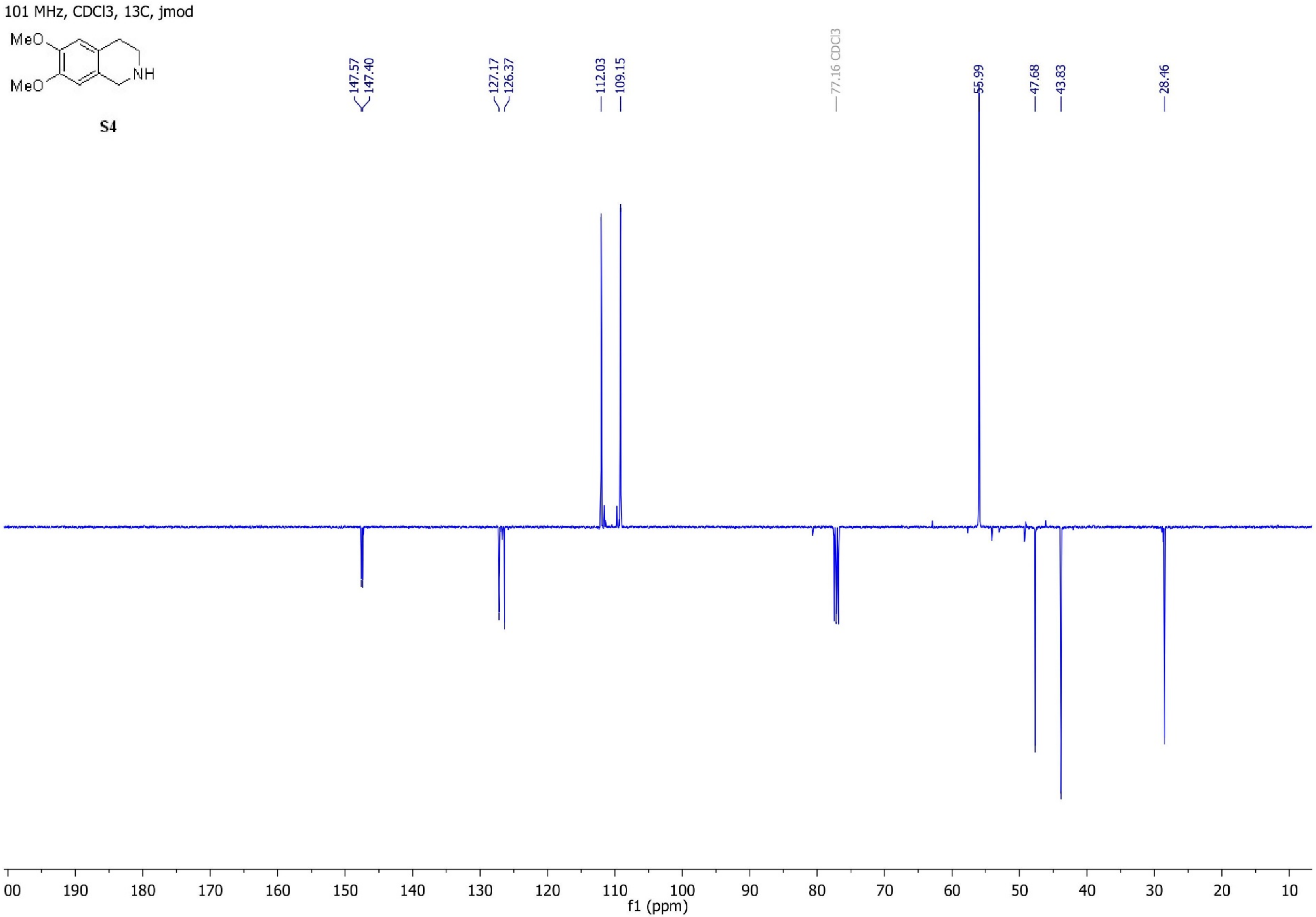

101 MHz, CDCl3, 13C, jmod
MeO
MeO
NH
S4
147.57
147.40
127.17
126.37
112.03
109.15
77.16 CDCl3
55.99
47.68
43.83
28.46
00
190
180
170
160
150
140
130
120
110
100
90
80
70
60
50
40
30
20
10
f1 (ppm)

1-Benzyl-2,4,6-triphenylpyridinium tetrafluoroborate (2)

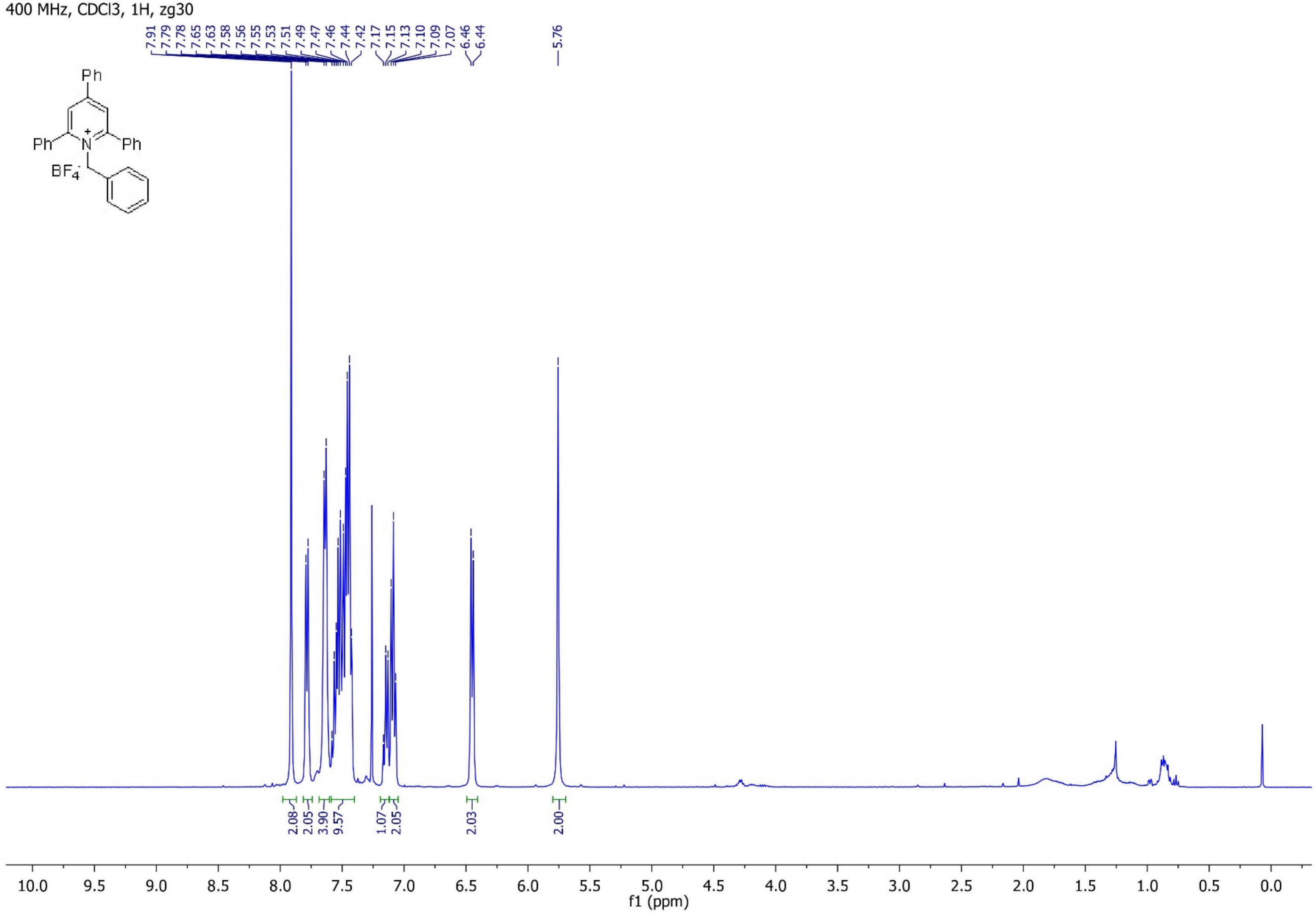

101 MHz, CDCl3, 13C, zgpg30

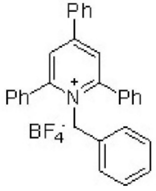

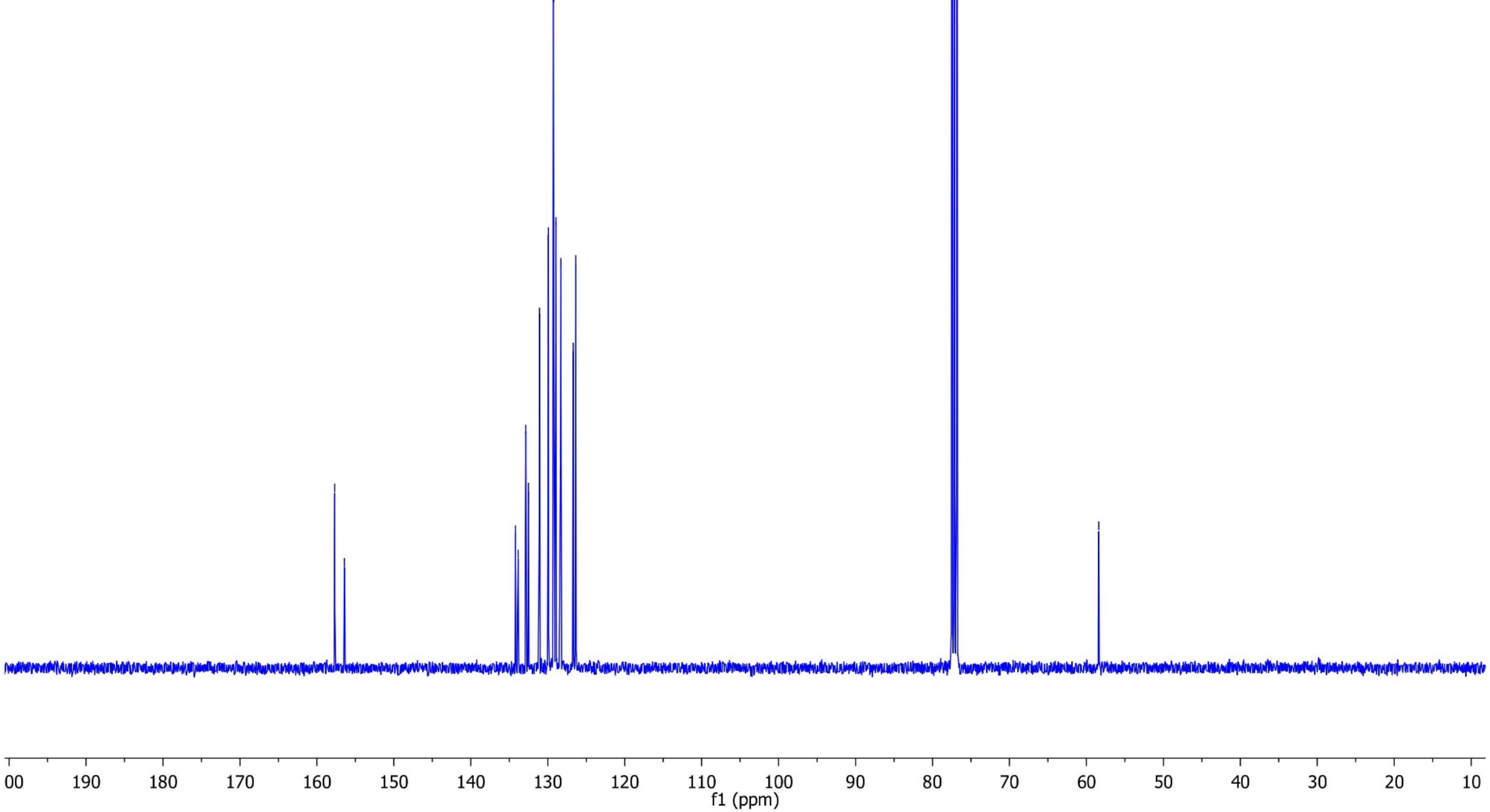

376 MHz. CDCl3. 19F, zgflqn

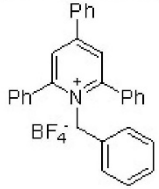

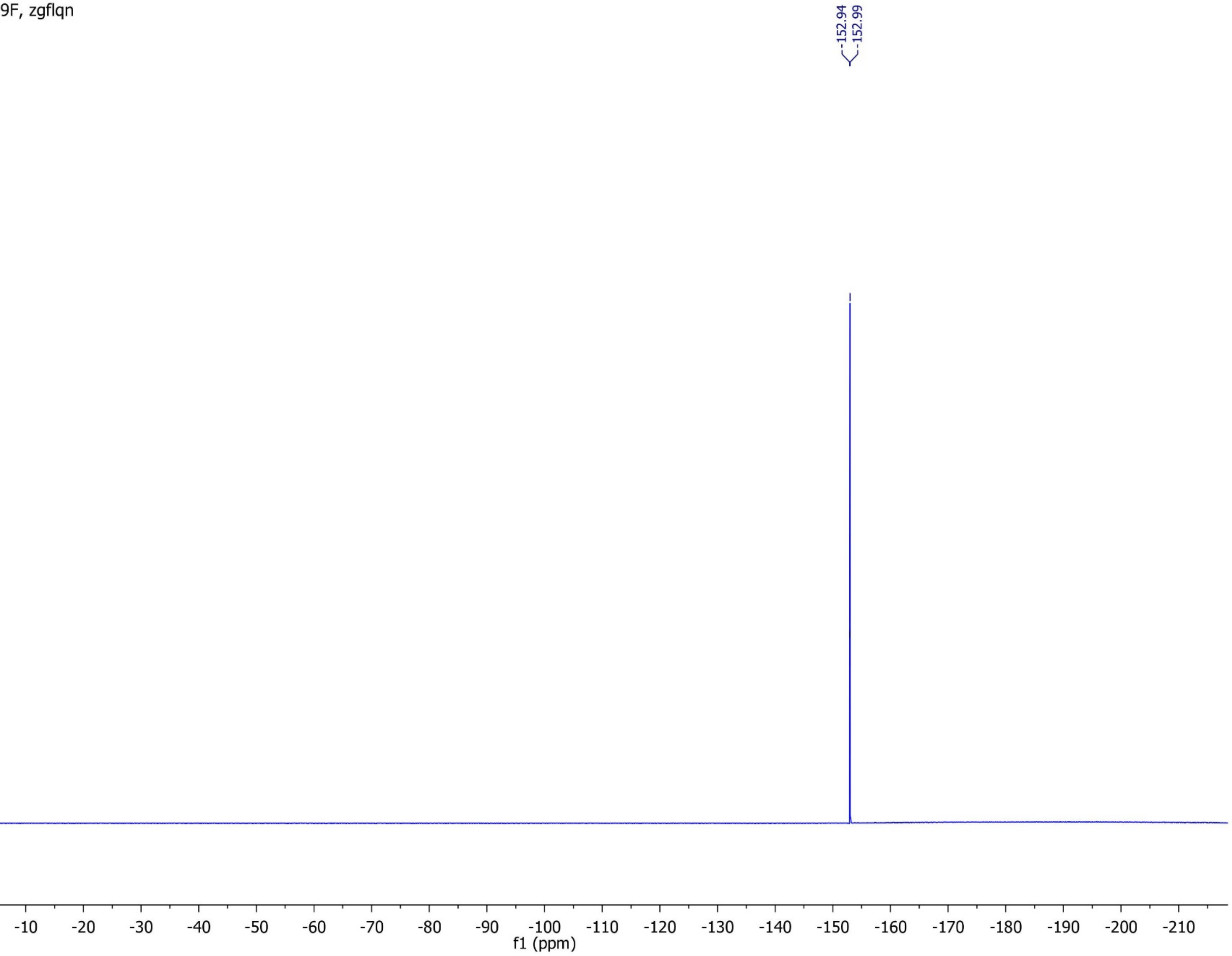

2,4,6-Triphenyl-1-(4-fluorobenzyl)pyridin-1-ium tetrafluoroborate (S5)

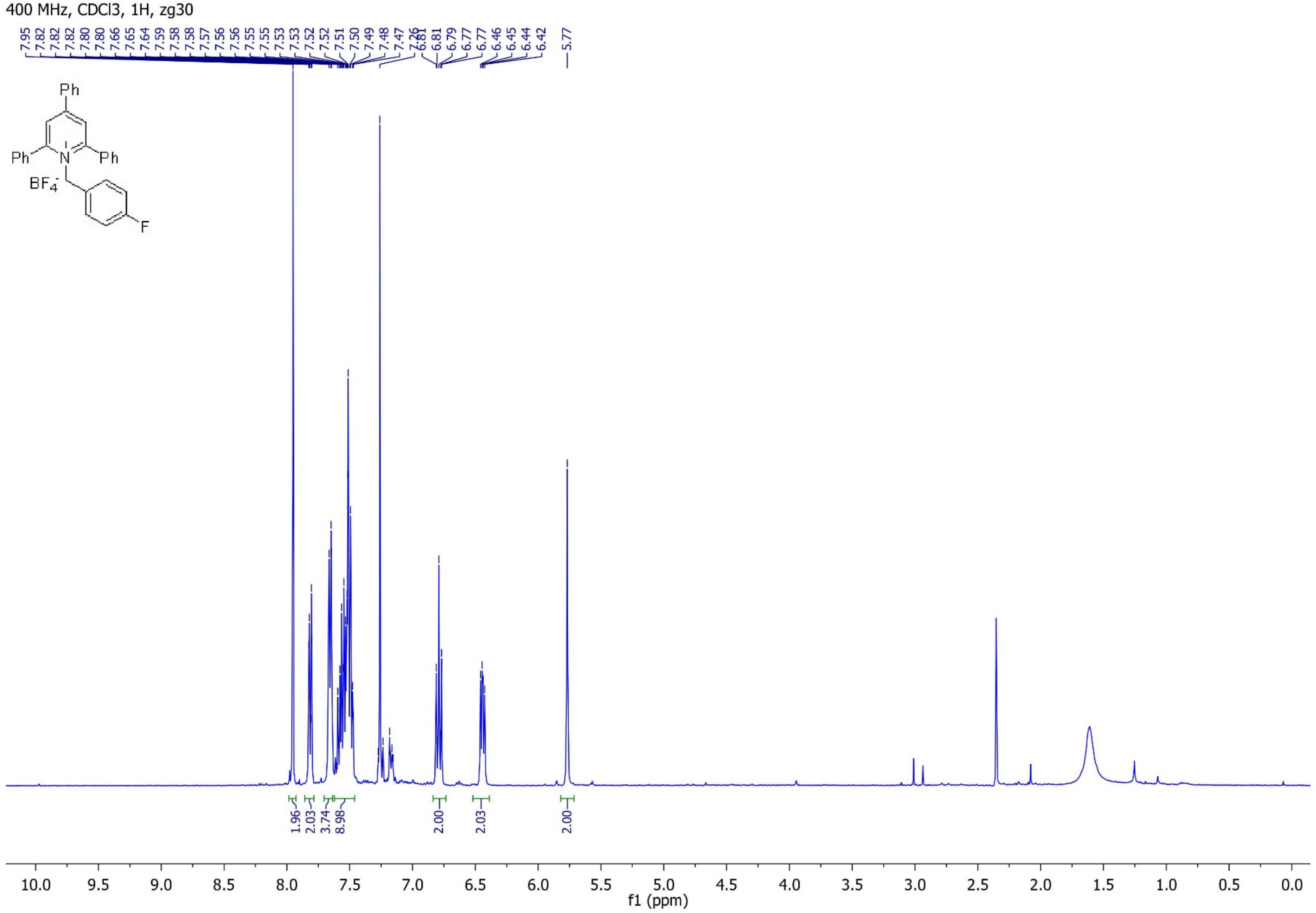

151 MHz, CDCl3, 13C, deptqgpsp

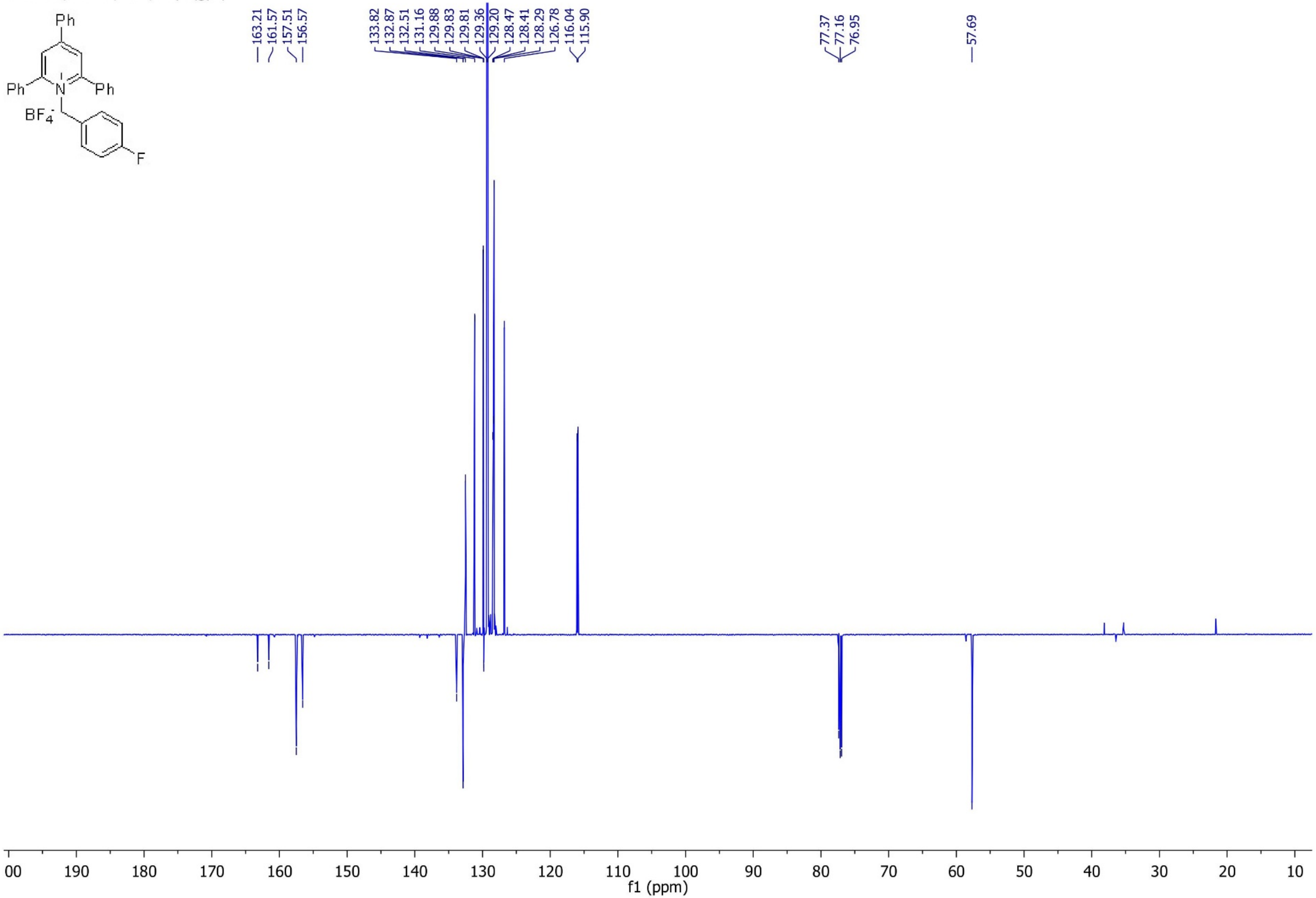

565 MHz, CDCl3, 19F, zgflqn

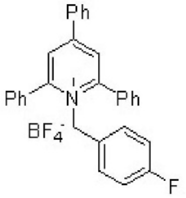

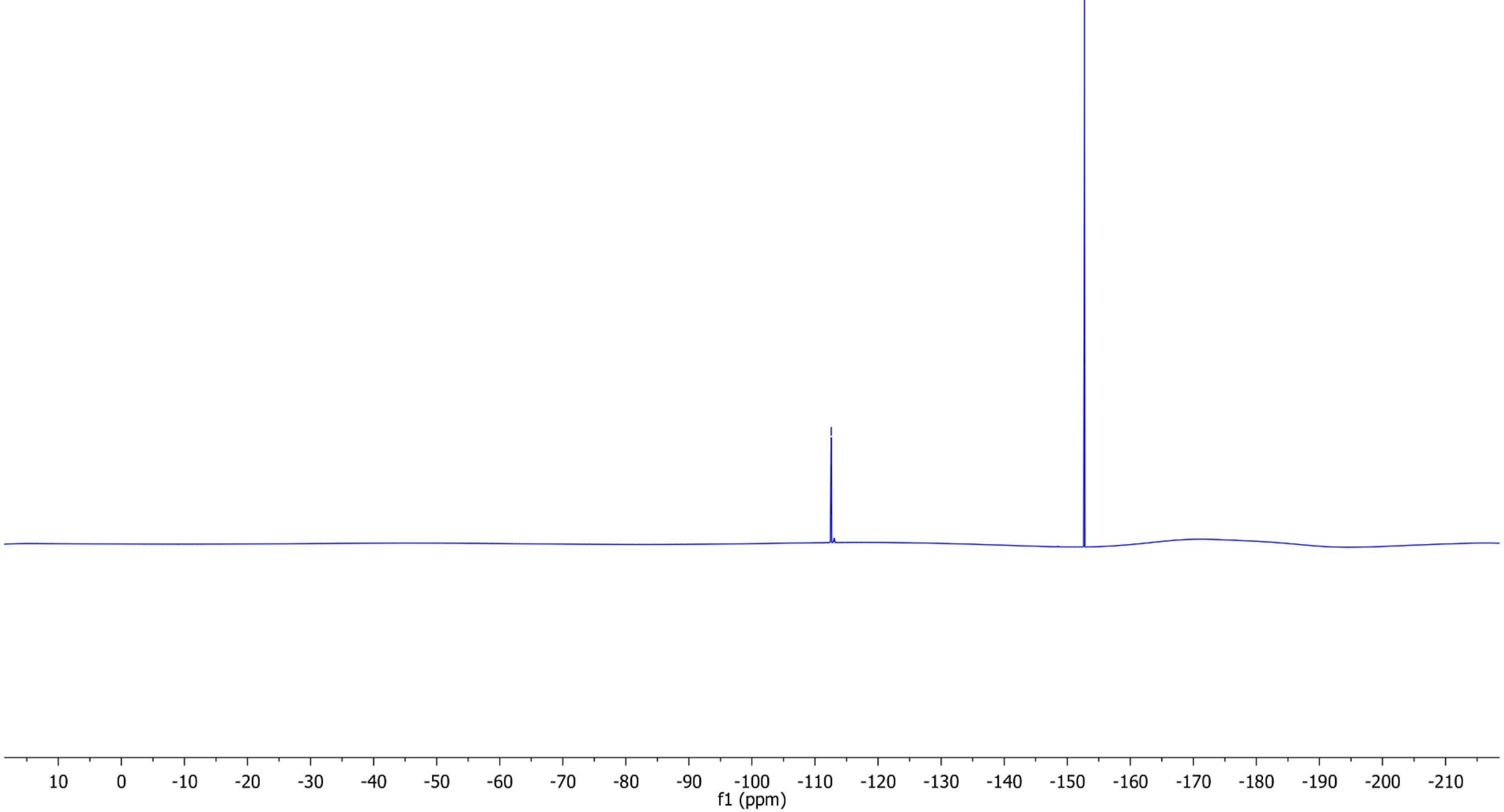

2,4,6-Triphenyl-1-(4-(trifluoromethyl)benzyl)pyridinium tetrafluoroborate (S6)

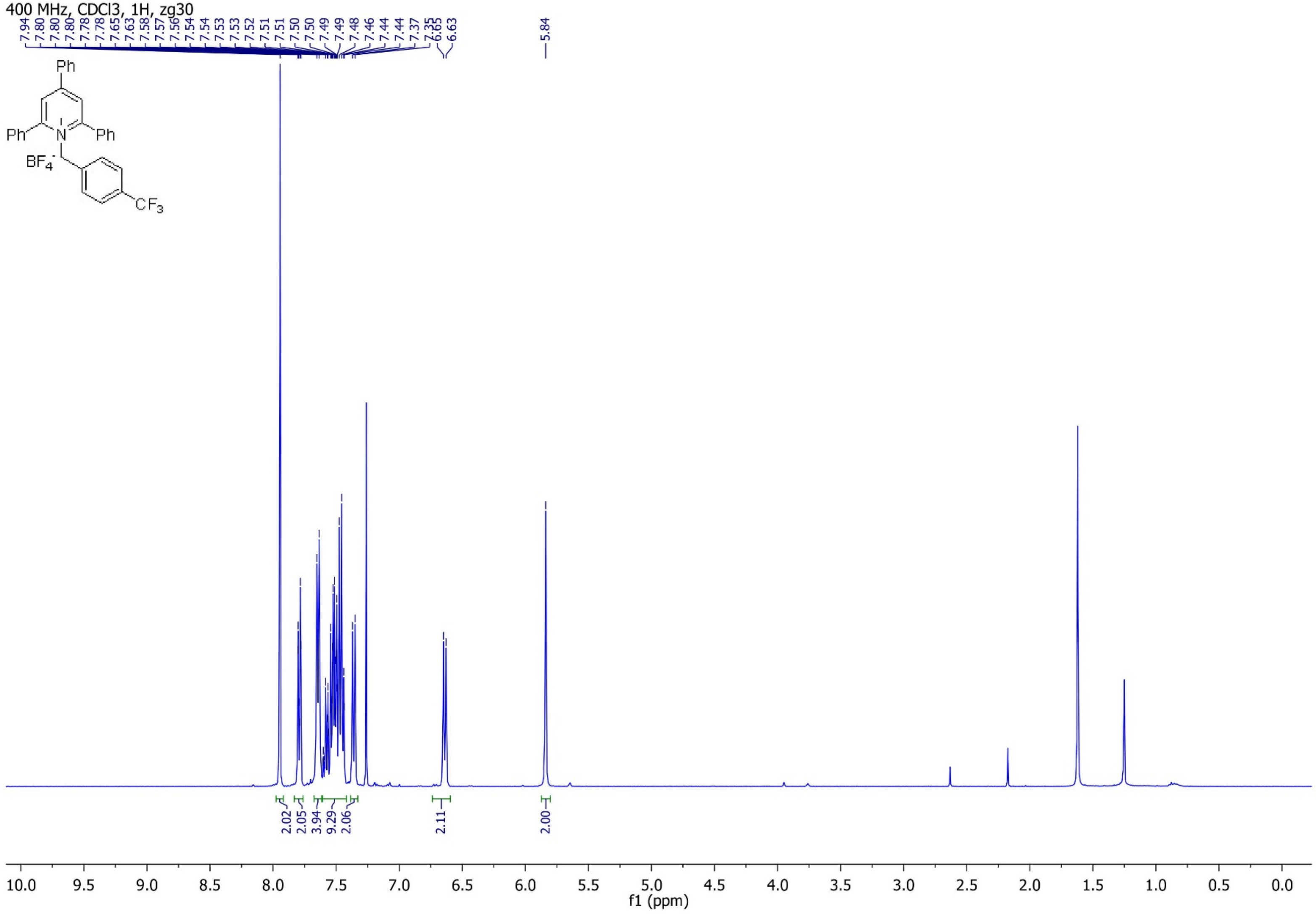

101 MHz, CDCl3, 13C, jmod

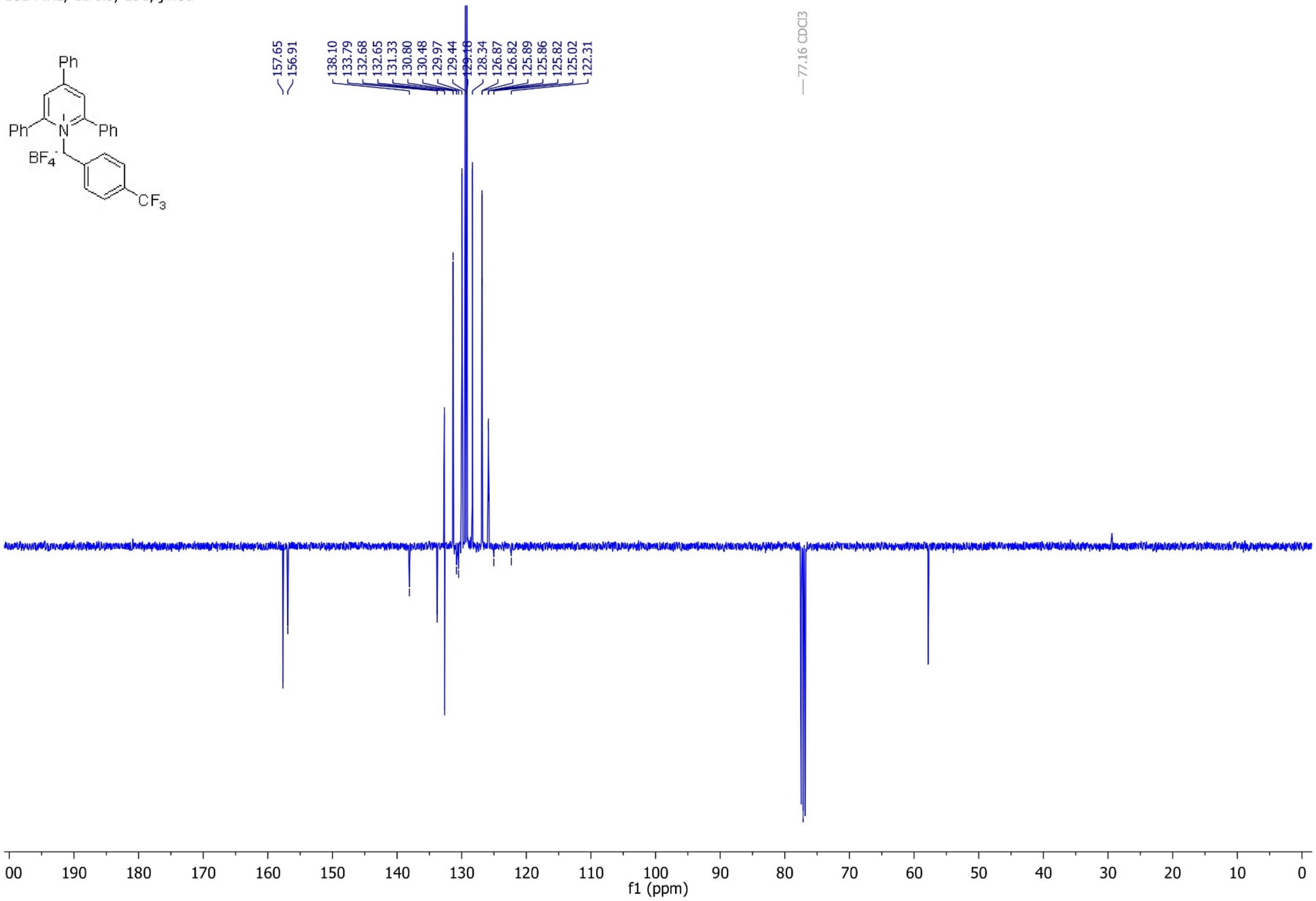

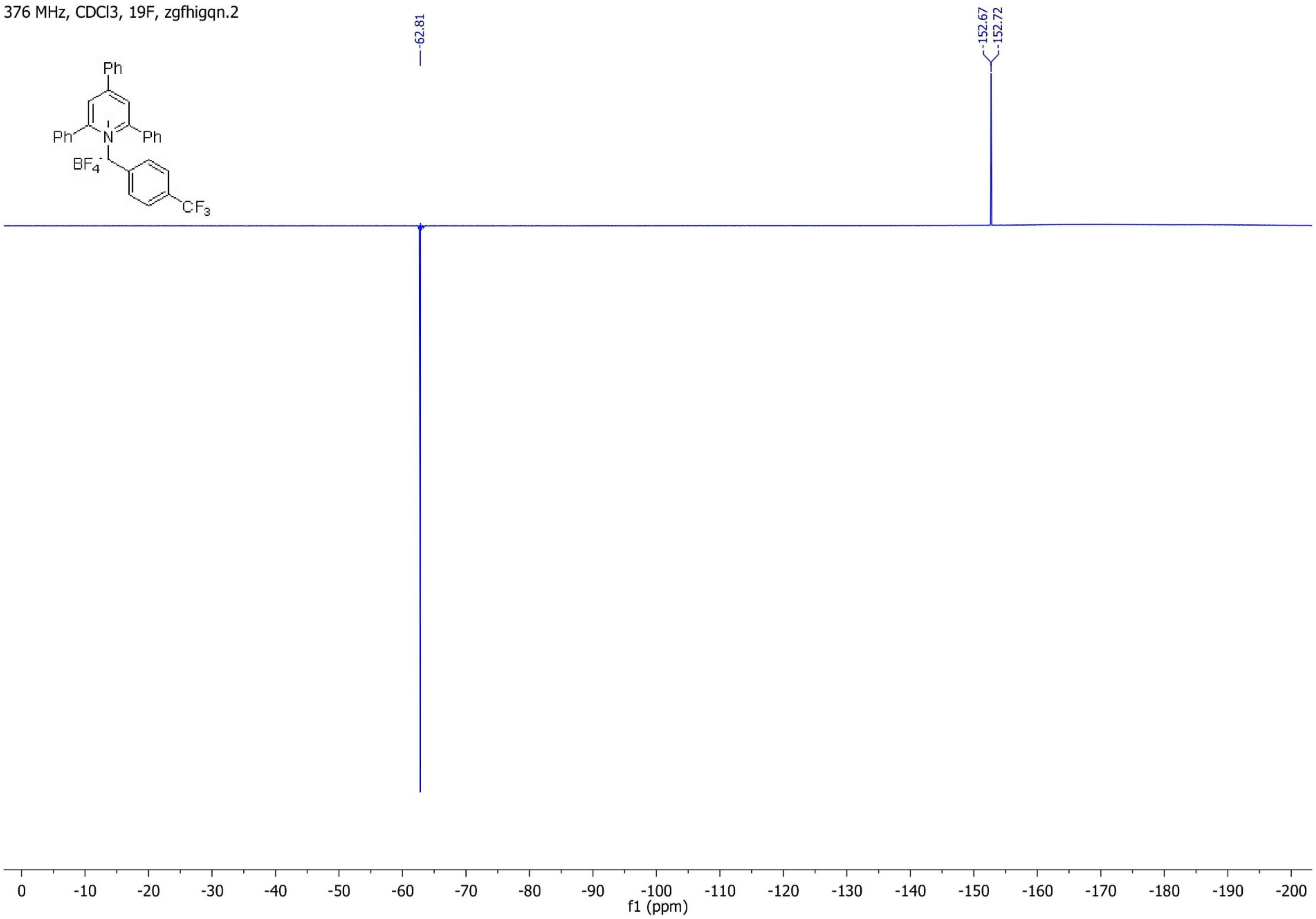
376 MHz, CDCl3, 19F, zgfhigqn.2
-62.81
-152.67
-152.72
Ph
Ph
Ph
N
BF4
CF3
0 -10 -20 -30 -40 -50 -60 -70 -80 -90 -100 -110 -120 -130 -140 -150 -160 -170 -180 -190 -200
f1 (ppm)

2,4,6-Triphenyl-1-(4-methoxybenzyl)pyridin-1-ium tetrafluoroborate (S7)

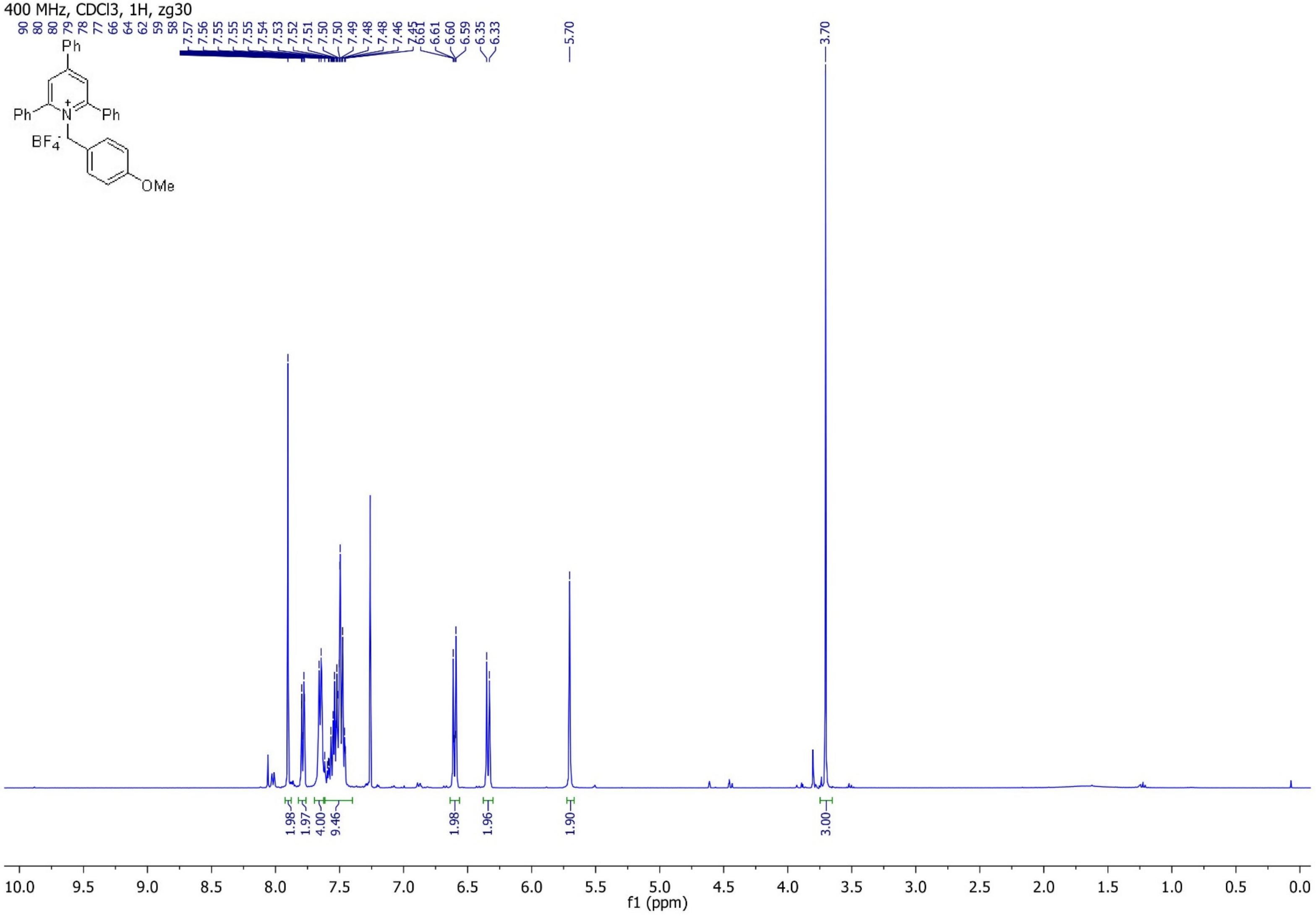

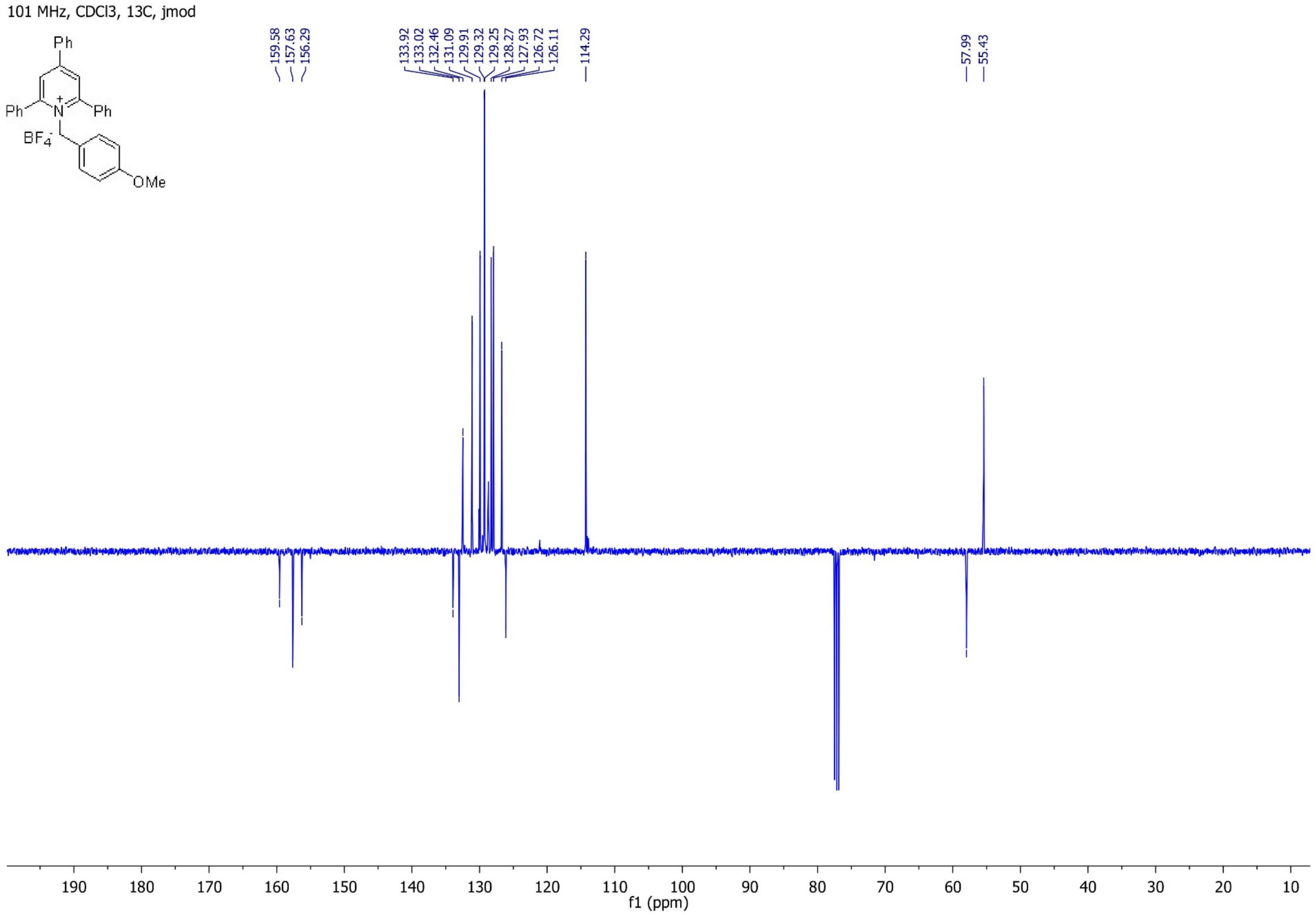

101 MHz, CDCl3, 13C, jmod
Ph
Ph
Ph
N
BF4
OMe
159.58
157.63
156.29
133.92
133.02
132.46
131.09
129.91
129.32
129.25
128.27
127.93
126.72
126.11
114.29
57.99
55.43
190
180
170
160
150
140
130
120
110
100
90
80
70
60
50
40
30
20
10
f1 (ppm)

376 MHz, CDCl3, 19F, zgfhigqn.2

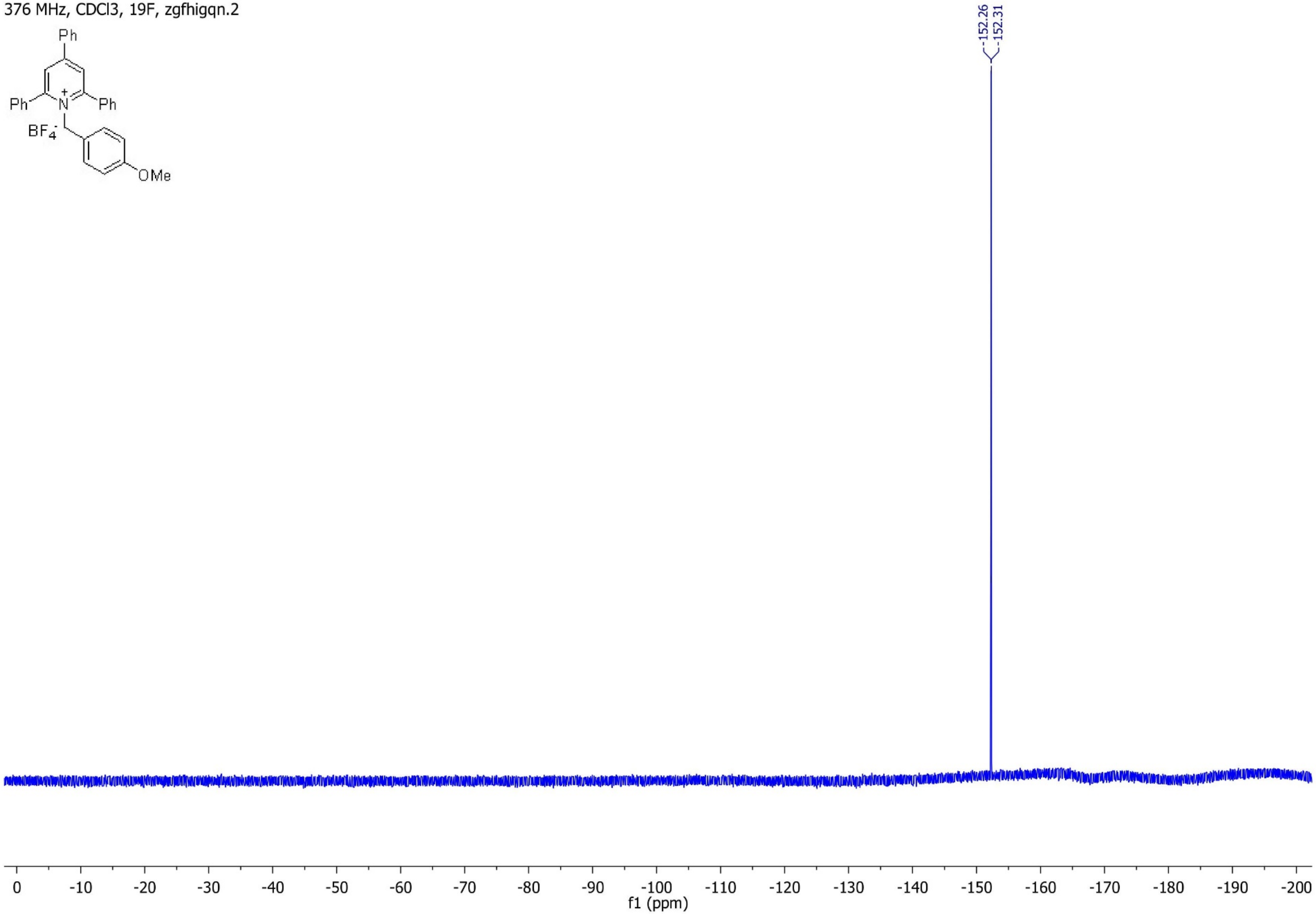

2,4,6-Triphenyl-1-(4-bromobenzyl)pyridin-1-ium tetrafluoroborate (S8)

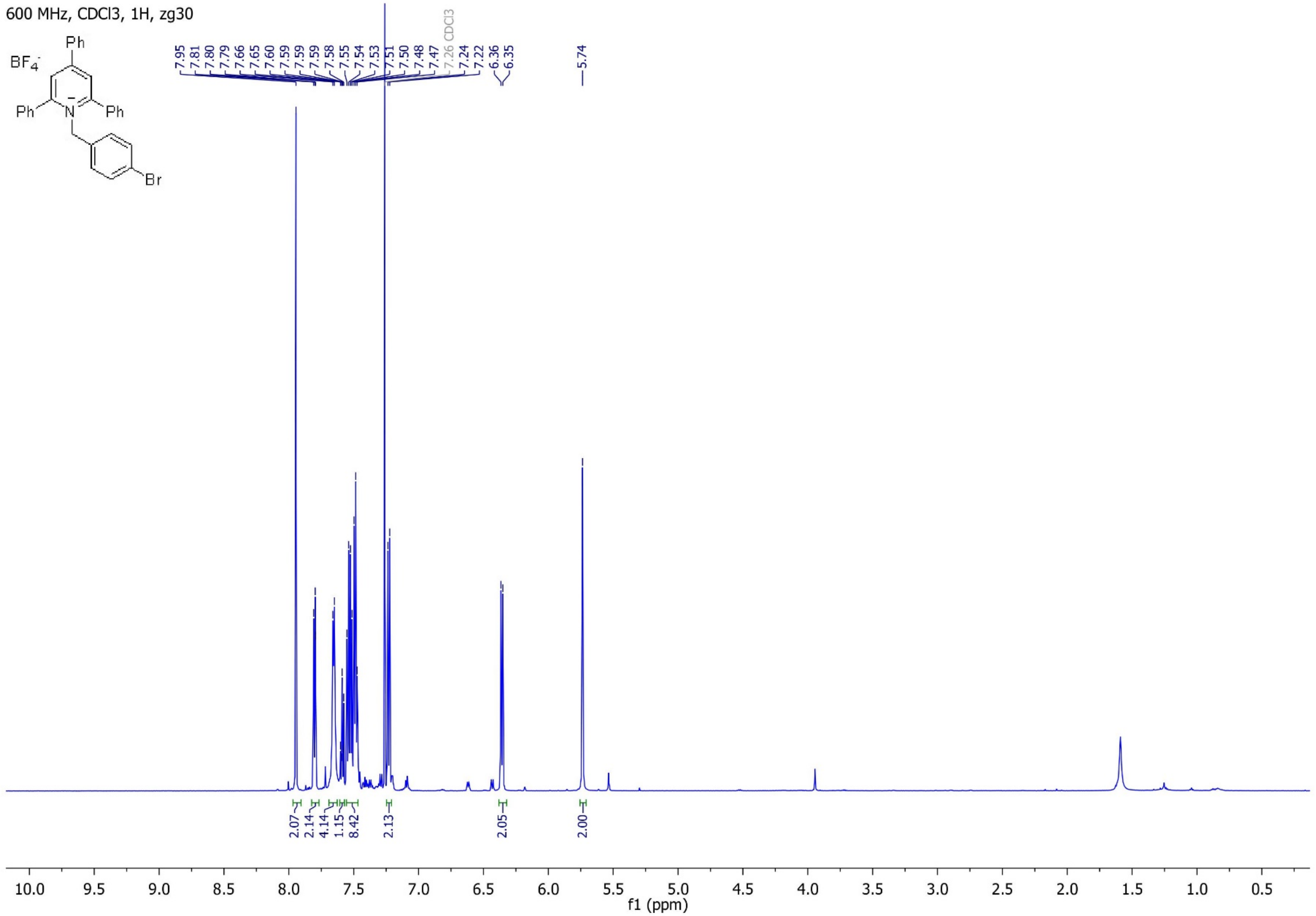

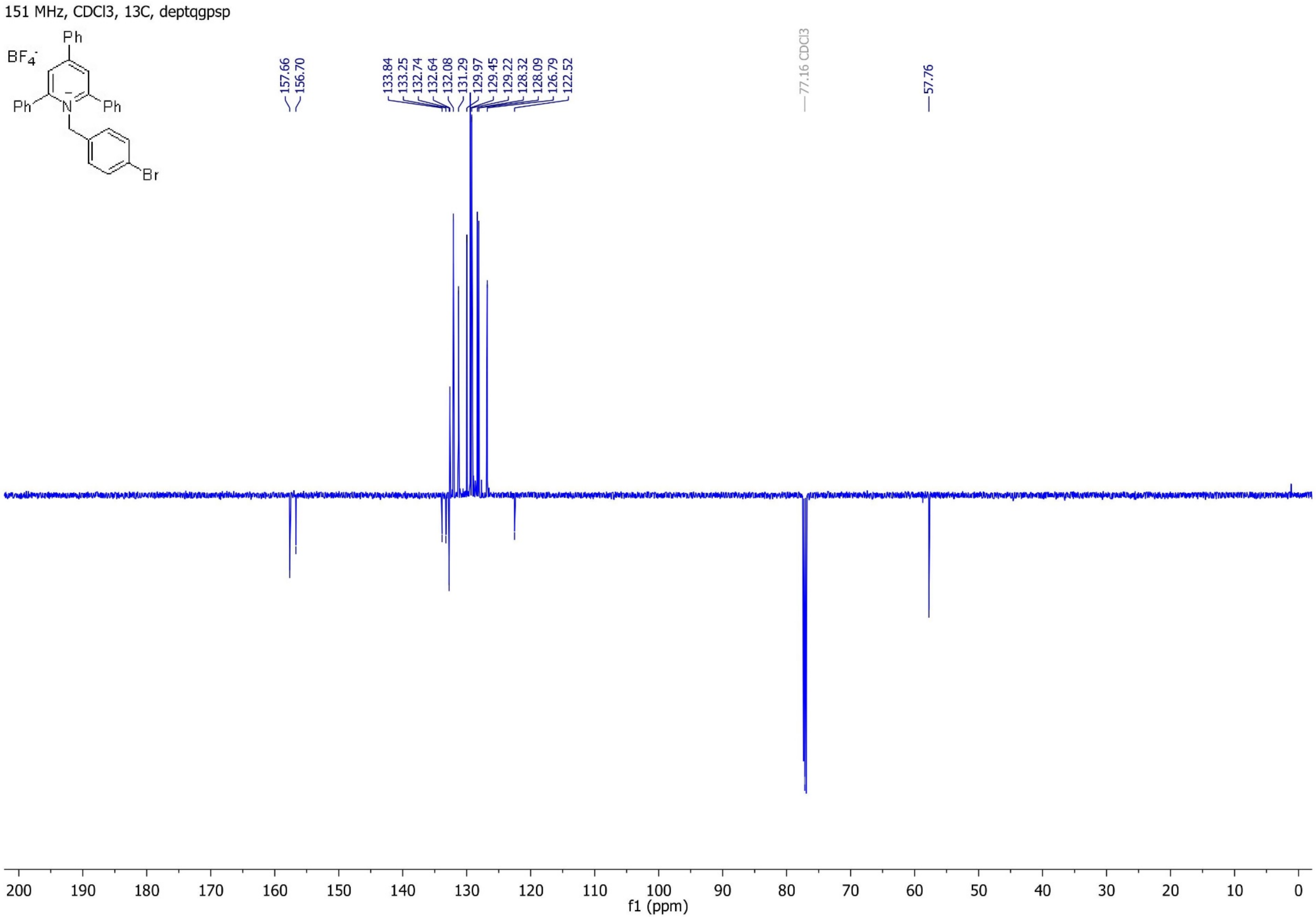
151 MHz, CDCl3, 13C, deptqgpsp
Ph
BF_4^-
Ph
N
Ph
Br
157.66
156.70
133.84
133.25
132.74
132.64
132.08
131.29
129.97
129.45
129.22
128.32
128.09
126.79
122.52
77.16 CDCl3
57.76
200 190 180 170 160 150 140 130 120 110 100 90 80 70 60 50 40 30 20 10 0
f1 (ppm)

565 MHz, CDCl3, 19F, zgflqn

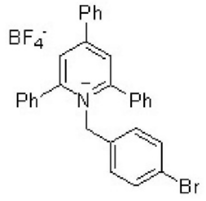

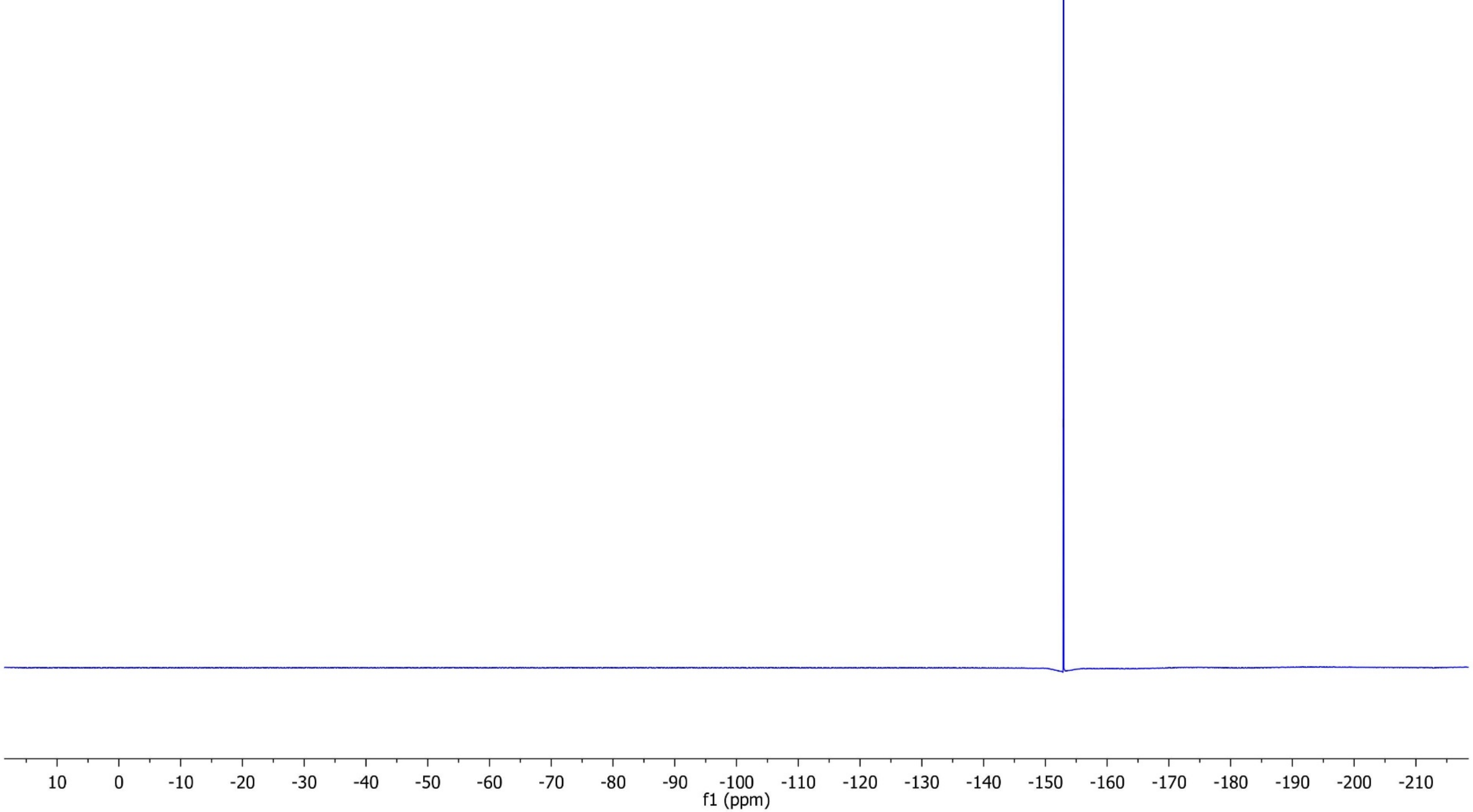

2,4,6-Triphenyl-1-(2-methylbenzyl)pyridin-1-ium tetrafluoroborate (S9)

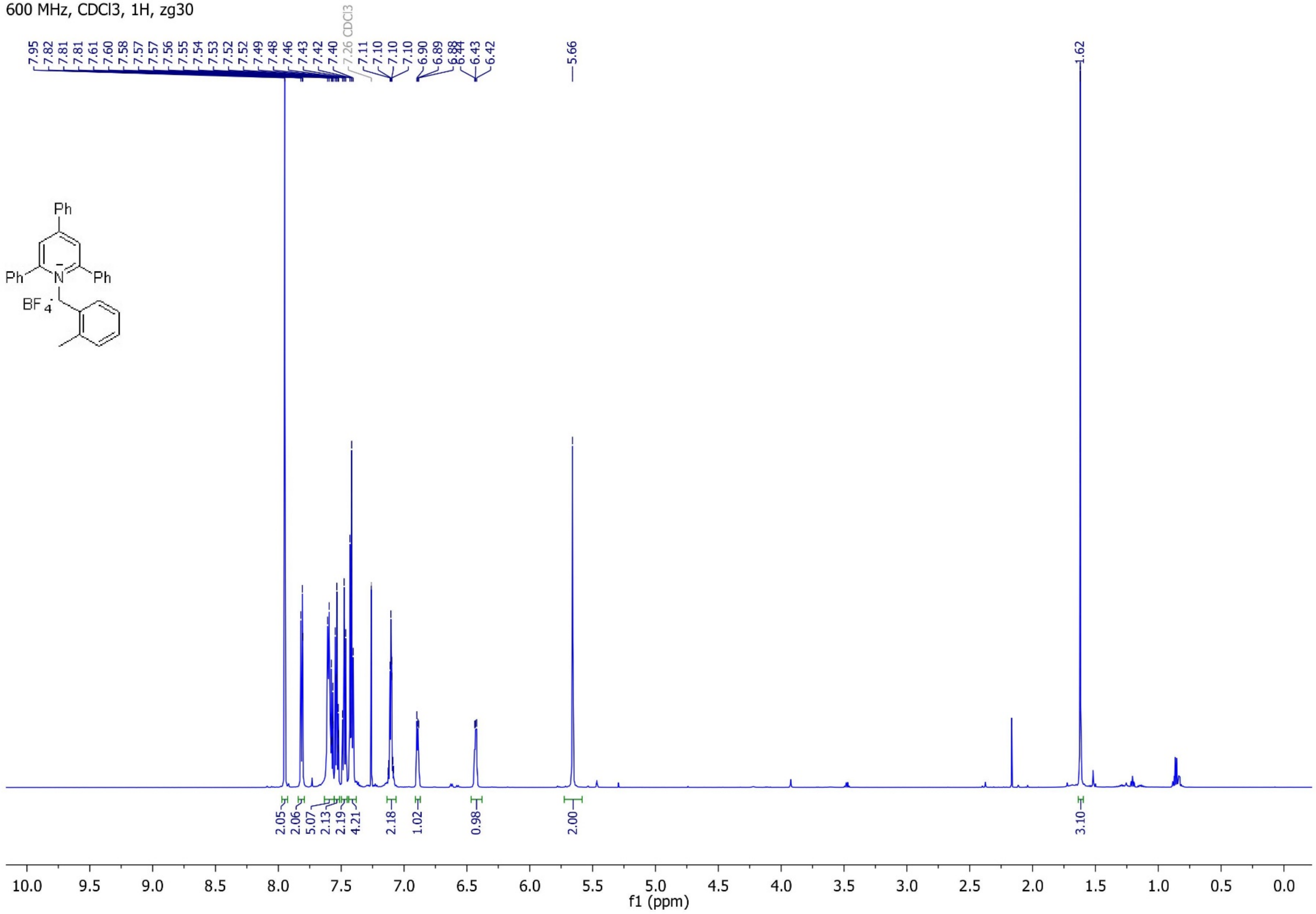

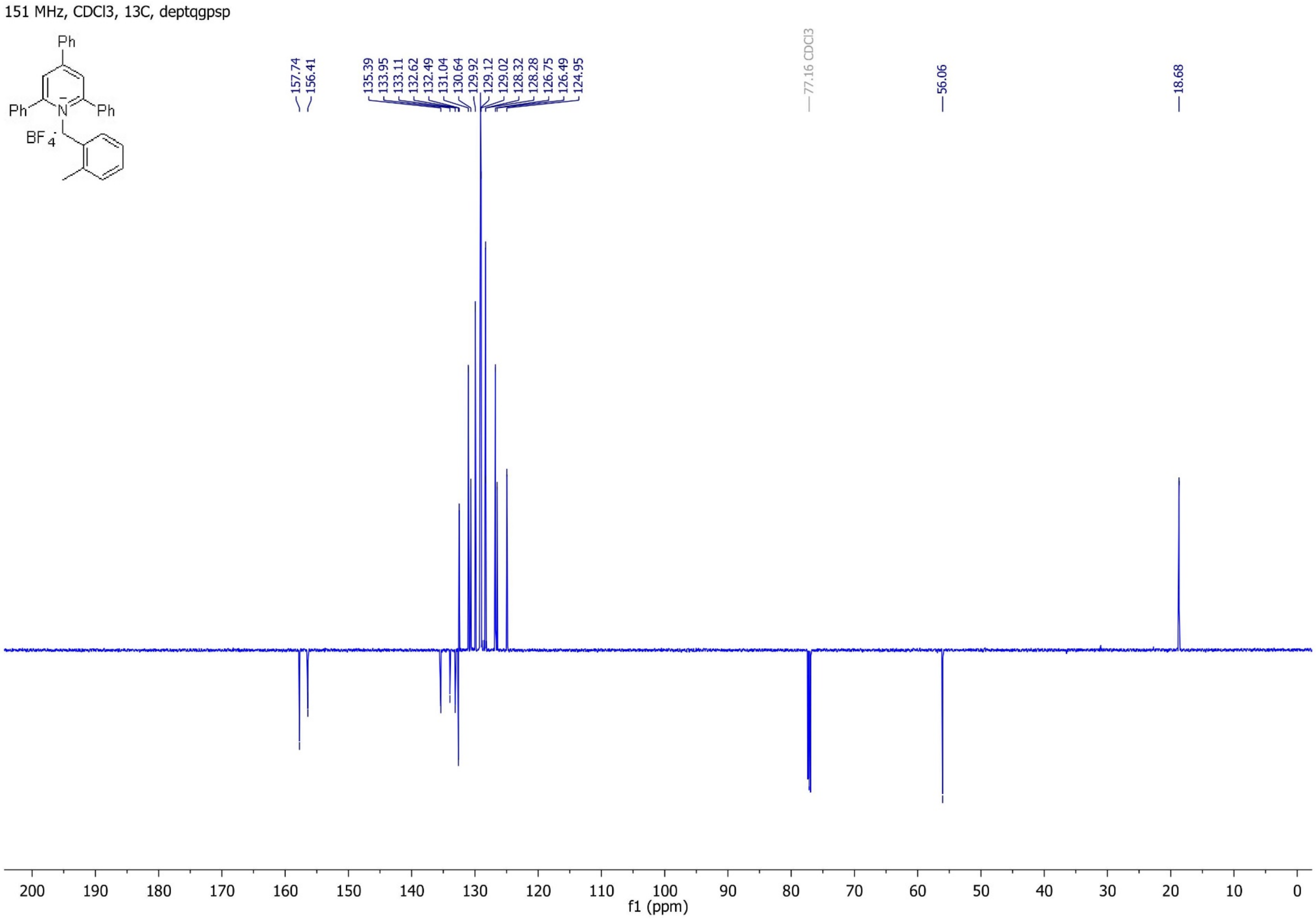

151 MHz, CDCl3, 13C, deptqgpsp
Ph
Ph
Ph
N
BF4
157.74
156.41
135.39
133.95
133.11
132.62
132.49
131.04
130.64
129.92
129.12
129.02
128.32
128.28
126.75
126.49
124.95
77.16 CDCl3
56.06
18.68
200 190 180 170 160 150 140 130 120 110 100 90 80 70 60 50 40 30 20 10 0
f1 (ppm)

565 MHz, CDCl3, 19F, zgflqn

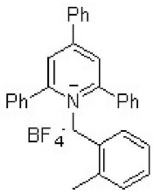

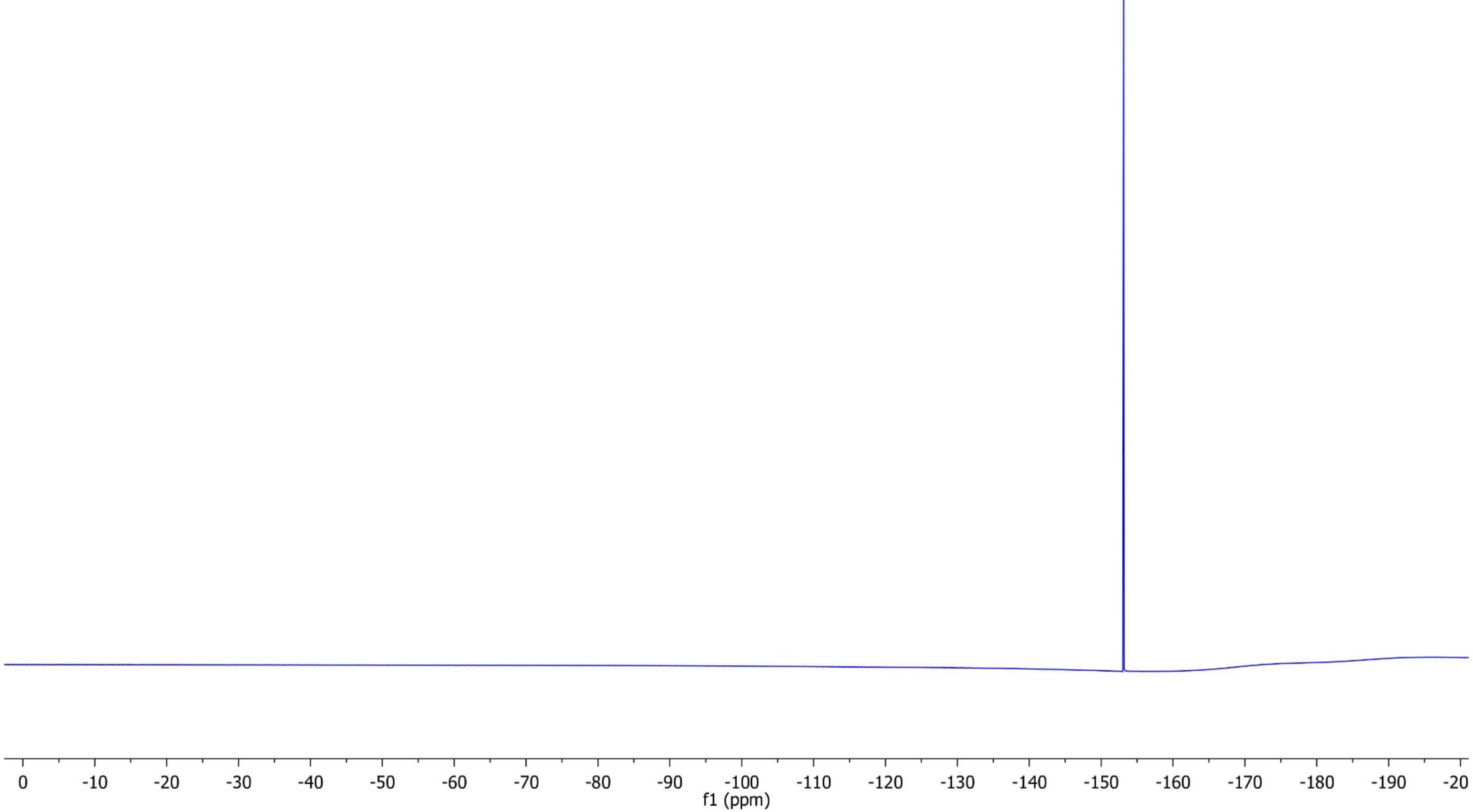

2,4,6-Triphenyl-1-(3-methylbenzyl)pyridin-1-ium tetrafluoroborate (S10)

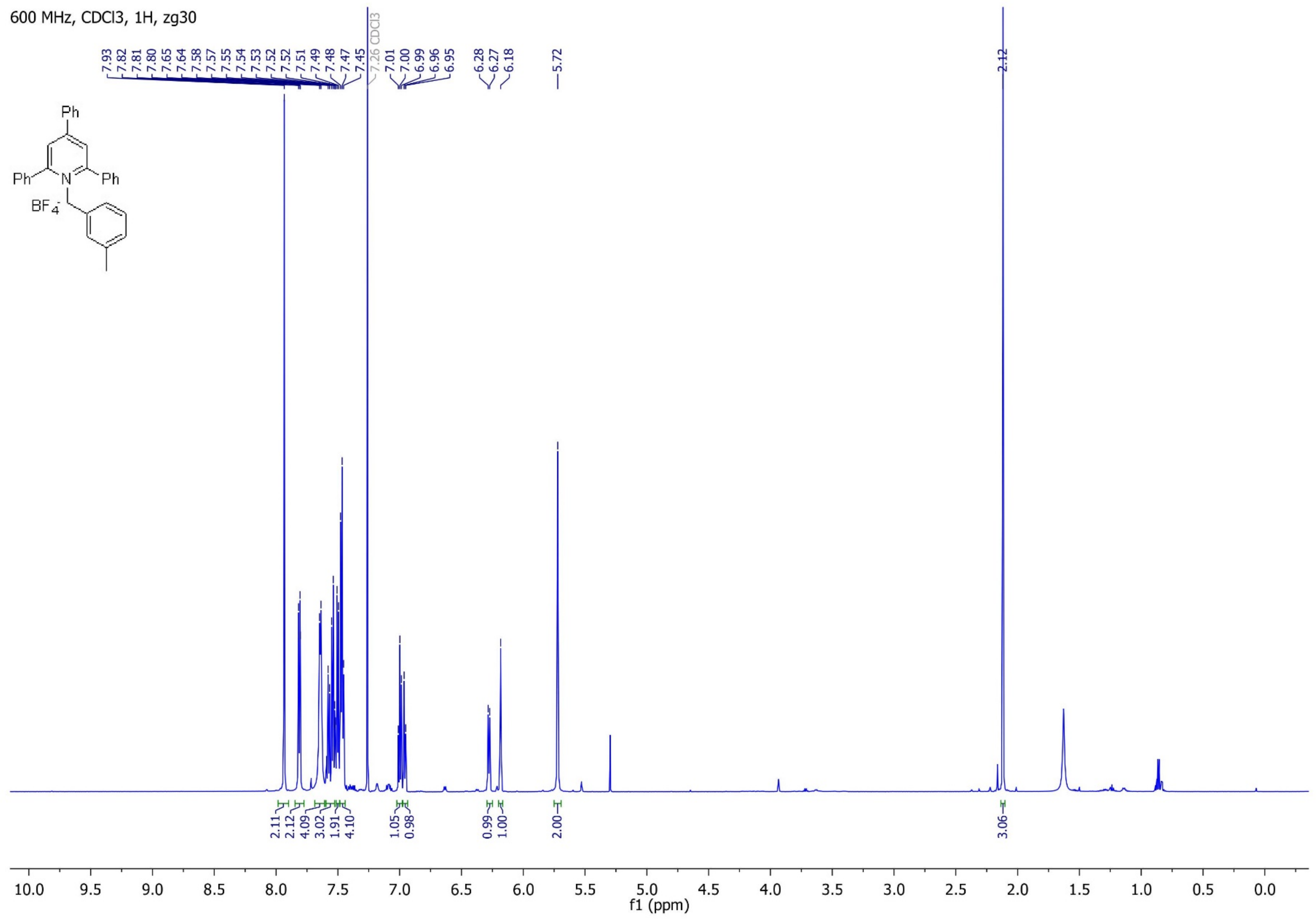

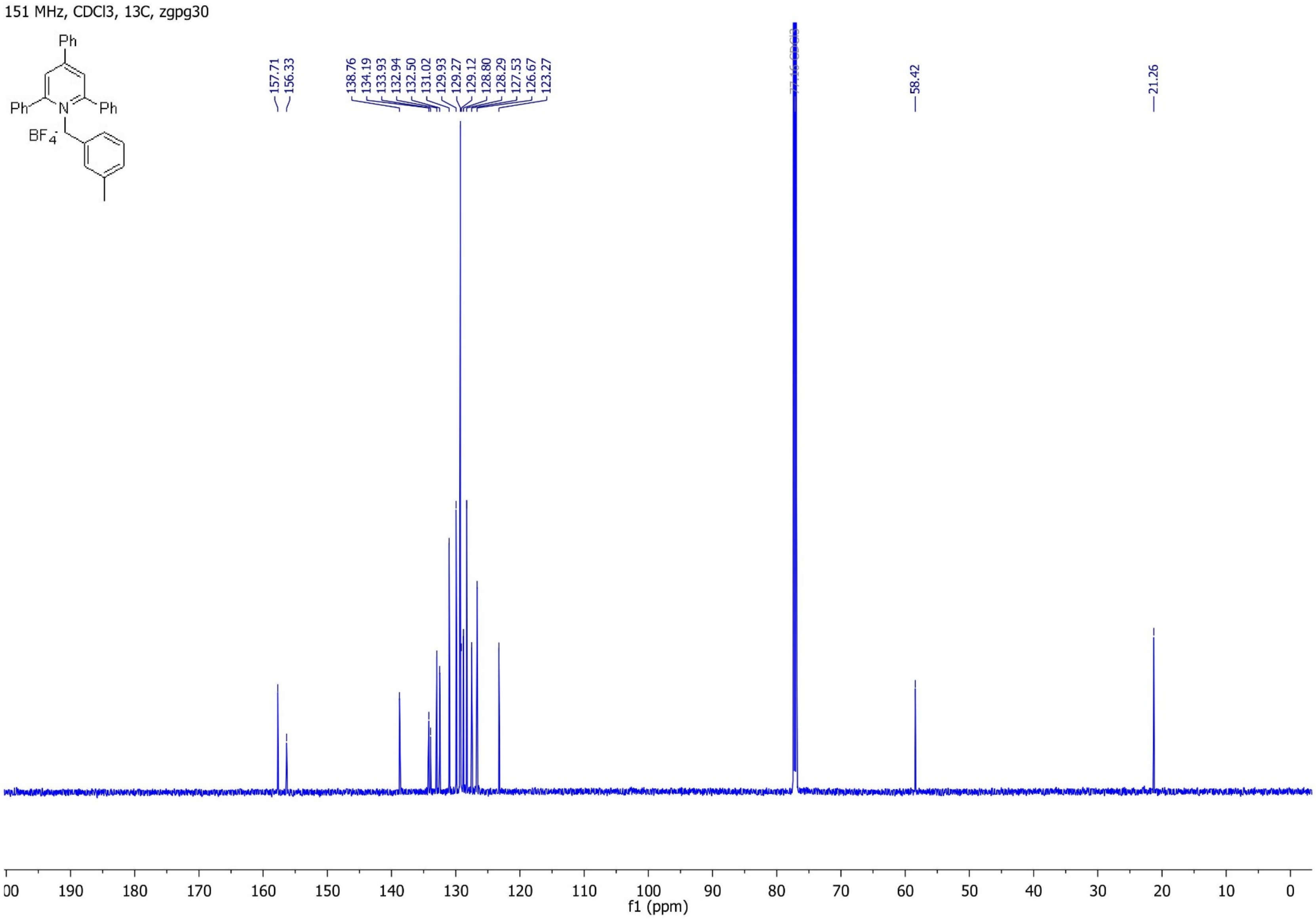
151 MHz, CDCl3, 13C, zgpg30
Ph
Ph
N
Ph
BF4-
157.71
156.33
138.76
134.19
133.93
132.94
132.50
131.02
129.93
129.27
129.12
128.80
128.29
127.53
126.67
123.27
58.42
21.26
200
190
180
170
160
150
140
130
120
110
100
90
80
70
60
50
40
30
20
10
0
f1 (ppm)

376 MHz, CDCl3, 19F, zgflqn

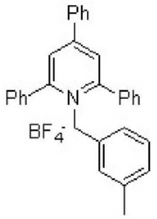

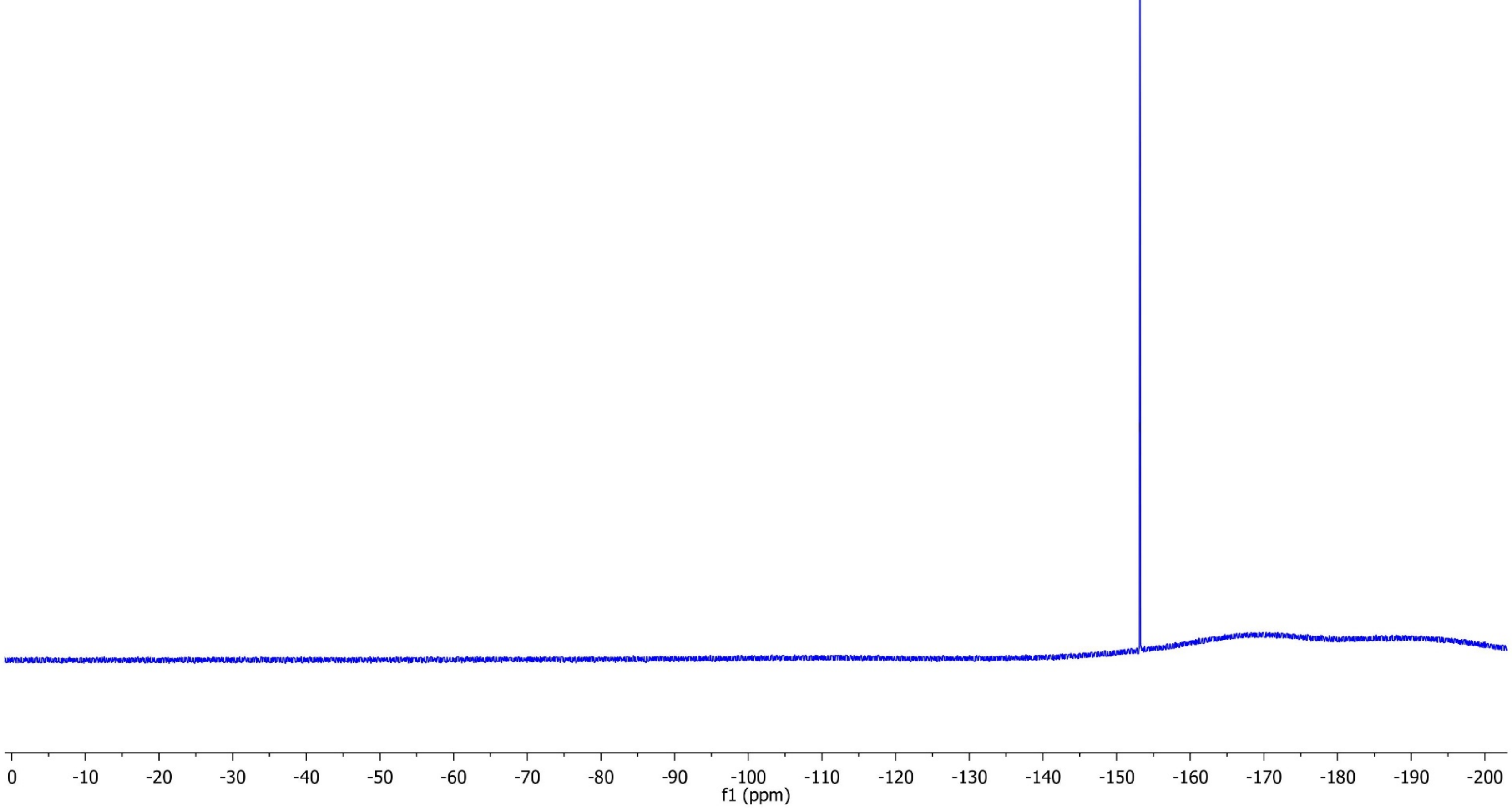

2,4,6-Triphenyl-1-(4-methylbenzyl)pyridin-1-ium tetrafluoroborate (S11)

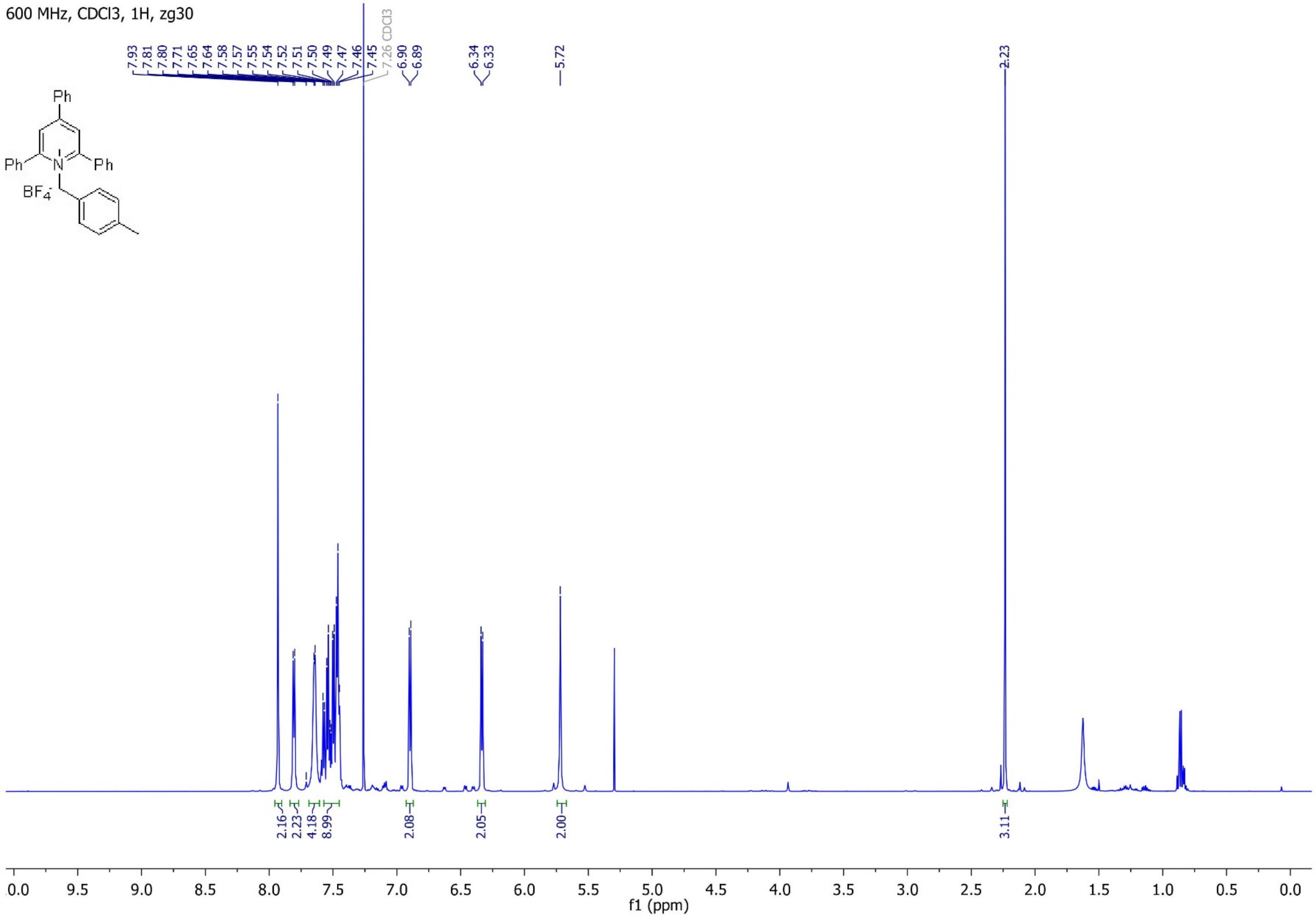

151 MHz, CDCl3, 13C, zgpg30

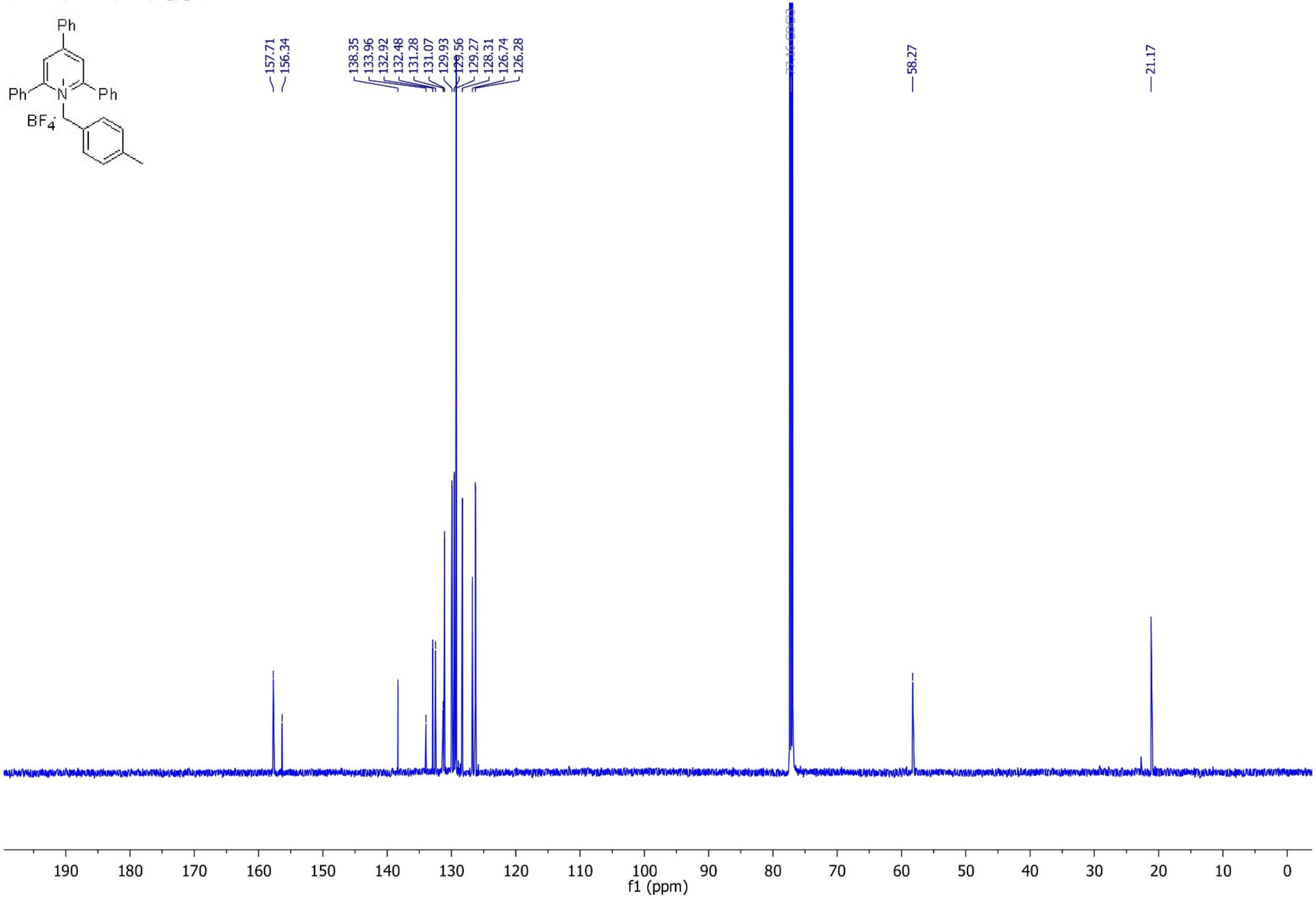

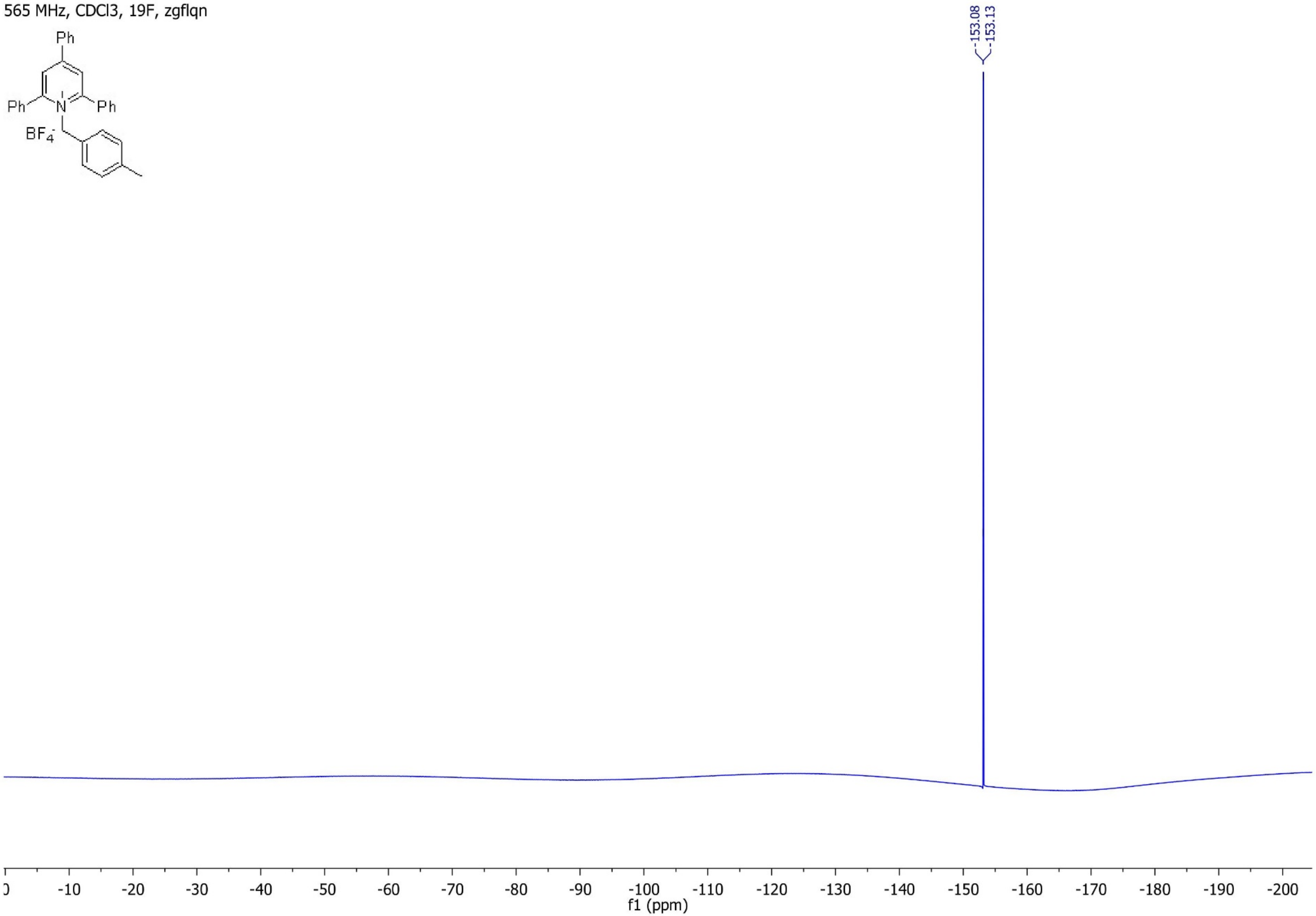
565 MHz, CDCl3, 19F, zgflqn
Ph
Ph
N
Ph
BF4
-153.08
-153.13
0
-10
-20
-30
-40
-50
-60
-70
-80
-90
-100
-110
-120
-130
-140
-150
-160
-170
-180
-190
-200
f1 (ppm)

1-(Naphthalen-2-ylmethyl)-2,4,6-triphenylpyridin-1-ium tetrafluoroborate (S12)

600 MHz, CDCl3, 1H, zg30

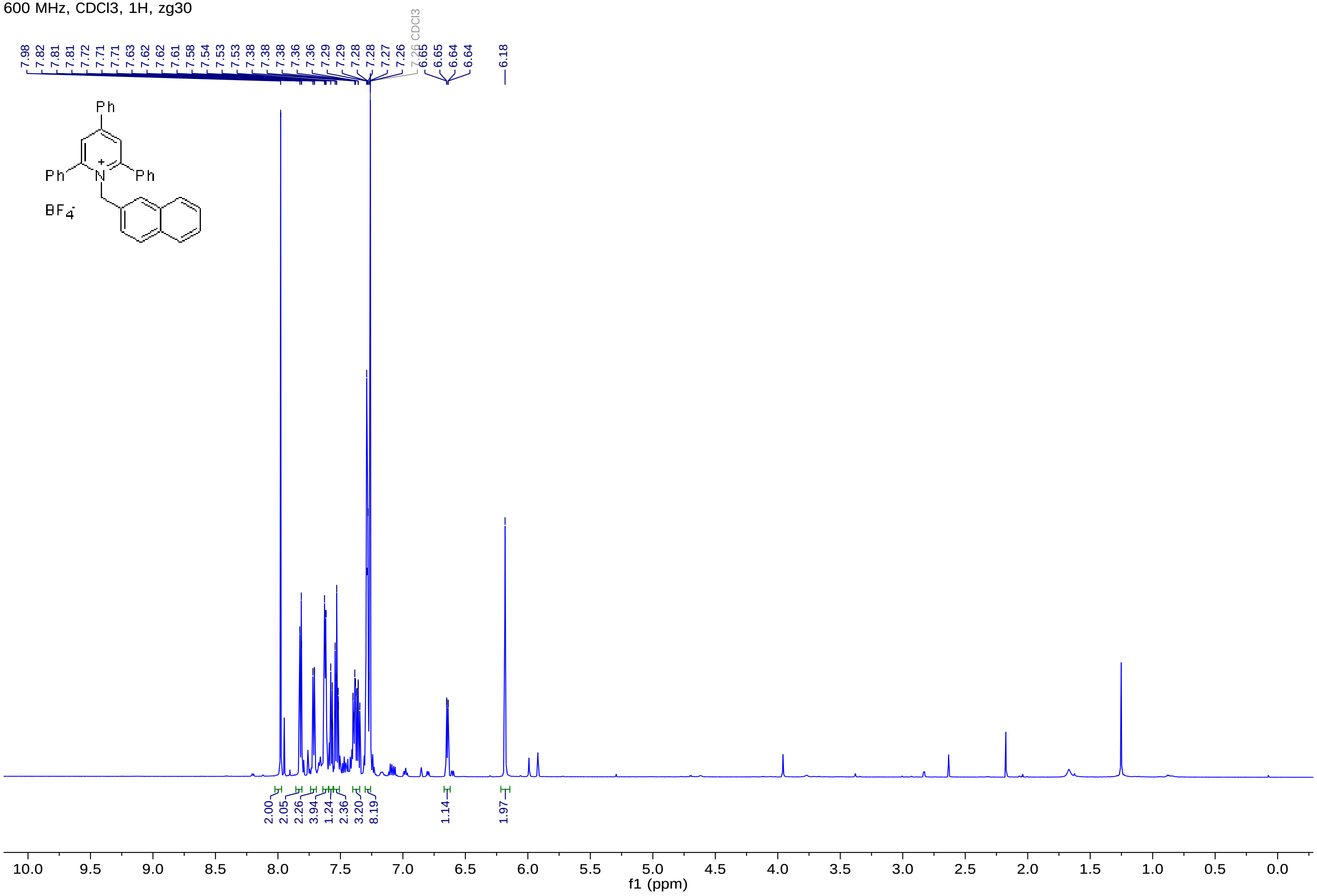

151 MHz, CDCl3, 13C, deptqgpsp

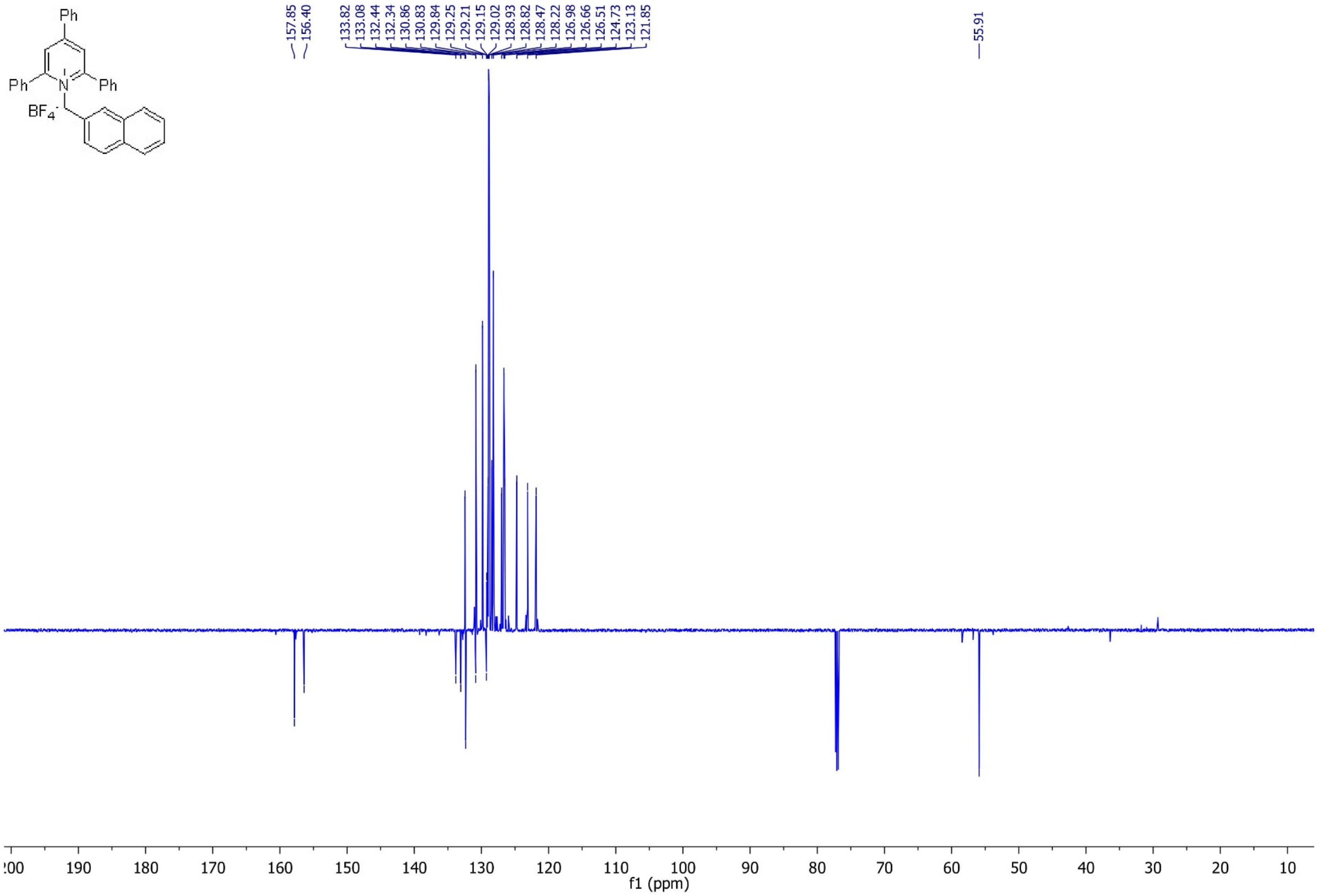

565 MHz, CDCl3, 19F, zgflqn

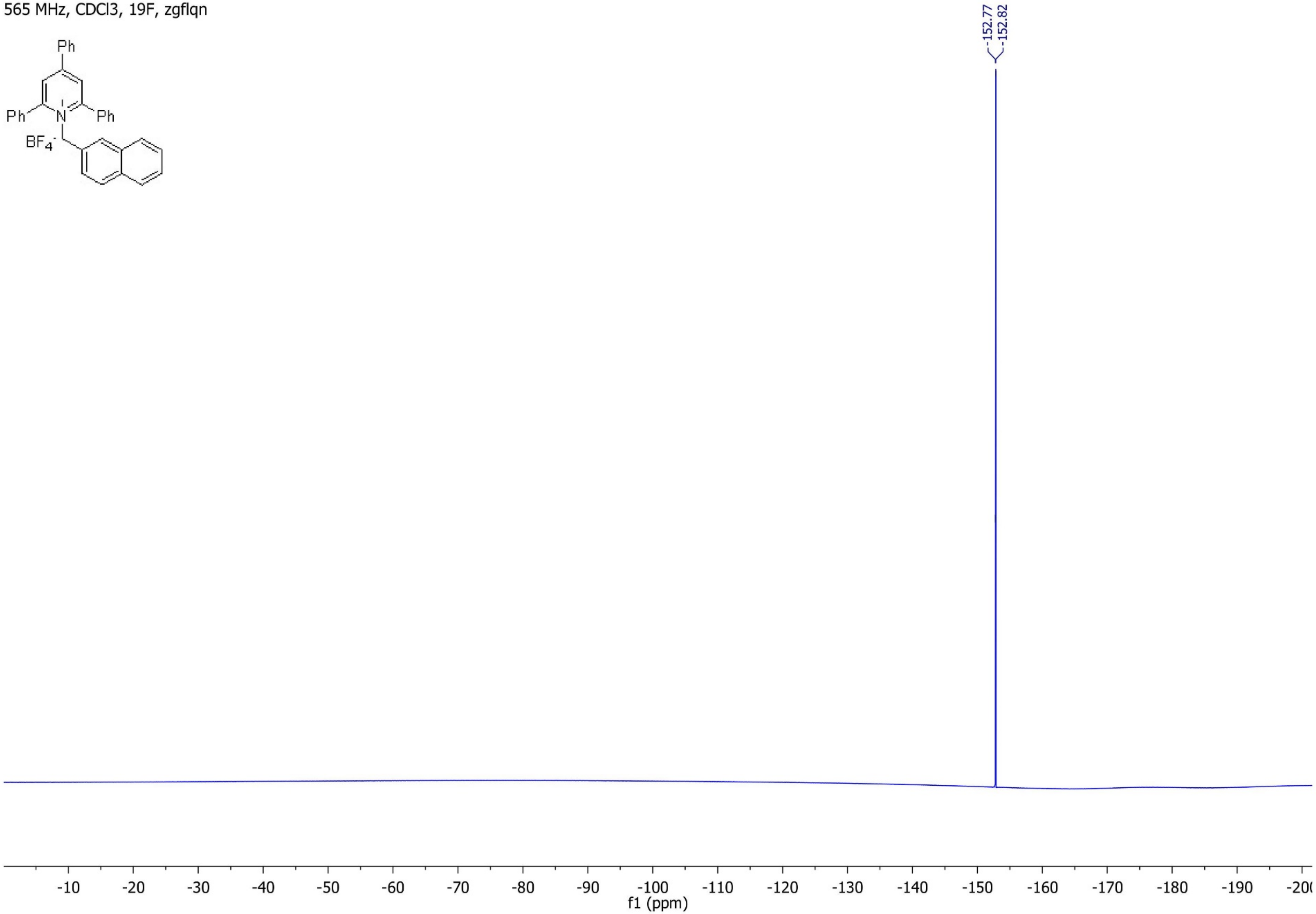

2,4,6-Triphenyl-1-(pyridin-2-ylmethyl)pyridin-1-ium tetrafluoroborate (S13)

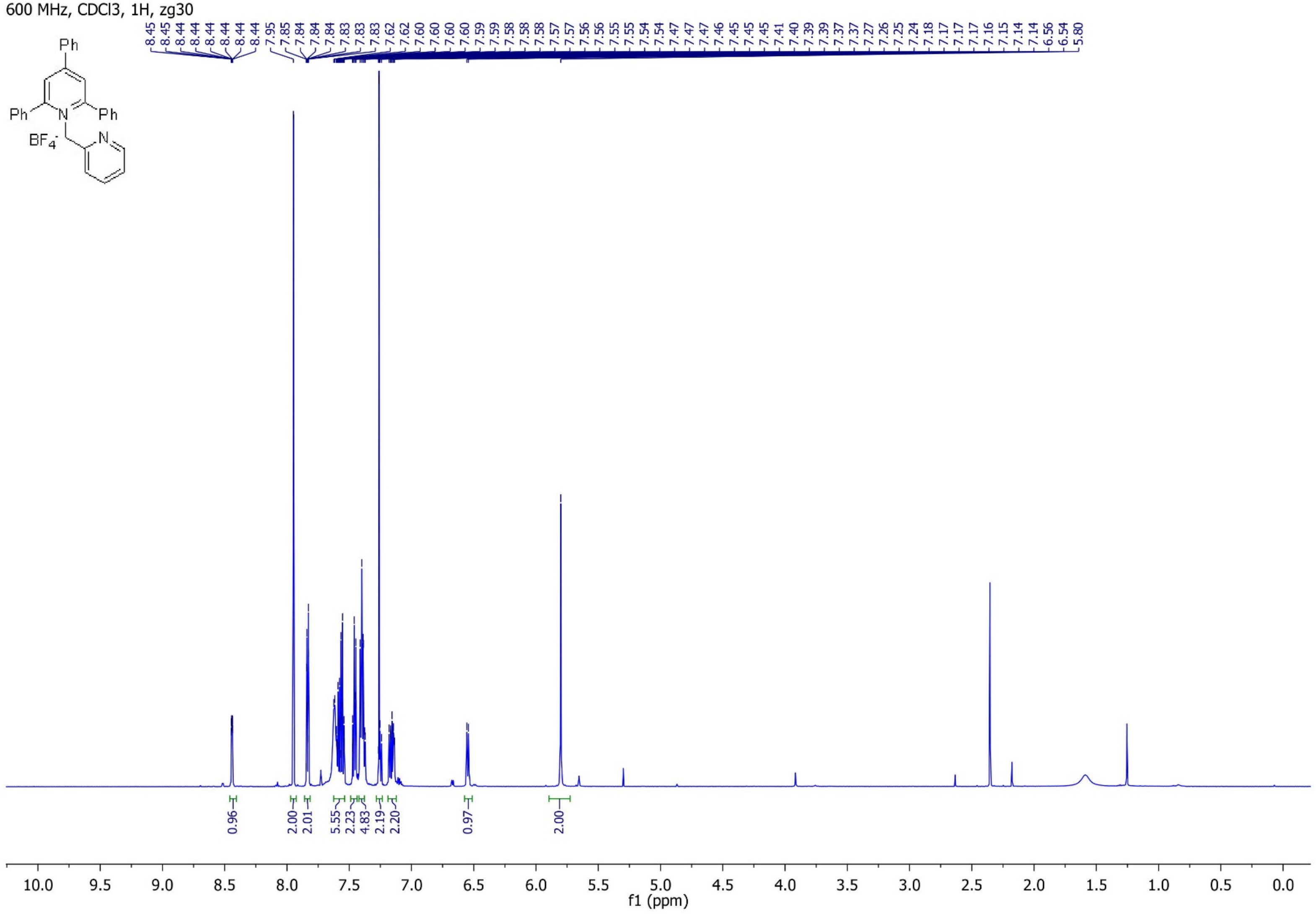

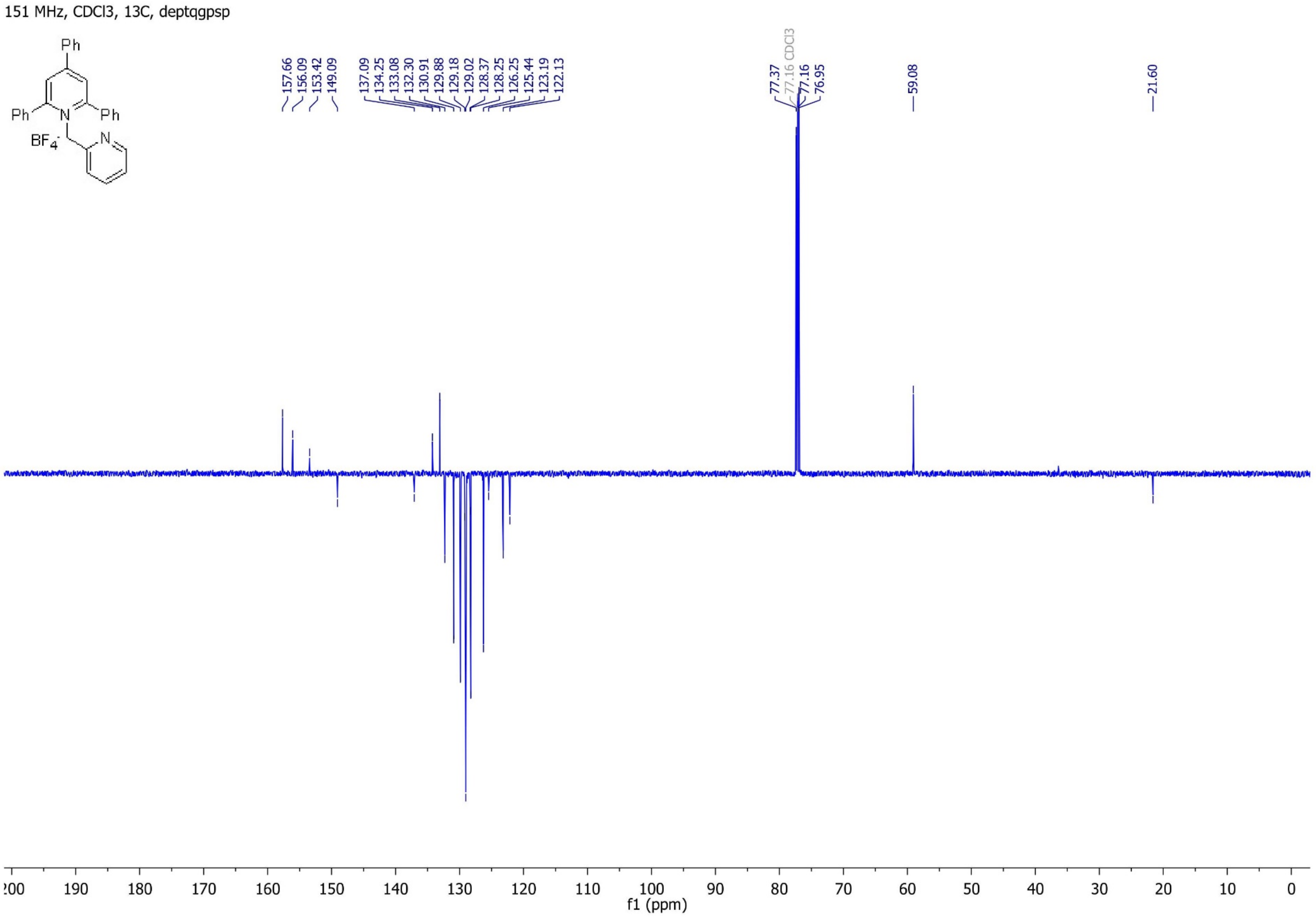

151 MHz, CDCl3, 13C, deptqgpsp
Ph
Ph
Ph
N
BF4-
N
157.66
156.09
153.42
149.09
137.09
134.25
133.08
132.30
130.91
129.88
129.18
129.02
128.37
128.25
126.25
125.44
123.19
122.13
77.37
77.16 CDCl3
77.16
76.95
59.08
21.60
200 190 180 170 160 150 140 130 120 110 100 90 80 70 60 50 40 30 20 10 0
f1 (ppm)

565 MHz, CDCl3, 19F, zgflqn

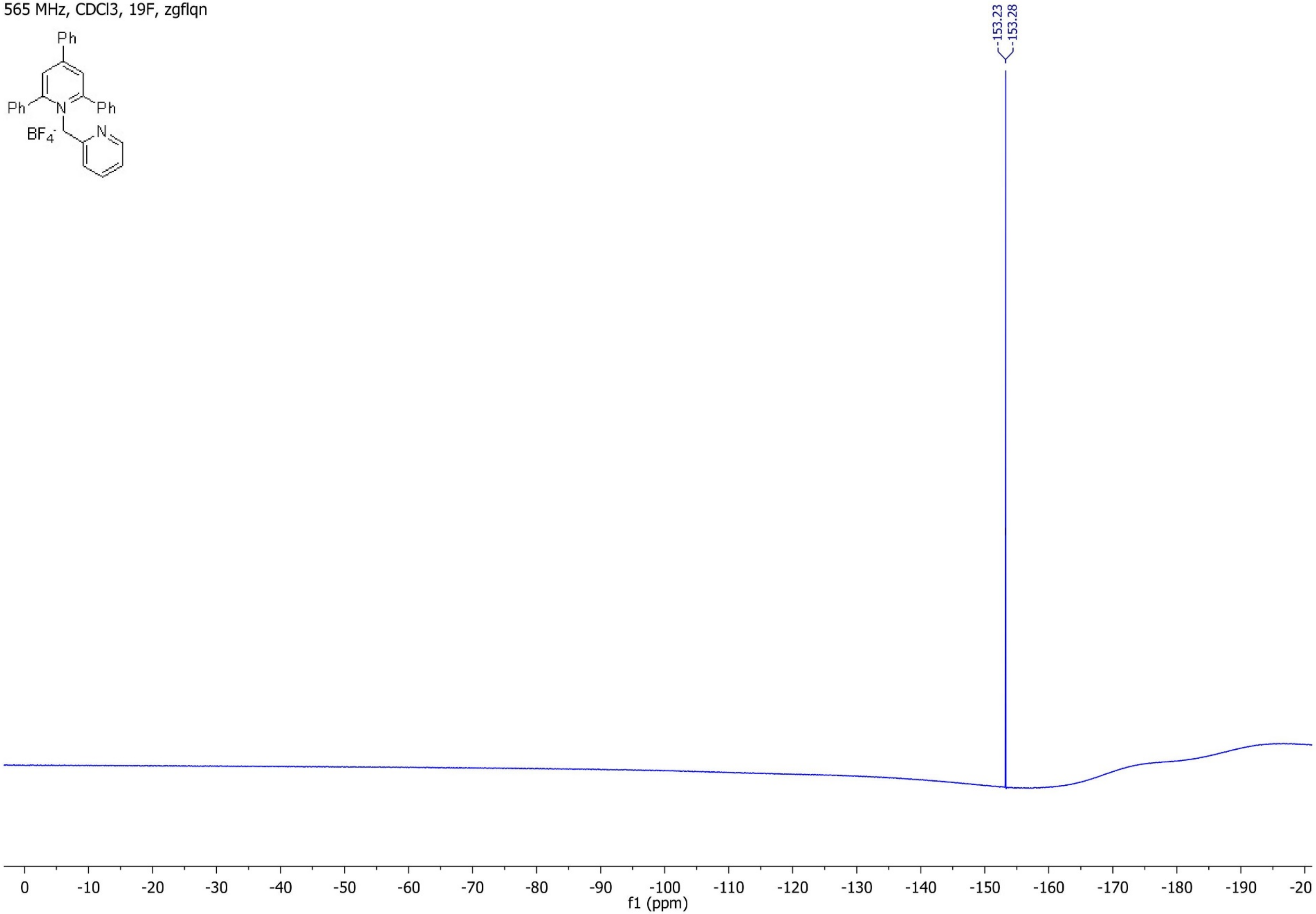

2,4,6-Triphenyl-1-(pyridin-3-ylmethyl)pyridin-1-ium tetrafluoroborate (S14)

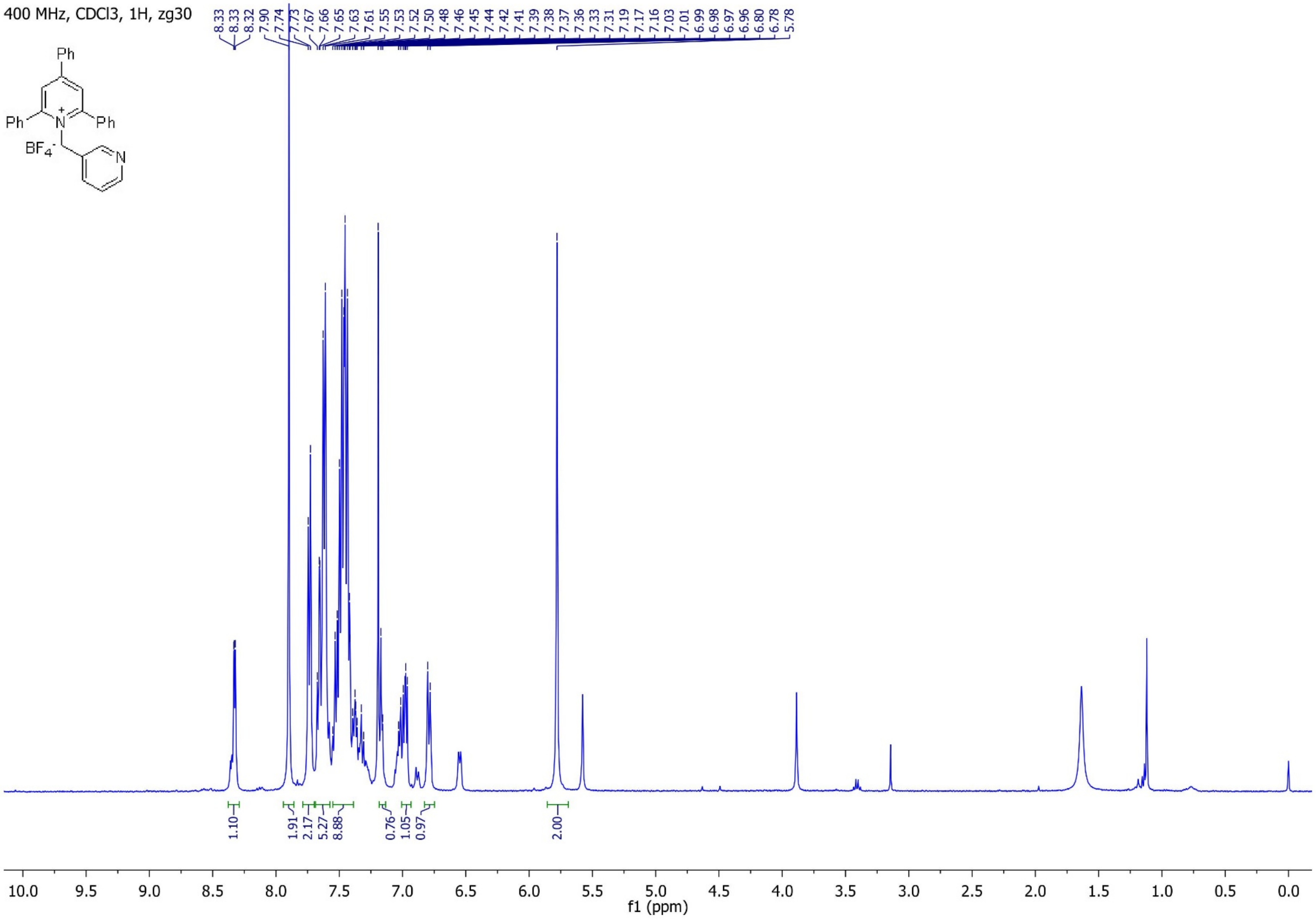

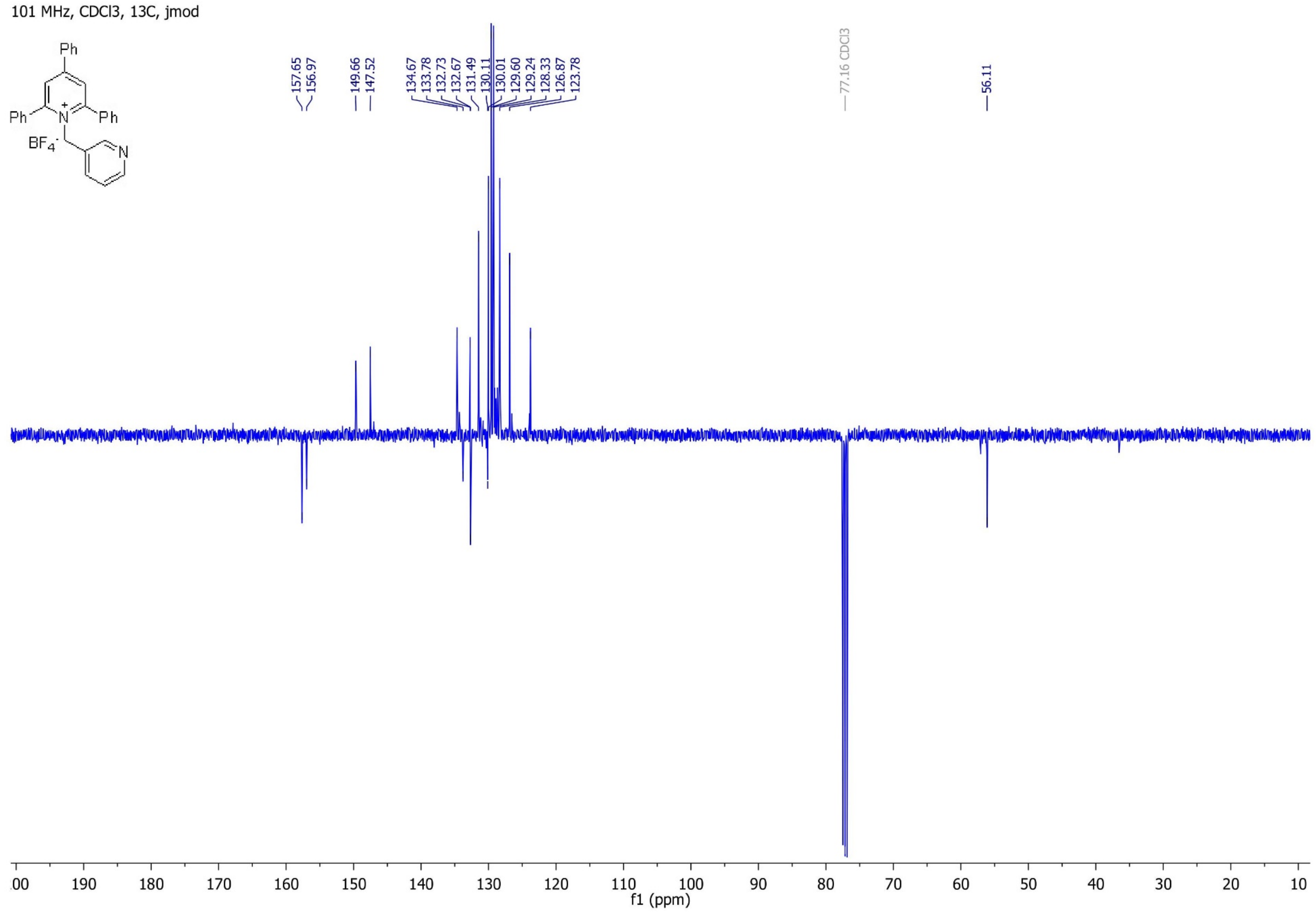
101 MHz, CDCl3, 13C, jmod
Ph
Ph
Ph
N
BF4-
N
157.65
156.97
149.66
147.52
134.67
133.78
132.73
132.67
131.49
130.11
130.01
129.60
129.24
128.33
126.87
123.78
77.16 CDCl3
56.11
00
190
180
170
160
150
140
130
120
110
100
90
80
70
60
50
40
30
20
10
f1 (ppm)

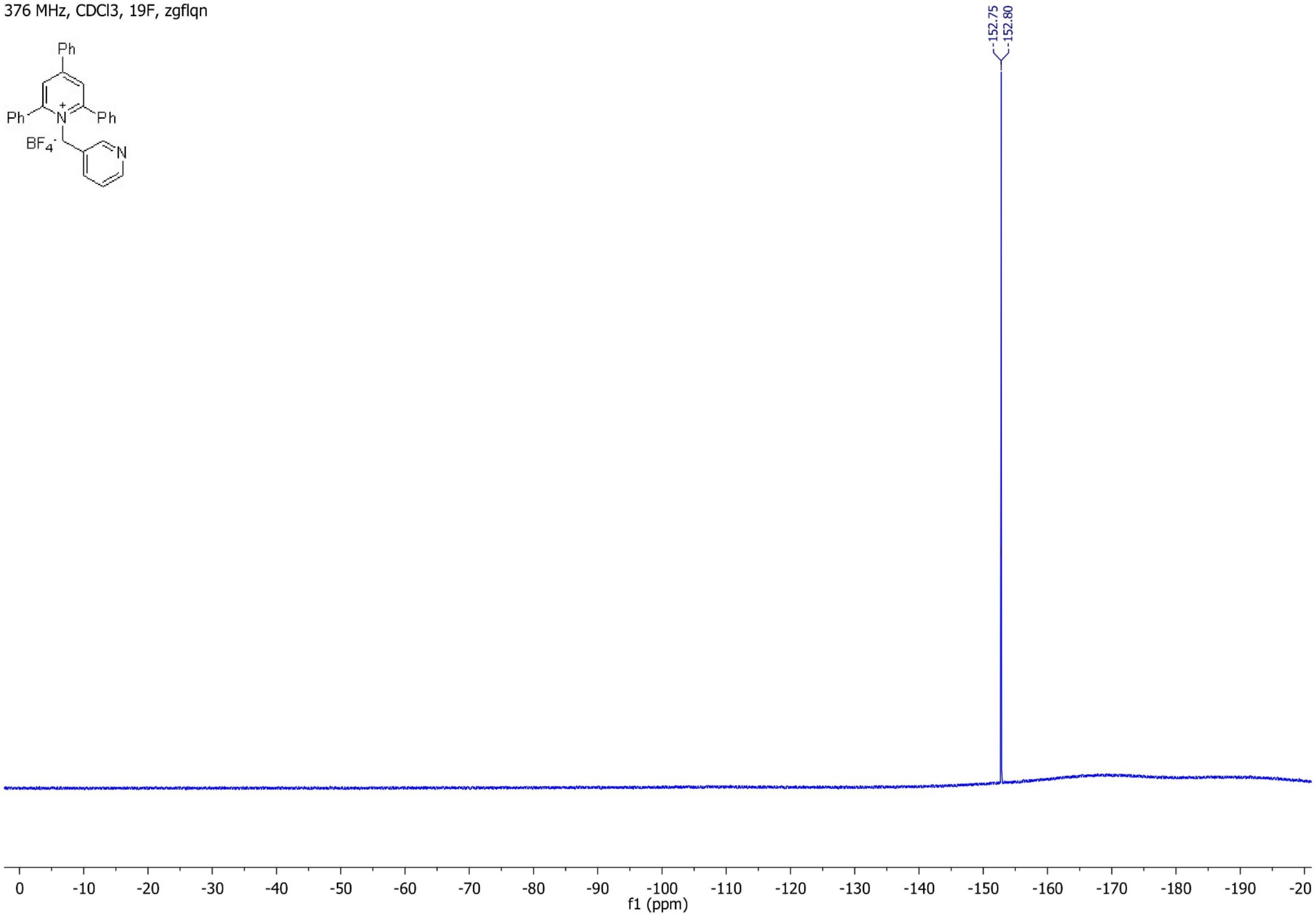

376 MHz, CDCl3, 19F, zgflqn
Ph
Ph
N+
Ph
BF4-
N
-152.75
-152.80
0
-10
-20
-30
-40
-50
-60
-70
-80
-90
-100
-110
-120
-130
-140
-150
-160
-170
-180
-190
-20
f1 (ppm)

1-Allyl-2,4,6-triphenylpyridin-1-ium tetrafluoroborate tetrafluoroborate (S15)

600 MHz, CDCl3, 1H, zg30

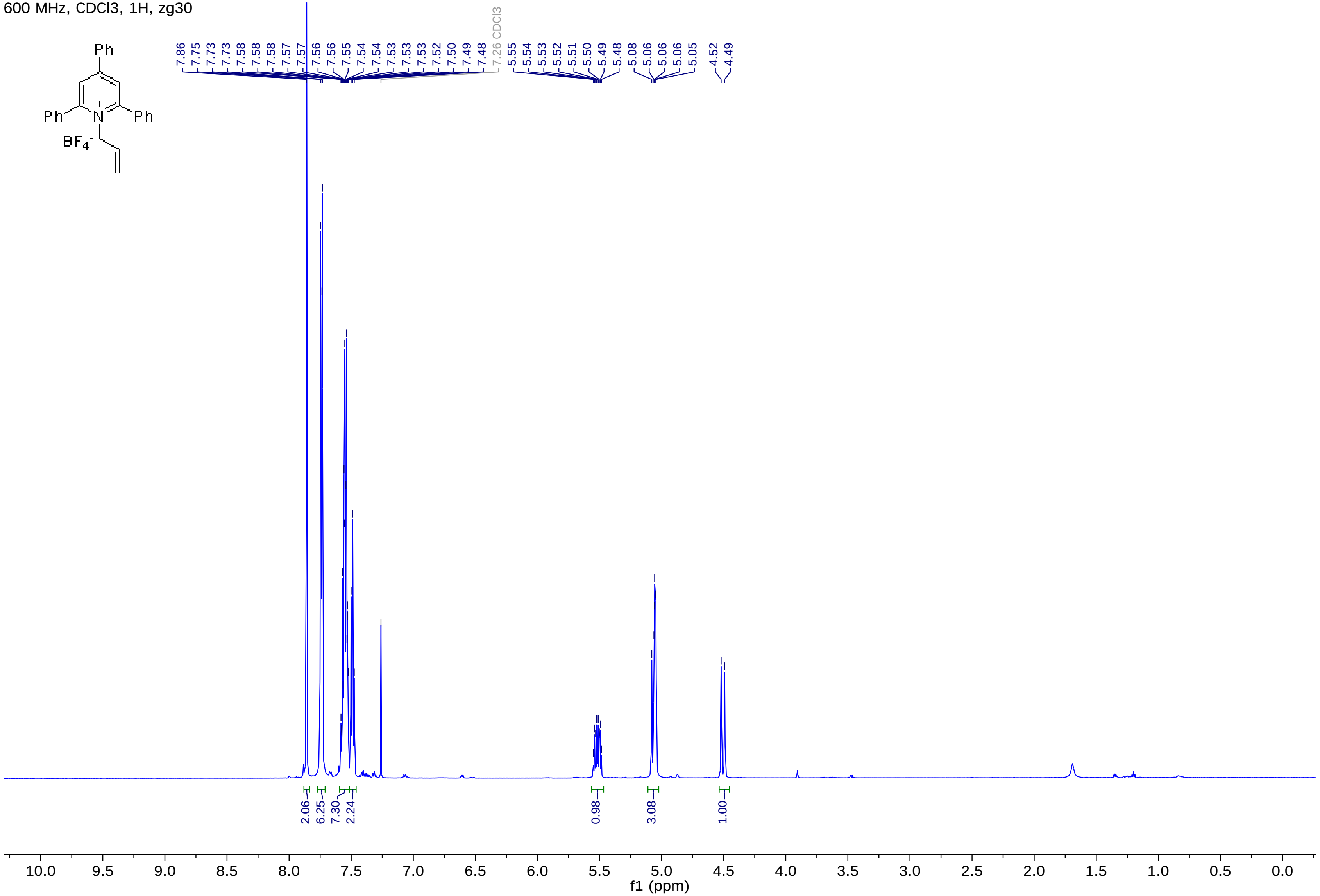

151 MHz, CDCl3, 13C, zgpg30

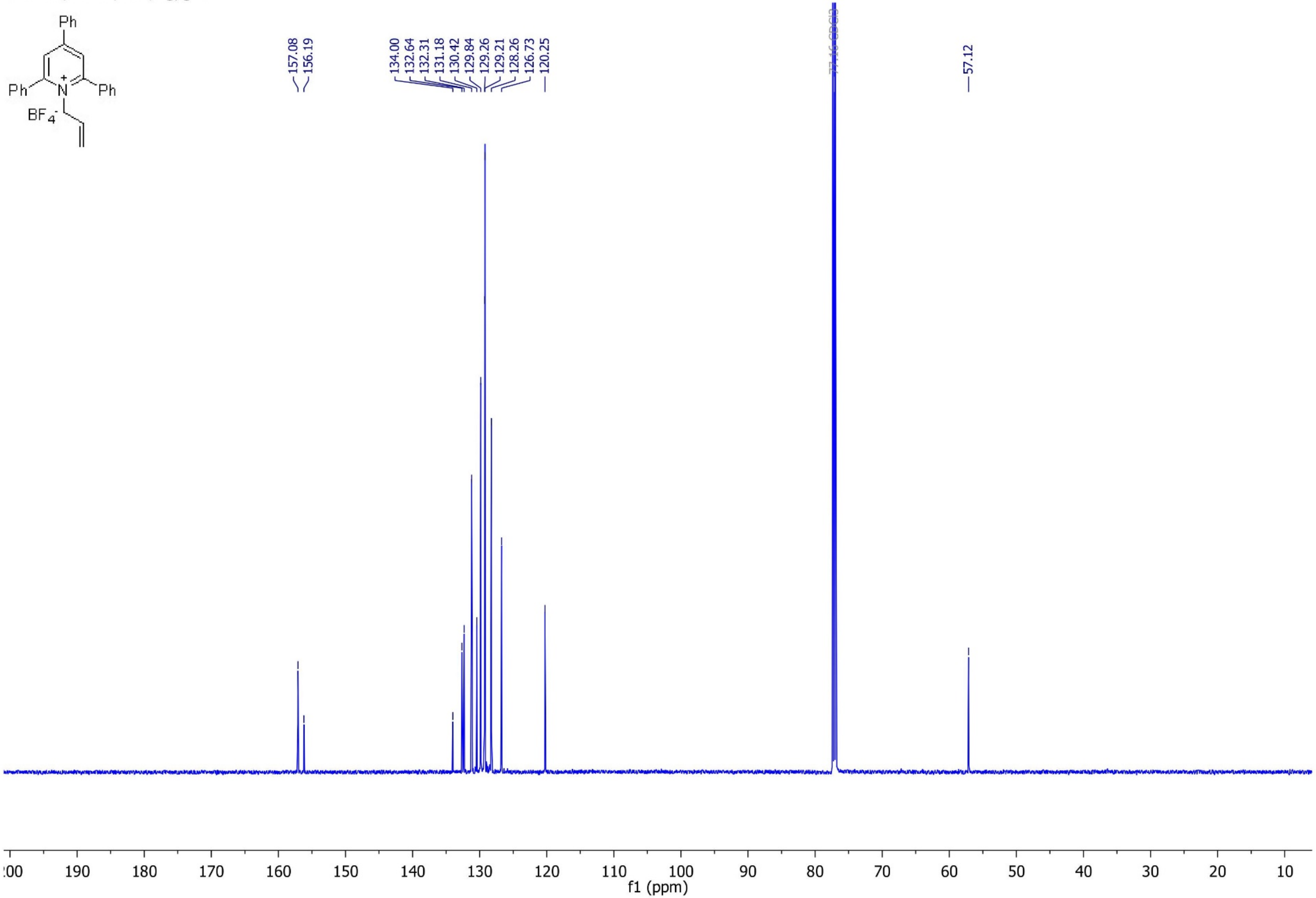

565 MHz, CDCl3, 19F, zgflqn

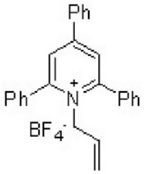

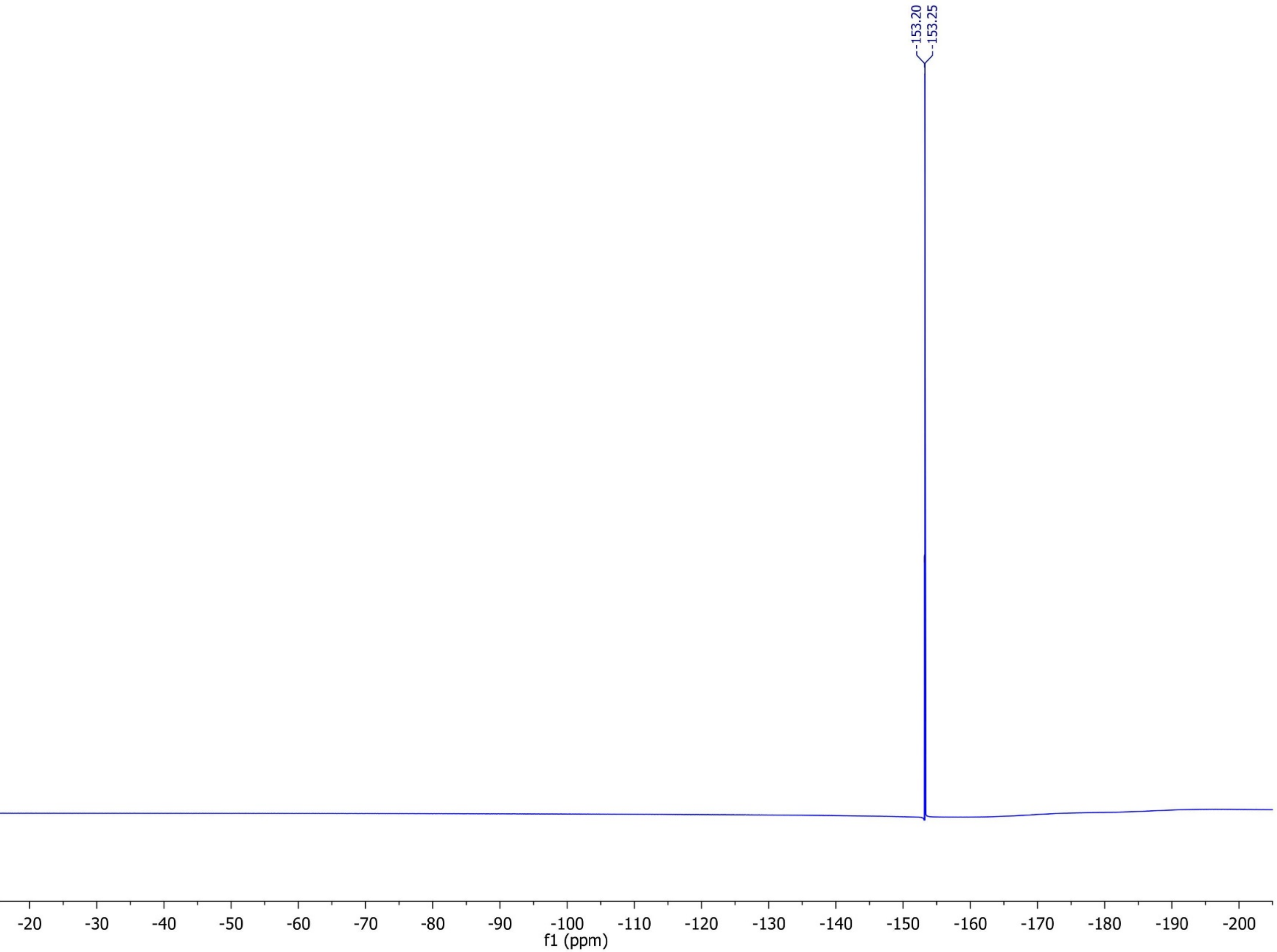

2,4,6-Triphenylpyrylium tetrafluoroborate (S16)

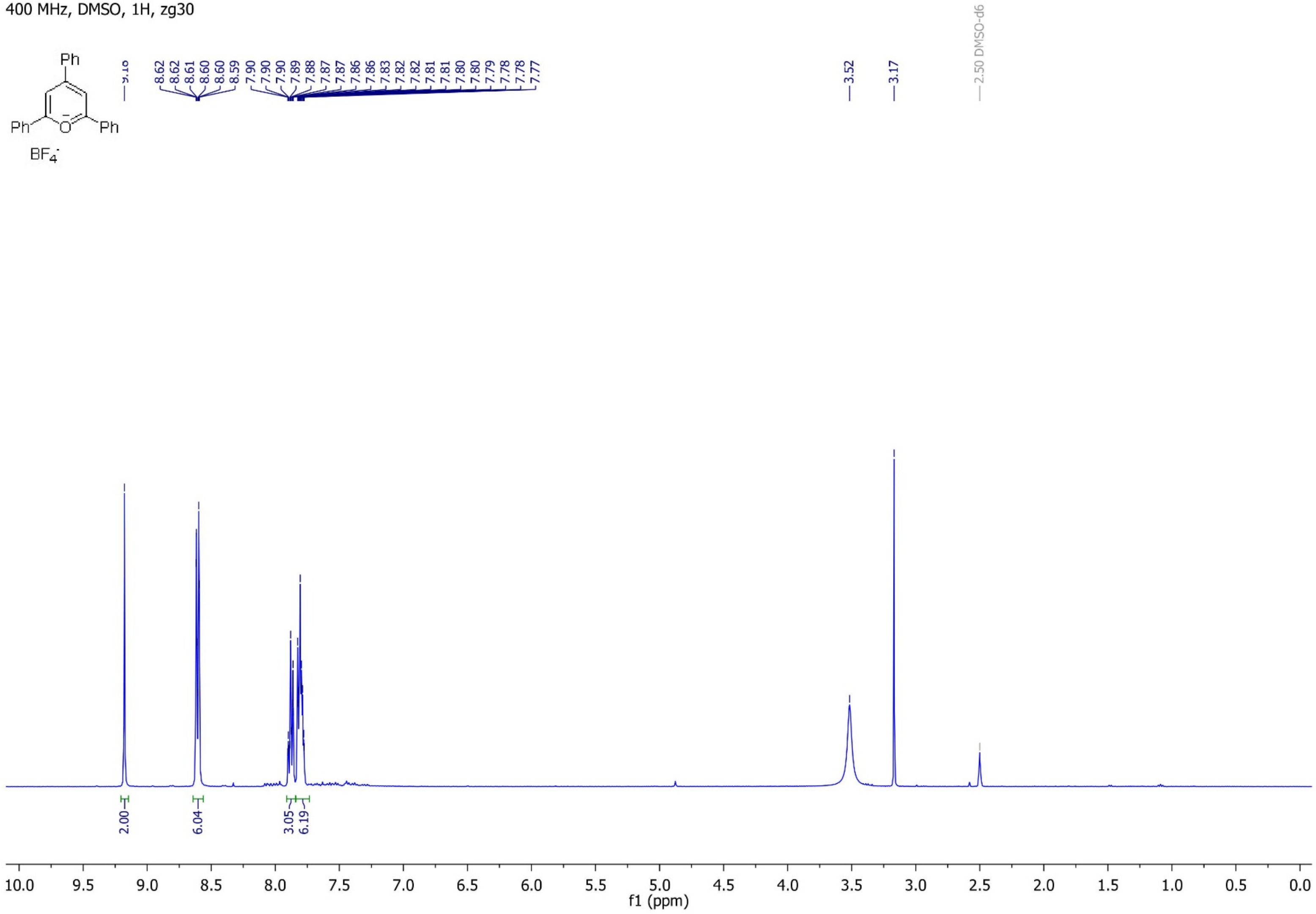

101 MHz, DMSO, 13C, zgpg30

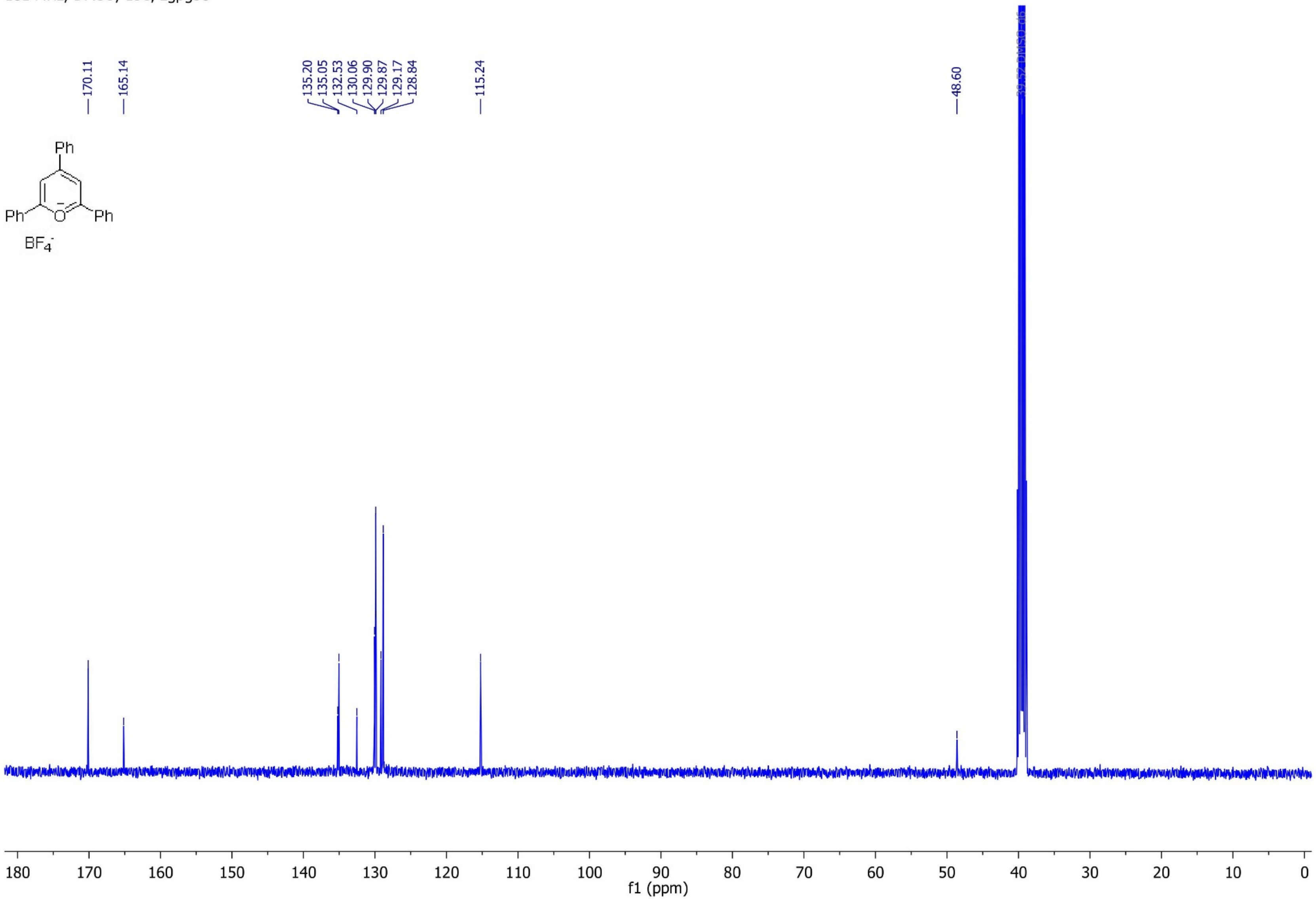

376 MHz, DMSO, 19F, zgflqn

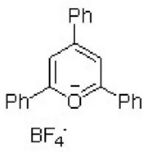

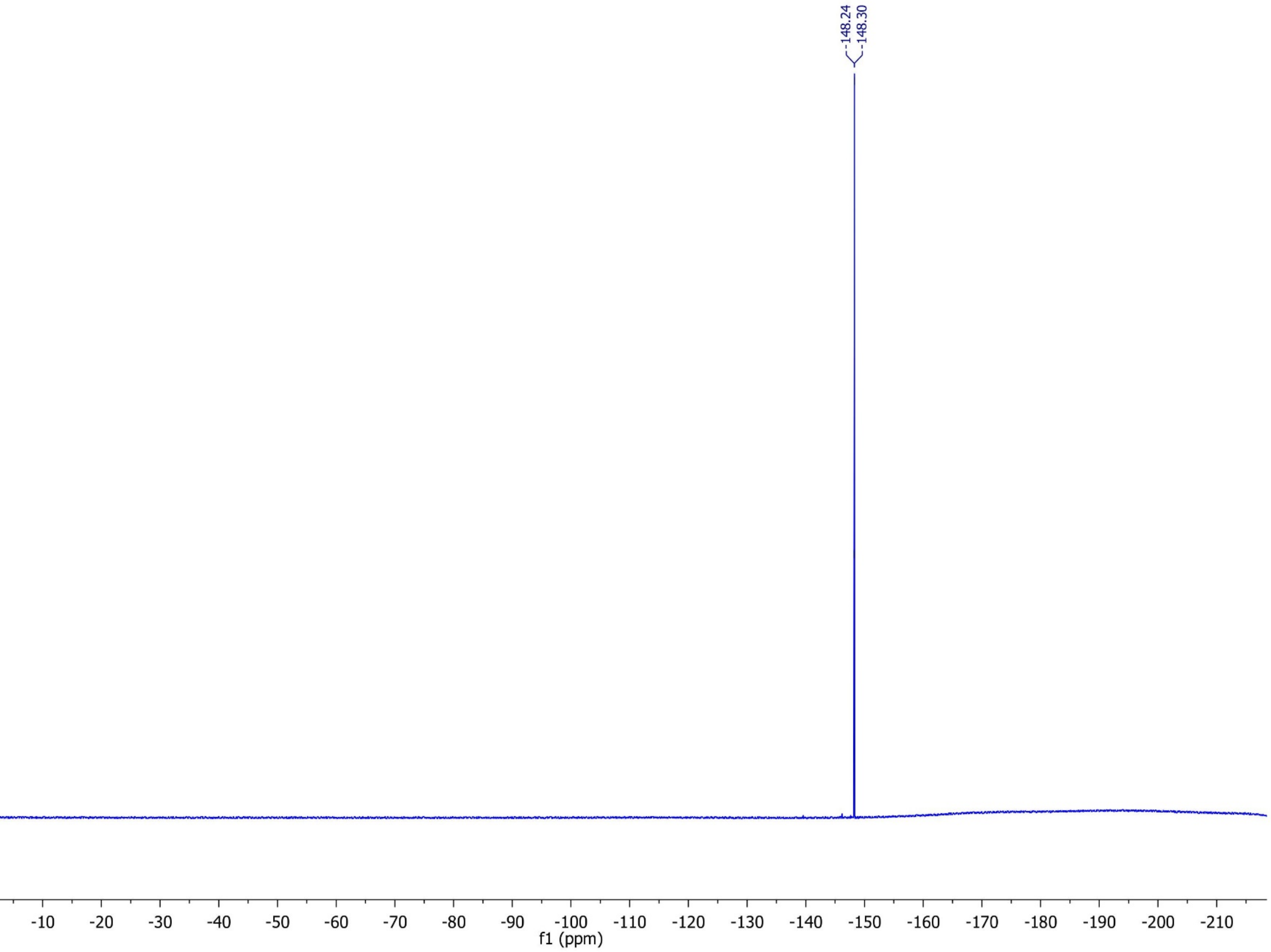

Sideproduct of 1-benzyl-2-(4-chlorophenyl)-1,2,3,4-tetrahydroisoquinoline (21a)

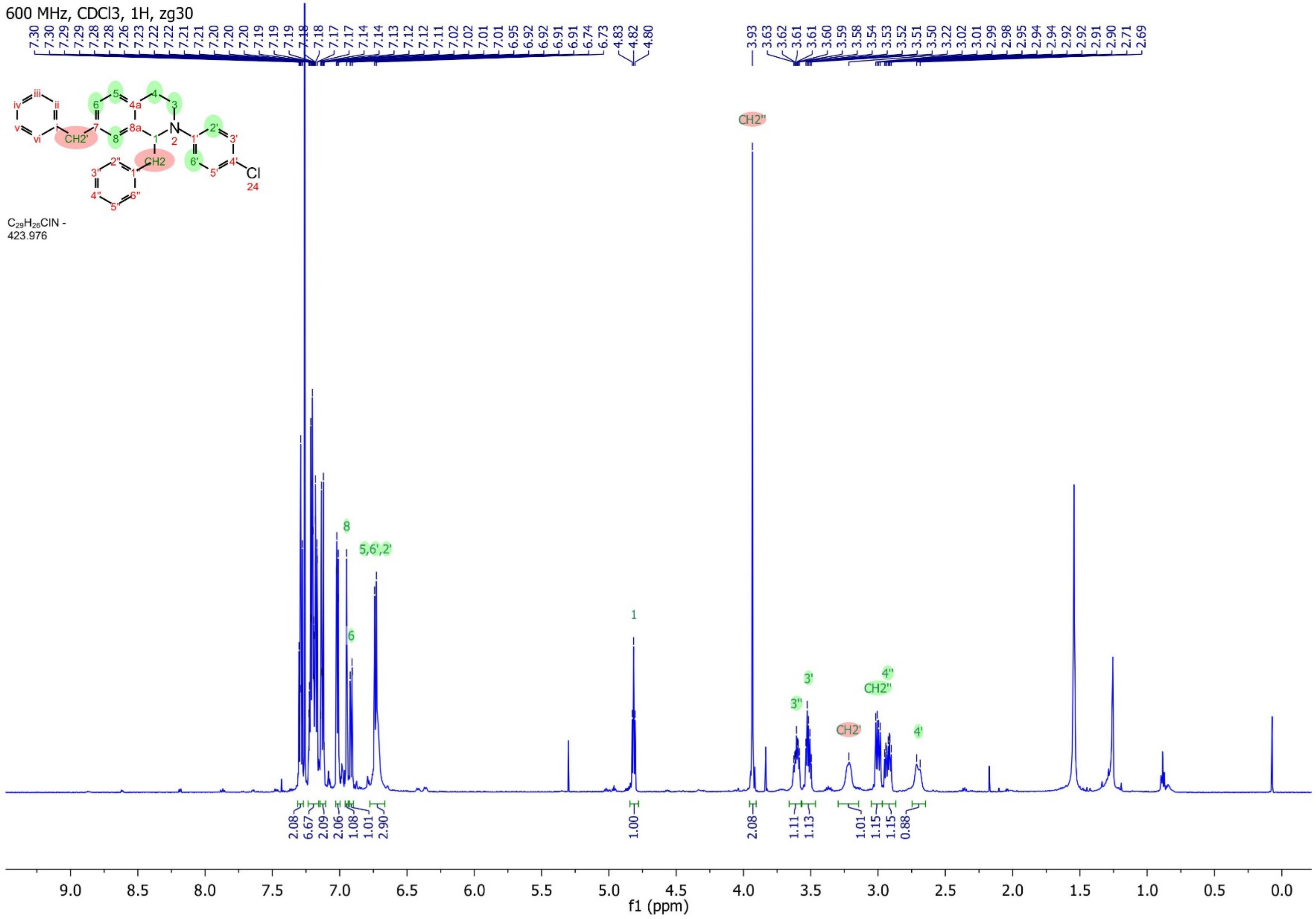

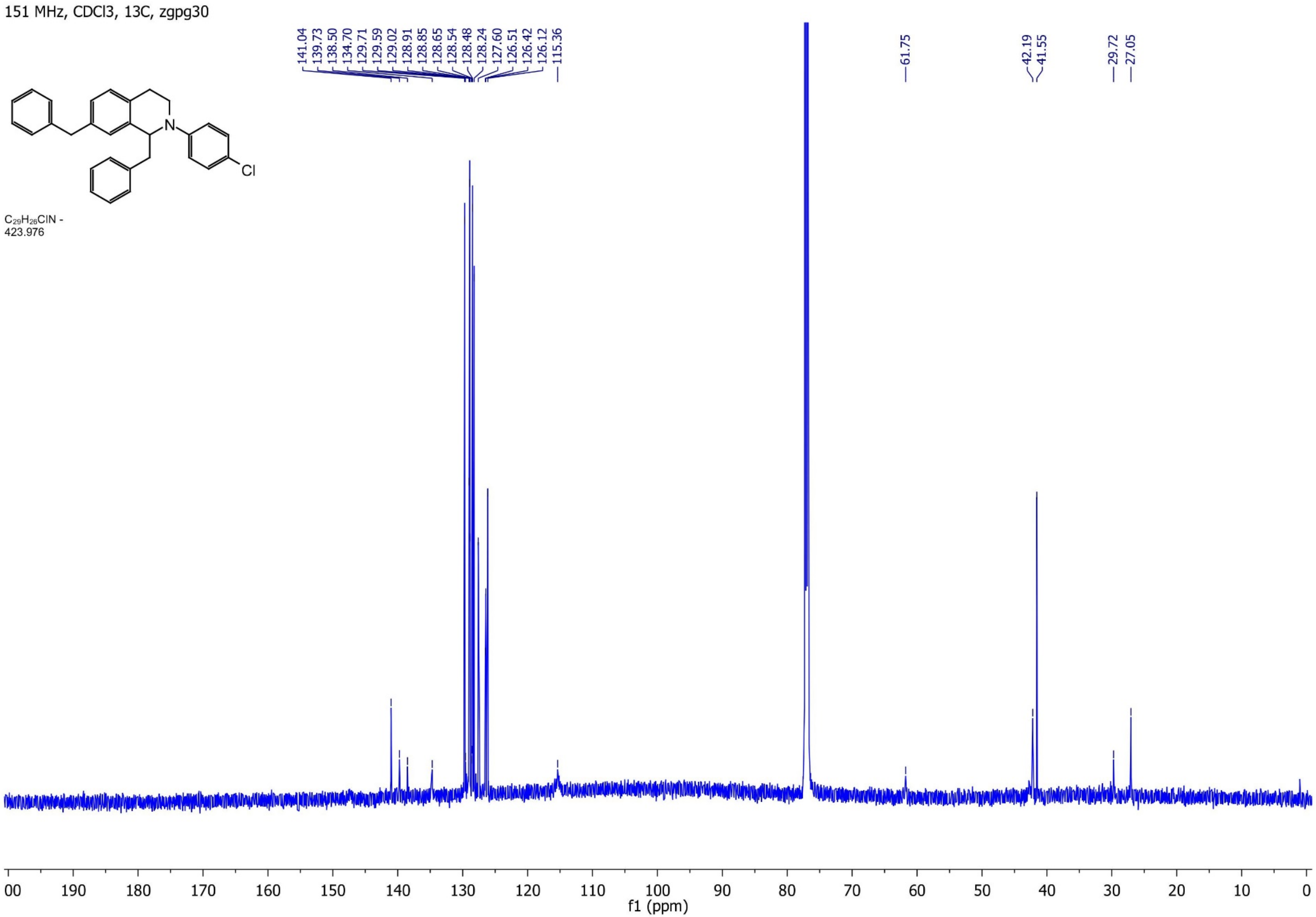
151 MHz, CDCl3, 13C, zgpg30
141.04
139.73
138.50
134.70
129.71
129.59
129.02
128.91
128.85
128.65
128.54
128.48
128.24
127.60
126.51
126.42
126.12
115.36
61.75
42.19
41.55
29.72
27.05
N
Cl
C29H26ClN -
423.976
f1 (ppm)

600 MHz, CDCl3, 1H, hsqcedetgpsisp2.3

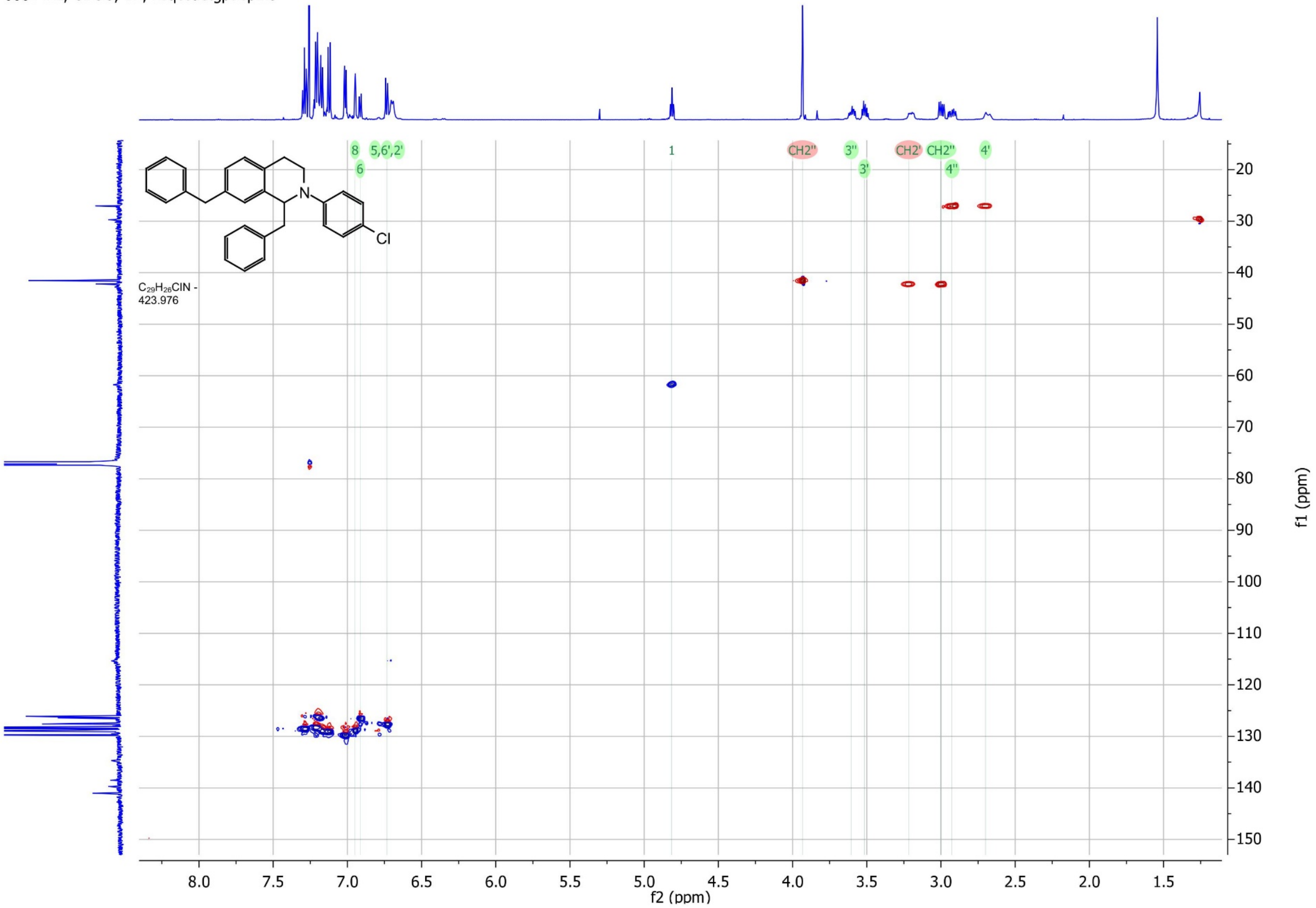

500 MHz, CDCl3, 1H, cosygpppqf

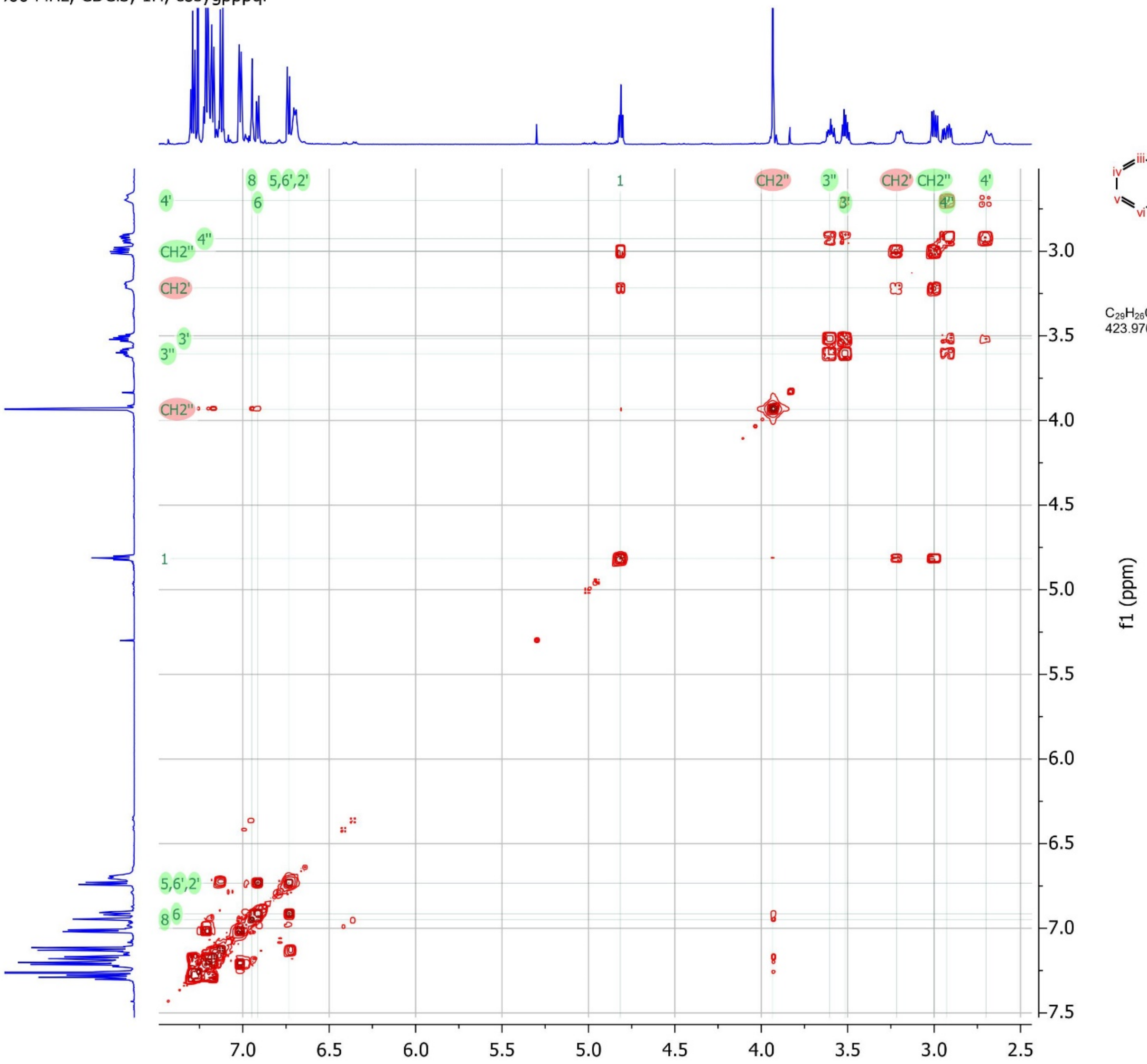

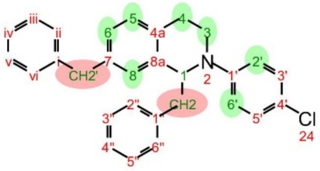

$C_{29}H_{26}ClN$ -
423.976

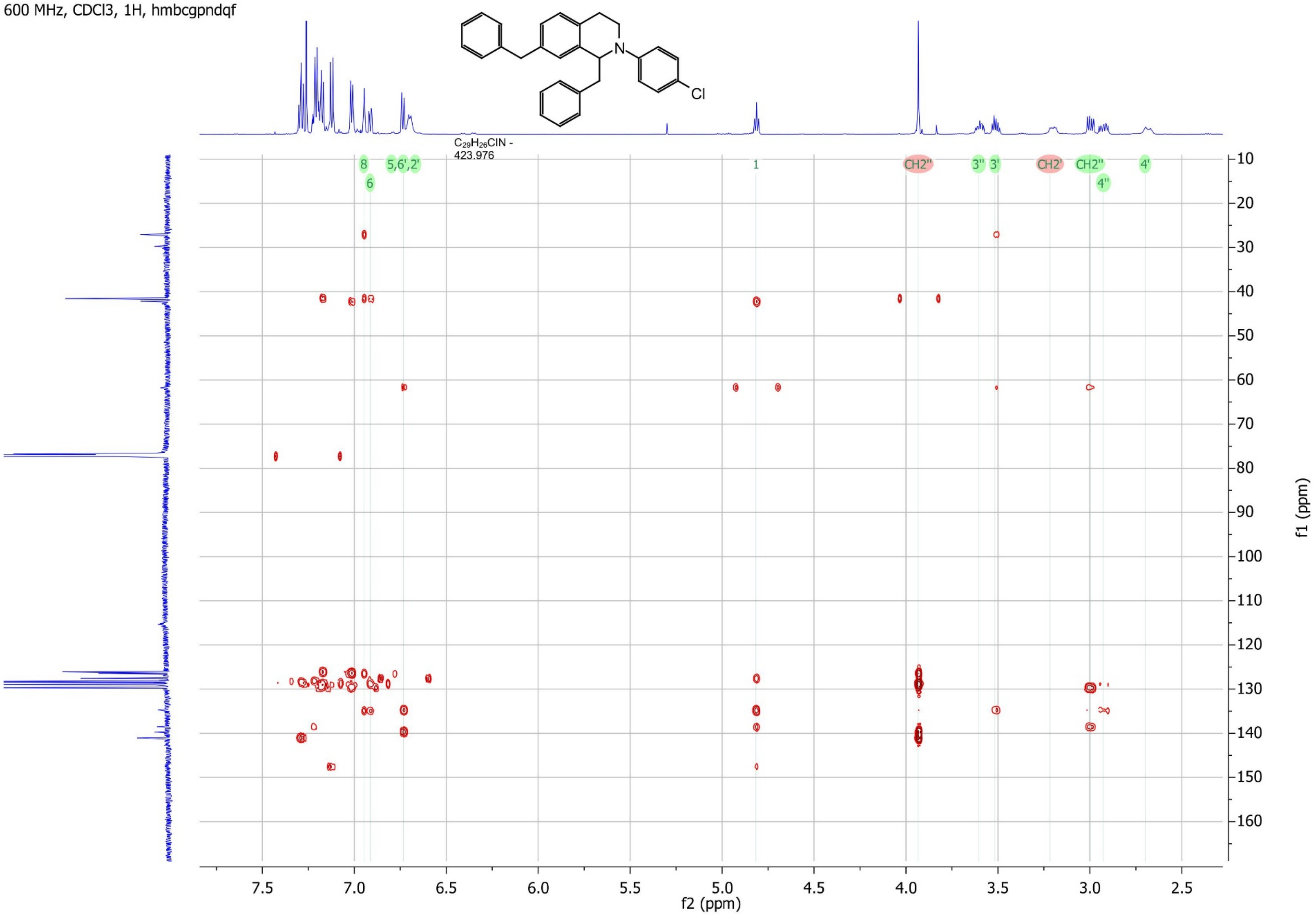
600 MHz, CDCl3, 1H, hmbcgpndqf
Cl
N
C29H26ClN - 423.976
8
5,6',2'
6
1
CH2''
3''
3'
CH2'
CH2''
4''
4'
f2 (ppm)
f1 (ppm)
